# The Use of Benzoin as a Privileged Structure: Synthesis, Characterization, Crystalline Form and an In Vitro Biological Evaluation of 1,2-Diphenyl-2-[1,2,3]triazol-1-yl-ethanol Derivatives

**DOI:** 10.3390/molecules31010170

**Published:** 2026-01-01

**Authors:** Noé Martínez-Romero, Mario Valle-Sánchez, Marco A. García-Eleno, Carlos A. González-González, David Corona-Becerril, Lizbeth Triana-Cruz, Diego Martínez-Otero, María Teresa Ramírez-Apan, David Morales-Morales, Jorge Andrés Ornelas-Guillén, Erick Cuevas-Yañez

**Affiliations:** 1 Centro Conjunto de Investigación en Química Sustentable UAEM-UNAM, Universidad Autónoma del Estado de México, Carretera Toluca-Atlacomulco Km 14.5, Toluca 50200, Estado de México, Mexico; nmartinezr@uaemex.mx (N.M.-R.); marvals_18@hotmail.com (M.V.-S.); magarciae@uaemex.mx (M.A.G.-E.); dcoronab@uaemex.mx (D.C.-B.); ltrianac@unam.mx (L.T.-C.); diegomtz@unam.mx (D.M.-O.); 2 Facultad de Química, Universidad Autónoma del Estado de México, Paseo Colon esq. Paseo Tollocan, Toluca 50120, Estado de México, Mexico; cagonzalezg@uaemex.mx; 3 Instituto de Química, Universidad Nacional Autónoma de México, Circuito Exterior S. N., Ciudad Universitaria, Coyoacán 04510, Ciudad de México, Mexicodamor@unam.mx (D.M.-M.); 4 Instituto de Investigaciones Químico-Biológicas, Universidad Michoacana de San Nicolás de Hidalgo, Ciudad Universitaria, Av. Fco. J. Múgica s/n, Morelia 58030, Michoacán, Mexico

**Keywords:** 1,2,3-triazole, click chemistry, benzoin, crystal structure, antimicrobial activity, cytotoxic activity

## Abstract

A collection of 40 derivatives of 1,2-diphenyl-2-[1,2,3]triazol-1-yl-ethanol was obtained through a sequence of reactions, starting with benzoin as the initial raw material and using the CuAAC reaction as the key step in this process. The structure of a pair of these compounds was ultimately corroborated by single-crystal X-ray diffraction studies, which also reveals important O-H···N interactions. The antimicrobial activity of synthesized 1,2,3-triazoles was assessed against strains that include *Candida albicans* and *Staphylococcus aureus*. The antiproliferative properties of some of these novel compounds were also tested using a variety of tumor cell lines, including U251 (human glioblastoma), PC-3 (human prostate cancer cell line), K562 (human leukemia), HCT-15 (human colorectal adenocarcinoma), MCF-7 (human breast adenocarcinoma), and SKLU (human lung adenocarcinoma).

## 1. Introduction

By definition, a privileged structure is a specific arrangement of atoms that exhibits a high affinity for binding to various biological molecules, often resulting in significant biological activity. These structures are considered “privileged” because they facilitate the discovery of new drugs by serving as templates or scaffolds for synthesizing compounds with therapeutic potential [1]. Some important examples of privileged structures include chalcones [2], quinones [3], curcuminoids [4,5] and morpholine [6].

**Scheme 1:**
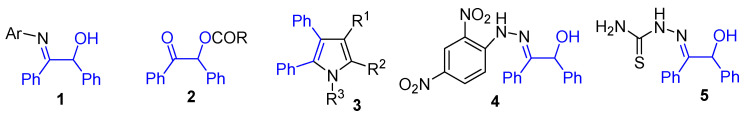
Structures of biologically active benzoin derivatives **1**–**5**. The parts of these molecules derived from benzoin are highlighted in blue.

On the other hand, benzoin and its derivatives constitute a relatively unexplored yet auspicious reservoir of biologically active molecules, exhibiting noteworthy antimicrobial, antioxidant and antitumor properties [7,8,9,10,11,12]. Moreover, benzoin is used as a framework in the preparation of pyrroles and oxazoles [13,14,15,16]. Some examples of biologically active benzoin derivatives, see Scheme 1, include Schiff Bases **1** and esters **2** with antiproliferative properties [10,11,17], potential antimicrobial and antioxidant pyrroles **3** [18], antimicrobial hydrazones **4** [19,20] as well as thiosemicarbazones **5** with antibacterial properties [21]. These characteristics hint at the potential use of benzoin as a privileged structure.

In another context, a widely used strategy in chemical synthesis is the copper-catalyzed azide-alkyne cycloaddition (CuAAC) which enables the fast and straightforward preparation of chemical libraries under mild conditions [22,23,24]. Through this approach, several biologically active molecules have been obtained, highlighting the development of 1,4-disubstituted-1,2,3-triazoles with antimicrobial [25,26,27,28,29,30,31,32,33,34,35,36] and antitumoral [37,38,39,40,41,42,43] properties.

Considering these facts, as well as our experience with the CuAAC reaction/Click Chemistry applied to the synthesis of biologically active 1,2,3-triazoles [44,45,46], we envisioned the benzoin structure as a molecular scaffold for preparing a novel family of 1,2,3-triazoles bearing to a 1,2-diphenyl motif. In this report, we disclose our most recent findings in this area, which we have outlined in the following sections.

## 2. Results and Discussion

**Scheme 2:**
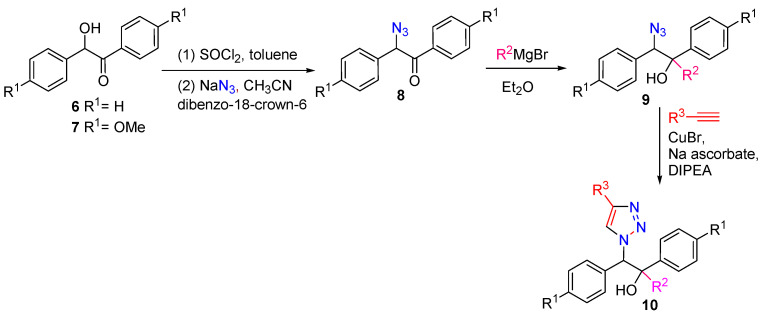
Synthetic route of 1,2-diphenyl-2-[1,2,3]triazol-1-yl-ethanol derivatives **10**.

A synthetic route for the titled compounds was designed using benzoin **1** as the starting material, as sketched in Scheme 2. In this route, benzoin **6** (R^1^ = H) was treated with thionyl chloride followed by sodium azide to give 2-azido-1,2-diphenyl-ethanone **8** which in turn was reacted with a series of alkyl magnesium bromides affording the respective 1-alkyl- 2-azido-1,2-diphenylethanol derivatives **9** that generate, under treatment with diverse alkynes and a CuI/DIPEA catalytic system, the corresponding 1,2-diphenyl-2-[1,2,3]triazol-1-yl-ethanol derivatives **10** (compounds **11**–**30**) in yields ranged from 40% to 80%, see Table 1. Compounds **31**–**50** were prepared using a similar synthetic sequence from *p*-anisoin **7** (R^1^ = OMe), and their behavior was comparable to that of benzoin.

The 1,2,3-triazoles **11**–**50** were fully characterized using conventional spectroscopic techniques. These techniques provided a set of signals that allowed for the structural elucidation of the compounds. Besides the signals in the NMR spectra that are characteristic of the 1,2,3-triazole ring—such as the appearance of a singlet signal corresponding to the hydrogen on triazole C-5 in the range of δ 7.4 to 8.8 ppm in the corresponding ^1^H NMR spectra—the ^1^H NMR spectra of these compounds also exhibit a singlet signal at approximately δ 5.5–6.0 ppm. This signal is associated with the methyne group bound to the N1 nitrogen atom of the 1,2,3-triazole core and to one of the phenyl groups.

Additionally, two characteristic signals appear in the ^13^C NMR spectra at approximately δ 146 and 121 ppm, which are assigned to C4 and C5 of the triazole moiety. Another noteworthy spectroscopic feature that distinguishes triazoles **11**–**50** is the presence of a quaternary aliphatic carbon signal at δ 79 ppm and a methyne carbon signal at δ 72 ppm in the respective ^13^C NMR spectra. Due to these signals, it is possible to generalize spectroscopically, allowing for the appropriate recognition of this novel class of 1,2,3-triazoles.

**Figure 1:**
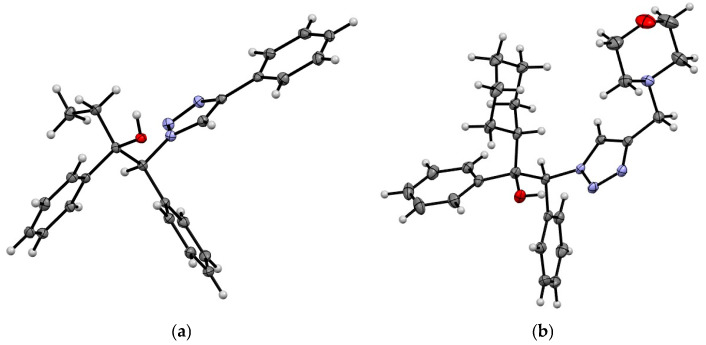
ORTEP diagrams for compounds **11** (**a**) and **30** (**b**).

In this respect, compounds **11** and **30** were found to be crystalline solids, which were subjected to X-ray crystallography analysis, thereby substantiating the proposed structural model for these compounds. The ORTEP representations of compounds **11** and **30** are plotted in Figure 1 and the corresponding crystallographic data and structural refinement parameters are presented in Table 2.

**Figure 2:**
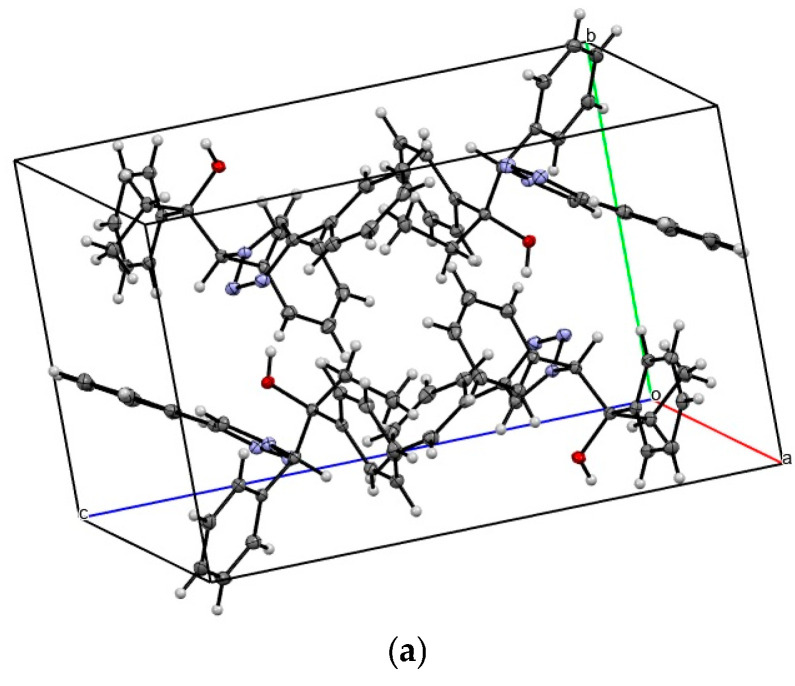

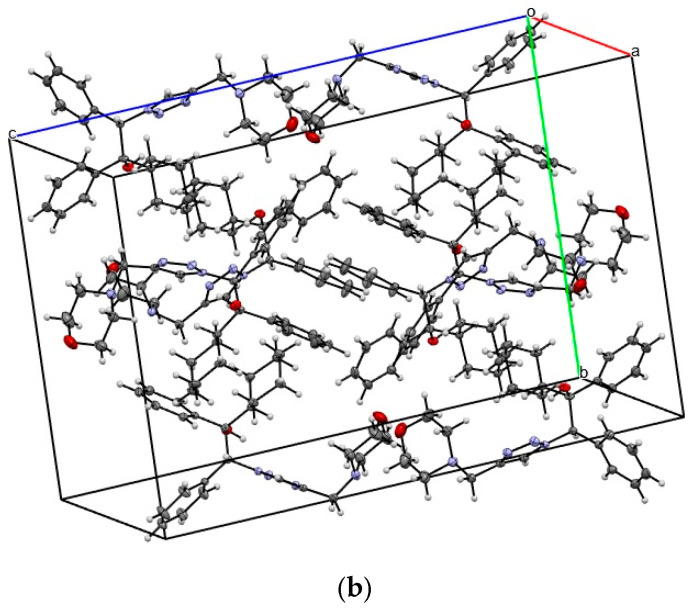
Crystal packing of compounds **11** (**a**) and **30** (**b**).

The crystals of triazoles **11** and **30** belong to the monoclinic system with nonpolar, centrosymmetric phases. Compound **6** belongs to the *P*2_1/c_ space group, and compound **30** belongs to the *P*2_1/n_ space group. This means that for every atom in the structure, there is an identical atom located on the opposite side of the center of symmetry. These arrangements can be seen in the crystal packings projected in Figure 2. Consequently, the respective crystalline structures lack a permanent electric dipole moment because their unit cells have an inversion center. This behavior has been well-documented in some imines [47] and is likely due to the presence of racemic mixtures in these compounds [48].

**Figure 3:**
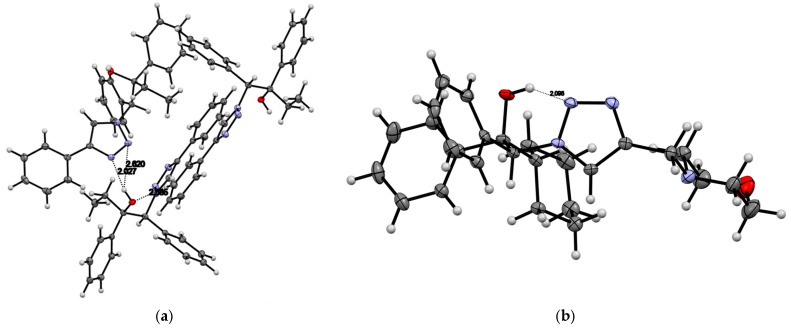
O-H···N interactions in compounds **11** (**a**) and **30** (**b**).

The crystal structures of triazoles **11** and **30** reveal significant O-H···N interactions that play a key role in shaping each structure. Compound **11** exhibits two important intermolecular interactions: one between the hydroxyl hydrogen atom and the N2 nitrogen atom, O1-H···N2 = 2.620 Å, and a second, closer intermolecular interaction between the hydroxyl hydrogen atom and the N3 nitrogen atom of the triazole moiety, O1-H···N3 = 2.027 Å, see Figure 3a. Compound **30**, in contrast, has an intramolecular interaction O1-H···N2 = 2.096 Å as depicted in Figure 3b.

The cytotoxic activity of 20 selected 1,2,3-triazoles was evaluated by quantifying their inhibitory activity on the growth of seven cell lines listed below: U251 = tumor cells derived from malignant glioblastoma, PC-3 = cell line derived from metastatic prostate cancer, K562 = cell line derived from myeloid leukemia, HCT-15 = cell line derived from colorectal adenocarcinoma, MCF-7 = cell line derived from breast cancer, SKLU-1 = cell line derived from lung adenocarcinoma and COS-7 = monkey kidney cell line (non-cancerous). Cytotoxic activity was assessed using the microcell culture method, which determines the endpoint of cell growth by staining with sulforhodamine B (SRB), a cell protein affinity dye, as described in the National Cancer Institute (NCI) protocols [49].

The results of the cytotoxicity tests are listed in Table 3. As can be seen, although almost all the evaluated compounds exhibit some degree of cytotoxicity in healthy, non-cancerous monkey kidney fibroblasts (COS-7), which makes them unsuitable in their current form as anticancer agents, some interesting trends can be established that may serve as a guide for the development of similar molecules. In this sense, it is worth noting that a significant decrease in cell inhibition percentage is observed for almost all triazoles with butyl alkyl chains compared to their ethyl alkyl chain counterparts in the U251, PC-3, and K562 cell lines. However, the opposite trend occurs with the HCT-15, MCF-7, SKLU-1, and COS-7 cell lines, since triazoles with longer chains generally show higher cell growth inhibition activity. Another direct comparison between the structure and cell growth inhibition percentage is promoted by the presence of methoxy substituents in compounds **31**–**40** derived from anisoin, where a decrease in cell growth inhibitory activity is observed compared to their much less polar counterparts derived from benzoin.

The antimicrobial activity of the synthesized 1,2,3-triazole derivatives was evaluated against *Candida albicans* and *Staphylococcus aureus* ATCC 29213, two clinically relevant pathogens frequently used as reference strains in antifungal and antibacterial susceptibility tests. The agar diffusion method was performed following the CLSI guidelines, using ketoconazole (25 µg/mL) and amikacin (30 µg) as positive controls, and methanol as the negative control [50]. The inhibition halos were measured after 24–48 h of incubation at 30 °C. The results of the antimicrobial activity tests are described only for the compounds that displayed inhibitory activity. The rest of the compounds were analyzed, and none of them showed significant activity.

**Figure 4:**
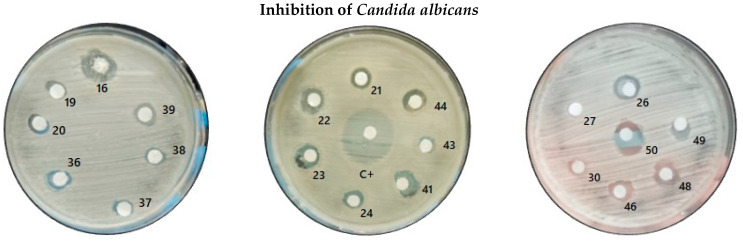

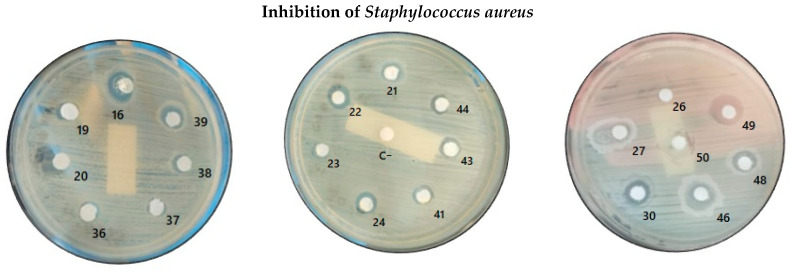
Diameters of inhibition zones of *C. albicans* and *S. aureus* culture on inoculation of selected 1,2,3-triazoles.

The antimicrobial activity from the family of compounds **16**–**20** and **36**–**40** (R^2^ = butyl) is of particular interest. As shown in Figure 4 and Table 4, compound **16** displayed the largest diameters of zone of inhibition (DIZ) against *C. albicans* (15 mm), comparable to the positive control ketoconazole (15 mm at 25 µg/mL). Additionally, this compound exhibited antibacterial activity against *S. aureus* (11 mm), suggesting a dual effect that positions it as a promising candidate for further studies. Other members of this group (**19**, **20**, **36**–**39**) exhibited lower or moderate inhibition, ranging from 6 to 10 mm. Regarding a class of triazoles with R^2^ = Bn, several compounds demonstrated moderate antifungal effects. Compounds **22**, **23**, and **24** produced DIZ = 10 and 12 mm against *C. albicans*, whereas their activity against *S. aureus* was less pronounced, generally not exceeding 8 mm. This indicates that structural features in this family may favor antifungal rather than antibacterial activity.

Interestingly, the activity of 1,2,3-triazoles **46**–**50** derived from 1-azido-1,2-bis-(4-methoxy-phenyl)-5-methyl-hexan-2-ol (R^1^ = OMe, R^2^ = CH_2_CH_2_CH(CH_3_)), especially **50**, suggests a promising dual antifungal–antibacterial effect, with inhibition halos consistently reaching 13 mm in both *C. albicans* and *S. aureus*. Similarly, compound **46** displayed dual activity (DIZ = 10 mm for fungi and 13 mm for bacteria). These results highlight the potential of this sort of compounds as a source of broad-spectrum antimicrobial agents. This dual action aligns with a previous work by Sari and coworkers, who reported the antibacterial activity of certain azole derivatives against *S. aureus* and *E. faecalis* with MIC < 1 µg/mL and negligible cytotoxicity. They proposed a mechanism involving the inhibition of bacterial flavohemoglobins, which is supported by docking studies [51]. This supports the potential expansion of azole applications beyond classical antifungal therapy.

In contrast, ethyl derivatives, compounds **11**–**15** and **31**–**35**, showed limited activity. Only compound **15** exhibited detectable inhibition (DIZ = 8 mm for fungi, DIZ = 6 mm for bacteria), while the rest of the series were inactive under the experimental conditions tested. Similarly, cyclohexyl derivatives **26**–**30** presented weak to moderate inhibition, with inhibition halos ranging from 5 to 10 mm.

These results confirm that the synthesized 1,2,3-triazole derivatives exhibit variable antimicrobial effects depending on their structural family. Molecules with butyl and s-butyl substituents demonstrated the highest potential, with compounds **16** and **50** showing inhibition halos comparable to reference drugs. The activity against *C. albicans* was more consistent across families, while only selected derivatives displayed notable antibacterial effects. These findings suggest that structural optimization of triazole families with butyl and s-butyl substituents could further enhance their antimicrobial efficacy and support their candidacy for subsequent biological evaluations. Overall, these results confirm that the inhibitory potential of triazole-derived compounds depends strongly on structural modifications and their interaction with microbial targets. Molecules with butyl and s-butyl substituents display comparable inhibition levels to ketoconazole, which has dual antimicrobial effects. These molecules are promising candidates for further optimization and integration into drug delivery platforms.

Although it is difficult to make a comparison of the antimicrobial activity of the synthesized triazoles with respect to other benzoin derivatives due to the different protocols used for the evaluation of biological activity, it can be seen in general terms that the presence of the 1,2,3-triazole ring increases the antibacterial and antifungal activity compared to moieties [19,20,21].

These examples demonstrate the synthetic value of the benzoin structure as a scaffold and departure point for obtaining new molecules with biological activity. To our knowledge, these are the first 1,2,3-triazoles derived from 1,2-diaryl-2-azido-1-alkyl-ethan-1-ol prepared via the CuAAC reaction from benzoin/anisoin in a sequence of only 4 reaction steps.

## 3. Materials and Methods

### 3.1. General Remarks

The starting materials were purchased from Aldrich Chemical Co. (Milwakee, WI, USA) and were used without further purification. The solvents were distilled before use. Silica plates of 0.20 mm thickness were used for thin layer chromatography. Melting points were determined with a Kruss Optronic melting point apparatus (Kruss GmbH, Hamburg, Germany) and were uncorrected. ^1^H and ^13^C NMR spectra were recorded using a Bruker Avance 300 MHz (Bruker Corporation, Billerica, MA, USA); the chemical shifts (δ) were given in ppm relative to TMS as an internal standard (0.00). For analytical purposes, the mass spectra were recorded on a JEOL AccuTOF JMS-T100LP (JEOL Ltd., Tokyo, Japan) device in the DART mode via a direct inlet probe. IR spectra were recorded on a Bruker Tensor 27 (Bruker Corporation, Billerica, MA, USA). (Compounds spectroscopic data are in Supplementary Materials).

For the X-ray diffraction studies, crystals of compounds **2** and **25** were obtained by the slow evaporation of a dilute AcOEt solution, and the reflections were acquired with a Bruker APEX DUO (Bruker Corporation, Billerica, MA, USA) diffractometer equipped with an Apex II CCD detector, with Mo Kα radiation (λ = 0.71073 A) at 100 K. Frames were collected using omega scans and integrated with SAINT, and multi-scan absorption correction (SADABS) was applied [52]. The structures were solved by direct methods (SHELXS-97) [53]; missing atoms were found by difference-Fourier synthesis and refined on F2 by a full-matrix least-squares procedure using anisotropic displacement parameters using SHELXL [54] and the ShelXle GUI [55]. The hydrogen atoms of the C–H bonds were placed in idealized positions. The molecular graphics were prepared using Mercury (version 2022.3.0) [56] and POV-Ray (version 3.6) [57]. Crystallographic data for the structure reported in this paper have been deposited with the Cambridge Crystallographic Data Centre, CCDC (No. 2517822 for compound **11** and No. 2517823 for compound **30**). Copies of available materials can be obtained free of charge on application to the Director, CCDC, 12 Union Road, Cambridge CB2 IEZ, UK (facsimile: (44) 01223 336033); e-mail: deposit@ccdc.ac.uk).

### 3.2. General Procedure for the Synthesis of 2-Diphenyl-2-[1,2,3]triazol-1-yl-ethanol Derivatives

Typical procedure. The appropriate azido alcohol (1 mmol) was added to a mixture of PEG-400 (3.2 mL) and H_2_O (0.8 mL), which was then gently warmed until the azido alcohol was fully dissolved. CuBr (0.014 g, 0.1 mmol), sodium ascorbate (0.019 g, 0.1 mmol), DIPEA (0.07 mL, 0.051 g, 0.4 mmol) and the corresponding alkyne (1.5 mmol) were added successively and the resulting mixture was stirred at 38–45 °C overnight. The mixture was cooled to room temperature and H_2_O (40 mL) was added. The product was filtered and washed with cold H_2_O. The solid was dissolved with a mixture 1:1 THF/AcOEt (10 mL) and dried over CaCl_2_. The mixture was filtered through a short silica column, the solvent was removed under reduced pressure and the product was purified by crystallization.

*1,2-Diphenyl-1-(4-phenyl-[1,2,3]triazol-1-yl)-butan-2-ol (* **11** *)*

1-Azido-1,2-diphenyl-butan-2-ol and phenylacetylene afforded 1,2-diphenyl-1-(4-phenyl-[1,2,3]triazol-1-yl)-butan-2-ol **11** as a white solid. Yield: 210 mg (57%), m.p. 196–197.5 °C. IR (ATR, cm^−1^): 3315, 3168, 3031, 2961, 2929, 1954, 1879, 1814, 1604, 1444. ^1^H NMR: (300 MHz, DMSO-d_6_) δ = 8.81 (s, 1H), 8.02–7.92 (m, 2H), 7.61–7.52 (m, 2H), 7.57–7.44 (m, 4H), 7.43–7.32 (m, 1H), 7.25 (t, *J* = 7.6 Hz, 2H), 7.23–7.08 (m, 4H), 6.30 (s, 1H), 5.71 (s, 1H), 2.07 (dq, *J* = 14.7, 7.4 Hz, 1H), 1.42 (dt, *J* = 14.3, 7.1 Hz, 1H), 0.59 (t, *J* = 7.3 Hz, 3H). ^13^C NMR: (75 MHz, DMSO-d_6_) δ = 146.59, 143.32, 137.16, 131.30, 130.16, 129.30, 128.28, 128.18, 128.04, 126.77, 126.60, 125.73, 122.00, 79.28, 72.59, 32.84, 8.10. HRMS (DART, [M+1]^+^) *m*/*z* calcd. For C_24_H_24_N_3_O: 370.1919; found: 370.1911.

*1-(4-Cyclopropyl-[1,2,3]triazol-1-yl)-1,2-diphenyl-butan-2-ol (* **12** *)*

1-Azido-1,2-diphenyl-butan-2-ol and cyclopropylacetylene afforded 1-(4-cyclopropyl-[1,2,3]triazol-1-yl)-1,2-diphenyl-butan-2-ol **12** as a white solid. Yield: 206 mg (62%), m.p. 171 °C. IR (ATR, cm^−1^): 3385, 3134, 3091, 2972, 2937, 1741, 1452, 1046, 819. ^1^H NMR: (300 MHz, CDCl_3_) δ = 7.43 (s, 1H), 7.23–6.91 (m, 10H), 5.59 (s, 1H), 3.97 (s, 1H), 2.04–1.79 (m, 2H), 1.46 (dq, *J* = 14.4, 7.3 Hz, 1H), 0.93–0.73 (m, 4H), 0.59 (t, *J* = 7.3 Hz, 3H). ^13^C NMR: (75 MHz, CDCl_3_) δ = 149.69, 141.11, 135.31, 128.87, 127.99, 127.82, 127.69, 126.91, 125.96, 121.75, 79.98, 73.74, 50.70, 32.84, 7.56, 6.68. HRMS (DART, [M+1]^+^) *m*/*z* calcd. For C_21_H_24_N_3_O: 334.1915; found: 334.1917.

*2-[1-(2-Hydroxy-1,2-diphenyl-butyl)-[1,2,3]triazol-4-ylmethyl]-isoindole-1,3-dione (* **13** *)*

1-Azido-1,2-diphenyl-butan-2-ol and 2-prop-2-ynyl-isoindole-1,3-dione afforded 2-[1-(2-hydroxy-1,2-diphenyl-butyl)-[1,2,3]triazol-4-ylmethyl]-isoindole-1,3-dione **13** as a white solid. Yield: 235 mg (52%), m.p.: 148.2–149 °C. IR (ATR, cm^−1^): 3496, 3292, 2969, 2939, 1711, 1395, 1094, 706. ^1^H NMR: (300 MHz, CDCl_3_) δ = 7.84–7.70 (m, 4H), 7.63 (dddd, *J* = 7.9, 6.9, 4.8, 4.0 Hz, 3H), 7.21–6.88 (m, 8H), 5.64 (s, 1H), 4.92 (s, 2H), 4.37 (d, *J* = 2.5 Hz, 1H), 1.94 (dq, *J* = 14.6, 7.3 Hz, 1H), 1.41 (dq, *J* = 14.3, 7.2 Hz, 1H), 0.56 (t, *J* = 7.3 Hz, 3H). ^13^C NMR: (75 MHz, CDCl_3_) δ = 167.68, 142.28, 140.97, 134.12, 132.05, 128.98, 128.02, 127.95, 127.76, 126.96, 125.95, 124.76, 123.49, 79.93, 74.06, 33.02, 27.00, 7.53. HRMS (DART, [M+1]^+^) *m*/*z* calcd. For C_27_H_25_N_4_O_3_: 453.1927; found: 453.1954.

*1-[4-(2,6-Diisopropylphenoxymethyl)-[1,2,3]triazol-1-yl]-1,2-diphenyl-butan-2-ol (* **14** *)*

1-Azido-1,2-diphenyl-butan-2-ol and 1,3-diisopropyl-2-prop-2-ynyloxybenzene afforded 1-[4-(2,6-diisopropylphenoxymethyl)-[1,2,3]triazol-1-yl]-1,2-diphenyl-butan-2-ol **14** as a white solid. Yield: 265 mg (55%), m.p.: 146.5–147.4 °C. IR (ATR, cm^−1^): 3432, 3058, 3032, 2963, 2868, 1740, 1448, 1191, 1036, 698. ^1^H NMR: (300 MHz, CDCl_3_) δ = 7.87 (s, 1H), 7.23–6.92 (m, 10H), 7.03 (s, 3H), 5.74 (s, 1H), 4.89 (s, 2H), 3.73 (s, 1H), 3.29 (p, *J* = 6.9 Hz, 2H), 2.00 (dq, *J* = 14.6, 7.4 Hz, 1H), 1.58–1.41 (m, 1H), 1.12 (d, *J* = 6.9 Hz, 12H), 0.60 (t, *J* = 7.3 Hz, 3H). ^13^C NMR: (75 MHz, CDCl_3_) δ = 152.86, 144.37, 141.90, 141.03, 135.11, 129.01, 128.13, 128.06, 127.86, 127.09, 125.99, 125.10, 124.39, 124.20, 80.04, 74.13, 68.11, 32.19, 26.66, 24.13, 7.60. HRMS (DART, [M+1]^+^) *m*/*z* calcd. For C_31_H_38_N_3_O_2_: 484.2964; found: 484.2955.

*1-(4-Morpholin-4-ylmethyl-[1,2,3]triazol-1-yl)-1,2-diphenyl-butan-2-ol (* **15** *)*

1-Azido-1,2-diphenyl-butan-2-ol and 4-prop-2-ynyl-morpholine afforded 1-(4-morpholin-4-ylmethyl-[1,2,3]triazol-1-yl)-1,2-diphenyl-butan-2-ol **15** as a white solid. Yield: 251 mg (64%), m.p.: 152.7–153.3 °C. IR (ATR, cm^−1^): 3194, 3144, 3066, 3030, 2966, 2930, 2894, 2816, 1741, 1663, 1604, 1558, 1495, 1450, 1210, 1114, 1003, 697. ^1^H NMR: (300 MHz, CDCl_3_) δ = 7.78 (s, 1H), 7.33–6.98 (m, 10H), 5.76 (s, 1H), 3.83 (s, 1H), 3.71 (q, *J* = 3.4 Hz, 6H), 2.57–2.48 (m, 4H), 2.06 (dq, *J* = 14.6, 7.4 Hz, 1H), 1.59–1.40 (m, 1H), 0.67 (t, *J* = 7.3 Hz, 3H). ^13^C NMR: (75 MHz, CDCl_3_) δ = 143.36, 141.05, 135.14, 128.95, 128.06, 127.98, 127.82, 127.01, 125.95, 124.60, 79.95, 73.94, 66.75, 53.62, 53.36, 32.04, 7.56. HRMS (DART, [M+1]^+^) *m*/*z* calcd. For C_23_H_29_N_4_O_2_: 393.2291; found: 393.2955.

*1,2-Diphenyl-1-(4-phenyl-[1,2,3]triazol-1-yl)-hexan-2-ol (* **16** *)*

1-Azido-1,2-diphenyl-hexan-2-ol and phenylacetylene afforded 1,2-diphenyl-1-(4-phenyl-[1,2,3]triazol-1-yl)-hexan-2-ol **16** as a white solid. Yield: 138 mg (35%), m.p.: 184.4–185.2 °C. IR (ATR, cm^−1^): 3463, 3142, 3030, 2959, 2930, 2863, 1955, 1882, 1768, 1448, 1159, 761, 697. ^1^H NMR: (300 MHz, CDCl_3_) δ = 8.00 (d, *J* = 2.5 Hz, 1H), 7.84–7.75 (m, 2H), 7.35 (t, *J* = 7.4 Hz, 2H), 7.31–6.95 (m, 11H), 5.74 (d, *J* = 2.7 Hz, 1H), 3.76 (d, *J* = 4.7 Hz, 1H), 2.00 (ddd, *J* = 16.7, 12.4, 4.5 Hz, 1H), 1.48 (dd, *J* = 13.6, 4.2 Hz, 1H), 1.24–1.00 (m, 1H), 1.06 (s, 1H), 0.81 (d, *J* = 14.3 Hz, 1H), 0.66 (t, *J* = 7.1 Hz, 3H). ^13^C NMR: (75 MHz, CDCl_3_) δ = 143.36, 141.05, 135.14, 128.95, 128.06, 127.98, 127.82, 127.01, 125.95, 124.60, 79.95, 73.94, 66.75, 53.62, 53.36, 32.04, 7.56. HRMS (DART, [M+1]^+^) *m*/*z* calcd. For C_26_H_28_N_3_O: 398.2232; found: 398.2223.

*1-(4-Cyclopropyl-[1,2,3]triazol-1-yl)-1,2-diphenyl-hexan-2-ol (* **17** *)*

1-Azido-1,2-diphenyl-hexan-2-ol and cyclopropylacetylene afforded 1-(4-cyclopropyl-[1,2,3]triazol-1-yl)-1,2-diphenyl-hexan-2-ol **17** as a white solid. Yield: 218 mg (60%), m.p.: 169–169.5 °C. IR (ATR, cm^−1^): 3177, 3092, 2956, 2869, 1743, 1564, 1449, 1214, 1054, 732, 699. ^1^H NMR: (300 MHz, CDCl_3_) δ 7.42 (s, 1H), 7.23–6.91 (m, 10H), 5.57 (s, 1H), 4.00 (s, 1H), 2.00–1.81 (m, 2H), 1.42 (ddd, *J* = 13.8, 11.7, 4.1 Hz, 1H), 1.28–0.96 (m, 3H), 0.94–0.73 (m, 5H), 0.66 (t, *J* = 7.2 Hz, 3H). ^13^C NMR: (75 MHz, CDCl_3_) δ = 149.66, 141.59, 135.29, 128.87, 128.01, 127.85, 127.70, 126.91, 125.86, 121.78, 79.72, 73.92, 39.12, 25.48, 22.83, 13.95, 7.90, 6.70. HRMS (DART, [M+1]^+^) *m*/*z* calcd. For C_23_H_28_N_3_O: 362.2232; found: 362.2222.

*2-[1-(2-Hydroxy-1,2-diphenyl-hexyl)-[1,2,3]triazol-4-ylmethyl]-isoindole-1,3-dione (* **18** *)*

1-Azido-1,2-diphenyl-hexan-2-ol and 2-prop-2-ynyl-isoindole-1,3-dione afforded 2-[1-(2-hydroxy-1,2-diphenyl-hexyl)-1H-[1,2,3]triazol-4-ylmethyl]-isoindole-1,3-dione **18** as a white solid. Yield: 341 mg (71%), m.p.: 187.8–188.5 °C. IR (ATR, cm^−1^): 3462, 3398, 3292, 3147, 3027, 2949, 2864, 1769, 1705, 1428, 1398, 1093, 935, 699. ^1^H NMR: (300 MHz, CDCl_3_) δ 7.82 (s, 1H), 7.82–7.70 (m, 2H), 7.70–7.56 (m, 2H), 7.21–6.90 (m, 10H), 5.62 (s, 1H), 4.94 (s, 2H), 1.92 (ddd, *J* = 13.7, 11.6, 4.5 Hz, 1H), 1.35 (ddd, *J* = 13.7, 11.7, 4.1 Hz, 1H), 1.24–1.04 (m, 1H), 1.09–0.91 (m, 1H), 1.02 (s, 1H), 0.87–0.69 (m, 1H), 0.76 (s, 1H), 0.61 (t, *J* = 7.2 Hz, 3H). ^13^C NMR: (75 MHz, CDCl_3_) δ = 167.67, 167.00, 142.27, 141.43, 134.91, 134.26, 134.12, 132.05, 128.95, 128.02, 127.95, 127.75, 126.94, 125.81, 124.81, 123.59, 123.48, 79.66, 74.20, 39.00, 33.02, 25.42, 22.75, 13.89. HRMS (DART, [M+1]^+^) *m*/*z* calcd. For C_29_H_29_N_4_O_3_: 481.2240; found: 481.2229.

*1-[4-(2,6-Diisopropy-phenoxymethyl)-[1,2,3]triazol-1-yl]-1,2-diphenyl-hexan-2-ol (* **19** *)*

1-Azido-1,2-diphenyl-hexan-2-ol and 1,3-diisopropyl-2-prop-2-ynyloxybenzene afforded 1-[4-(2,6-diisopropylphenoxymethyl)-[1,2,3]triazol-1-yl]-1,2-diphenyl-hexan-2-ol **14** as a white solid. Yield: 376 mg (73%), m.p.: 151–151.5 °C. IR (ATR, cm^−1^): 3380, 3167, 3027, 2962, 2868, 1741, 1449, 1172, 977, 757, 698. ^1^H NMR: (300 MHz, CDCl_3_) δ 7.94 (s, 1H), 7.34–6.99 (m, 13H), 5.79 (s, 1H), 4.98 (s, 2H), 3.82 (s, 1H), 3.38 (p, *J* = 6.9 Hz, 2H), 2.07 (ddd, *J* = 13.8, 11.6, 4.5 Hz, 1H), 1.55 (ddd, *J* = 13.8, 11.7, 4.1 Hz, 1H), 1.32–1.09 (m, 15H), 1.04–0.82 (m, 1H), 0.75 (t, *J* = 7.2 Hz, 3H). ^13^C NMR: (75 MHz, CDCl_3_) δ = 152.85, 144.38, 141.89, 141.49, 135.09, 128.95, 128.10, 128.02, 127.83, 127.03, 125.84, 125.22, 125.07, 124.30, 124.18, 124.11, 79.76, 74.20, 68.15, 39.17, 26.64, 25.50, 24.10, 22.84, 13.96. HRMS (DART, [M+1]^+^) *m*/*z* calcd. For C_33_H_42_N_3_O_2_: 512.3277; found: 512.3269.

*1-(4-Morpholin-4-ylmethyl-[1,2,3]triazol-1-yl)-1,2-diphenyl-hexan-2-ol (* **20** *)*

1-Azido-1,2-diphenyl-hexan-2-ol and 4-prop-2-ynyl-morpholine afforded 1-(4-morpholin-4-ylmethyl-[1,2,3]triazol-1-yl)-1,2-diphenyl-hexan-2-oll **20** as a white solid. Yield: 260 mg (62%), m.p.: 123–123.6 °C. IR (ATR, cm^−1^): 3243, 3030, 2958, 2932, 2864, 2818, 1451, 1115, 1003, 699. ^1^H NMR: (300 MHz, CDCl_3_) δ 7.74 (s, 1H), 7.23–6.93 (m, 10H), 5.70 (s, 1H), 3.62 (q, *J* = 7.1 Hz, 7H), 2.43 (t, *J* = 4.5 Hz, 4H), 1.96 (ddd, *J* = 13.6, 11.5, 4.5 Hz, 1H), 1.45–1.25 (m, 1H), 1.06 (hept, *J* = 6.9 Hz, 4H), 0.64 (t, *J* = 7.1 Hz, 4H). ^13^C NMR: (75 MHz, CDCl_3_) δ = 141.52, 135.05, 128.93, 128.06, 127.97, 127.79, 127.00, 125.81, 79.68, 74.08, 66.83, 53.72, 53.43, 39.02, 25.38, 22.81, 13.87. HRMS (DART, [M+1]^+^) *m*/*z* calcd. For C_25_H_33_N_4_O_2_: 421.2604; found: 421.2595.

*1,2,3-Triphenyl-1-(4-phenyl-[1,2,3]triazol-1-yl)-propan-2-ol (* **21** *)*

1-Azido-1,2,3-triphenyl-propan-2-ol and phenylacetylene afforded 1,2,3-triphenyl-1-(4-phenyl-[1,2,3]triazol-1-yl)-propan-2-ol 21 as a white solid. Yield: 186 mg (43%), m.p.: 228–228.5 °C. IR (ATR, cm^−1^): 3457, 3444, 3061, 3029, 2929, 1948, 1880, 1742, 1449, 1361, 1156, 759. ^1^H NMR: (300 MHz, DMSO-d_6_) δ = 8.82 (s, 1H), 8.00–7.91 (m, 2H), 7.56–7.42 (m, 4H), 7.42–7.30 (m, 3H), 7.19–6.96 (m, 9H), 6.89 (dd, *J* = 6.7, 3.0 Hz, 2H), 6.48 (s, 1H), 5.78 (s, 1H), 3.38 (d, *J* = 13.9 Hz, 1H), 2.77 (d, *J* = 13.8 Hz, 1H). ^13^C NMR: (75 MHz, DMSO-d_6_) δ = 146.72, 142.58, 136.97, 136.61, 131.26, 131.02, 130.15, 129.33, 128.35, 128.25, 128.10, 127.73, 127.69, 126.92, 126.85, 126.32, 125.81, 122.45, 79.62, 79.53, 72.24, 46.25, 40.84, 40.56, 40.28, 40.00, 39.72, 39.45, 39.17. HRMS (DART, [M+1]^+^) *m*/*z* calcd. For C_29_H_26_N_3_O: 432.2076; found: 432.2071.

*1-(4-Cyclopropyl-[1,2,3]triazol-1-yl)-1,2,3-triphenyl-propan-2-ol (* **22** *)*

1-Azido-1,2,3-triphenyl-propan-2-ol and cyclopropylacetylene afforded 1-(4-cyclopropyl-[1,2,3]triazol-1-yl)-1,2,3-triphenyl-propan-2-ol **22** as a white solid. Yield: 233 mg (59%), m.p.: 168.2–169.0 °C. IR (ATR, cm^−1^): 3433, 3146, 3060, 3028, 3000, 2933, 1743, 1450, 1362, 1161, 1046, 727, 696. ^1^H NMR: (300 MHz, CDCl_3_) δ = 7.74 (s, 1H), 7.20–7.11 (m, 4H), 7.02 (dq, *J* = 24.4, 6.3 Hz, 9H), 6.67–6.59 (m, 2H), 6.01 (s, 1H), 3.47 (s, 1H), 3.31 (d, *J* = 13.2 Hz, 1H), 2.69 (d, *J* = 13.4 Hz, 1H), 0.93–0.80 (m, 4H). ^13^C NMR: (75 MHz, CDCl_3_) δ = 148.95, 140.34, 134.20, 133.60, 129.60, 129.43, 128.32, 128.06, 126.84, 126.75, 126.73, 126.11, 125.89, 125.65, 125.35, 124.92, 120.12, 78.29, 71.29, 44.62, 6.76, 6.68, 5.67. HRMS (DART, [M+1]^+^) *m*/*z* calcd. For C_26_H_26_N_3_O: 396.2076; found: 396.2065.

*2-[1-(2-Hydroxy-1,2,3-triphenyl-propyl)-[1,2,3]triazol-4-ylmethyl]-isoindole-1,3-dione (* **23** *)*

1-Azido-1,2,3-triphenyl-propan-2-ol and 2-prop-2-ynyl-isoindole-1,3-dione afforded 2-[1-(2-hydroxy-1,2,3-triphenyl-propyl)-[1,2,3]triazol-4-ylmethyl]-isoindole-1,3-dione **23** as a white solid. Yield: 244 mg (44%), m.p.: 224.9–225.5 °C. IR (ATR, cm^−1^): 3435, 3149, 3010, 2947, 2862, 1715, 1612, 1515, 1370, 1255, 1096, 765. ^1^H NMR: (300 MHz, CDCl_3_) δ = 8.19 (s, 1H), 7.88 (dd, *J* = 5.4, 3.1 Hz, 2H), 7.72 (dd, *J* = 5.5, 3.0 Hz, 2H), 7.31–7.01 (m, 13H), 6.68 (dt, *J* = 6.6, 1.6 Hz, 2H), 6.13 (s, 1H), 5.07 (s, 1H), 4.07–3.91 (m, 1H), 3.82–3.59 (m, 1H), 3.38 (d, *J* = 13.6 Hz, 1H), 2.67 (d, *J* = 13.6 Hz, 1H). ^13^C NMR: (75 MHz, CDCl_3_) δ = 177.69, 167.69, 142.61, 141.46, 135.01, 134.49, 134.09, 132.12, 130.60, 129.29, 128.06, 128.03, 127.96, 127.94, 127.12, 126.88, 126.07, 124.42, 123.48, 79.34, 72.68, 45.64, 33.16, 29.69, 29.55, 29.52, 27.79, 23.92, 23.84, 23.81, 23.48, 22.18, 6.15. HRMS (DART, [M+1]^+^) *m*/*z* calcd. For C_32_H_27_N_4_O_3_: 515.2083; found: 515.2069.

*1-[4-(2,6-Diisopropyl-phenoxymethyl)-[1,2,3]triazol-1-yl]-1,2,3-triphenyl-propan-2-ol (* **24** *)*

1-Azido-1,2,3-triphenyl-propan-2-ol and 1,3-diisopropyl-2-prop-2-ynyloxybenzene afforded 1-[4-(2,6-diisopropyl-phenoxymethyl)-[1,2,3]triazol-1-yl]-1,2,3-triphenyl-propan-2-ol **24** as a white solid. Yield: 225 mg (41%), m.p.: 167.1–167.6 °C. IR (ATR, cm^−1^): 3250, 3173, 3061, 3030, 2962, 2926, 2867, 1494, 1447, 1357, 1253, 1180, 1102, 1052, 968, 699. ^1^H NMR: (300 MHz, CDCl_3_) δ = 8.22 (s, 1H), 7.36–7.00 (m, 16H), 6.75–6.67 (m, 2H), 6.21 (s, 1H), 5.27 (s, 1H), 5.02 (s, 2H), 3.53–3.34 (m, 3H), 3.28–3.22 (m, 1H), 1.31–1.20 (m, 13H). ^13^C NMR: (75 MHz, CDCl_3_) δ = 153.02, 144.88, 141.91, 141.49, 135.19, 134.49, 130.62, 129.33, 128.16, 128.11, 128.04, 127.21, 127.02, 126.10, 124.18, 123.85, 79.41, 72.70, 68.31, 45.81, 26.70, 24.12, 24.08. HRMS (DART, [M+1]^+^) *m*/*z* calcd. For C_36_H_40_N_3_O_2_: 546.3121; found: 546.3112.

*1-(4-Morpholin-4-ylmethyl-[1,2,3]triazol-1-yl)-1,2,3-triphenyl-propan-2-ol (* **25** *)*

1-Azido-1,2,3-triphenyl-propan-2-ol and 4-prop-2-ynyl-morpholine afforded 1-(4-morpholin-4-ylmethyl-[1,2,3]triazol-1-yl)-1,2,3-triphenyl-propan-2-ol **25** as a white solid. Yield: 218 mg (48%), m.p.: 160.2–161.8 °C. IR (ATR, cm^−1^): 3420, 3338, 3121, 3036, 3029, 2957, 2826, 1741, 1659, 1495, 1450, 1217, 1113, 698. ^1^H NMR: (300 MHz, CDCl_3_) δ = 8.06 (s, 1H), 7.35–7.00 (m, 13H), 6.74–6.64 (m, 2H), 6.17 (s, 1H), 3.77–3.67 (m, 6H), 3.43 (d, *J* = 13.5 Hz, 1H), 3.28 (s, 1H), 2.68 (d, *J* = 13.6 Hz, 1H), 2.60–2.51 (m, 4H). ^13^C NMR: (75 MHz, CDCl_3_) δ = 141.09, 134.80, 134.13, 130.20, 128.93, 127.76, 127.69, 127.64, 127.62, 126.80, 126.62, 125.69, 123.86, 79.02, 72.19, 66.43, 53.40, 53.02, 45.34, 31.22, 22.29, 13.76. HRMS (DART, [M+1]^+^) *m*/*z* calcd. For C_28_H_31_N_4_O_2_: 455.2447; found: 455.2439.

*1-Cyclohexyl-1,2-diphenyl-2-(4-phenyl-[1,2,3]triazol-1-yl)-ethanol (* **26** *)*

2-Azido-1-cyclohexyl-1,2-diphenyl-ethanol and phenylacetylene afforded 1-cyclohexyl-1,2-diphenyl-2-(4-phenyl-[1,2,3]triazol-1-yl)-ethanol **26** as a white solid. Yield: 260 mg (61%), m.p.: 237–237.5 °C. IR (ATR, cm^−1^): 3514, 3404, 3149, 3028, 2933, 2852, 1741, 1718, 1445, 1355, 1031, 759, 692. ^1^H NMR: (300 MHz, DMSO-d_6_) δ = 8.42 (s, 1H), 7.74–7.63 (m, 2H), 7.52–7.42 (m, 2H), 7.34–7.27 (m, 2H), 7.27–7.16 (m, 2H), 7.16–7.04 (m, 1H), 6.98 (t, *J* = 7.5 Hz, 2H), 6.94–6.78 (m, 4H), 6.59 (s, 1H), 5.39 (s, 1H), 2.19–0.45 (m, 11H). ^13^C NMR: (75 MHz, DMSO-d_6_) δ = 195.29, 146.42, 141.50, 137.93, 131.30, 130.72, 130.06, 129.97, 129.28, 128.27, 127.92, 127.44, 126.73, 125.72, 122.02, 80.98, 67.68, 46.52, 28.39, 26.94, 26.60, 26.34. HRMS (DART, [M+1]^+^) *m*/*z* calcd. For C_28_H_30_N_3_O: 424.2389; found: 424.2379.

*1-Cyclohexyl-2-(4-cyclopropyl-[1,2,3]triazol-1-yl)-1,2-diphenyl-ethanol (* **27** *)*

2-Azido-1-cyclohexyl-1,2-diphenyl-ethanol and cyclopropylacetylene afforded 1-cyclohexyl-2-(4-cyclopropyl-[1,2,3]triazol-1-yl)-1,2-diphenyl-ethanol **27** as a white solid. Yield: 166 mg (43%), m.p.: 190.2–192 °C. IR (ATR, cm^−1^): 3396, 3087, 3031, 2932, 2854, 1740, 1557, 1495, 1449, 1330, 1233, 1040, 701. ^1^H NMR: (300 MHz, CDCl_3_) δ = 7.54 (s, 1H), 7.44–7.33 (m, 2H), 7.33–7.17 (m, 4H), 7.21–7.10 (m, 1H), 7.15–6.99 (m, 3H), 6.34 (s, 1H), 3.79 (s, 1H), 1.95 (ddd, *J* = 13.4, 6.7, 4.2 Hz, 2H), 1.89–1.67 (m, 2H), 1.65 (s, 1H), 1.55 (dd, *J* = 27.8, 12.3 Hz, 2H), 1.39 (tt, *J* = 11.7, 2.7 Hz, 1H), 1.29–0.76 (m, 7H), 0.47–0.28 (m, 1H). ^13^C NMR: (75 MHz, CDCl_3_) δ = 148.89, 139.13, 135.15, 128.69, 127.16, 127.06, 126.65, 126.10, 126.02, 125.84, 120.64, 80.43, 67.82, 46.56, 28.03, 26.20, 25.82, 25.51, 25.35, 7.13, 7.09, 6.00, -0.75. HRMS (DART, [M+1]^+^) *m*/*z* calcd. For C_25_H_30_N_3_O: 388.2389; found: 388.2379.

*2-[1-(2-Cyclohexyl-2-hydroxy-1,2-diphenyl-ethyl)-[1,2,3]triazol-4-ylmethyl]-isoindole-1,3-dione (* **28** *)*

2-Azido-1-cyclohexyl-1,2-diphenyl-ethanol and 2-prop-2-ynyl-isoindole-1,3-dione afforded 2-[1-(2-cyclohexyl-2-hydroxy-1,2-diphenyl-ethyl)-[1,2,3]triazol-4-ylmethyl]-isoindole-1,3-dione **28** as a white solid. Yield: 324 mg (64%), m.p.: 218–218.3 °C. IR (ATR, cm^−1^): 3555, 3464, 3292, 2930, 2854, 1770, 1708, 1608, 1393, 1329, 1097, 939, 698. ^1^H NMR: (300 MHz, CDCl_3_) δ = 7.96 (s, 1H), 7.84 (dd, *J* = 5.5, 3.0 Hz, 2H), 7.70 (dd, *J* = 5.5, 3.1 Hz, 2H), 7.46–7.32 (m, 2H), 7.32–7.18 (m, 4H), 7.18–7.10 (m, 1H), 7.06 (p, *J* = 3.6 Hz, 3H), 6.40 (s, 1H), 5.08–4.95 (m, 2H), 2.01–1.89 (m, 1H), 1.81 (s, 1H), 1.65–1.52 (m, 3H), 1.45 (d, *J* = 11.7 Hz, 1H), 1.30 (tt, *J* = 11.8, 2.7 Hz, 1H), 1.19–0.76 (m, 4H), 0.36 (qd, *J* = 12.8, 3.4 Hz, 1H). ^13^C NMR: (75 MHz, CDCl_3_) δ = 191.68, 167.64, 142.10, 139.76, 135.48, 134.08, 132.08, 129.54, 129.06, 127.99, 127.43, 126.91, 126.58, 124.59, 123.46, 81.17, 68.94, 47.17, 33.09, 28.70, 26.93, 26.45, 26.19, 26.07. HRMS (DART, [M+1]^+^) *m*/*z* calcd. For C_31_H_31_N_4_O_3_: 507.2396; found: 507.2390.

*1-Cyclohexyl-2-[4-(2,6-diisopropyl-phenoxymethyl)-[1,2,3]triazol-1-yl]-1,2-diphenyl-ethanol (* **29** *)*

2-Azido-1-cyclohexyl-1,2-diphenyl-ethanol and 1,3-diisopropyl-2-prop-2-ynyloxybenzene afforded 1-cyclohexyl-2-[4-(2,6-diisopropyl-phenoxymethyl)-[1,2,3]triazol-1-yl]-1,2-diphenyl-ethanol **29** as a white solid. Yield: 163 mg (30%), m.p.: 144–144.3 °C. IR (ATR, cm^−1^): 3260, 3064, 3029, 2959, 2928, 2864, 1741, 1600, 1448, 1317, 1177, 996, 703. ^1^H NMR: (300 MHz, CDCl_3_) δ = 8.00 (s, 1H), 7.44–7.27 (m, 4H), 7.31–7.14 (m, 3H), 7.19–7.05 (m, 3H), 6.50 (s, 1H), 4.99 (d, *J* = 2.6 Hz, 2H), 3.53 (s, 1H), 3.37 (hept, *J* = 6.9 Hz, 2H), 2.05 (d, *J* = 11.5 Hz, 1H), 1.91–1.80 (m, 1H), 1.76–1.52 (m, 2H), 1.50 (s, 1H), 1.47–1.16 (m, 15H), 1.19–0.92 (m, 2H), 0.97–0.80 (m, 3H), 0.42 (qd, *J* = 12.7, 3.4 Hz, 1H). ^13^C NMR: (75 MHz, CDCl_3_) δ = 153.01, 144.39, 141.89, 139.79, 135.70, 129.56, 128.09, 128.01, 127.50, 126.98, 126.62, 125.02, 124.03, 81.22, 68.83, 68.24, 47.20, 28.74, 26.95, 26.68, 26.53, 26.26, 26.09, 25.30, 24.07, 24.04, 22.67. HRMS (DART, [M+1]^+^) *m*/*z* calcd. For C_35_H_44_N_3_O_2_: 538.3434; found: 538.3426.

*1-Cyclohexyl-2-(4-morpholin-4-ylmethyl-[1,2,3]triazol-1-yl)-1,2-diphenyl-ethanol (* **30** *)*

2-Azido-1-cyclohexyl-1,2-diphenyl-ethanol and 4-prop-2-ynyl-morpholine afforded 1-cyclohexyl-2-(4-morpholin-4-ylmethyl-[1,2,3]triazol-1-yl)-1,2-diphenyl-ethanol **30** as a white solid. Yield: 215 mg (48%), m.p.: 169.5–170.3 °C. IR (ATR, cm^−1^): 3480, 3109, 3033, 2930, 2853, 2813, 1673, 1496, 1449, 1116, 1052, 1002, 861, 702. ^1^H NMR: (300 MHz, CDCl_3_) δ = 7.92 (s, 1H), 7.39–7.05 (m, 10H), 6.45 (s, 1H), 3.72 (d, *J* = 5.5 Hz, 7H), 2.56 (d, *J* = 6.0 Hz, 4H), 1.97 (d, *J* = 11.7 Hz, 1H), 1.85 (s, 1H), 1.72–1.55 (m, 2H), 1.54–1.40 (m, 2H), 1.39–0.80 (m, 4H), 0.39 (qd, *J* = 12.6, 3.3 Hz, 1H). ^13^C NMR: (75 MHz, CDCl_3_) δ = 200.81, 139.87, 135.63, 129.48, 128.03, 127.96, 127.50, 126.96, 126.58, 124.65, 81.19, 68.86, 66.64, 60.09, 53.31, 47.28, 28.65, 26.94, 26.50, 26.36, 14.13, 12.03. HRMS (DART, [M+1]^+^) *m*/*z* calcd. For C_27_H_35_N_4_O_2_: 447.2760; found: 447.2850.

*1,2-Bis-(4-methoxyphenyl)-1-(4-phenyl-[1,2,3]triazol-1-yl)-butan-2-ol (* **31** *)*

1-Azido-1,2-bis-(4-methoxyphenyl)-butan-2-ol and phenylacetylene afforded 1,2-bis-(4-methoxy-phenyl)-1-(4-phenyl-[1,2,3]triazol-1-yl)-butan-2-ol **31** as a white solid. Yield: 262 mg (61%), m.p.: 171–171.6 °C. IR (ATR, cm^−1^): 3451, 3159, 2966, 2935, 2833, 1741, 1611, 1512, 1253, 1176, 1031, 797. ^1^H NMR: (300 MHz, DMSO-d_6_) δ = 8.68 (s, 1H), 7.96–7.88 (m, 2H), 7.53–7.40 (m, 4H), 7.40–7.27 (m, 3H), 6.82–6.74 (m, 2H), 6.74–6.65 (m, 2H), 6.17 (s, 1H), 5.55 (s, 1H), 3.65 (d, *J* = 14.6 Hz, 6H), 1.95 (dq, *J* = 14.5, 7.2 Hz, 1H), 1.33 (dt, *J* = 14.1, 7.1 Hz, 1H), 0.55 (t, *J* = 7.2 Hz, 3H). ^13^C NMR: (75 MHz, DMSO-d_6_) δ = 158.87, 157.92, 146.52, 131.43, 131.33, 129.55, 129.30, 128.25, 127.76, 125.70, 121.82, 113.56, 113.37, 79.06, 72.01, 55.30, 55.26, 33.05, 8.13. HRMS (DART, [M+1]^+^) *m*/*z* calcd. For C_26_H_28_N_3_O_3_: 430.2131; found: 430.2123.

*1-(4-Cyclopropyl-[1,2,3]triazol-1-yl)-1,2-bis-(4-methoxyphenyl)-butan-2-ol (* **32** *)*

1-Azido-1,2-bis-(4-methoxyphenyl)-butan-2-ol and cyclopropylacetylene afforded 1-(4-cyclopropyl-[1,2,3]triazol-1-yl)-1,2-bis-(4-methoxyphenyl)-butan-2-ol **32** as a white solid. Yield: 259 mg (66%), m.p.: 136.5–138 °C. IR (ATR, cm^−1^): 3211, 3012, 2957, 2837, 1741, 1610, 1511, 1462, 1245, 1177, 1029, 797. ^1^H NMR: (300 MHz, CDCl_3_) δ 7.40 (s, 1H), 7.13–7.02 (m, 2H), 7.02–6.91 (m, 2H), 6.73–6.63 (m, 2H), 6.59–6.48 (m, 2H), 5.52 (s, 1H), 3.81 (s, 1H), 3.68 (s, 3H), 3.61 (s, 3H), 1.97–1.84 (m, 1H), 1.84 (dd, *J* = 8.1, 5.0 Hz, 1H), 1.40 (dq, *J* = 14.3, 7.2 Hz, 1H), 0.93–0.72 (m, 4H), 0.59 (t, *J* = 7.3 Hz, 3H). ^13^C NMR: (75 MHz, CDCl_3_) δ = 159.03, 158.33, 149.58, 133.31, 130.14, 127.70, 127.19, 121.59, 113.33, 113.12, 79.77, 77.49, 77.27, 77.07, 76.64, 73.33, 55.14, 55.09, 32.08, 7.87, 7.85, 7.58, 6.67. HRMS (DART, [M+1]^+^) *m*/*z* calcd. For C_23_H_28_N_3_O_3_: 394.2131; found: 394.2121.

*2-{1-[2-Hydroxy-1,2-bis-(4-methoxy-phenyl)-butyl]-[1,2,3]triazol-4-ylmethyl}-isoindole-1,3-dione (* **33** *)*

1-Azido-1,2-bis-(4-methoxyphenyl)-butan-2-ol and 2-prop-2-ynyl-isoindole-1,3-dione afforded 2-{1-[2-hydroxy-1,2-bis-(4-methoxy-phenyl)-butyl]-[1,2,3]triazol-4-ylmethyl}-isoindole-1,3-dione **33** as a white solid. Yield: 230 mg (45%), m.p.: 148.2–149 °C. IR (ATR, cm^−1^): 3522, 3465, 3396, 3138, 2970, 2936, 1714, 1613, 1514, 1256, 1032, 716. ^1^H NMR: (300 MHz, CDCl_3_) δ 7.83–7.72 (m, 3H), 7.69–7.57 (m, 2H), 7.10–7.02 (m, 2H), 7.01–6.94 (m, 2H), 6.67 (d, *J* = 8.6 Hz, 2H), 6.52 (d, *J* = 8.5 Hz, 2H), 5.56 (s, 1H), 4.92 (s, 2H), 3.90–3.29 (m, 7H), 1.87 (dq, *J* = 14.6, 7.3 Hz, 1H), 1.35 (dq, *J* = 14.3, 7.2 Hz, 1H), 0.56 (t, *J* = 7.2 Hz, 3H). ^13^C NMR: (75 MHz, CDCl_3_) δ = 167.67, 159.09, 158.35, 134.11, 133.13, 132.05, 130.23, 127.35, 127.15, 124.56, 123.48, 113.35, 113.17, 79.71, 73.65, 55.13, 55.08, 33.01, 32.03, 7.53. HRMS (DART, [M+1]^+^) *m*/*z* calcd. For C_29_H_28_N_4_O_5_: 513.2138; found: 513.2132.

*1-[4-(2,6-Diisopropylphenoxymethyl)-[1,2,3]triazol-1-yl]-1,2-bis-(4-methoxyphenyl)-butan-2-ol (* **34** *)*

1-Azido-1,2-bis-(4-methoxyphenyl)-butan-2-ol and 1,3-diisopropyl-2-prop-2-ynyloxybenzene afforded 1-[4-(2,6-diisopropyl-phenoxymethyl)-[1,2,3]triazol-1-yl]-1,2-bis-(4-methoxy-phenyl)-butan-2-ol **34** as a white solid. Yield: 298 mg (55%), m.p.: 128.5–129 °C. IR (ATR, cm^−1^): 3474, 3149, 3013, 2962, 2932, 2838, 1612, 1513, 1462, 1250, 1178, 1036, 803. ^1^H NMR: (300 MHz, CDCl_3_) δ 7.83 (s, 1H), 7.15–7.06 (m, 2H), 7.06–6.97 (m, 5H), 6.75–6.65 (m, 2H), 6.62–6.51 (m, 2H), 5.66 (s, 1H), 4.89 (s, 2H), 3.68 (s, 3H), 3.62 (s, 3H), 3.30 (hept, *J* = 6.8 Hz, 2H), 1.94 (dq, *J* = 14.6, 7.4 Hz, 1H), 1.45 (dq, *J* = 14.3, 7.2 Hz, 1H), 1.14 (d, *J* = 6.9 Hz, 12H), 0.61 (t, *J* = 7.2 Hz, 3H). ^13^C NMR: (75 MHz, CDCl_3_) δ = 159.17, 158.43, 152.83, 144.23, 141.89, 133.18, 130.25, 127.18, 125.06, 124.17, 124.10, 113.43, 113.24, 79.81, 73.66, 68.08, 55.16, 55.13, 32.17, 26.63, 24.09, 7.59. HRMS (DART, [M+1]^+^) *m*/*z* calcd. For C_33_H_42_N_3_O_4_: 544.3175; found: 544.3168.

*1-(4-Morpholin-4-ylmethyl-[1,2,3]triazol-1-yl)- 1,2-bis-(4-methoxyphenyl)-butan-2-ol (* **35** *)*

1-Azido-1,2-bis-(4-methoxyphenyl)-butan-2-ol and 4-prop-2-ynyl-morpholine afforded 1-(4-morpholin-4-ylmethyl-[1,2,3]triazol-1-yl)-1,2-bis-(4-methoxyphenyl)-butan-2-ol **35** as a white solid. Yield: 271 mg (60%), m.p.: 198.5–199 °C. IR (ATR, cm^−1^): 3211, 3012, 2957, 2837, 1741, 1610, 1511, 1462, 1245, 1177, 1029, 797. ^1^H NMR: (300 MHz, CDCl_3_) δ 7.75 (s, 1H), 7.21–7.05 (m, 4H), 6.82–6.73 (m, 2H), 6.69–6.60 (m, 2H), 5.69 (s, 1H), 3.80–3.68 (m, 13H), 2.76 (s, 1H), 2.53 (t, *J* = 4.6 Hz, 4H), 2.00 (td, *J* = 13.3, 6.0 Hz, 1H), 1.46 (dq, *J* = 14.3, 7.2 Hz, 1H), 0.68 (t, *J* = 7.3 Hz, 3H). ^13^C NMR: (75 MHz, CDCl_3_) δ = 159.11, 158.38, 143.22, 133.22, 130.21, 127.17, 124.44, 113.37, 113.20, 79.74, 73.49, 66.74, 55.14, 55.10, 53.65, 53.34, 32.00, 7.57. HRMS (DART, [M+1]^+^) *m*/*z* calcd. For C_25_H_35_N_4_O_4_: 453.2502; found: 453.2492.

*1,2-Bis-(4-methoxyphenyl)-1-(4-phenyl-[1,2,3]triazol-1-yl)-hexan-2-ol (* **36** *)*

1-Azido-1,2-bis-(4-methoxyphenyl)-hexan-2-ol and phenylacetylene afforded 1,2-bis-(4-methoxy-phenyl)-1-(4-phenyl-[1,2,3]triazol-1-yl)-hexan-2-ol **36** as a white solid. Yield: 228 mg (50%), m.p.: 163.8–163.5 °C. IR (ATR, cm^−1^): 3196, 2950, 2930, 2834, 1610, 1512, 1457, 1250, 1174, 1033, 760. ^1^H NMR: (300 MHz, CDCl_3_) δ 7.97 (s, 1H), 7.77 (d, *J* = 7.5 Hz, 2H), 7.34 (t, *J* = 7.5 Hz, 2H), 7.29–7.20 (m, 1H), 7.11 (d, *J* = 8.3 Hz, 2H), 7.02 (d, *J* = 8.3 Hz, 2H), 6.70 (d, *J* = 8.3 Hz, 2H), 6.55 (d, *J* = 8.3 Hz, 2H), 5.67 (s, 1H), 3.68 (s, 3H), 3.60 (s, 3H), 1.92 (t, *J* = 12.8 Hz, 1H), 1.43 (td, *J* = 12.7, 3.9 Hz, 1H), 1.20–0.99 (m, 4H), 0.82 (d, *J* = 13.7 Hz, 1H), 0.65 (t, *J* = 7.1 Hz, 3H). ^13^C NMR: (75 MHz, CDCl_3_) δ = 159.13, 158.38, 147.22, 133.76, 132.51, 130.37, 130.21, 128.89, 128.30, 127.53, 127.07, 125.75, 121.32, 114.22, 113.68, 113.42, 113.26, 79.57, 73.64, 55.16, 55.11, 39.27, 25.52, 22.81, 13.97. HRMS (DART, [M+1]^+^) *m*/*z* calcd. For C_28_H_32_N_3_O_3_: 458.2444; found: 458.2436.

*1-(4-Cyclopropyl-[1,2,3]triazol-1-yl)-1,2-bis-(4-methoxyphenyl)-hexan-2-ol (* **37** *)*

1-Azido-1,2-bis-(4-methoxyphenyl)-hexan-2-ol and cyclopropylacetylene afforded 1-(4-cyclopropyl-[1,2,3]triazol-1-yl)-1,2-bis-(4-methoxy-phenyl)-hexan-2-ol **37** as a white solid. Yield: 160 mg (38%), m.p.: 176–176.7 °C. IR (ATR, cm^−1^): 3211, 3170, 2955, 2931, 2838, 1741, 1610, 1511, 1447, 1249, 1177, 1029, 801. ^1^H NMR: (300 MHz, CDCl_3_) δ 7.38 (s, 1H), 7.07 (dd, *J* = 9.0, 2.4 Hz, 2H), 6.98–6.89 (m, 2H), 6.73–6.63 (m, 2H), 6.58–6.49 (m, 2H), 5.50 (s, 1H), 3.89–3.82 (m, 1H), 3.68 (d, *J* = 1.0 Hz, 3H), 3.61 (d, *J* = 0.9 Hz, 3H), 1.94–1.79 (m, 1H), 1.36 (td, *J* = 12.6, 3.9 Hz, 1H), 1.20–0.97 (m, 3H), 0.94–0.73 (m, 5H), 0.66 (t, *J* = 7.1 Hz, 3H). ^13^C NMR: (75 MHz, CDCl_3_) δ = 159.02, 158.29, 149.59, 133.81, 130.11, 129.90, 127.70, 127.05, 121.56, 114.05, 113.32, 113.10, 79.50, 77.48, 77.06, 76.63, 73.44, 55.25, 55.08, 39.13, 25.51, 22.83, 13.96, 7.86, 6.69. HRMS (DART, [M+1]^+^) *m*/*z* calcd. For C_25_H_32_N_3_O_3_: 422.2444; found: 422.2435.

*2-{1-[2-Hydroxy-1,2-bis-(4-methoxyphenyl)-hexyl]-[1,2,3]triazol-4-ylmethyl}-isoindole-1,3-dione (* **38** *)*

1-Azido-1,2-bis-(4-methoxyphenyl)-hexan-2-ol and 2-prop-2-ynyl-isoindole-1,3-dione afforded 1-(4-cyclopropyl-[1,2,3]triazol-1-yl)-1,2-bis-(4-methoxy-phenyl)-hexan-2-ol **38** as a white solid. Yield: 226 mg (42%), m.p.: 100–101.5 °C. IR (ATR, cm^−1^): 3435, 3149, 3010, 2947, 2862, 1715, 1612, 1515, 1370, 1255, 1096, 765. ^1^H NMR: (300 MHz, CDCl_3_) δ 7.87–7.74 (m, 1H), 7.75 (q, *J* = 3.2 Hz, 2H), 7.70–7.56 (m, 2H), 7.10–7.00 (m, 2H), 6.94 (d, *J* = 8.5 Hz, 2H), 6.72–6.62 (m, 2H), 6.51 (d, *J* = 8.5 Hz, 2H), 5.54 (s, 1H), 4.93 (s, 2H), 3.67 (s, 3H), 3.59 (s, 3H), 1.93–1.72 (m, 1H), 1.29 (td, *J* = 12.6, 3.8 Hz, 1H), 1.21–0.93 (m, 4H), 0.81 (s, 1H), 0.62 (t, *J* = 7.1 Hz, 3H). ^13^C NMR: (75 MHz, CDCl_3_) δ = 190.17, 167.65, 159.07, 158.32, 134.09, 133.63, 132.06, 130.20, 127.30, 127.02, 125.48, 123.47, 113.34, 113.15, 79.45, 73.76, 55.08, 39.04, 33.04, 25.46, 22.76, 13.91. HRMS (DART, [M+1]^+^) *m*/*z* calcd. For C_31_H_33_N_4_O_5_: 541.2451; found: 541.2443.

*1-[4-(2,6-Diisopropy-phenoxymethyl)-[1,2,3]triazol-1-yl]-1,2-bis-(4-methoxyphenyl)-hexan-2-ol (* **39** *)*

1-Azido-1,2-bis-(4-methoxyphenyl)-hexan-2-ol and 1,3-diisopropyl-2-prop-2-ynyloxybenzene afforded 1-[4-(2,6-diisopropylphenoxymethyl)-[1,2,3]triazol-1-yl]-1,2-bis-(4-methoxy-phenyl)-hexan-2-ol **39** as a white solid. Yield: 226 mg (46%), m.p.: 120.8–121.5 °C. IR (ATR, cm^−1^): 3466, 3145, 3013, 2962, 2939, 1741, 1612, 1513, 1459, 1250, 1180, 1036, 806. ^1^H NMR: (300 MHz, CDCl_3_) δ 8.19–7.67 (m, 1H), 7.23–7.05 (m, 7H), 6.80 (d, *J* = 8.0 Hz, 2H), 6.67 (d, *J* = 7.6 Hz, 2H), 5.96–5.63 (m, 1H), 5.18–4.69 (m, 2H), 3.85–3.65 (m, 7H), 3.42 (s, 2H), 2.05 (d, *J* = 12.2 Hz, 1H), 1.26 (d, *J* = 4.3 Hz, 16H), 0.95 (s, 1H), 0.79 (t, *J* = 6.8 Hz, 3H). ^13^C NMR: (75 MHz, CDCl_3_) δ = 159.14, 158.35, 152.81, 141.89, 133.71, 130.64, 127.49, 126.57, 125.49, 124.59, 123.69, 114.00, 113.53, 112.79, 79.48, 55.10, 26.31, 24.21, 24.00, 22.85. HRMS (DART, [M+1]^+^) *m*/*z* calcd. For C_35_H_46_N_3_O_4_: 572.3488; found: 572.3483.

*1,2-Bis-(4-methoxyphenyl)-1-(4-morpholin-4-ylmethyl-[1,2,3]triazol-1-yl)-hexan-2-ol (* **40** *)*

1-Azido-1,2-bis-(4-methoxyphenyl)-hexan-2-ol and 4-prop-2-ynyl-morpholine afforded 1,2-bis-(4-methoxy-phenyl)-1-(4-morpholin-4-ylmethyl-[1,2,3]triazol-1-yl)-hexan-2-ol **40** as a white solid. Yield: 197 mg (41%), m.p.: 110.8–111.5 °C. IR (ATR, cm^−1^): 3258, 3004, 2951, 2863, 1741, 1610, 1512, 1243, 1115, 1034, 800. ^1^H NMR: (300 MHz, CDCl_3_) δ 7.75 (s, 1H), 7.16 (dd, *J* = 9.3, 2.5 Hz, 2H), 7.07 (dd, *J* = 9.3, 2.6 Hz, 2H), 6.82–6.72 (m, 2H), 6.72–6.58 (m, 2H), 5.67 (s, 1H), 3.89–3.68 (m, 13H), 2.57–2.48 (m, 4H), 1.97 (ddd, *J* = 13.7, 11.4, 4.3 Hz, 1H), 1.46–1.30 (m, 1H), 1.17 (m, 2H), 0.99–0.83 (m, 1H), 0.74 (t, *J* = 7.1 Hz, 3H). ^13^C NMR: (75 MHz, CDCl_3_) δ = 190.43, 159.10, 158.36, 143.35, 133.70, 130.19, 127.42, 127.03, 124.40, 113.36, 113.18, 79.48, 73.60, 66.77, 55.36, 53.38, 39.02, 25.42, 22.82, 13.89. HRMS (DART, [M+1]^+^) *m*/*z* calcd. For C_27_H_37_N_4_O_4_: 481.2815; found: 481.2806.

*1,2-Bis-(4-methoxyphenyl)-3-phenyl-1-(4-phenyl-[1,2,3]triazol-1-yl)-propan-2-ol (* **41** *)*

1-Azido-1,2-bis-(4-methoxyphenyl)-3-phenyl-propan-2-ol and phenylacetylene afforded 1,2-bis-(4-methoxy-phenyl)-3-phenyl-1-(4-phenyl-[1,2,3]triazol-1-yl)-propan-2-ol **41** as a white solid. Yield: 227 mg (46%), m.p.: 183–183.7 °C. IR (ATR, cm^−1^): 3457, 3144, 3061, 3029, 2929, 1880, 1742, 1493, 1449, 1361, 1329, 1156, 759, 693. ^1^H NMR: (300 MHz, DMSO-d_6_) δ = 8.75 (s, 1H), 7.99–7.89 (m, 2H), 7.52–7.41 (m, 4H), 7.40–7.30 (m, 1H), 7.30–7.21 (m, 2H), 7.03 (dd, *J* = 5.2, 1.9 Hz, 3H), 6.89 (dt, *J* = 6.2, 2.2 Hz, 2H), 6.76–6.62 (m, 4H), 6.37 (s, 1H), 5.65 (s, 1H), 3.63 (d, *J* = 2.7 Hz, 6H), 3.31 (d, *J* = 13.8 Hz, 1H), 2.70 (d, *J* = 13.8 Hz, 1H). ^13^C NMR: (75 MHz, DMSO-d_6_) δ = 156.45, 155.43, 144.22, 134.34, 132.21, 128.96, 128.84, 128.74, 128.61, 126.88, 126.39, 125.88, 125.66, 125.29, 123.85, 123.33, 111.18, 110.54, 76.96, 69.26, 52.87, 52.76, 44.00, 38.35, 38.08, 37.80, 37.52, 37.24, 36.96, 36.69. HRMS (DART, [M+1]^+^) *m*/*z* calcd. For C_31_H_30_N_3_O_3_: 492.2287; found: 492.2282.

*1-(4-Cyclopropyl-[1,2,3]triazol-1-yl)-1,2-bis-(4-methoxyphenyl)-3-phenyl-propan-2-ol (* **42** *)*

1-Azido-1,2-bis-(4-methoxyphenyl)-3-phenyl-propan-2-ol and cyclopropylacetylene afforded 1-(4-cyclopropyl-[1,2,3]triazol-1-yl)-1,2-bis-(4-methoxy-phenyl)-3-phenyl-propan-2-ol **42** as a white solid. Yield: 227 mg (50%), m.p.: 176.9–177.5 °C. IR (ATR, cm^−1^): 3418, 3151, 3079, 3013, 2952, 2835, 1741, 1612, 1512, 1460, 1251, 1179, 1030, 806, 541. ^1^H NMR: (300 MHz, CDCl_3_) δ = 7.62 (s, 1H), 7.15–7.07 (m, 2H), 7.07–6.93 (m, 5H), 6.68–6.58 (m, 4H), 6.58–6.48 (m, 2H), 5.90 (s, 1H), 3.77–3.63 (m, 1H), 3.64 (s, 3H), 3.58 (s, 3H), 3.31 (s, 1H), 3.25 (d, *J* = 13.5 Hz, 1H), 2.63 (d, *J* = 13.5 Hz, 1H), 1.91 (tt, *J* = 8.1, 5.1 Hz, 1H), 0.95–0.76 (m, 4H). ^13^C NMR: (75 MHz, CDCl_3_) δ = 159.02, 158.35, 135.00, 133.75, 130.67, 130.48, 128.01, 127.82, 127.29, 126.76, 113.29, 113.23, 78.95, 72.02, 55.09, 55.06, 45.87, 7.94, 7.85, 6.85. HRMS (DART, [M+1]^+^) *m*/*z* calcd. For C_28_H_30_N_3_O_3_: 456.2287; found: 456.2281.

*2-{1-[2-Hydroxy-1,2-bis-(4-methoxyphenyl)-3-phenyl-propyl]-[1,2,3]triazol-4-ylmethyl}-isoindole-1,3-dione (* **43** *)*

1-Azido-1,2-bis-(4-methoxyphenyl)-3-phenyl-propan-2-ol and 2-prop-2-ynyl-isoindole-1,3-dione afforded 2-{1-[2-hydroxy-1,2-bis-(4-methoxy-phenyl)-3-phenyl-propyl]-1H-[1,2,3]triazol-4-ylmethyl}-isoindole-1,3-dione **43** as a white solid. Yield: 229 mg (40%), m.p.: 192.3–193.9 °C. IR (ATR, cm^−1^): 3466, 3144, 3061, 3024, 2951, 2836, 1706, 1612, 1514, 1398, 1254, 1182, 1097, 1028, 712. ^1^H NMR: (300 MHz, DMSO-d_6_) δ = 8.32 (s, 1H), 7.95–7.80 (m, 4H), 7.39–7.28 (m, 2H), 7.26–7.15 (m, 2H), 7.00 (dd, *J* = 4.8, 1.9 Hz, 3H), 6.80 (dd, *J* = 6.6, 3.0 Hz, 2H), 6.66 (t, *J* = 8.6 Hz, 4H), 6.29 (s, 1H), 5.51 (s, 1H), 4.89 (s, 2H), 3.61 (d, *J* = 2.3 Hz, 6H), 3.18 (d, *J* = 13.6 Hz, 1H), 2.56 (d, *J* = 13.7 Hz, 1H). ^13^C NMR: (75 MHz, DMSO-d_6_) δ = 167.89, 158.87, 157.89, 142.39, 136.69, 135.04, 134.59, 132.07, 131.24, 131.03, 129.14, 128.05, 127.70, 126.25, 124.35, 123.70, 113.57, 112.95, 79.39, 71.45, 55.31, 55.20, 46.30, 40.79, 40.51, 40.23, 39.96, 39.68, 39.40, 39.12, 33.49, 30.89. HRMS (DART, [M+1]^+^) *m*/*z* calcd. For C_34_H_31_N_4_O_5_: 575.2294; found: 575.2087.

*1-[4-(2,6-Diisopropylphenoxymethyl)-[1,2,3]triazol-1-yl]-1,2-bis-(4-methoxy-phenyl)-3-phenyl-propan-2-ol (* **44** *)*

1-Azido-1,2-bis-(4-methoxyphenyl)-3-phenyl-propan-2-ol and 1,3-diisopropyl-2-prop-2-ynyloxybenzene afforded 1-[4-(2,6-diisopropyl-phenoxymethyl)-[1,2,3]triazol-1-yl]-1,2-bis-(4-methoxy-phenyl)-3-phenyl-propan-2-ol **44** as a white solid. Yield: 229 mg (38%), m.p.: 148.2–148.9 °C. IR (ATR, cm^−1^): 3388, 3139, 3064, 2960, 2908, 2868, 2837, 1740, 1612, 1514, 1457, 1251, 1182, 1035, 806, 542. ^1^H NMR: (300 MHz, CDCl_3_) δ = 8.23 (s, 1H), 7.29 (d, *J* = 8.5 Hz, 2H), 7.22–7.07 (m, 8H), 6.80–6.72 (m, 4H), 6.68 (d, *J* = 8.2 Hz, 2H), 6.18 (s, 1H), 5.05 (s, 2H), 3.77 (s, 3H), 3.72 (s, 3H), 3.44 (t, *J* = 9.7 Hz, 3H), 3.16 (s, 1H), 2.77 (d, *J* = 13.2 Hz, 1H), 1.28 (d, *J* = 5.7 Hz, 12H). ^13^C NMR: (75 MHz, CDCl_3_) δ = 159.16, 158.46, 153.02, 141.92, 134.69, 133.71, 130.68, 130.60, 128.14, 127.29, 126.95, 125.03, 124.17, 113.41, 113.34, 55.12, 55.11, 45.86, 26.69, 24.14, 24.10. HRMS (DART, [M+1]^+^) *m*/*z* calcd. For C_38_H_44_N_3_O_4_: 606.3332; found: 606.3327.

*1,2-Bis-(4-methoxyphenyl)-1-(4-morpholin-4-ylmethyl-[1,2,3]triazol-1-yl)-3-phenyl-propan-2-ol (* **45** *)*

1-Azido-1,2-bis-(4-methoxyphenyl)-3-phenyl-propan-2-ol and 4-prop-2-ynyl-morpholine afforded 1,2-bis-(4-methoxyphenyl)-1-(4-morpholin-4-ylmethyl-[1,2,3]triazol-1-yl)-3-phenyl-propan-2-ol **45** as a white solid. Yield: 262 mg (51%), m.p.: 161.5–162.4 °C. IR (ATR, cm^−1^): 3437, 3143, 2926, 2835, 1741, 1611, 1513, 1456, 1254, 1178, 1118, 1029, 752, 701, 547. ^1^H NMR: (300 MHz, CDCl_3_) δ 7.93 (s, 1H), 7.16 (d, *J* = 8.6 Hz, 3H), 7.08–6.94 (m, 6H), 6.67–6.51 (m, 7H), 5.99 (s, 1H), 3.66 (d, *J* = 6.0 Hz, 10H), 3.60 (s, 3H), 3.29 (d, *J* = 13.5 Hz, 1H), 3.07 (s, 1H), 2.56 (d, *J* = 13.5 Hz, 1H). ^13^C NMR: (75 MHz, CDCl_3_) δ = 159.09, 158.41, 134.73, 133.67, 131.21, 130.62, 130.55, 130.19, 130.03, 129.79, 128.45, 128.33, 127.26, 126.91, 125.91, 124.09, 114.23, 114.11, 113.65, 113.38, 113.29, 112.82, 112.52, 79.21, 72.12, 66.80, 65.85, 55.10, 55.07, 53.80, 53.37, 45.74, 37.94, 15.29. HRMS (DART, [M+1]^+^) *m*/*z* calcd. For C_30_H_35_N_4_O_4_: 515.2658; found: 515.2656.

*1,2-Bis-(4-methoxyphenyl)-5-methyl-1-(4-phenyl-[1,2,3]triazol-1-yl)-hexan-2-ol (* **46** *)*

1-Azido-1,2-bis-(4-methoxyphenyl)-5-methyl-hexan-2-ol and phenylacetylene afforded 1,2-bis-(4-methoxyphenyl)-5-methyl-1-(4-phenyl-[1,2,3]triazol-1-yl)-hexan-2-ol **46** as a white solid. Yield: 296 mg (63%), m.p.: 175–175.6 °C. IR (ATR, cm^−1^): 3249, 3151, 3001, 2952, 2867, 1741, 1611, 1510, 1461, 1365, 1248, 1177, 1036, 766, 561. ^1^H NMR: (300 MHz, CDCl_3_) δ 8.13–8.04 (m, 1H), 7.94–7.80 (m, 2H), 7.44 (ddd, *J* = 7.6, 6.5, 1.3 Hz, 2H), 7.43–7.29 (m, 1H), 7.27–7.15 (m, 2H), 7.18–7.06 (m, 2H), 6.92–6.74 (m, 2H), 6.72–6.60 (m, 2H), 5.77 (s, 1H), 3.91–3.67 (m, 7H), 2.06 (td, *J* = 13.0, 4.3 Hz, 1H), 1.62–1.46 (m, 1H), 1.45–1.12 (m, 2H), 1.24 (s, 1H), 0.74 (d, *J* = 6.4 Hz, 7H). ^13^C NMR: (75 MHz, CDCl_3_) δ = 159.13, 158.37, 147.20, 133.71, 130.18, 128.88, 128.29, 127.56, 127.09, 125.75, 121.28, 113.41, 113.25, 79.59, 73.71, 55.15, 55.11, 37.27, 32.19, 28.08, 22.82, 22.18. HRMS (DART, [M+1]^+^) *m*/*z* calcd. For C_29_H_34_N_3_O_3_: 472.2600; found: 472.2589.

*1-(4-Cyclopropyl-[1,2,3]triazol-1-yl)-1,2-bis-(4-methoxyphenyl)-5-methyl-hexan-2-ol (* **47** *)*

1-Azido-1,2-bis-(4-methoxyphenyl)-5-methyl-hexan-2-ol and cyclopropylacetylene afforded 1-(4-cyclopropyl-[1,2,3]triazol-1-yl)-1,2-bis-(4-methoxy-phenyl)-5-methyl-hexan-2-ol **47** as a white solid. Yield: 200 mg (46%), m.p.: 155–155.7 °C. IR (ATR, cm^−1^): 3264, 3178, 2958, 2837, 1741, 1611, 1511, 1249, 1178, 1029, 801, 552. ^1^H NMR: (300 MHz, CDCl_3_) δ 7.38 (s, 1H), 7.11–7.00 (m, 2H), 6.99–6.88 (m, 2H), 6.73–6.62 (m, 2H), 6.58–6.47 (m, 2H), 5.50 (s, 1H), 3.89–3.82 (m, 1H), 3.68 (s, 3H), 3.61 (s, 3H), 1.97–1.80 (m, 1H), 1.44–1.17 (m, 2H), 1.21–1.02 (m, 1H), 0.94–0.73 (m, 4H), 0.78–0.61 (m, 7H). ^13^C NMR: (75 MHz, CDCl_3_) δ = 159.01, 158.28, 149.60, 133.76, 130.09, 127.72, 127.07, 121.55, 113.31, 113.09, 79.51, 73.50, 55.13, 55.08, 37.13, 32.19, 28.08, 22.81, 22.19, 7.87, 7.83. HRMS (DART, [M+1]^+^) *m*/*z* calcd. For C_26_H_34_N_3_O_3_: 436.2600; found: 436.2594.

*2-{1-[2-Hydroxy-1,2-bis-(4-methoxyphenyl)-5-methyl-hexyl]-[1,2,3]triazol-4-ylmethyl}-isoindole-1,3-dione (* **48** *)*

1-Azido-1,2-bis-(4-methoxyphenyl)-5-methyl-hexan-2-ol and 2-prop-2-ynyl-isoindole-1,3-dione afforded 2-{1-[2-Hydroxy-1,2-bis-(4-methoxyphenyl)-5-methyl-hexyl]-[1,2,3]triazol-4-ylmethyl}-isoindole-1,3-dione **48** as a white solid. Yield: 288 mg (52%), m.p.: 173.3–174.4 °C. IR (ATR, cm^−1^): 3462, 3145, 2954, 2868, 2837, 1703, 1613, 1515, 1398, 1254, 1097, 764, 715, 521. ^1^H NMR: (300 MHz, CDCl_3_) δ 7.90–7.79 (m, 3H), 7.72 (dt, *J* = 5.4, 3.3 Hz, 2H), 7.17–6.98 (m, 5H), 6.81–6.70 (m, 2H), 6.66–6.55 (m, 2H), 5.63 (s, 1H), 5.02 (s, 3H), 3.72 (d, *J* = 22.6 Hz, 6H), 1.95 (td, *J* = 13.0, 4.3 Hz, 1H), 1.34 (qd, *J* = 12.6, 5.3 Hz, 2H), 1.14 (tt, *J* = 12.4, 5.1 Hz, 1H), 0.69 (dd, *J* = 6.6, 1.6 Hz, 8H). ^13^C NMR: (75 MHz, CDCl_3_) δ = 167.63, 159.08, 158.32, 134.08, 133.57, 132.05, 130.19, 127.28, 127.03, 124.62, 123.46, 113.34, 113.15, 79.47, 73.80, 55.12, 55.08, 37.02, 33.02, 32.12, 28.00, 22.70, 22.17. HRMS (DART, [M+1]^+^) *m*/*z* calcd. For C_32_H_35_N_4_O_5_: 555.2607; found: 555.2597.

*1-[4-(2,6-Diisopropylphenoxymethyl)-[1,2,3]triazol-1-yl]-1,2-bis-(4-methoxyphenyl)-5-methyl-hexan-2-ol (* **49** *)*

1-Azido-1,2-bis-(4-methoxyphenyl)-5-methyl-hexan-2-ol and 1,3-diisopropyl-2-prop-2-ynyloxybenzene afforded 1-[4-(2,6-diisopropylphenoxymethyl)-[1,2,3]triazol-1-yl]-1,2-bis-(4-methoxy-phenyl)-5-methyl-hexan-2-ol **49** as a pale- yellow oil. Yield: 288 mg (55%), colorless oil. IR (ATR, cm^−1^): 3453, 2958, 2866, 1741, 1608, 1512, 1447, 1249, 1177, 1119, 1033, 802. ^1^H NMR: (300 MHz, CDCl_3_) δ 7.84 (s, 1H), 7.13–7.04 (m, 2H), 7.05 (s, 3H), 7.03–6.95 (m, 2H), 6.74–6.65 (m, 2H), 6.61–6.52 (m, 2H), 5.64 (s, 1H), 4.90 (s, 2H), 3.69 (s, 3H), 3.63 (s, 3H), 3.30 (hept, *J* = 6.9 Hz, 2H), 1.94 (td, *J* = 13.5, 4.7 Hz, 1H), 1.47–1.35 (m, 1H), 1.33–1.22 (m, 1H), 1.15 (d, *J* = 6.9 Hz, 13H), 0.80–0.68 (m, 1H), 0.73 (s, 1H), 0.67 (dd, *J* = 6.6, 2.9 Hz, 6H). ^13^C NMR: (75 MHz, CDCl_3_) δ = 193.49, 159.17, 158.40, 152.88, 144.28, 141.87, 133.65, 130.20, 127.49, 127.06, 125.03, 124.15, 124.11, 113.41, 113.23, 79.55, 73.83, 68.14, 55.14, 55.12, 37.22, 32.22, 28.08, 26.64, 24.06, 22.80, 22.19, 1.85. HRMS (DART, [M+1]^+^) *m*/*z* calcd. For C_36_H_48_N_3_O_4_: 586.3645; found: 586.3636.

*1,2-Bis-(4-methoxyphenyl)-5-methyl-1-(4-morpholin-4-ylmethyl-[1,2,3]triazol-1-yl)-hexan-2-ol (* **50** *)*

1-Azido-1,2-bis-(4-methoxyphenyl)-5-methyl-hexan-2-ol and 4-prop-2-ynyl-morpholine afforded 1,2-bis-(4-methoxyphenyl)-5-methyl-1-(4-morpholin-4-ylmethyl-[1,2,3]triazol-1-yl)-hexan-2-ol **50** as a white solid. Yield: 296 mg (60%), 153.5–154. IR (ATR, cm^−1^): 3364, 2951, 2838, 1741, 1609, 1512, 1457, 1246, 1177, 1030, 803. ^1^H NMR: (300 MHz, CDCl_3_) δ 7.79 (s, 1H), 7.16–7.02 (m, 4H), 6.78–6.70 (m, 2H), 6.65–6.57 (m, 2H), 5.68 (s, 1H), 3.74 (s, 3H), 3.68 (s, 2H), 3.64 (dd, *J* = 3.9, 2.0 Hz, 6H), 3.18 (s, 4H), 2.00–1.84 (m, 2H), 1.30 (dq, *J* = 13.6, 5.0 Hz, 2H), 1.12 (td, *J* = 12.7, 6.0 Hz, 1H), 0.73–0.65 (m, 6H). ^13^C NMR: (75 MHz, CDCl_3_) δ = 177.70, 159.11, 158.33, 142.91, 133.72, 130.24, 127.45, 127.03, 124.53, 113.36, 113.22, 79.46, 73.59, 70.57, 66.90, 66.57, 61.62, 55.12, 53.22, 37.07, 32.05, 28.07, 22.55, 22.22. HRMS (DART, [M+1]^+^) *m*/*z* calcd. For C_28_H_39_N_4_O_4_: 495.2971; found: 495.2957.

### 3.3. Tumor Cell Lines and Culture Medium

The compounds were screened in vitro against human cancer cell lines: HCT-15 (human colorectal adenocarcinoma), MCF-7 (human mammary adenocarcinoma), K562 (human chronic myelogenous leukemia), U251 (human glioblastoma), PC-3 (human pro- static adenocarcinoma) which were supplied by National Cancer Institute (Bethesda, MD, USA); SKLU-1 (human lung adenocarcinoma) from National Institute of Cancerology, Mexico, and COS-7, adherent monkey kidney fibroblasts (ATTC).

The human tumor cytotoxicity was determined using the protein-binding dye sulforhodamine B (SRB) in microculture assay to measure cell growth, as described in the protocols established by the NCI [15]. The cell lines were cultured in RPMI-1640 medium supplemented with 10% fetal bovine serum, 2 mM L-glutamine, Penicillin-Streptomycin solution 100x (Gibco, Waltham, MA, USA) and 1% non-essential amino acids (Gibco^®^). They were maintained at 37 °C in humidified atmosphere with 5% CO_2_. The viability of the cells used in the experiments exceed 95% as determined with trypan blue.

#### Cytotoxicity Assay

Cytotoxicity after treatment of the tumors cells and normal cell with the test compounds was determined using the protein-binding dye sulforhodamine B (SRB) in microculture assay to measure cell growth as described in the reference [49]. The cells were removed from the tissue culture flasks by treatment with trypsin and diluted with fresh media. Of this cell suspension, 100 μL containing 5000–10,000 cell per well, were pipetted into 96 well microtiter plates (Costar) and the material was incubated at 37 °C for 24 h in a 5% CO_2_ atmosphere. Each cell line is deposited by triplicate and subsequently, 100 μL of a solution of the compound obtained by diluting the stocks were added to each well. The cultures were exposed for 48 h to the compound at concentrations 25 μM. After the incubation period, cells were fixed to the plastic substratum by the addition of 50 μL of cold 50% aqueous trichloroacetic acid. The plates were incubated at 4 °C for 1 h, washed with tap H_2_O, and air-dried. The trichloroacetic-acid-fixed cells were stained by the addition of 0.4% SRB. Free SRB solution was the removed by washing with 1% aqueous acetic acid. The plates were the air-dried, and the bound dye was solubilized by the addition of 10 mM un-buffered Tris base (100 μL). The plates were placed on a shaker for 10 min, and the absorption was determined at 515 nm using an ELISA plate reader (Bio-Tex Instruments, Bio-Tek Instruments, Winooski, VT, USA).

### 3.4. In Vitro Antimicrobial Activity

The antimicrobial activity of compounds **11**–**50** was screened using a reference strain from American Type Culture Collection, *Staphylococcus aureus* ATCC 29213, and a clinical isolate of *Candida albicans* obtained from a urine culture of a 34-year-old female patient. This strain was provided by the Laboratorio de Especialidades Clínicas de San Jerónimo (Tacámbaro, Michoacán, Mexico). Identification was performed using an automated identification system VITEK^®^ 2, ensuring high taxonomic reliability. All isolates were maintained on Sabouraud Dextrose Agar (SDA) at 37 °C for 24 h prior to biological assays.

## 4. Conclusions

A library of 1,2-diphenyl-2-[1,2,3]triazol-1-yl-ethanol derivatives was obtained from CuAAC reaction as the key reaction. Triazoles **11** and **30** have crystal structures that reveal significant O-H···N interactions, which are involved in the crystal form in these compounds. These interactions play a key role in shaping each structure. Synthesized 1,2-diphenyl-2-[1,2,3]triazol-1-yl-ethanol derivatives exhibited significant cytotoxic activity, which is enhanced by the presence of longer alkyl chains, providing an important starting point for future research. In addition, some of these compounds presented interesting selective inhibition activity against *C. albicans* and *S. aureus* strains. Worthy of mention is the use of benzoin as a privileged structure, which facilitated the preparation of target molecules, providing an alternative source for the synthesis and development of new therapeutic options in the field of medicinal chemistry, so these new molecules as well as the synthetic protocols will find widespread applications.

## Figures and Tables

**Table 1:** Synthesis of 1,2-diphenyl-2-[1,2,3]triazol-1-yl-ethanol derivatives.

Compound	R^1^	R^2^	R^3^	% Yield
**11**	H	CH_3_CH_2_-	Ph	57
**12**	H	CH_3_CH_2_-	Cyclopropyl	62
**13**	H	CH_3_CH_2_-	(1,3-dioxoisoindolin-2-yl)methyl	52
**14**	H	CH_3_CH_2_-	(2,6-diisopropylphenoxy)methyl	55
**15**	H	CH_3_CH_2_-	morpholin-4-ylmethyl	64
**16**	H	CH_3_CH_2_CH_2_CH_2_-	Ph	35
**17**	H	CH_3_CH_2_CH_2_CH_2_-	Cyclopropyl	60
**18**	H	CH_3_CH_2_CH_2_CH_2_-	(1,3-dioxoisoindolin-2-yl)methyl	71
**19**	H	CH_3_CH_2_CH_2_CH_2_-	(2,6-diisopropylphenoxy)methyl	73
**20**	H	CH_3_CH_2_CH_2_CH_2_-	morpholin-4-ylmethyl	62
**21**	H	PhCH_2_-	Ph	43
**22**	H	PhCH_2_	Cyclopropyl	59
**23**	H	PhCH_2_	(1,3-dioxoisoindolin-2-yl)methyl	44
**24**	H	PhCH_2_	(2,6-diisopropylphenoxy)methyl	41
**25**	H	PhCH_2_	morpholin-4-ylmethyl	48
**26**	H	Cyclohexyl	Ph	61
**27**	H	Cyclohexyl	Cyclopropyl	43
**28**	H	Cyclohexyl	(1,3-dioxoisoindolin-2-yl)methyl	64
**29**	H	Cyclohexyl	(2,6-diisopropylphenoxy)methyl	30
**30**	H	Cyclohexyl	morpholin-4-ylmethyl	48
**31**	OMe	CH_3_CH_2_-	Ph	61
**32**	OMe	CH_3_CH_2_-	Cyclopropyl	66
**33**	OMe	CH_3_CH_2_-	(1,3-dioxoisoindolin-2-yl)methyl	45
**34**	OMe	CH_3_CH_2_-	(2,6-diisopropylphenoxy)methyl	55
**35**	OMe	CH_3_CH_2_-	morpholin-4-ylmethyl	60
**36**	OMe	CH_3_CH_2_CH_2_CH_2_-	Ph	50
**37**	OMe	CH_3_CH_2_CH_2_CH_2_-	Cyclopropyl	38
**38**	OMe	CH_3_CH_2_CH_2_CH_2_-	(1,3-dioxoisoindolin-2-yl)methyl	42
**39**	OMe	CH_3_CH_2_CH_2_CH_2_-	(2,6-diisopropylphenoxy)methyl	46
**40**	OMe	CH_3_CH_2_CH_2_CH_2_-	morpholin-4-ylmethyl	41
**41**	OMe	PhCH_2_	Ph	46
**42**	OMe	PhCH_2_	Cyclopropyl	50
**43**	OMe	PhCH_2_	(1,3-dioxoisoindolin-2-yl)methyl	40
**44**	OMe	PhCH_2_	(2,6-diisopropylphenoxy)methyl	38
**45**	OMe	PhCH_2_	morpholin-4-ylmethyl	51
**46**	OMe	3-Methylbutyl	Ph	63
**47**	OMe	3-Methylbutyl	Cyclopropyl	46
**48**	OMe	3-Methylbutyl	(1,3-dioxoisoindolin-2-yl)methyl	52
**49**	OMe	3-Methylbutyl	(2,6-diisopropylphenoxy)methyl	55
**50**	OMe	3-Methylbutyl	morpholin-4-ylmethyl	60

**Table 2:** Crystallographic data for the structural analysis of compounds **11** and **30**.

Crystal Data	11	30
Empirical formula	C_24_H_23_N_3_O	C_27_H_34_N_4_O_2_
Formula weight	369.45	446.58
Temperature (K)	100(2)	100(2)
Radiation type	Mo Kα	Mo Kα
Crystal system	Monoclinic	Monoclinic
Space group	*P*2_1_/c	*P*2_1_/n
Unit cell dimensions (Å, °)		
*a*	12.5197(3)	11.2048(5)
*b*	9.7981(2)	16.9893(8)
*c*	15.5740(4)	25.5635(12)
*α*	90	90
*β*	97.3272(9)	92.6407(10)
*γ*	90	90
Volume (Å^3^)	1894.85(8)	4861.1(4)
Z	4	8
Density (calculated, Mg/m^3^)	1.295	1.220
Absorption coefficient μ (mm^−1^)	0.081	0.078
F(000)	784	1920
Crystal size (mm^3^)	0.592 × 0.580 × 0.523	0.447 × 0.169 × 0.088
Θ range (deg)	2.020 to 27.445	
Index ranges	−16 h ≤ 16, −12 ≤ k ≤ 12, −20 ≤ l ≤ 20	−14 h ≤ 14, −22 ≤ k ≤ 22, −33 ≤ l ≤ 33
Reflections collected	40,152	54,823
Independent reflections	4331 [R(int) = 0.0338]	11,102 [R(int) = 0.0334]
Data/restraints/parameters	11,102/2/601	11,102/2/601
Goodness of fit on F2	1.025	1.022
Final R indices [I > 2sigma(I)]	R1 = 0.0364, wR2 = 0.0911	R1 = 0.0463, wR2 = 0.1144
R indices (all data)	R1 = 0.0388, wR2 = 0.0934	R1 = 0.0591, wR2 = 0.1223
Largest diff. peak and hole (e Å^−3^)	0.351, −0.251	0.530, −0.277

**Table 3:** Growth inhibition (%) of tumor cell lines at 25 μM by compounds **11**–**20** and **31**–**40** ^1^.

Compound	U251	PC-3	K562	HCT-15	MCF-7	SKLU-1	COS-7
**11**	25.44	57.55	50.54	73	89.26	80.27	78.66
**12**	23.08	30.67	37.24	10.29	9.51	36.25	23.25
**13**	9.12	19.52	24.35	1.13	7.2	12.06	16.64
**14**	52.7	60.87	41.41	60.8	55.69	66.73	6.44
**15**	17.76	12.74	30.34	9.98	12.81	36.43	19.26
**16**	16.81	46.20	NC ^2^	34.34	38.14	43.83	33.40
**17**	27.28	16.69	NC	21.25	25.58	40.57	20.61
**18**	18.13	17.54	NC	16.04	27.39	23.10	29.78
**19**	19.26	39.58	14.16	46.44	52.51	66.82	41.95
**20**	18.31	14.51	6.30	6.24	14.37	24.58	26.19
**31**	37.88	53.89	44.75	35.41	49.36	54.25	47.89
**32**	13.08	19.92	41.84	30.81	33.59	45.01	56.55
**33**	52.7	60.87	12.8	11.41	9.55	15.82	28.55
**34**	35.54	50.61	31.51	24.77	35.97	57.14	39.57
**35**	6.54	5.65	16.19	NC	4.28	14.51	13.28
**36**	16.74	19.44	34.06	32.39	51.23	67.04	33.51
**37**	10.43	15.63	NC	33.12	31.48	52.70	28.43
**38**	10.83	17.39	NC	13.90	41.18	44.50	32.78
**39**	6.20	21.34	1.74	26.00	45.44	56.74	30.05
**40**	12.29	22.18	32.05	12.79	18.57	31.91	26.00

^1^ The values indicate the mean calculated from experiments conducted in triplicate. ^2^ NC: not cytotoxic.

**Table 4:** Values of diameter of the inhibition zone in mm for selected compounds.

Compound	Diameter of the Inhibition Zones (mm)	
	*C. albicans*	*S. aureus* ATCC 29213
**15**	8	6
**16**	15	11
**19**	7	7
**20**	7	10
**21**	8	7
**22**	10	8
**23**	12	7
**24**	10	8
**26**	10	5
**27**	5	8
**28**	9	7
**36**	8	6
**37**	6	6
**38**	7	7
**39**	8	9
**41**	8	8
**43**	10	7
**44**	6	8
**46**	10	13
**48**	11	8
**49**	8	11
**50**	13	13
Amikacin	-	11
ketoconazole	15	-

## Data Availability

The raw data supporting the conclusions of this article will be made available by the authors on request.

## Supplementary Materials

The following supporting information can be downloaded at: https://www.mdpi.com/article/10.3390/molecules31010170/s1.
