# Supplementary material for: The Use of Benzoin as a Privileged Structure: Synthesis, Characterization, Crystalline Form and an In Vitro Biological Evaluation of 1,2-Diphenyl-2-[1,2,3]triazol-1-yl-ethanol Derivatives
Source: Molecules. 2026 Jan 1;31(1):170. doi: 10.3390/molecules31010170 (PMC12787705; doi:10.3390/molecules31010170)

Supplementary Material

"The Use of Benzoin as a Privileged Structure: Synthesis, Characterization, Crystalline Form and an In Vitro Biological Evaluation of 1,2-Diphenyl-2-[1,2,3]triazol-1-yl-ethanol Derivatives"

Noé Martínez-Romero [1], Mario Valle-Sánchez [1], Marco A. García-Eleno [1,2], Carlos A. González-González [2], David Corona-Becerril [1,2], Lizbeth Triana-Cruz [1,3], Diego Martínez-Otero [1,3], María Teresa Ramírez-Apan [3], David Morales-Morales [3], Jorge Andrés Ornelas-Guillén [4] and Erick Cuevas-Yañez [1,2,*]

[1] Centro Conjunto de Investigación en Química Sustentable UAEM-UNAM, Universidad Autónoma del Estado de México, Carretera Toluca-Atlacomulco Km 14.5, Toluca 50200, Estado de México, Mexico; nmartinezr@uaemex.mx (N.M.-R.); marvals_18@hotmail.com (M.V.-S.); magarciae@uaemex.mx (M.A.G.-E.); dcoronab@uaemex.mx (D.C.-B.); ltrianac@unam.mx (L.T.-C.); diegomtz@unam.mx (D.M.-O.)
[2] Facultad de Química, Universidad Autónoma del Estado de México, Paseo Colon esq. Paseo Tollocan, Toluca 50120, Estado de México, Mexico; cagonzalezg@uaemex.mx
[3] Instituto de Química, Universidad Nacional Autónoma de México, Circuito Exterior S. N., Ciudad Universitaria, Coyoacán 04510, Ciudad de México, Mexico; damor@unam.mx (D.M.-M.)
[4] Instituto de Investigaciones Químico-Biológicas, Universidad Michoacana de San Nicolás de Hidalgo, Ciudad Universitaria, Av. Fco. J. Múgica s/n, Morelia 58030, Michoacán, Mexico
* Correspondence: ecuevasy@uaemex.mx; Tel.: +52-722-276-6610 (ext. 7734); Fax: +52-722-217-5109

CONTENTS:

1. General considerations

2. General procedures for the synthesis of Compounds.

3. Spectroscopic data of compounds

4. ^{1}H NMR and ^{13}C NMR spectra for compounds

5. Crystallographic data for compounds

1. - General Considerations

The starting materials were purchased from Aldrich Chemical Co., Meyer, and J.T. Baker. Anisoin (4,4′-Dimethoxybenzoin) was prepared from the condensation of p-methoxy benzaldehyde. Solvents were dried and distilled before use. Silica gel (230–400 mesh) and silica plates of 0.20 mm thickness were purchased from Merck. Melting points were determined with a Krüss Optronic KSP1N melting point apparatus and they are uncorrected.

^{1}H and ^{13}C NMR spectra were recorded using a Bruker Avance 300 MHz, and a Varian 500 MHz using deuterated chloroform, the chemical shifts (δ) are given in ppm relative to TMS as internal standard (0.00). For analytical purposes the mass spectra were recorded on a JEOL AccuTOF JMS-T100LP using DART technology. Only the molecular and parent ions (m/z) are reported. IR spectra were obtained with a Bruker TENSOR 27 FT instrument.

2. - General procedures for the synthesis of Compounds.

2.1. Synthesis of 2-Azido-1,2-diphenylethanones 3.

Typical procedure. The corresponding benzoin (10 mmol) was added to a mixture of dry toluene (20 mL) and activated charcoal (0.04 g). Thionyl chloride (1.19 g) was added in one portion via syringe addition maintaining room temperature using a water bath under a nitrogen atmosphere. A vigorous evolution of gas occurred, and the reaction mixture was stirred at 50 °C for 1 h. The mixture was cooled to room temperature and thionyl chloride and toluene were removed under reduced pressure. The mixture was filtered through celite, and the product was purified by crystallization (hexane). The synthesized 2-chloro-1,2-diphenylethanone (10 g) was added to a mixture of sodium azide (3.099 g, 47.67 mmol) and 18-crown-6 (0.078g, 0.2167 mmol) in acetonitrile (150 mL). The reaction mixture was stirred for 1 h at room temperature under a nitrogen atmosphere. The mixture was filtered and the solvent was removed under reduced pressure. The compound was purified by crystallization (petroleum ether).

2.2. General procedure for the synthesis of 1,2-Diaryl-2-azidoethan-1-ol derivatives.

Typical procedure. The corresponding 1M solution of Grignard reagent in diethyl ether (1.5-1.8 equivalents) was added dropwise via a syringe to a solution of 2-azido-1,2-diphenylethanone **2** (10 mmol) in diethyl ether (30 mL) at 0 °C. After Grignard reagent completion, the reaction mixture was stirred for 10 min at room temperature. A 20% solution of ammonium chloride (25 mL) was added and the product was extracted with CH_2Cl_2 (3 X 30 mL), the organic layers were joined and dried over Na_2SO_4 and the solvent was removed under reduced pressure. The final product was purified by column chromatography (SiO_2, hexane /AcOEt 95:5).

2.3. General procedure for the synthesis of 2-diphenyl-2-[1,2,3]triazol-1-yl-ethanol derivatives.

Typical procedure. The appropriate azido alcohol (1 mmol) was added to a to a mixture of PEG-400 (3.2 mL) and H_2O (0.8 mL), which was then gently warmed until the azido alcohol was fully dissolved. CuBr (0.014 g, 0.1 mmol), sodium ascorbate (0.019 g, 0.1 mmol), DIPEA (0.07 mL, 0.051 g, 4 mmol) and the resulting mixture was gently warmed 8:2 mixture of 1-azido-3-nitro-5- (trifluoromethyl) benzene (0.92 mmol, 1 eq), the appropriate alkyne (1.09 mmol, 1.2 eq), CuI (0.13 mmol, 0.15 eq), DIPEA (4.58 mmol, 5.0 eq) and the corresponding alkyne (1.5 mmol) were added successively and the resulting mixture was stirred at 38-45°C overnight. The mixture was cooled to room temperature and H_2O (40 mL) was added. The product was filtered and washed with cold H_2O followed by cold THF and AcOEt. The product was dried under reduced pressure and purified by crystallization.

3. Spectroscopic data of compounds

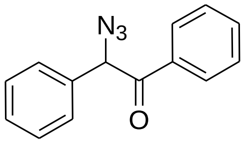

2-Azido-1,2-diphenyl-ethanone

Yield: 65%, **Melting point:** 73-74.5ºC. **FT-IR (ATR cm^{-1}):** 2090, 1682, 1210. **^{1}H RMN (CDCl3, 500 MHz):** 7.89 (d, 2H), 7.52 (m, 1H), 7.39 (m, 7H), 5.72 (s, 1H). **^{13}C RMN (CDCl3, 126 MHz):** 67.89, 128.31, 128.78, 128.87, 129.38, 129.56, 133.76, 133.81, 134.34, 194.34. **HRMS (DART, [M+1]$^{+}$)** m/z calcd. For $C_{14}H_{12}N_3O$: 238.0980; found: 297.0940.

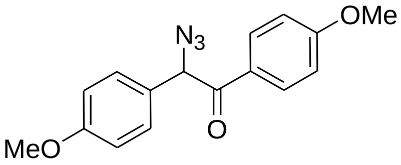

2-Azido-1,2-bis-(4-methoxy-phenyl)-ethanone

Yield: 60%, **Melting point:** colorless oil. **FT-IR (ATR cm^{-1}):** 2096, 1678, 1252. **^{1}H RMN (CDCl3, 300 MHz):** *δ* 7.87 (m, 2H), 7.33 (m, 2H), 6.90 (m, 4H), 5.66 (s, 1H), 3.81 (d, 6H).

^{13}C RMN (CDCl3, 75 MHz) *δ* 192.92, 163.89, 160.24, 131.49, 131.24, 129.84, 129.66, 62.09, 114.87, 114.05, 67.12. **HRMS (DART, [M+1]$^{+}$)** m/z calcd. For $C_{16}H_{16}N_3O_3$: 298.1192; found: 298.1081

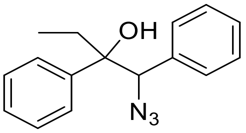

1-azido-1,2-diphenylbutan-2-ol (A)

Yield: 48%, **Melting point:** 57.6-58ºC. **IR (ATR, cm^{-1}):** 3496, 3031, 2983, 2944, 2098, 1220. **^{1}H RMN (CDCl3, 300 MHz)** δ 7.20 (m, 8H), 7.02 (m, 2H), 4.72 (s, 1H), 2.24 (s, 1H), 2.07 (q, 2H), 0.74 (t, 3H). **^{13}C RMN (CDCl3, 500 MHz)** δ 140.82, 135.50, 128.11, 127.81, 127.71, 126.87, 126.29, 79.23, 75.15, 31.33, 7.52 **HRMS (DART, [M+1] $^{+}$)** ***m/z*** calculated for: $C_{16}H_{17}N_3O$: 267.13716; found: 268.14444

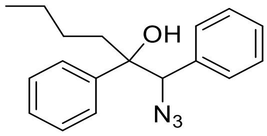

1-azido-1,2-diphenylhexan-2-ol

Yield: 62%, **Melting point:** 42-42.5ºC. **IR (ATR, cm^{-1}):** 3507, 3031, 2037, 2871, 2103, 1224. **^{1}H RMN (CDCl3, 300 MHz):** δ 7.10 (m, 8H), 6.97 (m, 2H), 4.64 (s, 1H), 2.09 (s, 1H), 1.96 (m, 2H), 1.20 (m, 3H), 0.88 (m, 1H), 0.75 (t, 3H). **^{13}C RMN (CDCl3, 300 MHz):** δ 141.33, 135.48, 128.83, 128.12, 127.83, 127.73, 126, 83, 126.17, 78.98, 75.31, 38.55, 25.39, 23.02, 14.01. **HRMS-DART [M+1]$^{+}$ *m/z*:** Not observed. **[M-N_2]** calculated for $C_{18}H_{21}NO$: 267.16231; found: 269.16959

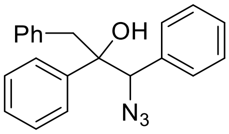

1-azido-1,2,3-triphenylpropan-2-ol

Yield: 57.6**, Melting point:** 90-95.5ºC.

IR (ATR, cm^{-1}): 3558, 3028, 2933, 2121, 1202, 698. **^{1}H RMN (CDCl3, 500 MHz):** δ 7.18 (m, 14H), 6.95 (m, 2H), 4.91 (s, 1H), 3.45 (m, 2H). **^{13}C RMN (CDCl3, 500 MHz):** δ 141.27, 135.54, 135.36, 130.75, 129.17, 128.19, 128.07, 127.90, 127.62, 126.97, 126.75, 126.44, 79.23, 73.64, 45.48. **HRMS-DART [M+1]$^{+}$ *m/z*:** Not observed. **[M-N_2]** calculated for $C_{21}H_{19}NO$: 301.14666; found: 302.15394

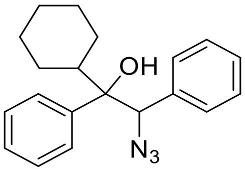

2-azido-1-cyclohexyl-1,2-diphenylethan-1-ol

Yield: 32%**, Melting point:** 79.70.8ºC. **IR (ATR, cm^{-1}):** 3518, 3031, 2929, 2854, 2102, 1680, 1223, 696. **^{1}H RMN (CDCl3, 300 MHz):** δ 7.21 (m, 10H), 5.26 (s, 1H), 2.33 (s, 1H), 1.37 (m, 11H) **^{13}C RMN (CDCl3, 300 MHz):** δ 139.66, 135.29, 128.84, 127.72, 127.56,

127.76, 126.40, 126.33, 80.28, 69.91, 45.60, 27.78, 26.39, 26.11, 25.96. **HRMS-DART [M+1]** $^+$ ***m/z**:* Not observed. **[M-N_2]** calculated for $C_{20}H_{23}NO$: 293.17796; found: 294.18524

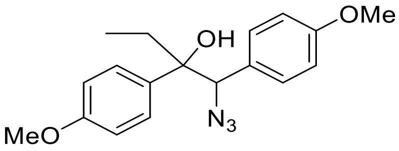

1-azido-1,2-bis(4-methoxyphenyl) butan-2-ol

Yield: 52%, **Melting point:** 84.5-85.3ºC. **IR (ATR cm^{-1}):** 3505, 2967, 2936, 2100, 1610, 1510, 1243, 1031, 799. **^{1}H NMR (300 MHz, $CDCl_3$) :** δ 7.15 – 7.06 (m, 2H), 7.03 – 6.96 (m, 2H), 6.83 – 6.71 (m, 4H), 4.70 (s, 1H), 3.78 (d, *J* = 4.8 Hz, 6H), 2.15 (s, 1H), 2.03 (qd, *J* = 7.3, 1.2 Hz, 2H), 0.77 (t, *J* = 7.3 Hz, 3H). **^{13}C NMR (75 MHz, $CDCl_3$) :** δ 159.30, 158.37, 133.03, 130.01, 127.74, 127.62, 113.25, 113.04, 79.05, 74.85, 55.15, 31.26, 7.53. **HRMS-DART [M+1]** $^+$ ***m/z**:* Not observed. **[M-46.02]** calculated for $C_{18}H_{19}NO_2$: 281.14158; found: 282.14886

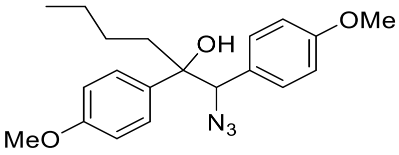

1-azido-1,2-bis(4-methoxyphenyl)-5-methylhexan-2-ol

Yield: 63%, **Melting point:** 62.1-62.5ºC. **IR (ATR cm^{-1}):** 3502, 2955, 2934, 2838, 2101, 1607, 1511, 1245, 1032, 803. **^{1}H NMR (300 MHz, $CDCl_3$)** δ 7.08 – 7.02 (m, 2H), 6.98 – 6.92 (m, 2H), 6.74 (ddt, J = 15.5, 6.5, 1.9 Hz, 4H), 4.66 (d, J = 1.8 Hz, 1H), 3.76 (dd, J = 7.5, 1.8 Hz, 6H), 2.21 (s, 1H), 1.96 (td, J = 7.0, 2.8 Hz, 2H), 1.32 – 1.21 (m, 3H), 1.02 – 0.93 (m, 1H), 0.83 (td, J = 7.3, 2.0 Hz, 3H). **^{13}C NMR (75 MHz, $CDCl_3$)** δ 156.69, 156.65, 155.74, 130.90, 127.67, 127.42, 127.17, 125.07, 124.88, 124.64, 110.75, 110.64, 110.56, 110.54, 110.42, 110.29, 76.18, 72.48, 72.29, 52.77, 52.62, 52.54, 52.48, 52.46, 35.87, 22.81, 20.48. **HRMS-DART [M+1]** $^+$ ***m/z**:* Not observed. **[M-16.02]** calculated for $C_{20}H_{23}N_2O_3$: 339.17087; found: 340.17814

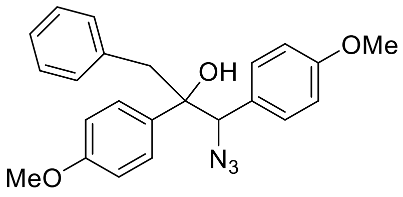

1-azido-1,2-bis(4-methoxyphenyl)-3-phenylpropan-2-ol

Yield: 68%, **Melting point:** colorless oil. **IR (ATR cm^{-1}):** 3552, 3029, 2934, 2102, 1608, 1511, 1244, 1031, 700. **^{1}H NMR (300 MHz, CDCl$_3$)** δ 7.17 – 7.06 (m, 5H), 7.03 (td, J = 6.9, 3.1 Hz, 3H), 6.96 – 6.89 (m, 2H), 6.77 – 6.68 (m, 3H), 4.81 (s, 1H), 3.76 – 3.70 (m, 6H), 3.35 (t, J = 3.1 Hz, 1H), 3.33 (s, 1H). **^{13}C NMR (75 MHz, CDCl$_3$)** δ 159.27, 158.34, 135.73, 133.40, 130.78, 130.34, 128.01, 127.68, 127.54, 126.64, 113.29, 112.87, 79.04, 73.25, 65.85, 55.10, 55.06, 45.47, 15.28. **HRMS-DART [M+1]$^+$ *m/z:*** Not observed. **[M-N$_2$]** calculated for $C_{23}H_{23}NO_3$: 361.16779; found: 362.17507

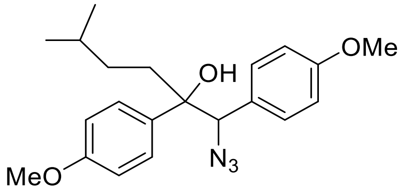

1-azido-1,2-bis(4-metoxifenil)-5-metilhexan-2-ol

Yield: 54%, **Melting point:** 76-77ºC. **FT-IR (ATR cm^{-1}):** 3505, 2954, 2868, 2101, 1610, 1510, 1244, 1033, 828, 804. **^{1}H NMR (300 MHz, CDCl$_3$) :** δ 7.08 – 7.01 (m, 2H), 6.98 – 6.91 (m, 2H), 6.79 – 6.69 (m, 4H), 4.66 (s, 1H), 3.76 (d, J = 7.6 Hz, 6H), 2.22 (s, 1H), 2.02 – 1.92 (m, 2H), 1.48 (hept, J = 6.6 Hz, 1H), 1.29 – 1.16 (m, 2H), 0.83 (dd, J = 11.1, 6.6 Hz, 6H). **^{13}C NMR (75 MHz, CDCl$_3$)** δ 159.26, 158.30, 133.42, 129.98, 129.88, 127.65, 127.45, 114.04, 113.21, 113.00, 118.00, 118.00, 78.75, 75.03, 55.11, 55.10, 36.47, 32.06, 28.36, 22.73, 22.40, 22.31, 15.27. **HRMS-DART [M+1]$^+$ *m/z:*** Not observed. **[M-N$_2$]** calculated for $C_{21}H_{27}NO_3$: 341.19909; found: 342.20637

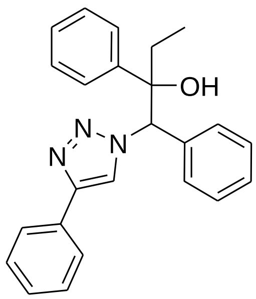

1,2-Diphenyl-1-(4-phenyl-[1,2,3]triazol-1-yl)-butan-2-ol (11)

Yield: 210 mg (57.1 %), m.p. 196 - 197.5 °C**. IR (ATR, cm^{-1}):** 3315, 3168, 3031, 2961, 2929, 1954, 1879, 1814, 1604, 1444**. ^{1}H NMR: (300 MHz, DMSO-d$_6$) δ =** 8.81 (s, 1H), 8.02 – 7.92 (m, 2H), 7.61 – 7.52 (m, 2H), 7.57 – 7.44 (m, 4H), 7.43 – 7.32 (m, 1H), 7.25 (t, *J* = 7.6 Hz, 2H), 7.23 – 7.08 (m, 4H), 6.30 (s, 1H), 5.71 (s, 1H), 2.07 (dq, *J* = 14.7, 7.4 Hz, 1H), 1.42 (dt, *J* = 14.3, 7.1 Hz, 1H), 0.59 (t, *J* = 7.3 Hz, 3H)**. ^{13}C NMR: (75 MHz, DMSO-d$_6$) δ =** 146.59, 143.32, 137.16, 131.30, 130.16, 129.30, 128.28, 128.18, 128.04, 126.77, 126.60, 125.73, 122.00, 79.28, 72.59, 32.84, 8.10**. HRMS (DART, [M+1]$^+$)** m/z calcd. For $C_{24}H_{24}N_3O$: 370.1919; found: 370.1911.

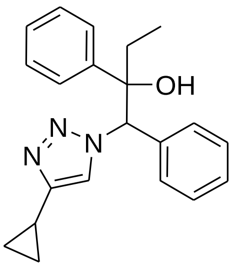

1-(4-Cyclopropyl-[1,2,3]triazol-1-yl)-1,2-diphenyl-butan-2-ol (12)

Yield: 206 mg (62.0 %), m.p. 171 °C**. IR (ATR, cm^{-1}):** 3385, 3134, 3091, 2972, 2937, 1741, 1452, 1046, 819**. ^{1}H NMR: (300 MHz, CDCl$_3$) δ =** 7.43 (s, 1H), 7.23 – 6.91 (m, 10H), 5.59 (s, 1H), 3.97 (s, 1H), 2.04 – 1.79 (m, 2H), 1.46 (dq, *J* = 14.4, 7.3 Hz, 1H), 0.93 – 0.73 (m, 4H), 0.59 (t, *J* = 7.3 Hz, 3H)**. ^{13}C NMR: (75 MHz, CDCl$_3$) δ =** 149.69, 141.11, 135.31, 128.87, 127.99, 127.82, 127.69, 126.91, 125.96, 121.75, 79.98, 73.74, 50.70, 32.84, 7.56, 6.68**. HRMS (DART, [M+1]$^+$)** m/z calcd. For $C_{21}H_{24}N_3O$: 334.1915; found: 334.1917**.**

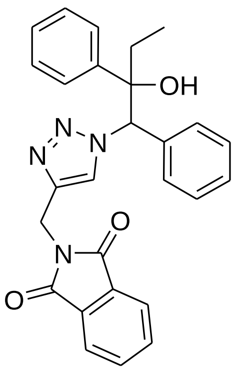

2-[1-(2-Hydroxy-1,2-diphenyl-butyl)-[1,2,3]triazol-4-ylmethyl]-isoindole-1,3-dione (13)

Yield: 235 mg (52.0 %), m.p. : 148.2–149 °C**. IR (ATR, cm^{-1}):** 3496, 3292, 2969, 2939, 1711, 1395, 1094, 706**.** ^{1}**H NMR: (300 MHz, $CDCl_3$) δ =** 7.84 – 7.70 (m, 4H), 7.63 (dddd, *J* = 7.9, 6.9, 4.8, 4.0 Hz, 3H), 7.21 – 6.88 (m, 8H), 5.64 (s, 1H), 4.92 (s, 2H), 4.37 (d, *J* = 2.5 Hz, 1H), 1.94 (dq, *J* = 14.6, 7.3 Hz, 1H), 1.41 (dq, *J* = 14.3, 7.2 Hz, 1H), 0.56 (t, *J* = 7.3 Hz, 3H)**.** ^{13}**C NMR: (75 MHz, $CDCl_3$)** δ = 167.68, 142.28, 140.97, 134.12, 132.05, 128.98, 128.02, 127.95, 127.76, 126.96, 125.95, 124.76, 123.49, 79.93, 74.06, 33.02, 27.00, 7.53**. HRMS (DART, $[M+1]^+$)** m/z calcd. For $C_{27}H_{25}N_4O_3$: 453.1927; found: 453.1954.

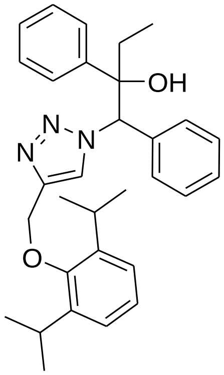

1-[4-(2,6-Diisopropylphenoxymethyl)-[1,2,3]triazol-1-yl]-1,2-diphenyl-butan-2-ol (14)

Yield: 265 mg (55.0 %), m.p. : 146.5-147.4 °C**. IR (ATR, cm^{-1}):** 3432, 3058, 3032, 2963, 2868, 1740, 1448, 1191, 1036, 698**.** ^{1}**H NMR: (300 MHz, $CDCl_3$) δ =** 7.87 (s, 1H), 7.23 – 6.92 (m, 10H), 7.03 (s, 3H), 5.74 (s, 1H), 4.89 (s, 2H), 3.73 (s, 1H), 3.29 (p, *J* = 6.9 Hz, 2H), 2.00 (dq, *J* = 14.6, 7.4 Hz, 1H), 1.58 – 1.41 (m, 1H), 1.12 (d, *J* = 6.9 Hz, 12H), 0.60 (t, *J* = 7.3 Hz, 3H)**.** ^{13}**C NMR: (75 MHz, $CDCl_3$) δ =** 152.86, 144.37, 141.90, 141.03, 135.11,

129.01, 128.13, 128.06, 127.86, 127.09, 125.99, 125.10, 124.39, 124.20, 80.04, 74.13, 68.11, 32.19, 26.66, 24.13, 7.60. **HRMS (DART, $[M+1]^+$)** m/z calcd. For $C_{31}H_{38}N_3O_2$: 484.2964; found: 484.2955.

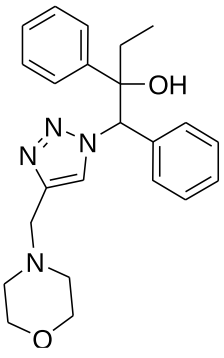

1-(4-Morpholin-4-ylmethyl-[1,2,3]triazol-1-yl)-1,2-diphenyl-butan-2-ol (15)

Yield: 251 mg (64.2 %), m.p. : 152.7-153.3 °C. **IR (ATR, cm^{-1}):** 3194, 3144, 3066, 3030, 2966, 2930, 2894, 2816, 1741, 1663, 1604, 1558, 1495, 1450, 1210, 1114, 1003, 697. **^{1}H NMR: (300 MHz, $CDCl_3$) δ =** 7.78 (s, 1H), 7.33 – 6.98 (m, 10H), 5.76 (s, 1H), 3.83 (s, 1H), 3.71 (q, *J* = 3.4 Hz, 6H), 2.57 – 2.48 (m, 4H), 2.06 (dq, *J* = 14.6, 7.4 Hz, 1H), 1.59 – 1.40 (m, 1H), 0.67 (t, *J* = 7.3 Hz, 3H). **^{13}C NMR: (75 MHz, $CDCl_3$) δ =** 143.36, 141.05, 135.14, 128.95, 128.06, 127.98, 127.82, 127.01, 125.95, 124.60, 79.95, 73.94, 66.75, 53.62, 53.36, 32.04, 7.56. **HRMS (DART, $[M+1]^+$) m/z** calcd. For $C_{23}H_{29}N_4O_2$: 393.2291; found: 393.2955.

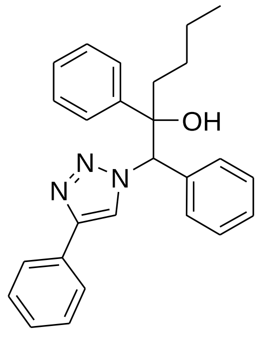

1,2-Diphenyl-1-(4-phenyl-[1,2,3]triazol-1-yl)-hexan-2-ol (16)

Yield: 138 mg (35.0 %), m.p. : 184.4-185.2 °C. **IR (ATR, cm^{-1}):** 3463, 3142, 3030, 2959, 2930, 2863, 1955, 1882, 1768, 1448, 1159, 761, 697. **^{1}H NMR: (300 MHz, $CDCl_3$) δ =** 8.00 (d, *J* = 2.5 Hz, 1H), 7.84 – 7.75 (m, 2H), 7.35 (t, *J* = 7.4 Hz, 2H), 7.31 – 6.95 (m, 11H), 5.74

(d, *J* = 2.7 Hz, 1H), 3.76 (d, *J* = 4.7 Hz, 1H), 2.00 (ddd, *J* = 16.7, 12.4, 4.5 Hz, 1H), 1.48 (dd, *J* = 13.6, 4.2 Hz, 1H), 1.24 – 1.00 (m, 1H), 1.06 (s, 1H), 0.81 (d, J = 14.3 Hz, 1H), 0.66 (t, J = 7.1 Hz, 3H). **^{13}C NMR: (75 MHz, $CDCl_3$) δ =** 143.36, 141.05, 135.14, 128.95, 128.06, 127.98, 127.82, 127.01, 125.95, 124.60, 79.95, 73.94, 66.75, 53.62, 53.36, 32.04, 7.56. **HRMS (DART, $[M+1]^+$) m/z** calcd. For $C_{26}H_{28}N_3O$: 398.2232; found: 398.2223.

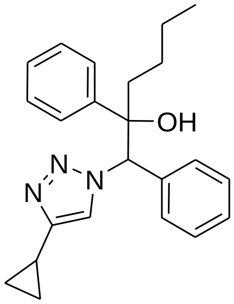

1-(4-Cyclopropyl-[1,2,3]triazol-1-yl)-1,2-diphenyl-hexan-2-ol (17)

Yield: 218 mg (60.3 %), m.p. : 169-169.5 °C**. IR (ATR, cm^{-1}):** 3177, 3092, 2956, 2869, 1743, 1564, 1449, 1214, 1054, 732, 699. **^{1}H NMR: (300 MHz, $CDCl_3$) δ** 7.42 (s, 1H), 7.23 – 6.91 (m, 10H), 5.57 (s, 1H), 4.00 (s, 1H), 2.00 – 1.81 (m, 2H), 1.42 (ddd, *J* = 13.8, 11.7, 4.1 Hz, 1H), 1.28 – 0.96 (m, 3H), 0.94 – 0.73 (m, 5H), 0.66 (t, *J* = 7.2 Hz, 3H). **^{13}C NMR: (75 MHz, $CDCl_3$)** δ = 149.66, 141.59, 135.29, 128.87, 128.01, 127.85, 127.70, 126.91, 125.86, 121.78, 79.72, 73.92, 39.12, 25.48, 22.83, 13.95, 7.90, 6.70. **HRMS (DART, $[M+1]^+$)** m/z calcd. For $C_{23}H_{28}N_3O$: 362.2232; found: 362.2222.

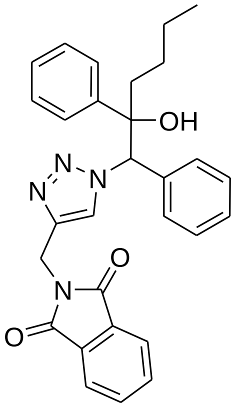

2-[1-(2-Hydroxy-1,2-diphenyl-hexyl)-[1,2,3]triazol-4-ylmethyl]-isoindole-1,3-dione (18)

Yield: 341 mg (71.0 %), m.p. : 187.8-188.5 °C. **IR (ATR, cm^{-1})**: 3462, 3398, 3292, 3147, 3027, 2949, 2864, 1769, 1705, 1428, 1398, 1093, 935, 699. **^{1}H NMR: (300 MHz, $CDCl_3$) δ**

7.82 (s, 1H), 7.82 – 7.70 (m, 2H), 7.70 – 7.56 (m, 2H), 7.21 – 6.90 (m, 10H), 5.62 (s, 1H), 4.94 (s, 2H), 1.92 (ddd, *J* = 13.7, 11.6, 4.5 Hz, 1H), 1.35 (ddd, *J* = 13.7, 11.7, 4.1 Hz, 1H), 1.24 – 1.04 (m, 1H), 1.09 – 0.91 (m, 1H), 1.02 (s, 1H), 0.87 – 0.69 (m, 1H), 0.76 (s, 1H), 0.61 (t, *J* = 7.2 Hz, 3H). **^{13}C NMR: (75 MHz, $CDCl_3$) δ** = 167.67, 167.00, 142.27, 141.43, 134.91, 134.26, 134.12, 132.05, 128.95, 128.02, 127.95, 127.75, 126.94, 125.81, 124.81, 123.59, 123.48, 79.66, 74.20, 39.00, 33.02, 25.42, 22.75, 13.89. **HRMS (DART, $[M+1]^+$)** m/z calcd. For $C_{29}H_{29}N_4O_3$: 481.2240; found: 481.2229.

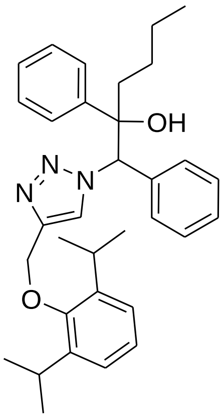

1-[4-(2,6-Diisopropy-phenoxymethyl)-[1,2,3]triazol-1-yl]-1,2-diphenyl-hexan-2-ol (19)

Yield: 376 mg (73.4 %), m.p. : 151-151.5 °C. **IR (ATR, cm^{-1}):** 3380, 3167, 3027, 2962, 2868, 1741, 1449, 1172, 977, 757, 698. **^{1}H NMR: (300 MHz, $CDCl_3$) δ** .94 (s, 1H), 7.34 – 6.99 (m, 13H), 5.79 (s, 1H), 4.98 (s, 2H), 3.82 (s, 1H), 3.38 (p, *J* = 6.9 Hz, 2H), 2.07 (ddd, *J* = 13.8, 11.6, 4.5 Hz, 1H), 1.55 (ddd, *J* = 13.8, 11.7, 4.1 Hz, 1H), 1.32 – 1.09 (m, 15H), 1.04 – 0.82 (m, 1H), 0.75 (t, *J* = 7.2 Hz, 3H). **^{13}C NMR: (75 MHz, $CDCl_3$) δ =** 152.85, 144.38, 141.89, 141.49, 135.09, 128.95, 128.10, 128.02, 127.83, 127.03, 125.84, 125.22, 125.07, 124.30, 124.18, 124.11, 79.76, 74.20, 68.15, 39.17, 26.64, 25.50, 24.10, 22.84, 13.96. **HRMS (DART, $[M+1]^+$)** m/z calcd. For $C_{33}H_{42}N_3O_2$: 512.3277; found: 512.3269.

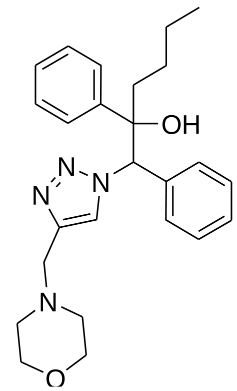

1-(4-Morpholin-4-ylmethyl-[1,2,3]triazol-1-yl)-1,2-diphenyl-hexan-2-ol (20)

Yield: 260 mg (62.0 %), m.p. : 123-123.6 °C. **IR (ATR, cm^{-1}):** 3243, 3030, 2958, 2932, 2864, 2818, 1451, 1115, 1003, 699. **^{1}H NMR: (300 MHz, $CDCl_3$) δ =** 7.74 (s, 1H), 7.23 – 6.93 (m, 10H), 5.70 (s, 1H), 3.62 (q, *J* = 7.1 Hz, 7H), 2.43 (t, *J* = 4.5 Hz, 4H), 1.96 (ddd, *J* = 13.6, 11.5, 4.5 Hz, 1H), 1.45 – 1.25 (m, 1H), 1.06 (hept, *J* = 6.9 Hz, 4H), 0.64 (t, *J* = 7.1 Hz, 4H). **^{13}C NMR: (75 MHz, $CDCl_3$) δ =** 141.52, 135.05, 128.93, 128.06, 127.97, 127.79, 127.00, 125.81, 79.68, 74.08, 66.83, 53.72, 53.43, 39.02, 25.38, 22.81, 13.87. **HRMS (DART, $[M+1]^+$)** m/z calcd. For $C_{25}H_{33}N_4O_2$: 421.2604; found: 421.2595.

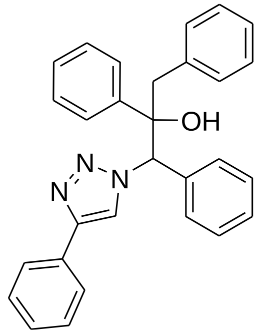

1,2,3-Triphenyl-1-(4-phenyl-[1,2,3]triazol-1-yl)-propan-2-ol (21)

Yield: 186 mg (43.1 %), m.p. 228-228.5°C. **IR (ATR, cm^{-1}):** 3457, 3444, 3061, 3029, 2929, 1948, 1880, 1742, 1449, 1361, 1156, 759. **^{1}H NMR: (300 MHz, DMSO-d_6) δ =** 8.82 (s, 1H), 8.00 – 7.91 (m, 2H), 7.56 – 7.42 (m, 4H), 7.42 – 7.30 (m, 3H), 7.19 – 6.96 (m, 9H), 6.89 (dd, *J* = 6.7, 3.0 Hz, 2H), 6.48 (s, 1H), 5.78 (s, 1H), 3.38 (d, *J* = 13.9 Hz, 1H), 2.77 (d, *J* = 13.8 Hz, 1H). **^{13}C NMR: (75 MHz, DMSO-d_6) δ =** 146.72, 142.58, 136.97, 136.61, 131.26, 131.02, 130.15, 129.33, 128.35, 128.25, 128.10, 127.73, 127.69, 126.92, 126.85, 126.32, 125.81, 122.45, 79.62, 79.53, 72.24, 46.25, 40.84, 40.56, 40.28, 40.00, 39.72, 39.45, 39.17. **HRMS (DART, $[M+1]^+$)** m/z calcd. For $C_{29}H_{26}N_3O$: 432.2076; found: 432.2071.

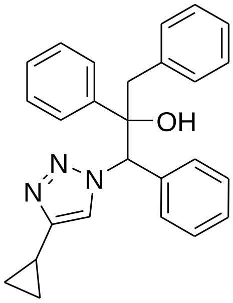

1-(4-Cyclopropyl-[1,2,3]triazol-1-yl)-1,2,3-triphenyl-propan-2-ol (22)

Yield: 233 mg (59.0 %), m.p. 168.2-169.0 °C. **IR (ATR, cm^{-1}):** 3433, 3146, 3060, 3028, 3000, 2933, 1743, 1450, 1362, 1161, 1046, 727, 696. **^{1}H NMR: (300 MHz, $CDCl_3$) δ =** 7.74 (s, 1H), 7.20 – 7.11 (m, 4H), 7.02 (dq, *J* = 24.4, 6.3 Hz, 9H), 6.67 – 6.59 (m, 2H), 6.01 (s, 1H), 3.47 (s, 1H), 3.31 (d, *J* = 13.2 Hz, 1H), 2.69 (d, *J* = 13.4 Hz, 1H), 0.93 – 0.80 (m, 4H). **^{13}C NMR: (75 MHz, $CDCl_3$) δ =** 148.95, 140.34, 134.20, 133.60, 129.60, 129.43, 128.32, 128.06, 126.84, 126.75, 126.73, 126.11, 125.89, 125.65, 125.35, 124.92, 120.12, 78.29, 71.29, 44.62, 6.76, 6.68, 5.67. **HRMS (DART, $[M+1]^+$)** m/z calcd. For $C_{26}H_{26}N_3O$: 396.2076; found: 396.2065.

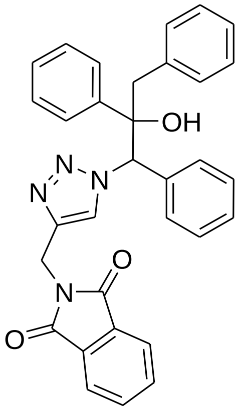

2-[1-(2-Hydroxy-1,2,3-triphenyl-propyl)-[1,2,3]triazol-4-ylmethyl]-isoindole-1,3-dione (23)

Yield: 244 mg (44.0 %), m.p. 224.9-225.5 °C. **IR (ATR, cm^{-1}):** 3435, 3149, 3010, 2947, 2862, 1715, 1612, 1515, 1370, 1255, 1096, 765. **^{1}H NMR: (300 MHz, $CDCl_3$) δ** = 8.19 (s, 1H), 7.88 (dd, *J* = 5.4, 3.1 Hz, 2H), 7.72 (dd, *J* = 5.5, 3.0 Hz, 2H), 7.31 – 7.01 (m, 13H), 6.68 (dt, *J* = 6.6, 1.6 Hz, 2H), 6.13 (s, 1H), 5.07 (s, 1H), 4.07 – 3.91 (m, 1H), 3.82 – 3.59 (m, 1H), 3.38 (d, *J* = 13.6 Hz, 1H), 2.67 (d, *J* = 13.6 Hz, 1H). **^{13}C NMR: (75 MHz, $CDCl_3$) δ =** 177.69, 167.69, 142.61, 141.46, 135.01, 134.49, 134.09, 132.12, 130.60, 129.29, 128.06, 128.03,

127.96, 127.94, 127.12, 126.88, 126.07, 124.42, 123.48, 79.34, 72.68, 45.64, 33.16, 29.69, 29.55, 29.52, 27.79, 23.92, 23.84, 23.81, 23.48, 22.18, 6.15. **HRMS (DART, [M+1]$^+$)** m/z calcd. For $C_{32}H_{27}N_4O_3$: 515.2083; found: 515.2069.

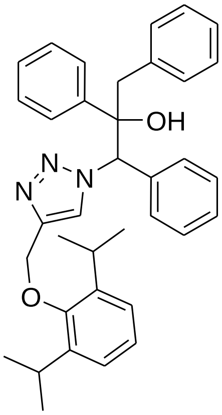

1-[4-(2,6-Diisopropyl-phenoxymethyl)-[1,2,3]triazol-1-yl]-1,2,3-triphenyl-propan-2-ol (24)

Yield: 225 mg (41.3 %), m.p. 167.1-167.6 °C. **IR (ATR, cm^{-1}):** 3250, 3173, 3061, 3030, 2962, 2926, 2867, 1494, 1447, 1357, 1253, 1180, 1102, 1052, 968, 699. **^{1}H NMR: (300 MHz, $CDCl_3$) δ =** 8.22 (s, 1H), 7.36 – 7.00 (m, 16H), 6.75 – 6.67 (m, 2H), 6.21 (s, 1H), 5.27 (s, 1H), 5.02 (s, 2H), 3.53 – 3.34 (m, 3H), 3.28 – 3.22 (m, 1H), 1.31 – 1.20 (m, 13H). **^{13}C NMR: (75 MHz, $CDCl_3$) δ =** 153.02, 144.88, 141.91, 141.49, 135.19, 134.49, 130.62, 129.33, 128.16, 128.11, 128.04, 127.21, 127.02, 126.10, 124.18, 123.85, 79.41, 72.70, 68.31, 45.81, 26.70, 24.12, 24.08. **HRMS (DART, [M+1]$^+$)** m/z calcd. For $C_{36}H_{40}N_3O_2$: 546.3121; found: 546.3112.

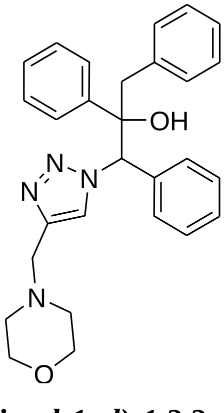

1-(4-Morpholin-4-ylmethyl-[1,2,3]triazol-1-yl)-1,2,3-triphenyl-propan-2-ol (25)

Yield: 218 mg (48.0 %), m.p. 160.2–161.8 °C. **IR (ATR, cm^{-1}):** 3420, 3338, 3121, 3036, 3029, 2957, 2826, 1741, 1659, 1495, 1450, 1217, 1113, 698. **^{1}H NMR: (300 MHz, $CDCl_3$) δ =** 8.06 (s, 1H), 7.35 – 7.00 (m, 13H), 6.74 – 6.64 (m, 2H), 6.17 (s, 1H), 3.77 – 3.67 (m, 6H), 3.43 (d, *J* = 13.5 Hz, 1H), 3.28 (s, 1H), 2.68 (d, *J* = 13.6 Hz, 1H), 2.60 – 2.51 (m, 4H). **^{13}C NMR: (75 MHz, $CDCl_3$) δ** = 141.09, 134.80, 134.13, 130.20, 128.93, 127.76, 127.69, 127.64, 127.62, 126.80, 126.62, 125.69, 123.86, 79.02, 72.19, 66.43, 53.40, 53.02, 45.34, 31.22, 22.29, 13.76. **HRMS (DART, $[M+1]^+$)** m/z calcd. For $C_{28}H_{31}N_4O_2$: 455.2447; found: 455.2439.

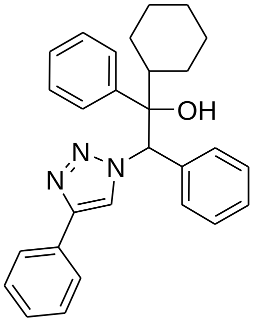

1-Cyclohexyl-1,2-diphenyl-2-(4-phenyl-[1,2,3]triazol-1-yl)-ethanol (26)

Yield: 260 mg (61.4 %), m.p. 237–237.5 °C. **IR (ATR, cm^{-1}):** 3514, 3404, 3149, 3028, 2933, 2852, 1741, 1718, 1445, 1355, 1031, 759, 692. **^{1}H NMR: (300 MHz, DMSO-d_6) δ =** 8.42 (s, 1H), 7.74 – 7.63 (m, 2H), 7.52 – 7.42 (m, 2H), 7.34 – 7.27 (m, 2H), 7.27 – 7.16 (m, 2H), 7.16 – 7.04 (m, 1H), 6.98 (t, *J* = 7.5 Hz, 2H), 6.94 – 6.78 (m, 4H), 6.59 (s, 1H), 5.39 (s, 1H), 2.19 – 0.45 (m, 11H). **^{13}C NMR: (75 MHz, DMSO-d_6) δ =** 195.29, 146.42, 141.50, 137.93, 131.30, 130.72, 130.06, 129.97, 129.28, 128.27, 127.92, 127.44, 126.73, 125.72, 122.02, 80.98, 67.68, 46.52, 28.39, 26.94, 26.60, 26.34. **HRMS (DART, $[M+1]^+$)** m/z calcd. For $C_{28}H_{30}N_3O$: 424.2389; found: 424.2379.

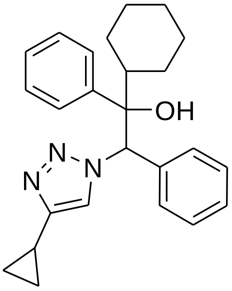

1-Cyclohexyl-2-(4-cyclopropyl-[1,2,3]triazol-1-yl)-1,2-diphenyl-ethanol (27)

Yield: 166 mg (43.0 %), m.p. 190.2–192 °C. **IR (ATR, cm^{-1}):** 3396, 3087, 3031, 2932, 2854, 1740, 1557, 1495, 1449, 1330, 1233, 1040, 701. **^{1}H NMR: (300 MHz, $CDCl_3$) δ =** 7.54 (s, 1H), 7.44 – 7.33 (m, 2H), 7.33 – 7.17 (m, 4H), 7.21 – 7.10 (m, 1H), 7.15 – 6.99 (m, 3H), 6.34 (s, 1H), 3.79 (s, 1H), 1.95 (ddd, *J* = 13.4, 6.7, 4.2 Hz, 2H), 1.89 – 1.67 (m, 2H), 1.65 (s, 1H), 1.55 (dd, *J* = 27.8, 12.3 Hz, 2H), 1.39 (tt, *J* = 11.7, 2.7 Hz, 1H), 1.29 – 0.76 (m, 7H), 0.47 – 0.28 (m, 1H). **^{13}C NMR: (75 MHz, $CDCl_3$) δ =** 148.89, 139.13, 135.15, 128.69, 127.16, 127.06, 126.65, 126.10, 126.02, 125.84, 120.64, 80.43, 67.82, 46.56, 28.03, 26.20, 25.82, 25.51, 25.35, 7.13, 7.09, 6.00, -0.75. **HRMS (DART, $[M+1]^+$)** m/z calcd. For $C_{25}H_{30}N_3O$: 388.2389; found: 388.2379.

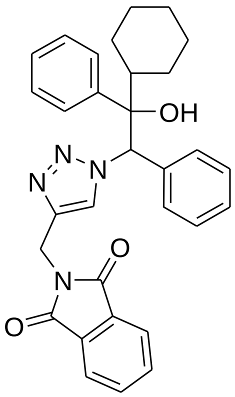

2-[1-(2-Cyclohexyl-2-hydroxy-1,2-diphenyl-ethyl)-[1,2,3]triazol-4-ylmethyl]-isoindole-1,3-dione (28)

Yield: 324 mg (64.0 %), m.p. 218–218.3 °C. **IR (ATR, cm^{-1}):** 3555, 3464, 3292, 2930, 2854, 1770, 1708, 1608, 1393, 1329, 1097, 939, 698. **^{1}H NMR: (300 MHz, $CDCl_3$) δ =** 7.96 (s, 1H), 7.84 (dd, *J* = 5.5, 3.0 Hz, 2H), 7.70 (dd, *J* = 5.5, 3.1 Hz, 2H), 7.46 – 7.32 (m, 2H), 7.32 – 7.18 (m, 4H), 7.18 – 7.10 (m, 1H), 7.06 (p, *J* = 3.6 Hz, 3H), 6.40 (s, 1H), 5.08 – 4.95 (m, 2H), 2.01 – 1.89 (m, 1H), 1.81 (s, 1H), 1.65 – 1.52 (m, 3H), 1.45 (d, *J* = 11.7 Hz, 1H), 1.30 (tt, *J* = 11.8, 2.7 Hz, 1H), 1.19 – 0.76 (m, 4H), 0.36 (qd, *J* = 12.8, 3.4 Hz, 1H). **^{13}C NMR: (75 MHz, $CDCl_3$) δ =** 191.68, 167.64, 142.10, 139.76, 135.48, 134.08, 132.08, 129.54, 129.06, 127.99, 127.43, 126.91, 126.58, 124.59, 123.46, 81.17, 68.94, 47.17, 33.09, 28.70, 26.93, 26.45, 26.19, 26.07. **HRMS (DART, $[M+1]^+$)** m/z calcd. For $C_{31}H_{31}N_4O_3$: 507.2396; found: 507.2390.

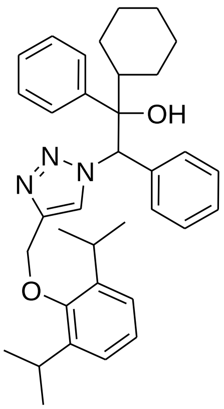

1-Cyclohexyl-2-[4-(2,6-diisopropyl-phenoxymethyl)-[1,2,3]triazol-1-yl]-1,2-diphenyl-ethanol (29)

Yield: 163 mg (30.5 %), m.p. 144-144.3 °C. **IR (ATR, cm^{-1}):** 3260, 3064, 3029, 2959, 2928, 2864, 1741, 1600, 1448, 1317, 1177, 996, 703. **^{1}H NMR: (300 MHz, $CDCl_3$) δ =** 8.00 (s, 1H), 7.44 – 7.27 (m, 4H), 7.31 – 7.14 (m, 3H), 7.19 – 7.05 (m, 3H), 6.50 (s, 1H), 4.99 (d, *J* = 2.6 Hz, 2H), 3.53 (s, 1H), 3.37 (hept, *J* = 6.9 Hz, 2H), 2.05 (d, *J* = 11.5 Hz, 1H), 1.91 – 1.80 (m, 1H), 1.76 – 1.52 (m, 2H), 1.50 (s, 1H), 1.47 – 1.16 (m, 15H), 1.19 – 0.92 (m, 2H), 0.97 – 0.80 (m, 3H), 0.42 (qd, *J* = 12.7, 3.4 Hz, 1H). **^{13}C NMR: (75 MHz, $CDCl_3$) δ =** 153.01, 144.39, 141.89, 139.79, 135.70, 129.56, 128.09, 128.01, 127.50, 126.98, 126.62, 125.02, 124.03, 81.22, 68.83, 68.24, 47.20, 28.74, 26.95, 26.68, 26.53, 26.26, 26.09, 25.30, 24.07, 24.04, 22.67. **HRMS (DART, [M+1]$^{+}$)** m/z calcd. For $C_{35}H_{44}N_3O_2$: 538.3434; found: 538.3426.

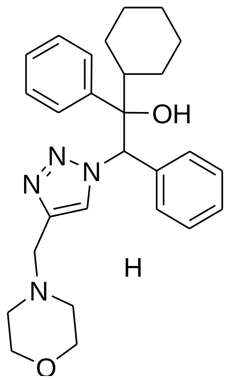

1-Cyclohexyl-2-(4-morpholin-4-ylmethyl-[1,2,3]triazol-1-yl)-1,2-diphenyl-ethanol (30)

Yield: 215 mg (48.3 %), m.p. 169.5–170.3 °C. **IR (ATR, cm^{-1}):** 3480, 3109, 3033, 2930, 2853, 2813, 1673, 1496, 1449, 1116, 1052, 1002, 861, 702. **^{1}H NMR: (300 MHz, $CDCl_3$) δ** = 7.92 (s, 1H), 7.39 – 7.05 (m, 10H), 6.45 (s, 1H), 3.72 (d, *J* = 5.5 Hz, 7H), 2.56 (d, *J* = 6.0

Hz, 4H), 1.97 (d, J = 11.7 Hz, 1H), 1.85 (s, 1H), 1.72 – 1.55 (m, 2H), 1.54 – 1.40 (m, 2H), 1.39 – 0.80 (m, 4H), 0.39 (qd, J = 12.6, 3.3 Hz, 1H). **^{13}C NMR: (75 MHz, $CDCl_3$) δ =** 200.81, 139.87, 135.63, 129.48, 128.03, 127.96, 127.50, 126.96, 126.58, 124.65, 81.19, 68.86, 66.64, 60.09, 53.31, 47.28, 28.65, 26.94, 26.50, 26.36, 14.13, 12.03. **HRMS (DART, $[M+1]^+$)** m/z calcd. For $C_{27}H_{35}N_4O_2$: 447.2760; found: 447.2850.

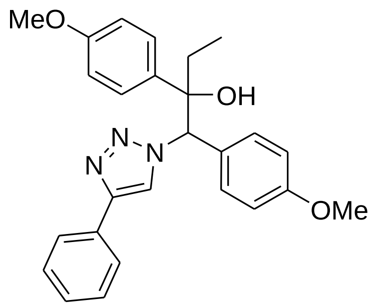

1,2-Bis-(4-methoxyphenyl)-1-(4-phenyl-[1,2,3]triazol-1-yl)-butan-2-ol (31)

Yield: 262 mg (61.0 %), m.p. 171-171.6 °C. **IR (ATR, cm^{-1}):** 3451, 3159, 2966, 2935, 2833, 1741, 1611, 1512, 1253, 1176, 1031, 797. **^{1}H NMR: (300 MHz, DMSO-d_6) δ =** 8.68 (s, 1H), 7.96 – 7.88 (m, 2H), 7.53 – 7.40 (m, 4H), 7.40 – 7.27 (m, 3H), 6.82 – 6.74 (m, 2H), 6.74 – 6.65 (m, 2H), 6.17 (s, 1H), 5.55 (s, 1H), 3.65 (d, J = 14.6 Hz, 6H), 1.95 (dq, J = 14.5, 7.2 Hz, 1H), 1.33 (dt, J = 14.1, 7.1 Hz, 1H), 0.55 (t, J = 7.2 Hz, 3H). **^{13}C NMR: (75 MHz, DMSO-d_6) δ =** 158.87, 157.92, 146.52, 131.43, 131.33, 129.55, 129.30, 128.25, 127.76, 125.70, 121.82, 113.56, 113.37, 79.06, 72.01, 55.30, 55.26, 33.05, 8.13. **HRMS (DART, $[M+1]^+$)** m/z calcd. For $C_{26}H_{28}N_3O_3$: 430.2131; found: 430.2123.

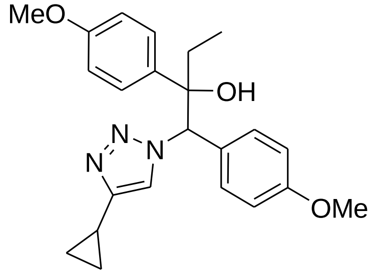

1-(4-Cyclopropyl-[1,2,3]triazol-1-yl)-1,2-bis-(4-methoxyphenyl)-butan-2-ol (32)

Yield: 259 mg (66.0 %), m.p. 136.5–138 °C. **IR (ATR, cm^{-1}):** 3211, 3012, 2957, 2837, 1741, 1610, 1511, 1462, 1245, 1177, 1029, 797. **^{1}H NMR: (300 MHz, $CDCl_3$) δ =** 7.40 (s, 1H),

7.13 – 7.02 (m, 2H), 7.02 – 6.91 (m, 2H), 6.73 – 6.63 (m, 2H), 6.59 – 6.48 (m, 2H), 5.52 (s, 1H), 3.81 (s, 1H), 3.68 (s, 3H), 3.61 (s, 3H), 1.97 – 1.84 (m, 1H), 1.84 (dd, J = 8.1, 5.0 Hz, 1H), 1.40 (dq, J = 14.3, 7.2 Hz, 1H), 0.93 – 0.72 (m, 4H), 0.59 (t, J = 7.3 Hz, 3H). **^{13}C NMR: (75 MHz, $CDCl_3$) δ =** 159.03, 158.33, 149.58, 133.31, 130.14, 127.70, 127.19, 121.59, 113.33, 113.12, 79.77, 77.49, 77.27, 77.07, 76.64, 73.33, 55.14, 55.09, 32.08, 7.87, 7.85, 7.58, 6.67. **HRMS (DART, $[M+1]^+$)** m/z calcd. For $C_{23}H_{28}N_3O_3$: 394.2131; found: 394.2121.

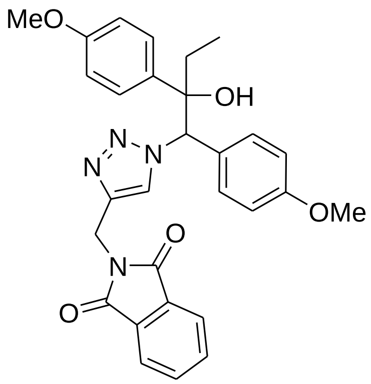

2-{1-[2-Hydroxy-1,2-bis-(4-methoxy-phenyl)-butyl]-[1,2,3]triazol-4-ylmethyl}-isoindole-1,3-dione (33)

Yield: 230 mg (45.0 %), m.p. 148.2–149 °C. **IR (ATR, cm^{-1}):** 3522, 3465, 3396, 3138, 2970, 2936, 1714, 1613, 1514, 1256, 1032, 716. **^{1}H NMR: (300 MHz, $CDCl_3$) δ** 7.83 – 7.72 (m, 3H), 7.69 – 7.57 (m, 2H), 7.10 – 7.02 (m, 2H), 7.01 – 6.94 (m, 2H), 6.67 (d, J = 8.6 Hz, 2H), 6.52 (d, J = 8.5 Hz, 2H), 5.56 (s, 1H), 4.92 (s, 2H), 3.90 – 3.29 (m, 7H), 1.87 (dq, J = 14.6, 7.3 Hz, 1H), 1.35 (dq, J = 14.3, 7.2 Hz, 1H), 0.56 (t, J = 7.2 Hz, 3H). **^{13}C NMR: (75 MHz, $CDCl_3$)** δ = 167.67, 159.09, 158.35, 134.11, 133.13, 132.05, 130.23, 127.35, 127.15, 124.56, 123.48, 113.35, 113.17, 79.71, 73.65, 55.13, 55.08, 33.01, 32.03, 7.53. **HRMS (DART, $[M+1]^+$)** m/z calcd. For $C_{23}H_{28}N_4O_5$: 513.2138; found: 513.2132.

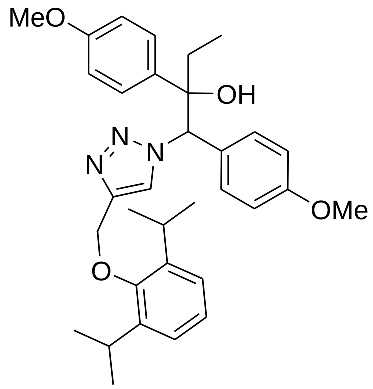

1-[4-(2,6-Diisopropylphenoxymethyl)-[1,2,3]triazol-1-yl]-1,2-bis-(4-methoxy-phenyl)-butan-2-ol (34)

Yield: 298 mg (55.0 %), m.p. 128.5–129 °C. **IR (ATR, cm^{-1}):** 3474, 3149, 3013, 2962, 2932, 2838, 1612, 1513, 1462, 1250, 1178, 1036, 803. **^{1}H NMR: (300 MHz, $CDCl_3$) δ** 7.83 (s, 1H), 7.15 – 7.06 (m, 2H), 7.06 – 6.97 (m, 5H), 6.75 – 6.65 (m, 2H), 6.62 – 6.51 (m, 2H), 5.66 (s, 1H), 4.89 (s, 2H), 3.68 (s, 3H), 3.62 (s, 3H), 3.30 (hept, *J* = 6.8 Hz, 2H), 1.94 (dq, *J* = 14.6, 7.4 Hz, 1H), 1.45 (dq, *J* = 14.3, 7.2 Hz, 1H), 1.14 (d, *J* = 6.9 Hz, 12H), 0.61 (t, *J* = 7.2 Hz, 3H). **^{13}C NMR: (75 MHz, $CDCl_3$) δ =** 159.17, 158.43, 152.83, 144.23, 141.89, 133.18, 130.25, 127.18, 125.06, 124.17, 124.10, 113.43, 113.24, 79.81, 73.66, 68.08, 55.16, 55.13, 32.17, 26.63, 24.09, 7.59. **HRMS (DART, [M+1]$^+$)** m/z calcd. For $C_{33}H_{42}N_3O_4$: 544.3175; found: 544.3168.

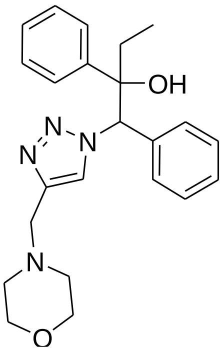

1-(4-Morpholin-4-ylmethyl-[1,2,3]triazol-1-yl)-1,2-diphenyl-butan-2-ol (35)

Yield: 271 mg (60.0 %), m.p. 198.5-199 °C. **IR (ATR, cm^{-1}):** 3211, 3012, 2957, 2837, 1741, 1610, 1511, 1462, 1245, 1177, 1029, 797. **^{1}H NMR: (300 MHz, $CDCl_3$) δ** 7.75 (s, 1H), 7.21 – 7.05 (m, 4H), 6.82 – 6.73 (m, 2H), 6.69 – 6.60 (m, 2H), 5.69 (s, 1H), 3.80 – 3.68 (m, 13H), 2.76 (s, 1H), 2.53 (t, *J* = 4.6 Hz, 4H), 2.00 (td, J = 13.3, 6.0 Hz, 1H), 1.46 (dq, *J* = 14.3, 7.2

Hz, 1H), 0.68 (t, *J* = 7.3 Hz, 3H). **^{13}C NMR: (75 MHz, $CDCl_3$) δ =** 159.11, 158.38, 143.22, 133.22, 130.21, 127.17, 124.44, 113.37, 113.20, 79.74, 73.49, 66.74, 55.14, 55.10, 53.65, 53.34, 32.00, 7.57. **HRMS (DART, $[M+1]^+$)** m/z calcd. For $C_{25}H_{35}N_4O_4$: 453.2502; found: 453.2492.

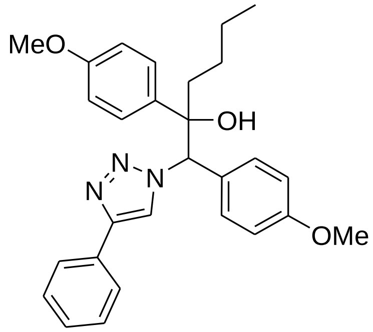

1,2-Bis-(4-methoxyphenyl)-1-(4-phenyl-[1,2,3]triazol-1-yl)-hexan-2-ol (36)

Yield: 228 mg (50.0 %), m.p. 163.8-163.5 °C. IR (ATR, cm^{-1}): 3196, 2950, 2930, 2834, 1610, 1512, 1457, 1250, 1174, 1033, 760. **^{1}H NMR: (300 MHz, $CDCl_3$) δ** 7.97 (s, 1H), 7.77 (d, *J* = 7.5 Hz, 2H), 7.34 (t, *J* = 7.5 Hz, 2H), 7.29 – 7.20 (m, 1H), 7.11 (d, *J* = 8.3 Hz, 2H), 7.02 (d, *J* = 8.3 Hz, 2H), 6.70 (d, *J* = 8.3 Hz, 2H), 6.55 (d, *J* = 8.3 Hz, 2H), 5.67 (s, 1H), 3.68 (s, 3H), 3.60 (s, 3H), 1.92 (t, *J* = 12.8 Hz, 1H), 1.43 (td, *J* = 12.7, 3.9 Hz, 1H), 1.20 – 0.99 (m, 4H), 0.82 (d, *J* = 13.7 Hz, 1H), 0.65 (t, *J* = 7.1 Hz, 3H). **^{13}C NMR: (75 MHz, $CDCl_3$) δ =** 159.13, 158.38, 147.22, 133.76, 132.51, 130.37, 130.21, 128.89, 128.30, 127.53, 127.07, 125.75, 121.32**, 114.22, 113.68, 113.42, 113.26, 79.57, 73.64, 55.16, 55.11, 39.27, 25.52, 22.81, 13.97. HRMS (DART, $[M+1]^+$)** m/z calcd. For $C_{28}H_{32}N_3O_3$: 458.2444; found: 458.2436.

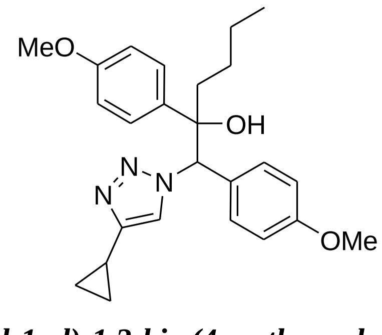

1-(4-Cyclopropyl-[1,2,3]triazol-1-yl)-1,2-bis-(4-methoxyphenyl)-hexan-2-ol (37)

Yield: 160 mg (38.2 %), m.p. 176-176.7 °C. **IR (ATR, cm^{-1}):** 3211, 3170, 2955, 2931, 2838, 1741, 1610, 1511, 1447, 1249, 1177, 1029, 801. **^{1}H NMR: (300 MHz, $CDCl_3$) δ** 7.38 (s, 1H), 7.07 (dd, *J* = 9.0, 2.4 Hz, 2H), 6.98 – 6.89 (m, 2H), 6.73 – 6.63 (m, 2H), 6.58 – 6.49 (m, 2H), 5.50 (s, 1H), 3.89 – 3.82 (m, 1H), 3.68 (d, *J* = 1.0 Hz, 3H), 3.61 (d, *J* = 0.9 Hz, 3H), 1.94 – 1.79 (m, 1H), 1.36 (td, *J* = 12.6, 3.9 Hz, 1H), 1.20 – 0.97 (m, 3H), 0.94 – 0.73 (m, 5H), 0.66 (t, *J* = 7.1 Hz, 3H)**.** **^{13}C NMR: (75 MHz, $CDCl_3$) δ =** 159.02, 158.29, 149.59, 133.81, 130.11, 129.90, 127.70, 127.05, 121.56, 114.05, 113.32, 113.10, 79.50, 77.48, 77.06, 76.63, 73.44, 55.25, 55.08, 39.13, 25.51, 22.83, 13.96, 7.86, 6.69. **HRMS (DART, $[M+1]^+$)** m/z calcd. For $C_{25}H_{32}N_3O_3$: 422.2444; found: 422.2435.

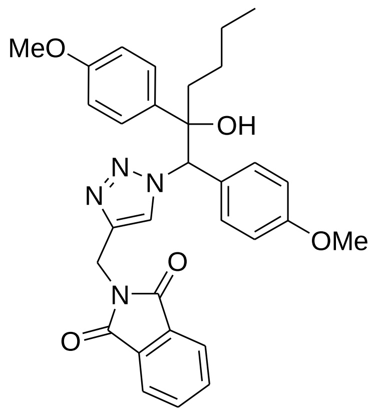

2-{1-[2-Hydroxy-1,2-bis-(4-methoxyphenyl)-hexyl]-[1,2,3]triazol-4-ylmethyl}-isoindole-1,3-dione (38)

Yield: 226 mg (42.0 %), m.p. 100-101.5 °C. **IR (ATR, cm^{-1}):** 3435, 3149, 3010, 2947, 2862, 1715, 1612, 1515, 1370, 1255, 1096, 765. **^{1}H NMR: (300 MHz, $CDCl_3$) δ** 7.87 – 7.74 (m, 1H), 7.75 (q, *J* = 3.2 Hz, 2H), 7.70 – 7.56 (m, 2H), 7.10 – 7.00 (m, 2H), 6.94 (d, *J* = 8.5 Hz, 2H), 6.72 – 6.62 (m, 2H), 6.51 (d, *J* = 8.5 Hz, 2H), 5.54 (s, 1H), 4.93 (s, 2H), 3.67 (s, 3H), 3.59 (s, 3H), 1.93 – 1.72 (m, 1H), 1.29 (td, *J* = 12.6, 3.8 Hz, 1H), 1.21 – 0.93 (m, 4H), 0.81 (s, 1H), 0.62 (t, *J* = 7.1 Hz, 3H). **^{13}C NMR: (75 MHz, $CDCl_3$) δ =** 190.17, 167.65, 159.07, 158.32, 134.09, 133.63, 132.06, 130.20, 127.30, 127.02, 125.48, 123.47, 113.34, 113.15, 79.45, 73.76, 55.08, 39.04, 33.04, 25.46, 22.76, 13.91. **HRMS (DART, $[M+1]^+$)** m/z calcd. For $C_{31}H_{33}N_4O_5$: 541.2451; found: 541.2443.

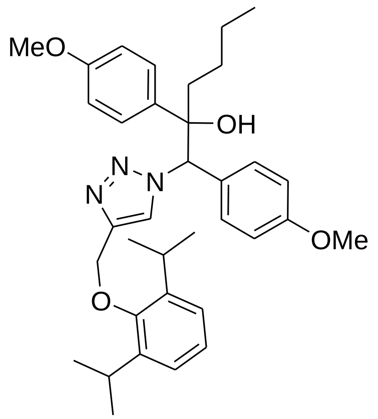

1-[4-(2,6-Diisopropylphenoxymethyl)-[1,2,3]triazol-1-yl]-1,2-bis-(4-methoxyphenyl)-hexan-2-ol (39)

Yield: 226 mg (46.0 %), m.p. 120.8-121.5 °C. **IR (ATR, cm^{-1}):** 3466, 3145, 3013, 2962, 2939, 1741, 1612, 1513, 1459, 1250, 1180, 1036, 806. **^{1}H NMR: (300 MHz, $CDCl_3$) δ** 8.19 – 7.67 (m, 1H), 7.23 – 7.05 (m, 7H), 6.80 (d, *J* = 8.0 Hz, 2H), 6.67 (d, *J* = 7.6 Hz, 2H), 5.96 – 5.63 (m, 1H), 5.18 – 4.69 (m, 2H), 3.85 – 3.65 (m, 7H), 3.42 (s, 2H), 2.05 (d, *J* = 12.2 Hz, 1H), 1.26 (d, *J* = 4.3 Hz, 16H), 0.95 (s, 1H), 0.79 (t, *J* = 6.8 Hz, 3H). **^{13}C NMR: (75 MHz, $CDCl_3$) δ =** 159.14, 158.35, 152.81, 141.89, 133.71, 130.64, 127.49, 126.57, 125.49, 124.59, 123.69, 114.00, 113.53, 112.79, 79.48, 55.10, 26.31, 24.21, 24.00, 22.85. **HRMS (DART, [M+1]$^{+}$)** m/z calcd. For $C_{35}H_{46}N_3O_4$: 572.3488; found: 572.3483.

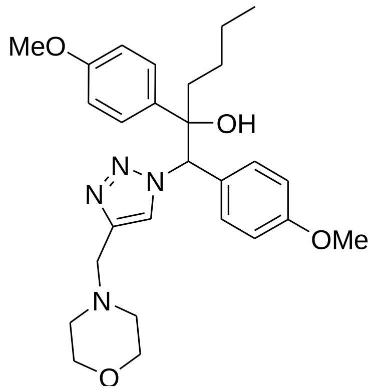

1,2-Bis-(4-methoxyphenyl)-1-(4-morpholin-4-ylmethyl-[1,2,3]triazol-1-yl)-hexan-2-ol (40)

Yield: 197 mg (41.0 %), m.p. 110.8-111.5 °C. **IR (ATR, cm^{-1}):** 3258, 3004, 2951, 2863, 1741, 1610, 1512, 1243, 1115, 1034, 800. **^{1}H NMR: (300 MHz, $CDCl_3$) δ** 7.75 (s, 1H), 7.16 (dd, *J* = 9.3, 2.5 Hz, 2H), 7.07 (dd, *J* = 9.3, 2.6 Hz, 2H), 6.82 – 6.72 (m, 2H), 6.72 – 6.58 (m,

2H), 5.67 (s, 1H), 3.89 – 3.68 (m, 13H), 2.57 – 2.48 (m, 4H), 1.97 (ddd, *J* = 13.7, 11.4, 4.3 Hz, 1H), 1.46 – 1.30 (m, 1H), 1.17 (m, 2H), 0.99 – 0.83 (m, 1H), 0.74 (t, *J* = 7.1 Hz, 3H). **^{13}C NMR: (75 MHz, $CDCl_3$) δ =** 190.43, 159.10, 158.36, 143.35, 133.70, 130.19, 127.42, 127.03, 124.40, 113.36, 113.18, 79.48, 73.60, 66.77, 55.36, 53.38, 39.02, 25.42, 22.82, 13.89. **HRMS (DART, $[M+1]^+$)** m/z calcd. For $C_{27}H_{37}N_4O_4$: 481.2815; found: 481.2806.

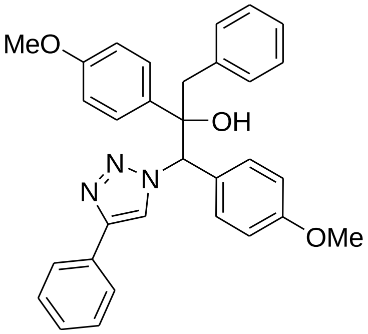

1,2-Bis-(4-methoxyphenyl)-3-phenyl-1-(4-phenyl-[1,2,3]triazol-1-yl)-propan-2-ol (41)

Yield: 227 mg (46.3 %), m.p. 183-183.7 °C. **IR (ATR, cm^{-1}):** 3457, 3144, 3061, 3029, 2929, 1880, 1742, 1493, 1449, 1361, 1329, 1156, 759, 693. **^{1}H NMR: (300 MHz, DMSO-d_6) δ =** 8.75 (s, 1H), 7.99 – 7.89 (m, 2H), 7.52 – 7.41 (m, 4H), 7.40 – 7.30 (m, 1H), 7.30 – 7.21 (m, 2H), 7.03 (dd, *J* = 5.2, 1.9 Hz, 3H), 6.89 (dt, *J* = 6.2, 2.2 Hz, 2H), 6.76 – 6.62 (m, 4H), 6.37 (s, 1H), 5.65 (s, 1H), 3.63 (d, *J* = 2.7 Hz, 6H), 3.31 (d, *J* = 13.8 Hz, 1H), 2.70 (d, *J* = 13.8 Hz, 1H). **^{13}C NMR: (75 MHz, DMSO-d_6) δ =** 156.45, 155.43, 144.22, 134.34, 132.21, 128.96, 128.84, 128.74, 128.61, 126.88, 126.39, 125.88, 125.66, 125.29, 123.85, 123.33, 111.18, 110.54, 76.96, 69.26, 52.87, 52.76, 44.00, 38.35, 38.08, 37.80, 37.52, 37.24, 36.96, 36.69. **HRMS (DART, $[M+1]^+$)** m/z calcd. For $C_{31}H_{30}N_3O_3$: 492.2287; found: 492.2282.

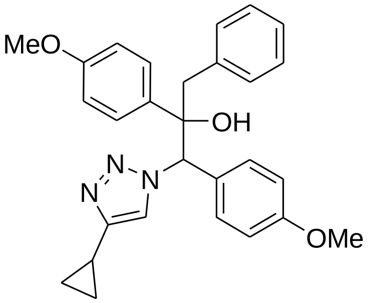

1-(4-Cyclopropyl-[1,2,3]triazol-1-yl)-1,2-bis-(4-methoxyphenyl)-3-phenyl-propan-2-ol (42)

Yield: 227 mg (50.0 %), m.p. 176.9-177.5 °C. **IR (ATR, cm^{-1}):** 3418, 3151, 3079, 3013, 2952, 2835, 1741, 1612, 1512, 1460, 1251, 1179, 1030, 806, 541. **^{1}H NMR: (300 MHz, $CDCl_3$) δ =** 7.62 (s, 1H), 7.15 – 7.07 (m, 2H), 7.07 – 6.93 (m, 5H), 6.68 – 6.58 (m, 4H), 6.58 – 6.48 (m, 2H), 5.90 (s, 1H), 3.77 – 3.63 (m, 1H), 3.64 (s, 3H), 3.58 (s, 3H), 3.31 (s, 1H), 3.25 (d, J = 13.5 Hz, 1H), 2.63 (d, J = 13.5 Hz, 1H), 1.91 (tt, J = 8.1, 5.1 Hz, 1H), 0.95 – 0.76 (m, 4H). **^{13}C NMR: (75 MHz, $CDCl_3$) δ =** 159.02, 158.35, 135.00, 133.75, 130.67, 130.48, 128.01, 127.82, 127.29, 126.76, 113.29, 113.23, 78.95, 72.02, 55.09, 55.06, 45.87, 7.94, 7.85, 6.85. **HRMS (DART, $[M+1]^+$)** m/z calcd. For $C_{28}H_{30}N_3O_3$: 456.2287; found: 456.2281.

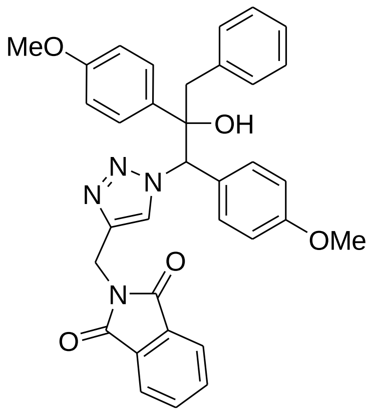

2-{1-[2-Hydroxy-1,2-bis-(4-methoxyphenyl)-3-phenyl-propyl]-[1,2,3]triazol-4-ylmethyl}-isoindole-1,3-dione (43)

Yield: 229 mg (40.0 %), m.p. 192.3°C–193.9 °C. **IR (ATR, cm^{-1}):** 3466, 3144, 3061, 3024, 2951, 2836, 1706, 1612, 1514, 1398, 1254, 1182, 1097, 1028, 712. **^{1}H NMR: (300 MHz, DMSO-d_6) δ =** 8.32 (s, 1H), 7.95 – 7.80 (m, 4H), 7.39 – 7.28 (m, 2H), 7.26 – 7.15 (m, 2H), 7.00 (dd, J = 4.8, 1.9 Hz, 3H), 6.80 (dd, J = 6.6, 3.0 Hz, 2H), 6.66 (t, J = 8.6 Hz, 4H), 6.29 (s, 1H), 5.51 (s, 1H), 4.89 (s, 2H), 3.61 (d, J = 2.3 Hz, 6H), 3.18 (d, J = 13.6 Hz, 1H), 2.56 (d, J = 13.7 Hz, 1H). **^{13}C NMR: (75 MHz, DMSO-d_6) δ =** 167.89, 158.87, 157.89, 142.39, 136.69, 135.04, 134.59, 132.07, 131.24, 131.03, 129.14, 128.05, 127.70, 126.25, 124.35, 123.70, 113.57, 112.95, 79.39, 71.45, 55.31, 55.20, 46.30, 40.79, 40.51, 40.23, 39.96, 39.68, 39.40, 39.12, 33.49, 30.89. **HRMS (DART, $[M+1]^+$)** m/z calcd. For $C_{34}H_{31}N_4O_5$: 575.2294; found: 575.2087.

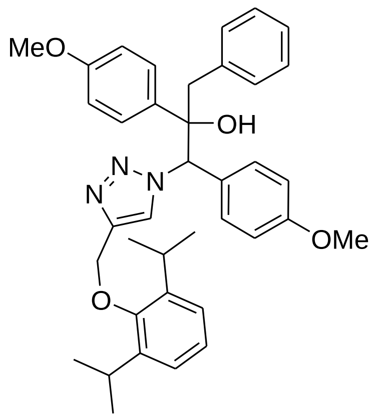

1-[4-(2,6-Diisopropylphenoxymethyl)-[1,2,3]triazol-1-yl]-1,2-bis-(4-methoxy-phenyl)-3-phenyl-propan-2-ol (44)

Yield: 229 mg (38.0 %), m.p. 148.2–148.9 °C. **IR (ATR, cm^{-1}):** 3388, 3139, 3064, 2960, 2908, 2868, 2837, 1740, 1612, 1514, 1457, 1251, 1182, 1035, 806, 542. **^{1}H NMR: (300 MHz, $CDCl_3$) δ =** 8.23 (s, 1H), 7.29 (d, J = 8.5 Hz, 2H), 7.22 – 7.07 (m, 8H), 6.80 – 6.72 (m, 4H), 6.68 (d, J = 8.2 Hz, 2H), 6.18 (s, 1H), 5.05 (s, 2H), 3.77 (s, 3H), 3.72 (s, 3H), 3.44 (t, J = 9.7 Hz, 3H), 3.16 (s, 1H), 2.77 (d, J = 13.2 Hz, 1H), 1.28 (d, J = 5.7 Hz, 12H). **^{13}C NMR: (75 MHz, $CDCl_3$) δ =** 159.16, 158.46, 153.02, 141.92, 134.69, 133.71, 130.68, 130.60, 128.14, 127.29, 126.95, 125.03, 124.17, 113.41, 113.34, 55.12, 55.11, 45.86, 26.69, 24.14, 24.10. **HRMS (DART, [M+1]$^+$)** m/z calcd. For $C_{38}H_{44}N_3O_4$: 606.3332; found: 606.3327.

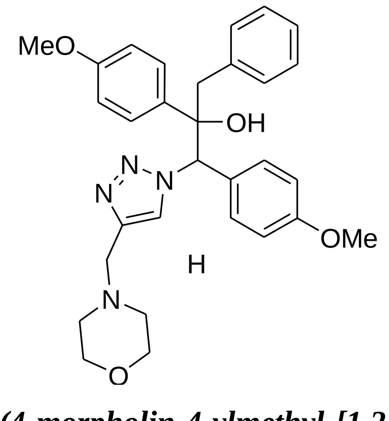

1,2-Bis-(4-methoxyphenyl)-1-(4-morpholin-4-ylmethyl-[1,2,3]triazol-1-yl)-3-phenyl-propan-2-ol (45)

Yield: 262 mg (51.0 %), m.p. 161.5–162.4 °C. **IR (ATR, cm^{-1}):** 3437, 3143, 2926, 2835, 1741, 1611, 1513, 1456, 1254, 1178, 1118, 1029, 752, 701, 547**. ^{1}H NMR: (300 MHz,**

$CDCl_3$) δ 7.93 (s, 1H), 7.16 (d, *J* = 8.6 Hz, 3H), 7.08 – 6.94 (m, 6H), 6.67 – 6.51 (m, 7H), 5.99 (s, 1H), 3.66 (d, *J* = 6.0 Hz, 10H), 3.60 (s, 3H), 3.29 (d, *J* = 13.5 Hz, 1H), 3.07 (s, 1H), 2.56 (d, *J* = 13.5 Hz, 1H). **^{13}C NMR: (75 MHz, $CDCl_3$) δ =** 159.09, 158.41, 134.73, 133.67, 131.21, 130.62, 130.55, 130.19, 130.03, 129.79, 128.45, 128.33, 127.26, 126.91, 125.91, 124.09, 114.23, 114.11, 113.65, 113.38, 113.29, 112.82, 112.52, 79.21, 72.12, 66.80, 65.85, 55.10, 55.07, 53.80, 53.37, 45.74, 37.94, 15.29. **HRMS (DART, $[M+1]^+$)** m/z calcd. For $C_{30}H_{35}N_4O_4$: 515.2658; found: 515.2656.

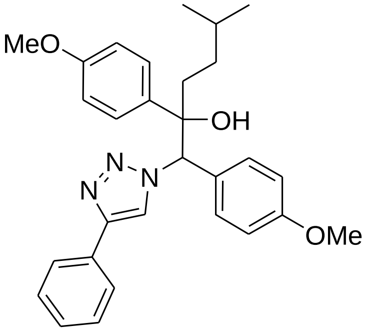

1,2-Bis-(4-methoxyphenyl)-5-methyl-1-(4-phenyl-[1,2,3]triazol-1-yl)-hexan-2-ol (46)

Yield: 296 mg (63.0 %), m.p. 175–175.6 °C. **IR (ATR, cm^{-1}):** 3249, 3151, 3001, 2952, 2867, 1741, 1611, 1510, 1461, 1365, 1248, 1177, 1036, 766, 561. **^1H NMR: (300 MHz, $CDCl_3$) δ** 8.13 – 8.04 (m, 1H), 7.94 – 7.80 (m, 2H), 7.44 (ddd, *J* = 7.6, 6.5, 1.3 Hz, 2H), 7.43 – 7.29 (m, 1H), 7.27 – 7.15 (m, 2H), 7.18 – 7.06 (m, 2H), 6.92 – 6.74 (m, 2H), 6.72 – 6.60 (m, 2H), 5.77 (s, 1H), 3.91 – 3.67 (m, 7H), 2.06 (td, *J* = 13.0, 4.3 Hz, 1H), 1.62 – 1.46 (m, 1H), 1.45 – 1.12 (m, 2H), 1.24 (s, 1H), 0.74 (d, *J* = 6.4 Hz, 7H). **^{13}C NMR: (75 MHz, $CDCl_3$) δ =** 159.13, 158.37, 147.20, 133.71, 130.18, 128.88, 128.29, 127.56, 127.09, 125.75, 121.28, 113.41, 113.25, 79.59, 73.71, 55.15, 55.11, 37.27, 32.19, 28.08, 22.82, 22.18. **HRMS (DART, $[M+1]^+$)** m/z calcd. For $C_{29}H_{34}N_3O_3$: 472.2600; found: 472.2589.

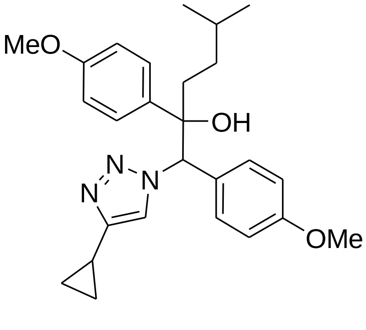

1-(4-Cyclopropyl-[1,2,3]triazol-1-yl)-1,2-bis-(4-methoxyphenyl)-5-methyl-hexan-2-ol (47)

Yield: 200 mg (46.2 %), m.p. 155–155.7 °C. **IR (ATR, cm^{-1}):** 3264, 3178, 2958, 2837, 1741, 1611, 1511, 1249, 1178, 1029, 801, 552. **^{1}H NMR: (300 MHz, $CDCl_3$) δ** 7.38 (s, 1H), 7.11 – 7.00 (m, 2H), 6.99 – 6.88 (m, 2H), 6.73 – 6.62 (m, 2H), 6.58 – 6.47 (m, 2H), 5.50 (s, 1H), 3.89 – 3.82 (m, 1H), 3.68 (s, 3H), 3.61 (s, 3H), 1.97 – 1.80 (m, 1H), 1.44 – 1.17 (m, 2H), 1.21 – 1.02 (m, 1H), 0.94 – 0.73 (m, 4H), 0.78 – 0.61 (m, 7H). **^{13}C NMR: (75 MHz, $CDCl_3$) δ =** 159.01, 158.28, 149.60, 133.76, 130.09, 127.72, 127.07, 121.55, 113.31, 113.09, 79.51, 73.50, 55.13, 55.08, 37.13, 32.19, 28.08, 22.81, 22.19, 7.87, 7.83. **HRMS (DART, [M+1]$^{+}$)** m/z calcd. For $C_{26}H_{34}N_3O_3$: 436.2600; found: 436.2594.

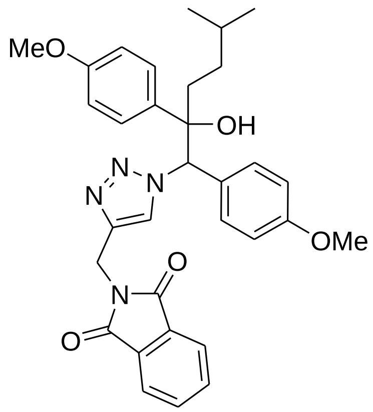

2-{1-[2-Hydroxy-1,2-bis-(4-methoxyphenyl)-5-methyl-hexyl]-[1,2,3]triazol-4-ylmethyl}-isoindole-1,3-dione (48)

Yield: 288 mg (52.0 %), m.p. 173.3-174.4 °C. **IR (ATR, cm^{-1}):** 3462, 3145, 2954, 2868, 2837, 1703, 1613, 1515, 1398, 1254, 1097, 764, 715, 521. **^{1}H NMR: (300 MHz, $CDCl_3$) δ** 7.90 – 7.79 (m, 3H), 7.72 (dt, J = 5.4, 3.3 Hz, 2H), 7.17 – 6.98 (m, 5H), 6.81 – 6.70 (m, 2H), 6.66 – 6.55 (m, 2H), 5.63 (s, 1H), 5.02 (s, 3H), 3.72 (d, J = 22.6 Hz, 6H), 1.95 (td, J = 13.0, 4.3 Hz, 1H), 1.34 (qd, J = 12.6, 5.3 Hz, 2H), 1.14 (tt, J = 12.4, 5.1 Hz, 1H), 0.69 (dd, J = 6.6, 1.6 Hz, 8H). **^{13}C NMR: (75 MHz, $CDCl_3$) δ =** 167.63, 159.08, 158.32, 134.08, 133.57, 132.05, 130.19, 127.28, 127.03, 124.62, 123.46, 113.34, 113.15, 79.47, 73.80, 55.12, 55.08, 37.02, 33.02, 32.12, 28.00, 22.70, 22.17. **HRMS (DART, [M+1]$^{+}$)** m/z calcd. For $C_{32}H_{35}N_4O_5$: 555.2607; found: 555.2597.

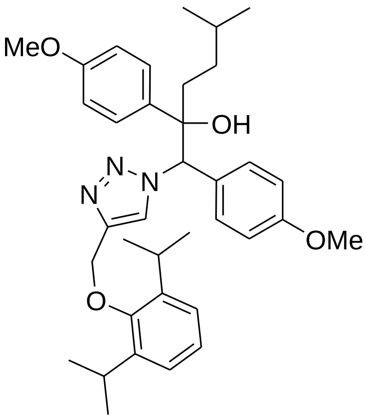

1-[4-(2,6-Diisopropylphenoxymethyl)-[1,2,3]triazol-1-yl]-1,2-bis-(4-methoxyphenyl)-5-methyl-hexan-2-ol (49)

Yield: 288 mg (55.0 %), pale-yellow oil. **IR (ATR, cm^{-1}):** 3453, 2958, 2866, 1741, 1608, 1512, 1447, 1249, 1177, 1119, 1033, 802. **^{1}H NMR: (300 MHz, CDCl$_3$) δ** 7.84 (s, 1H), 7.13 – 7.04 (m, 2H), 7.05 (s, 3H), 7.03 – 6.95 (m, 2H), 6.74 – 6.65 (m, 2H), 6.61 – 6.52 (m, 2H), 5.64 (s, 1H), 4.90 (s, 2H), 3.69 (s, 3H), 3.63 (s, 3H), 3.30 (hept, *J* = 6.9 Hz, 2H), 1.94 (td, *J* = 13.5, 4.7 Hz, 1H), 1.47 – 1.35 (m, 1H), 1.33 – 1.22 (m, 1H), 1.15 (d, *J* = 6.9 Hz, 13H), 0.80 – 0.68 (m, 1H), 0.73 (s, 1H), 0.67 (dd, *J* = 6.6, 2.9 Hz, 6H). **^{13}C NMR: (75 MHz, CDCl$_3$) δ =** 193.49, 159.17, 158.40, 152.88, 144.28, 141.87, 133.65, 130.20, 127.49, 127.06, 125.03, 124.15, 124.11, 113.41, 113.23, 79.55, 73.83, 68.14, 55.14, 55.12, 37.22, 32.22, 28.08, 26.64, 24.06, 22.80, 22.19, 1.85. **HRMS (DART, [M+1]$^+$)** m/z calcd. For $C_{36}H_{48}N_3O_4$: 586.3645; found: 586.3636.

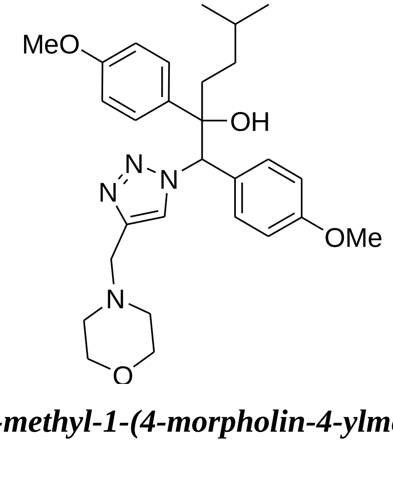

1,2-Bis-(4-methoxyphenyl)-5-methyl-1-(4-morpholin-4-ylmethyl-[1,2,3]triazol-1-yl)-hexan-2-ol (50)

Yield: 296 mg (60.0 %), 153.5–154. **IR (ATR, cm^{-1}):** 3364, 2951, 2838, 1741, 1609, 1512, 1457, 1246, 1177, 1030, 803. **^{1}H NMR: (300 MHz, $CDCl_3$) δ** 7.79 (s, 1H), 7.16 – 7.02 (m, 4H), 6.78 – 6.70 (m, 2H), 6.65 – 6.57 (m, 2H), 5.68 (s, 1H), 3.74 (s, 3H), 3.68 (s, 2H), 3.64 (dd, *J* = 3.9, 2.0 Hz, 6H), 3.18 (s, 4H), 2.00 – 1.84 (m, 2H), 1.30 (dq, *J* = 13.6, 5.0 Hz, 2H), 1.12 (td, *J* = 12.7, 6.0 Hz, 1H), 0.73 – 0.65 (m, 6H). **^{13}C NMR: (75 MHz, $CDCl_3$) δ =** 177.70, 159.11, 158.33, 142.91, 133.72, 130.24, 127.45, 127.03, 124.53, 113.36, 113.22, 79.46, 73.59, 70.57, 66.90, 66.57, 61.62, 55.12, 53.22, 37.07, 32.05, 28.07, 22.55, 22.22. **HRMS (DART, $[M+1]^+$)** m/z calcd. For $C_{28}H_{39}N_4O_4$: 495.2971; found: 495.2957.

4. ^{1}H NMR and ^{13}C NMR spectra for compounds

1-azido-1,2-diphenylbutan-2-ol

^{1}H NMR spectrum

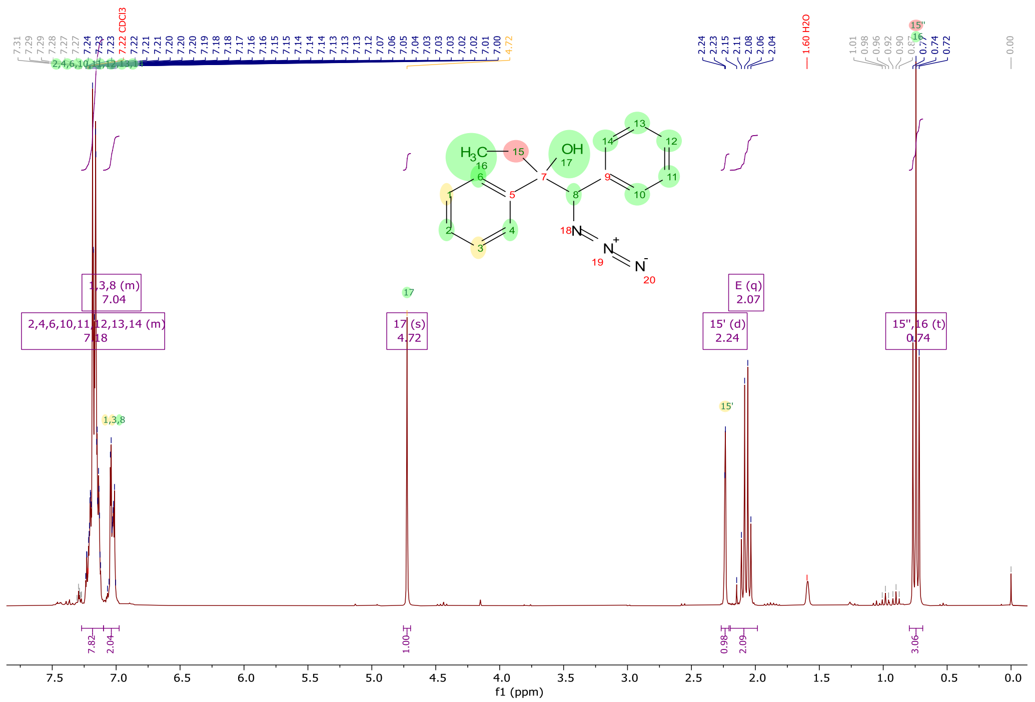

^{13}C NMR spectrum

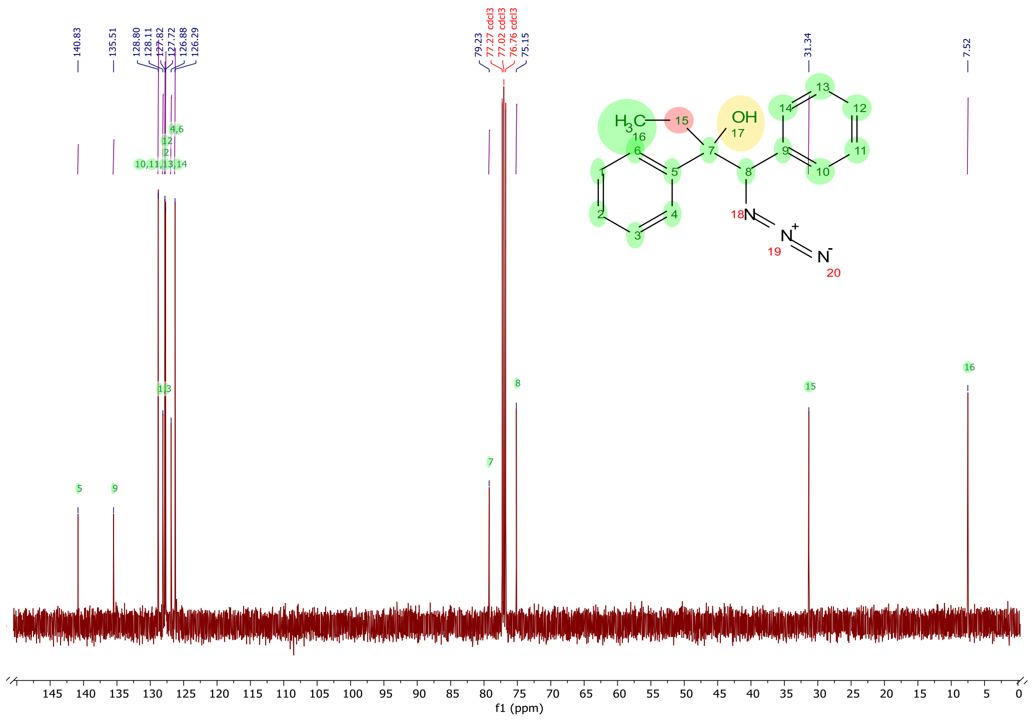

2-Azido-1,2-bis-(4-methoxy-phenyl)-ethanone

^{1}H NMR spectrum

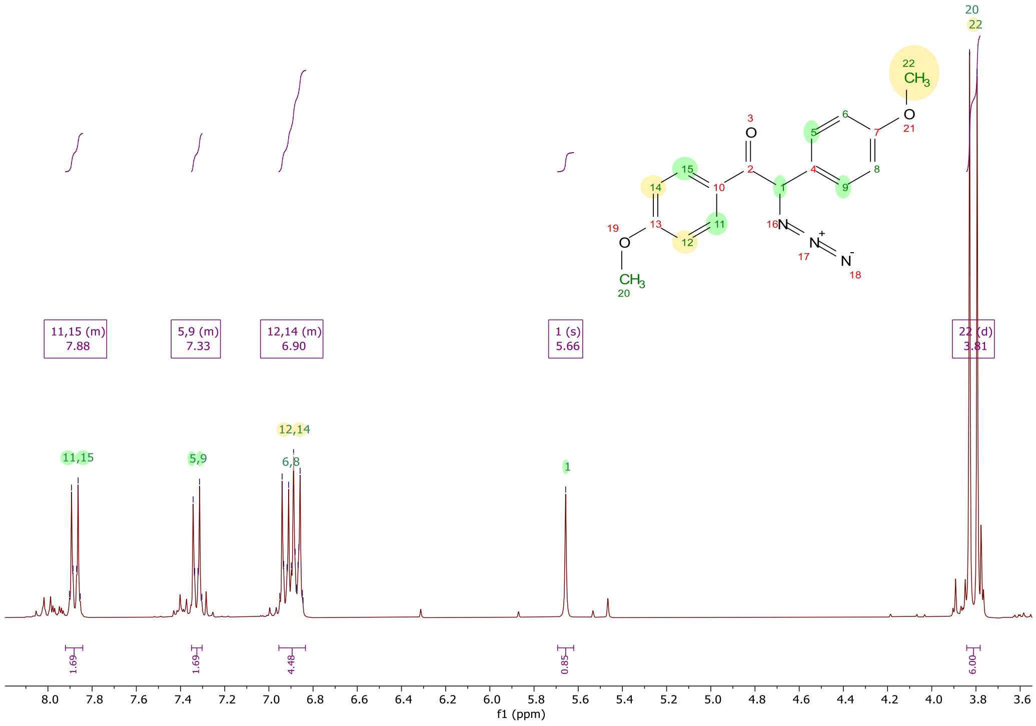

^{13}C NMR spectrum

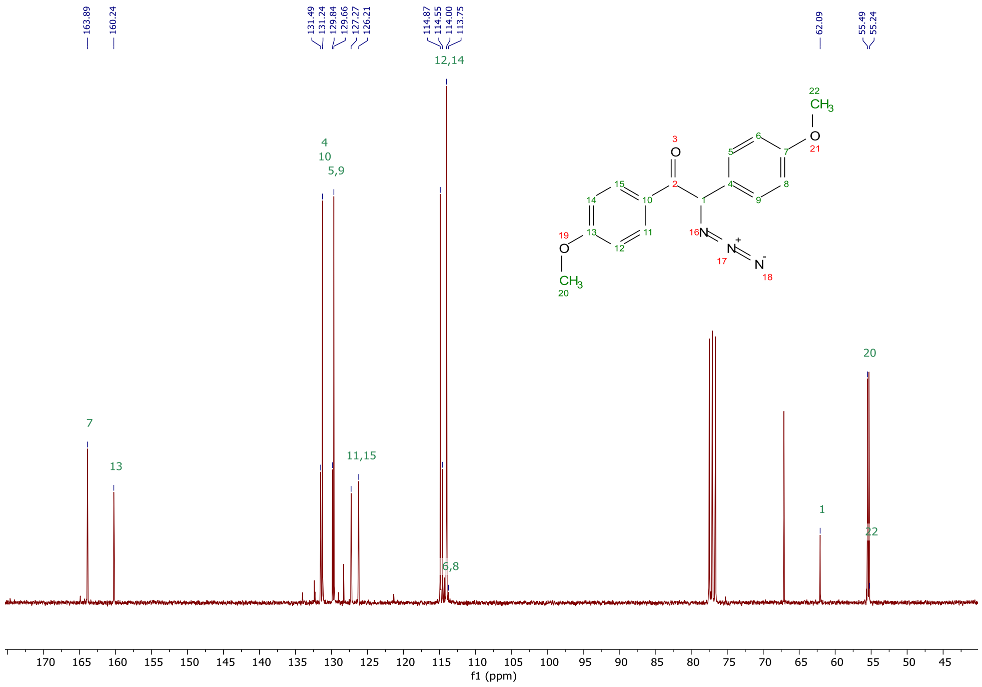

1-azido-1,2-diphenylbutan-2-ol

^{1}H NMR spectru

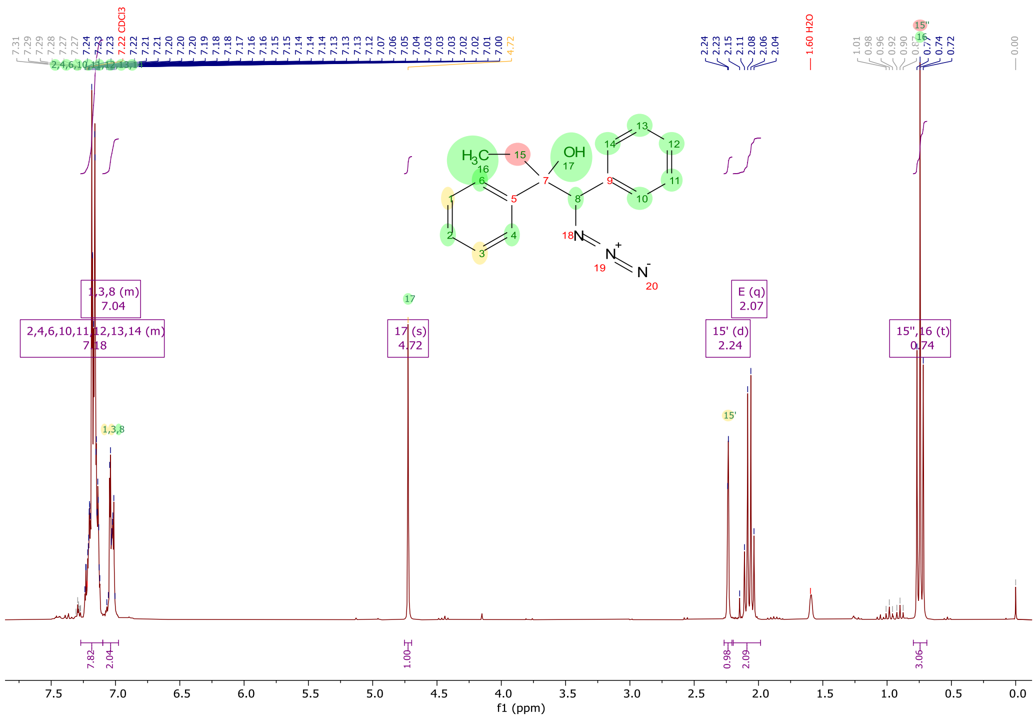

^{13}C NMR spectrum

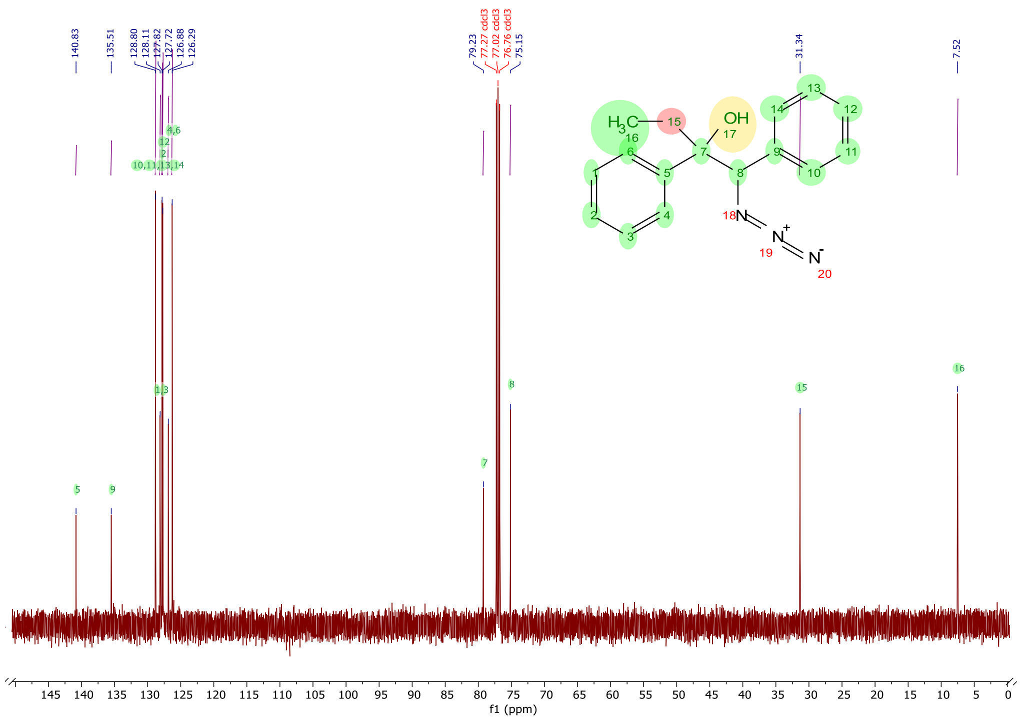

1-azido-1,2-diphenylhexan-2-ol

^{1}H NMR spectrum

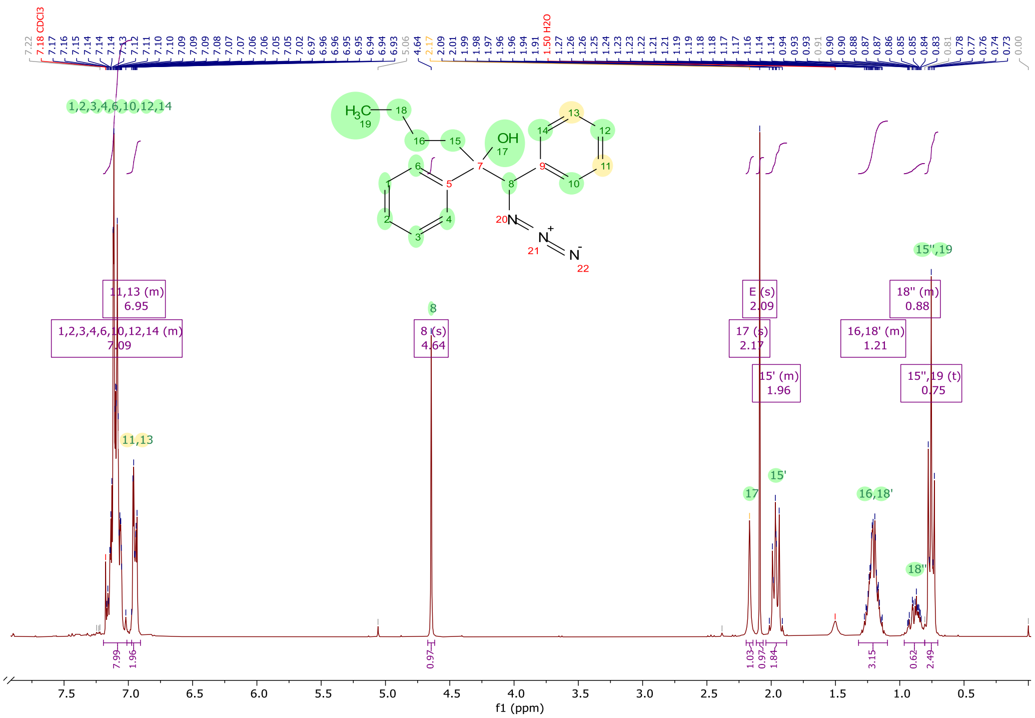

^{13}C NMR spectrum

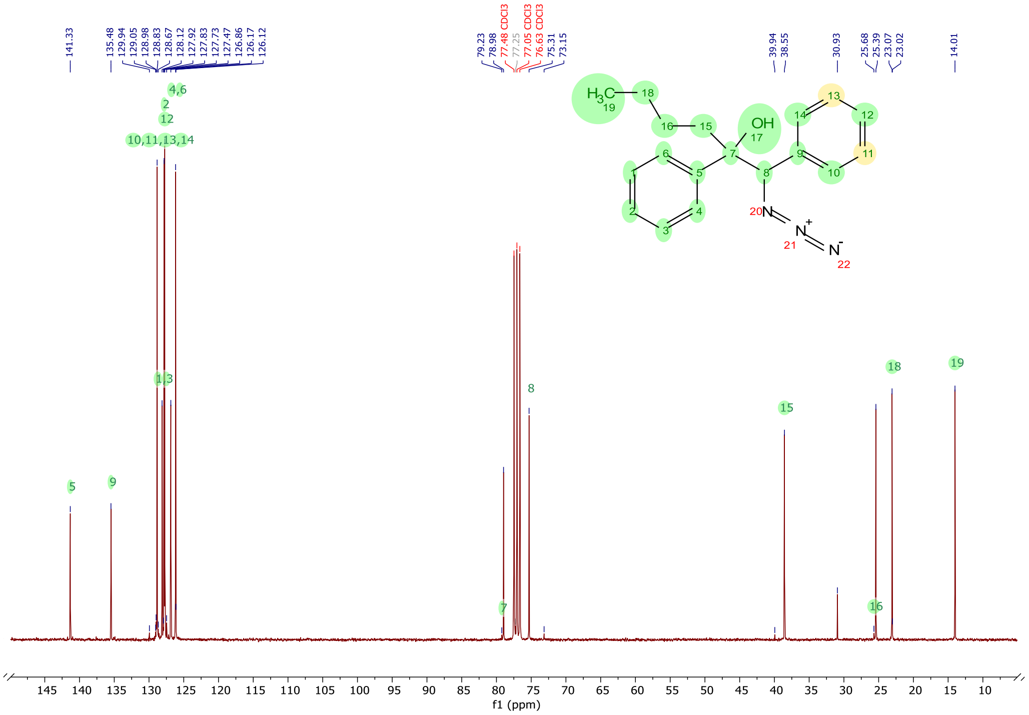

1-azido-1,2,3-triphenylpropan-2-ol

^{1}H NMR spectrum

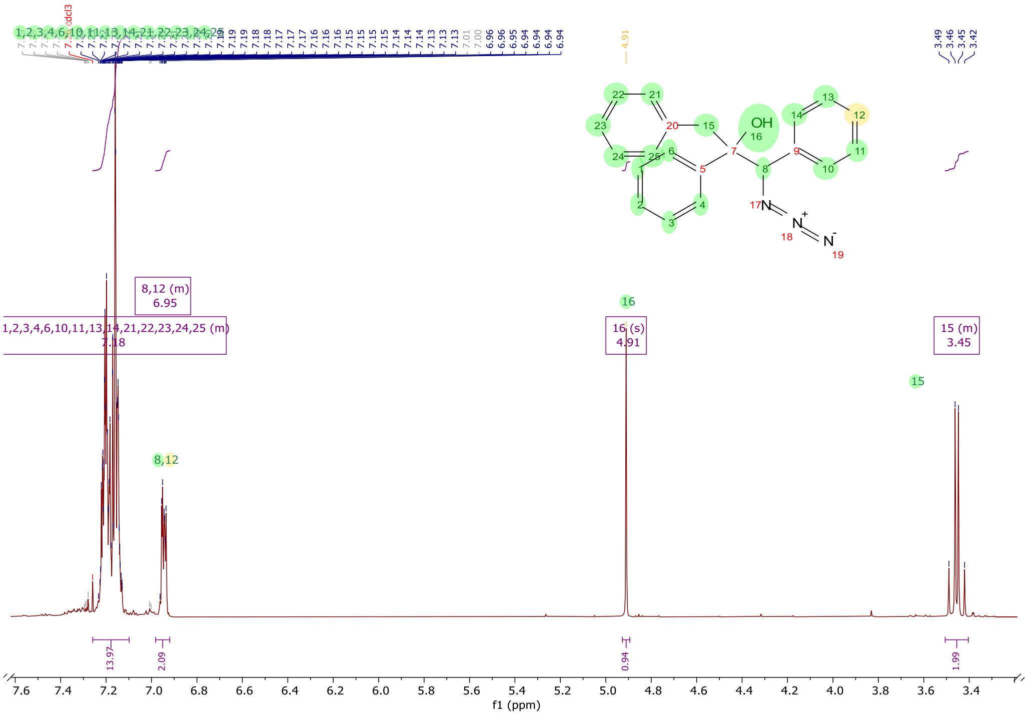

^{13}C NMR spectrum

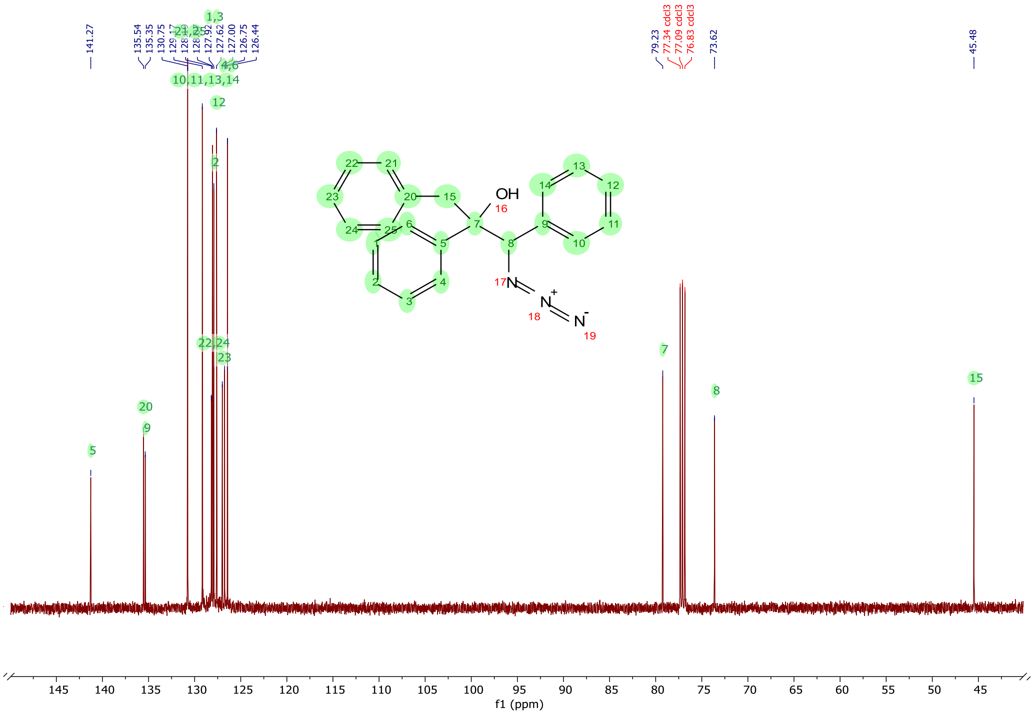

2-azido-1-cyclohexyl-1,2-diphenylethan-1-ol

^{1}H NMR spectrum

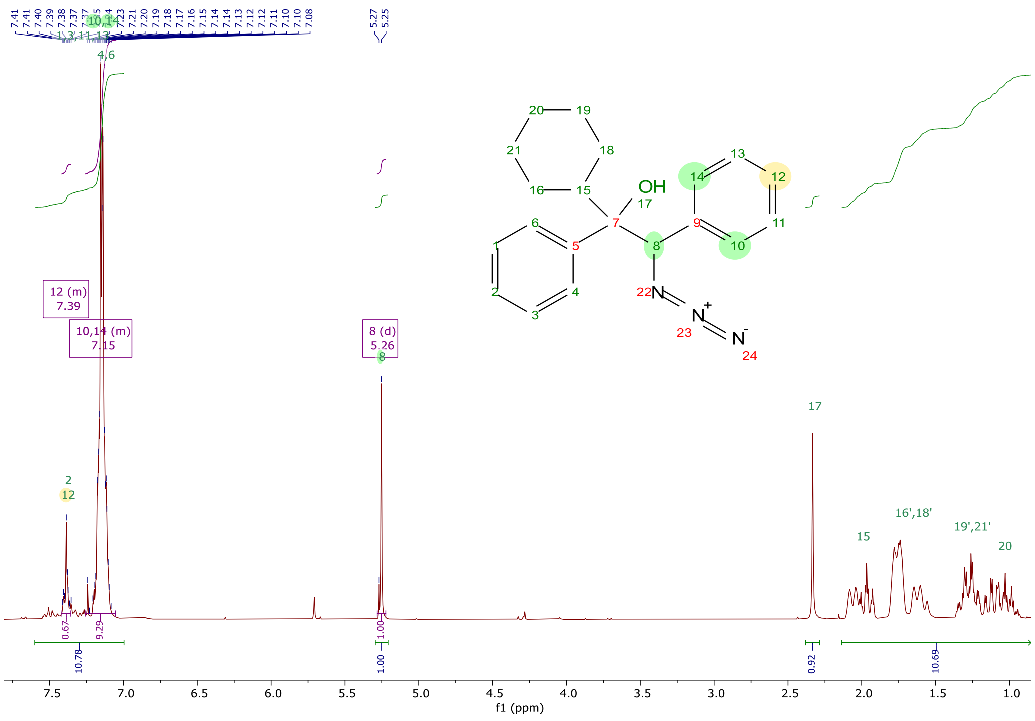

^{13}C NMR spectrum

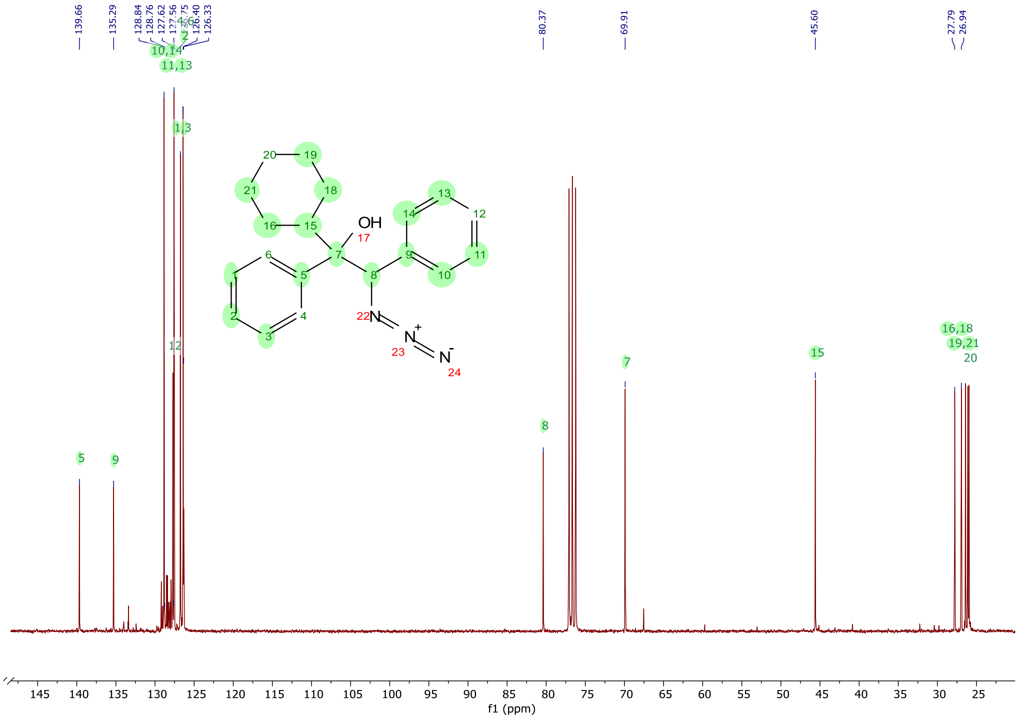

1-azido-1,2-bis(4-methoxyphenyl) butan-2-ol

^{1}H NMR spectrum

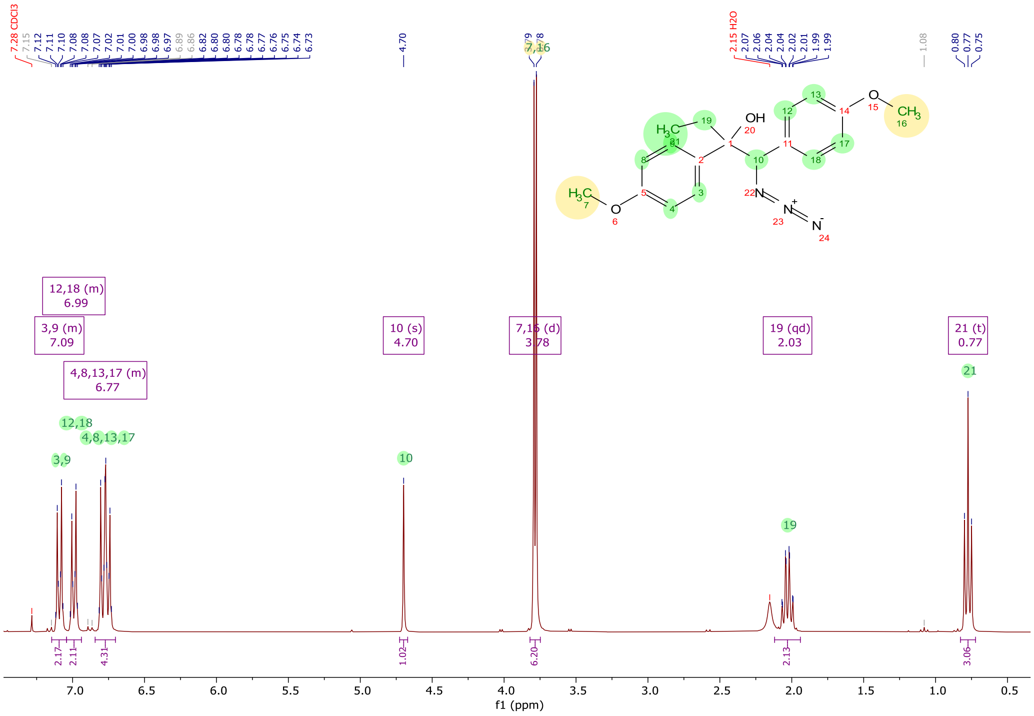

^{13}C NMR spectrum

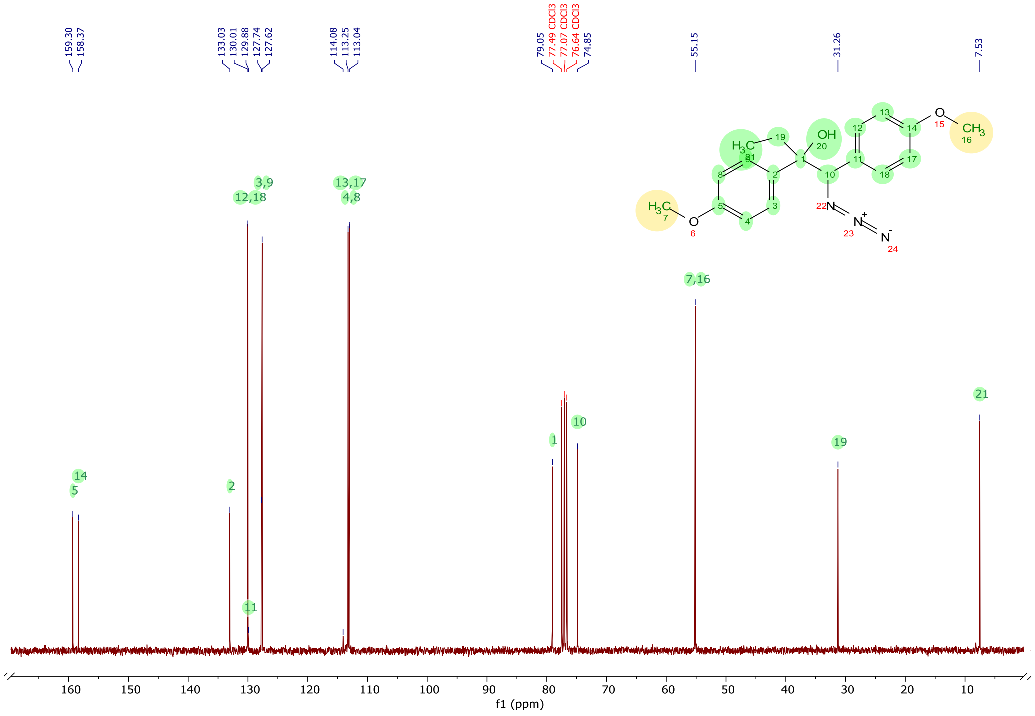

1-azido-1,2-bis(4-methoxyphenyl)-5-methylhexan-2-ol

^{1}H NMR spectrum

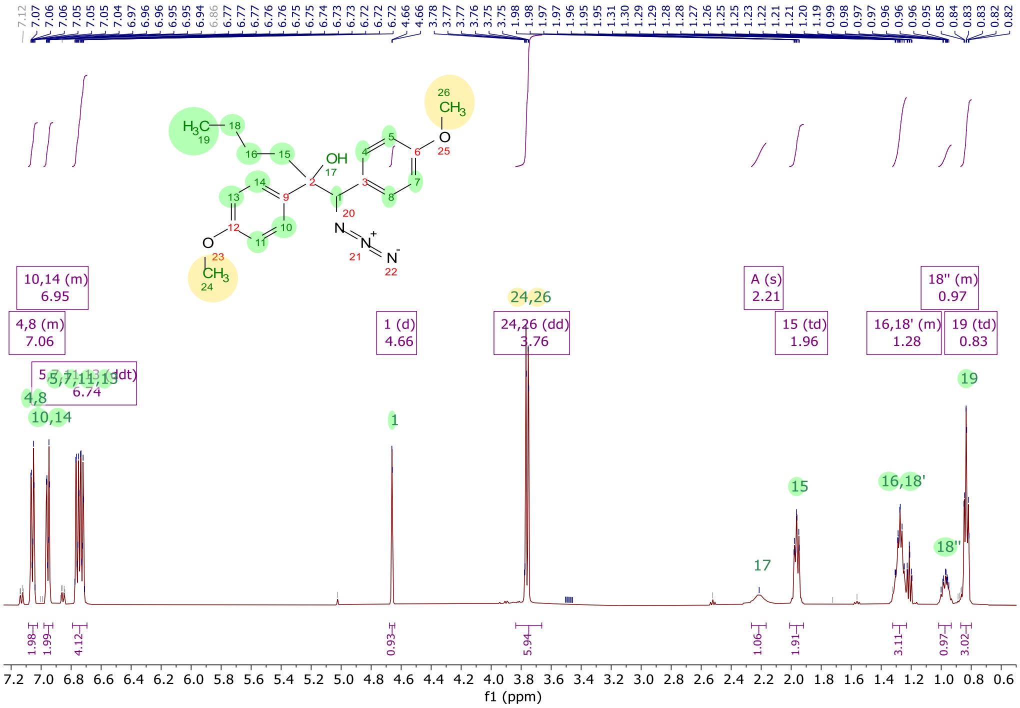

^{13}C NMR spectrum

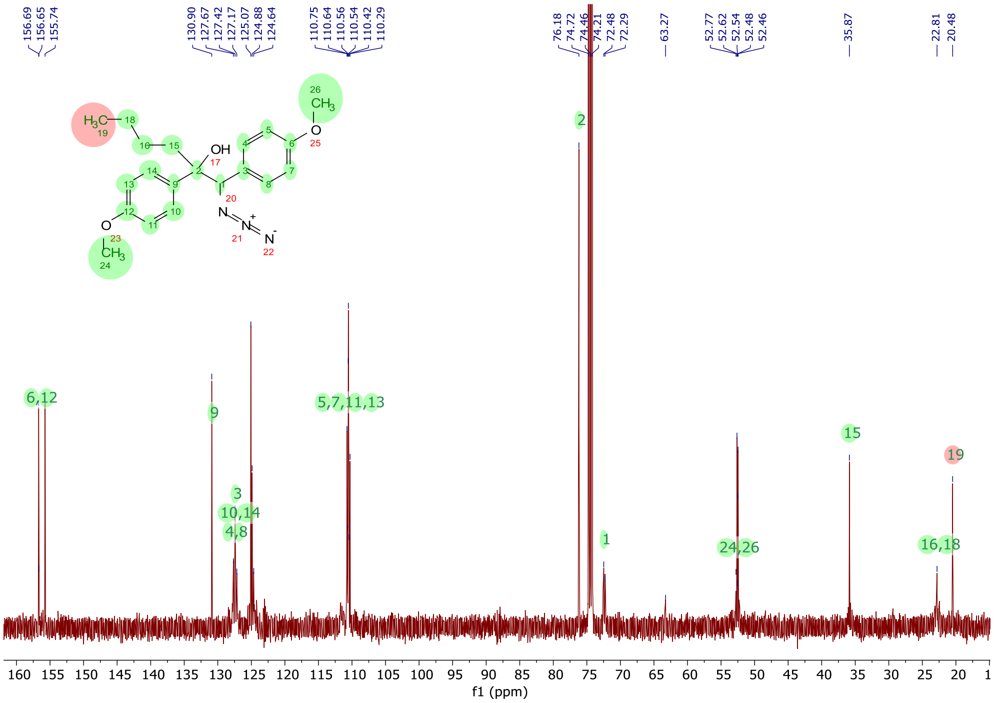

1-azido-1,2-bis(4-methoxyphenyl)-3-phenylpropan-2-ol

^{1}H NMR spectrum

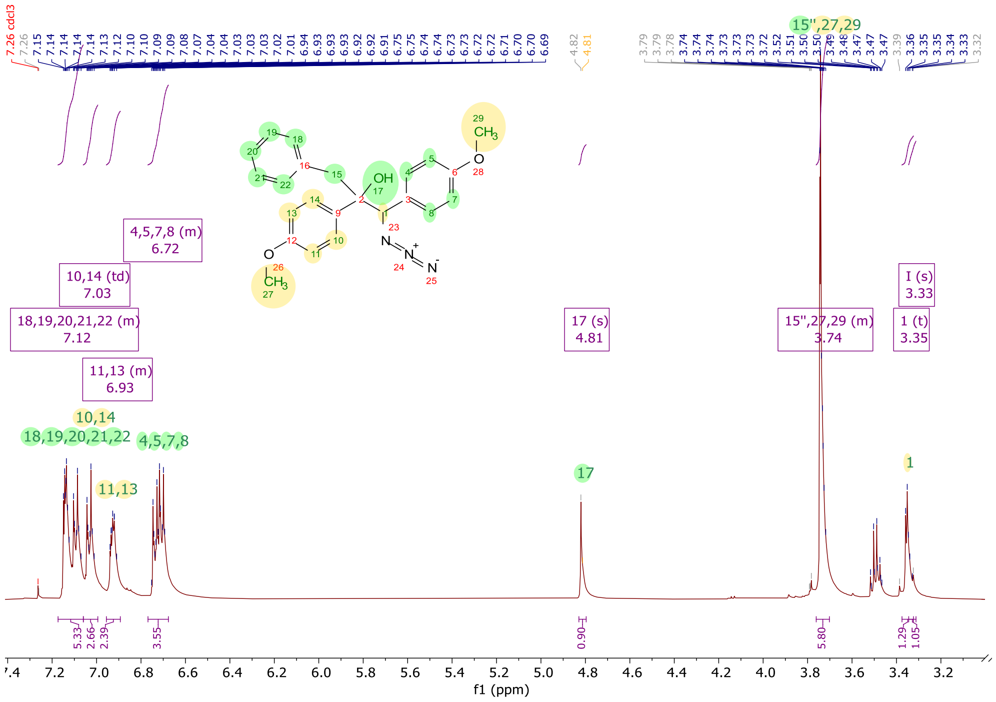

^{13}C NMR spectrum

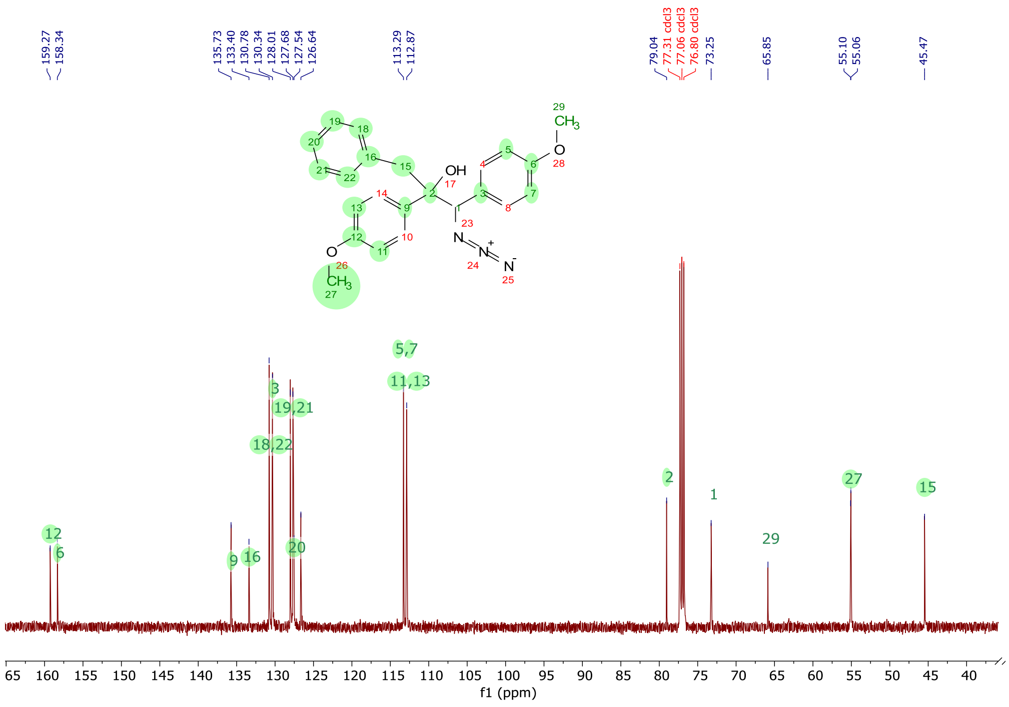

1-azido-1,2-bis(4-metoxifenil)-5-metilhexan-2-ol

^{1}H NMR spectrum

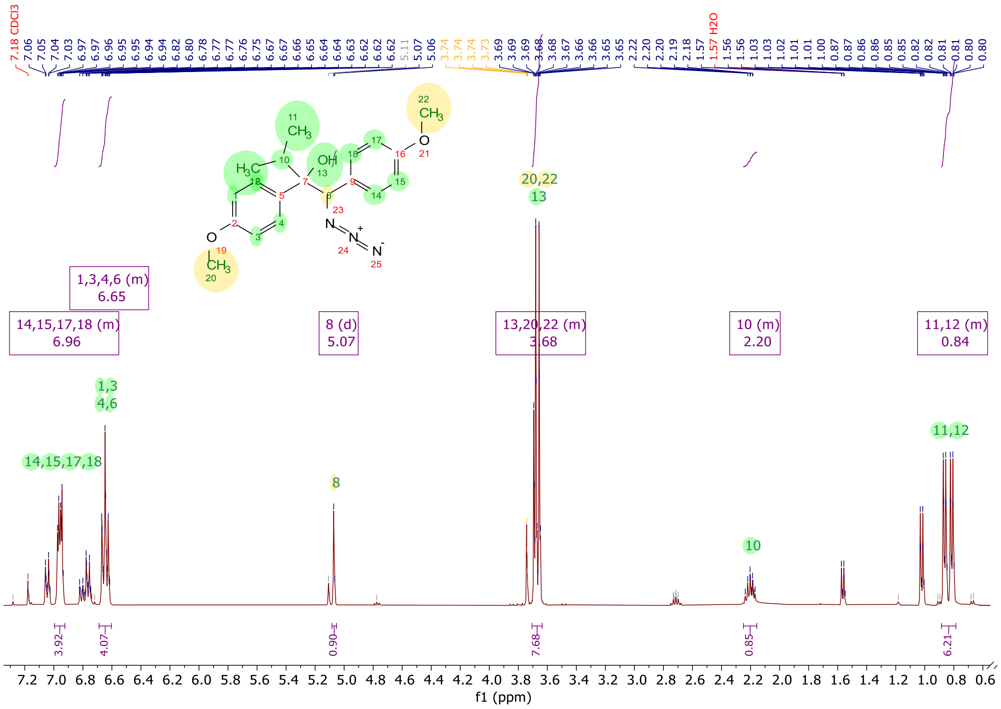

^{13}C NMR spectrum

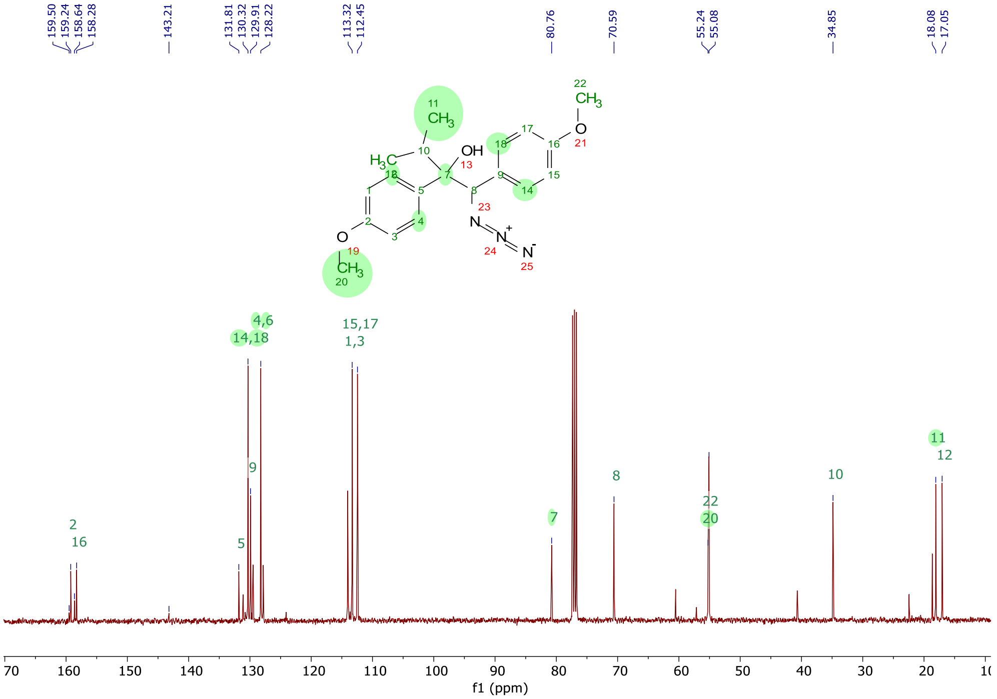

1,2-Diphenyl-1-(4-phenyl-[1,2,3]triazol-1-yl)-butan-2-ol (11)

^{1}H NMR spectrum

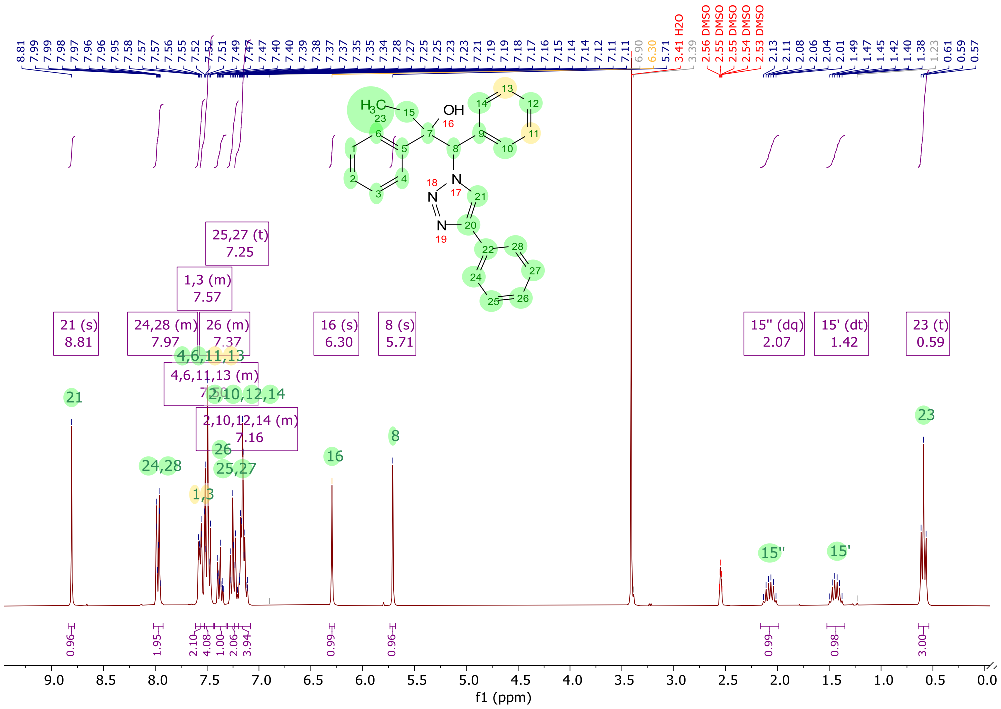

^{13}C NMR spectrum

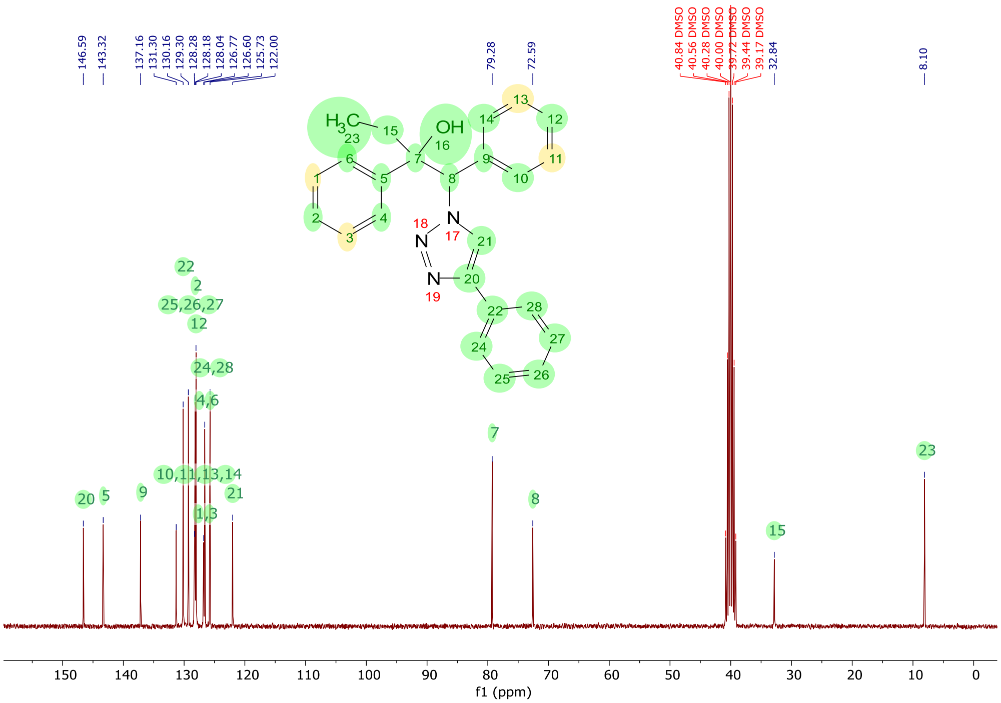

1-(4-Cyclopropyl-[1,2,3]triazol-1-yl)-1,2-diphenyl-butan-2-ol (12)

^{1}H NMR spectrum

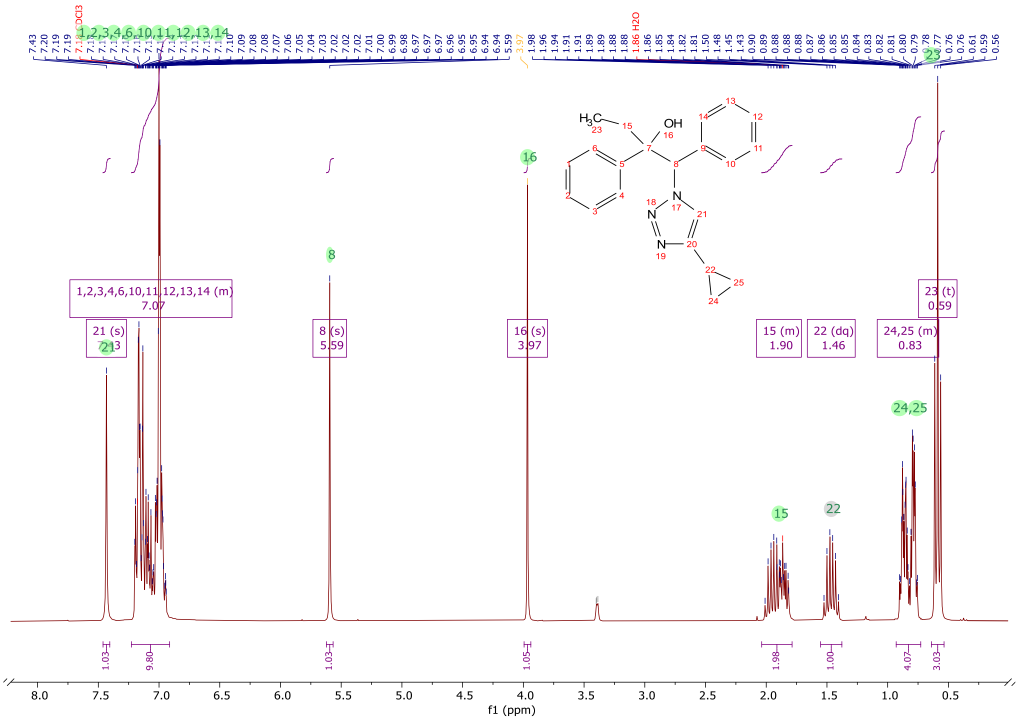

^{13}C NMR spectrum

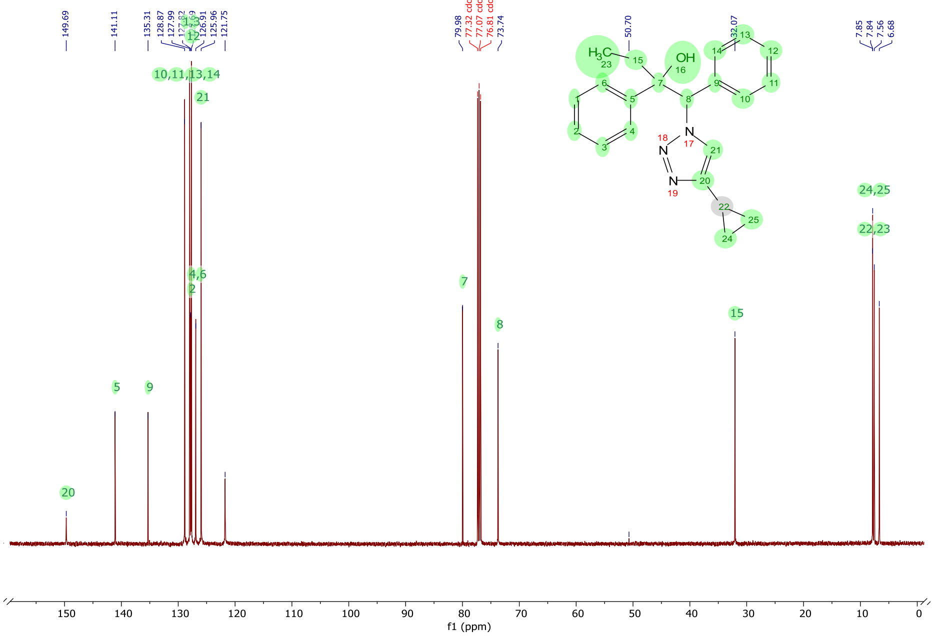

2-[1-(2-Hydroxy-1,2-diphenyl-butyl)-[1,2,3]triazol-4-ylmethyl]-isoindole-1,3-dione (13)

^{1}H NMR spectrum

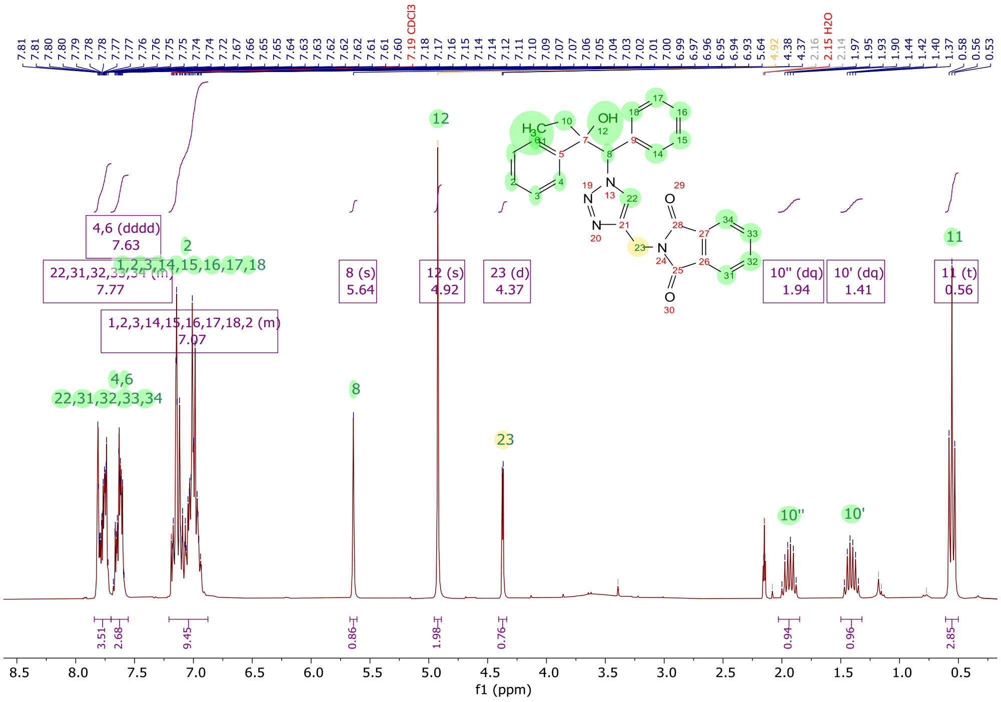

^{13}C NMR spectrum

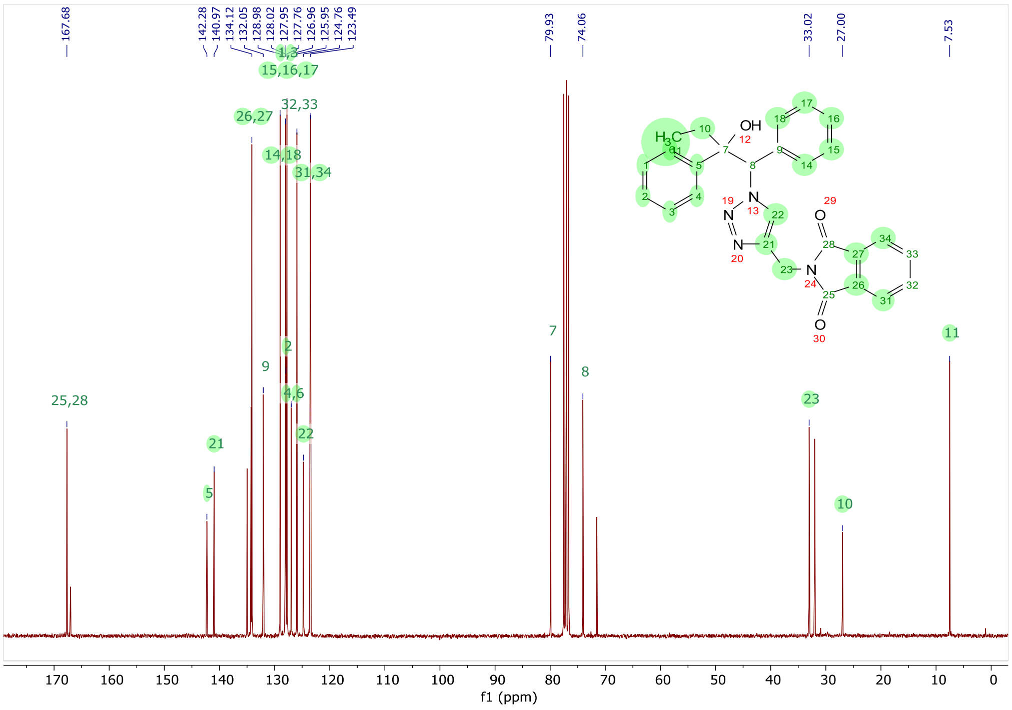

1-[4-(2,6-Diisopropylphenoxymethyl)-[1,2,3]triazol-1-yl]-1,2-diphenyl-butan-2-ol (14)

^{1}H NMR spectrum

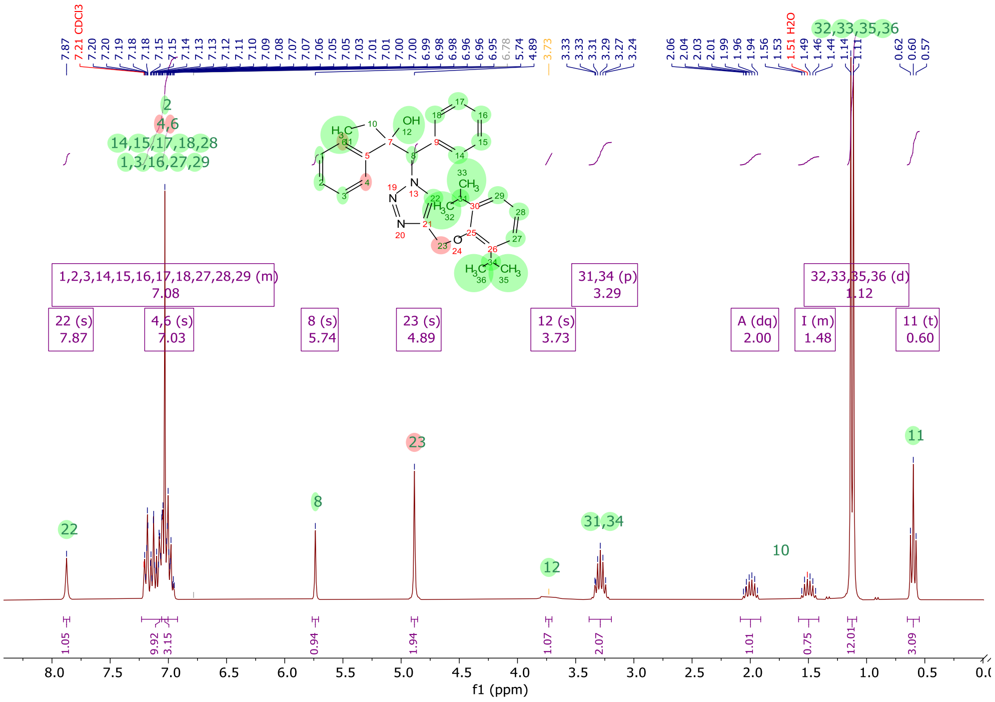

^{13}C NMR spectrum

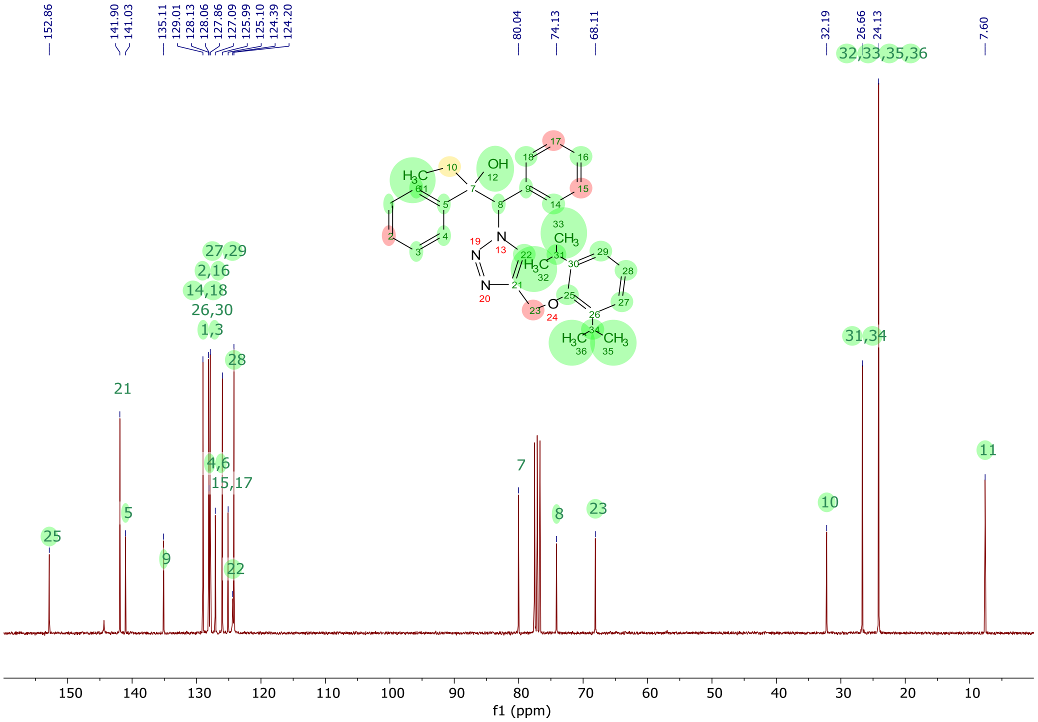

1-(4-Morpholin-4-ylmethyl-[1,2,3]triazol-1-yl)-1,2-diphenyl-butan-2-ol (15)

^{1}H NMR spectrum

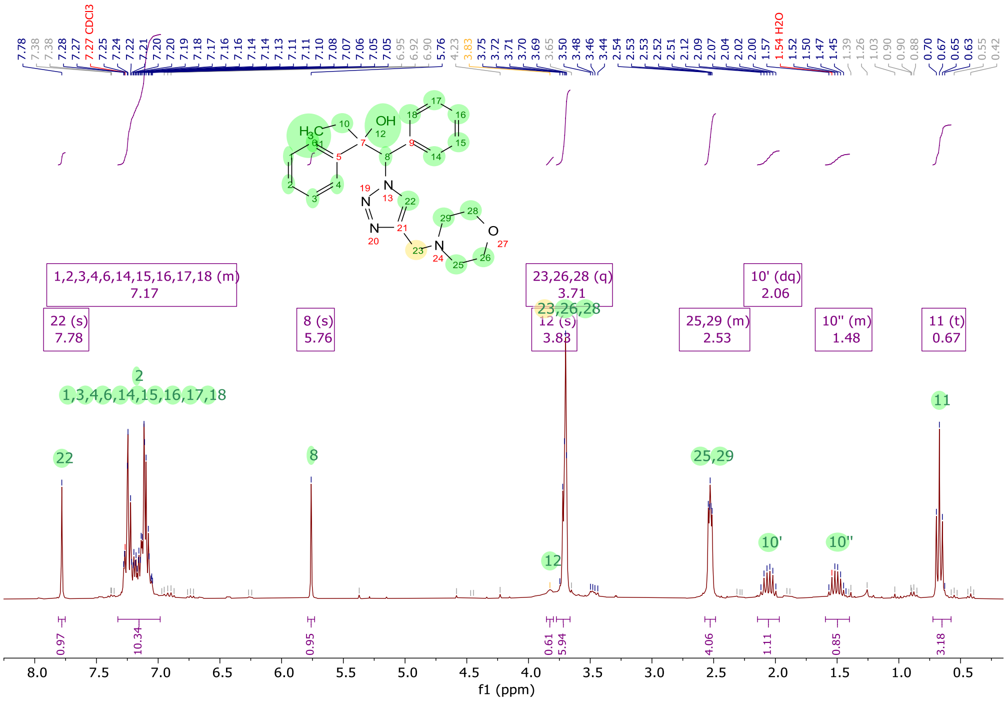

^{13}C NMR spectrum

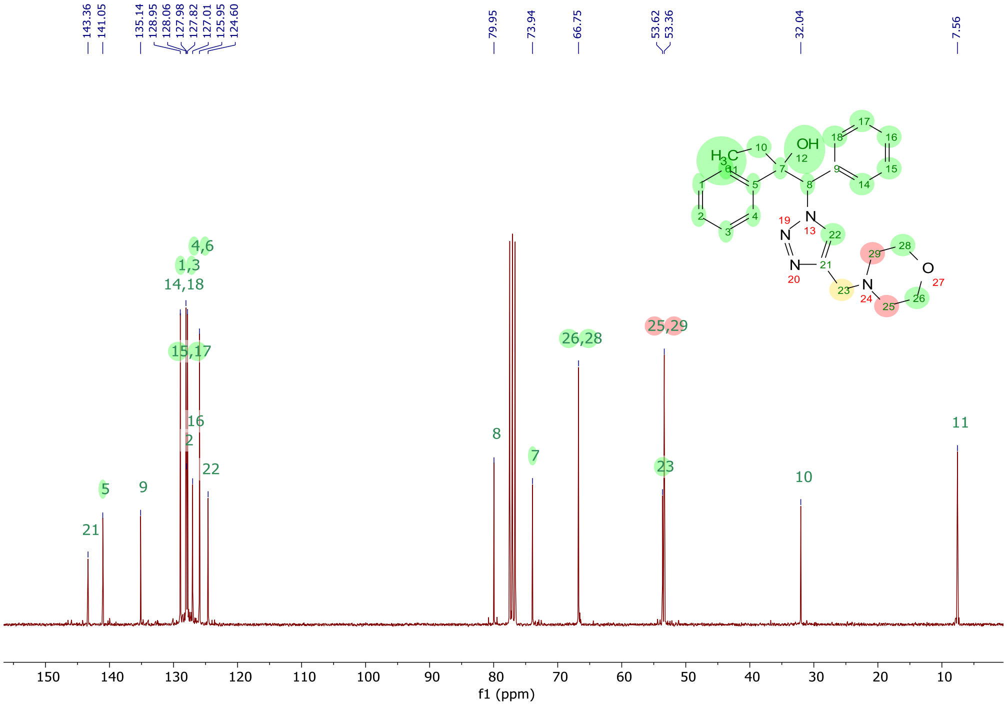

1,2-Diphenyl-1-(4-phenyl-[1,2,3]triazol-1-yl)-hexan-2-ol (16)

^{1}H NMR spectrum

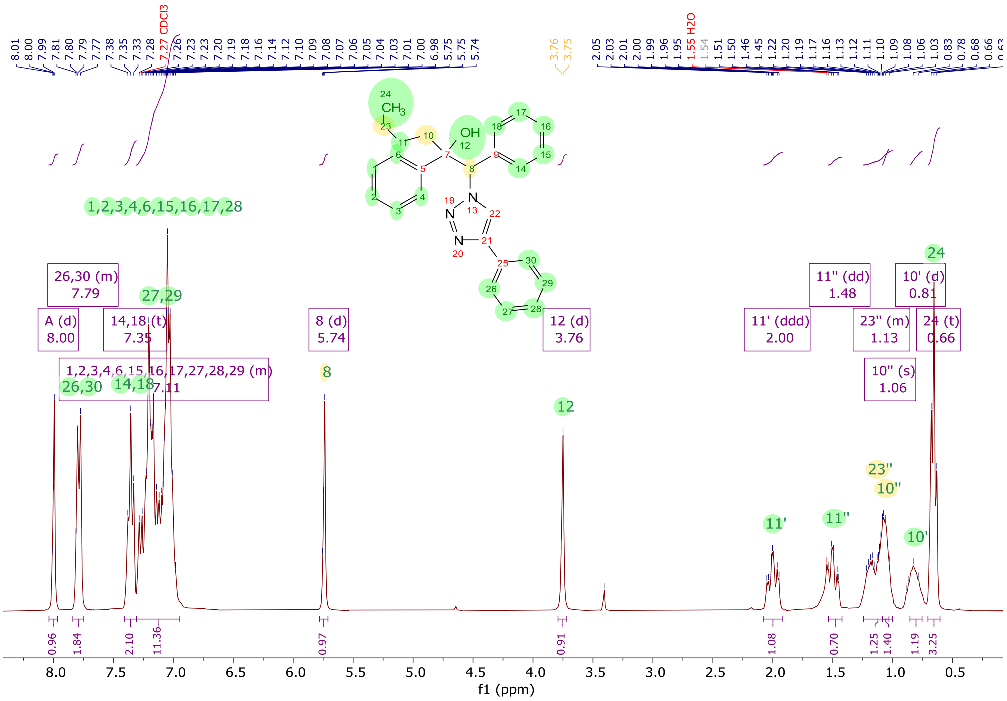

^{13}C NMR spectrum

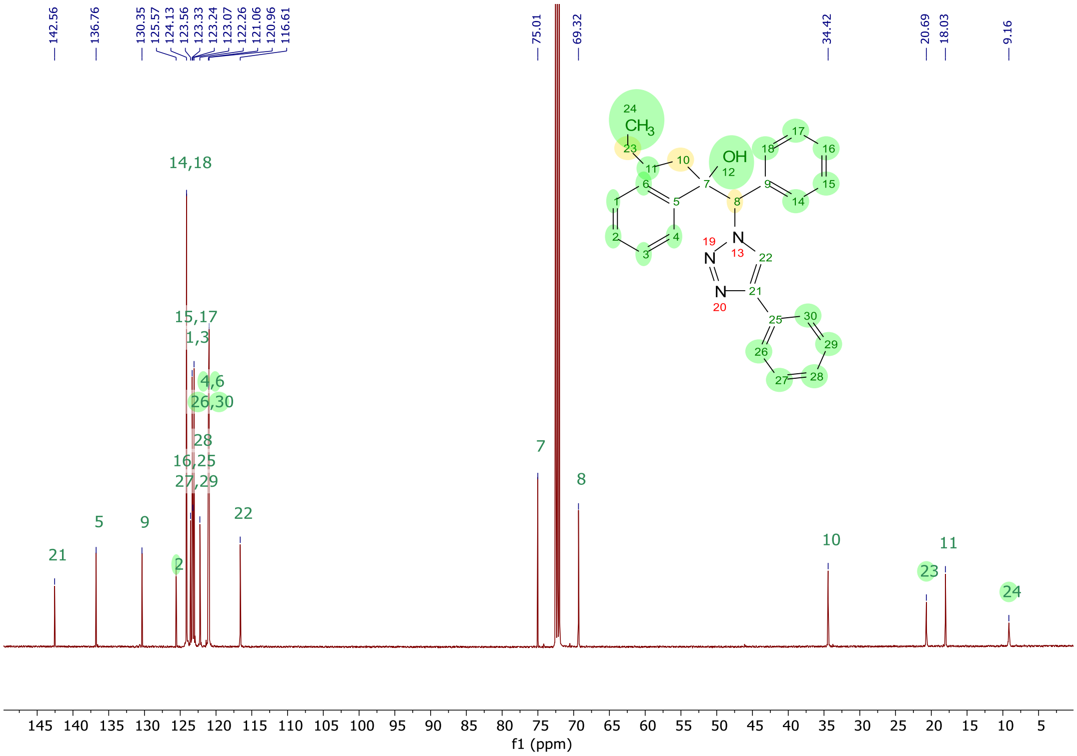

1-(4-Cyclopropyl-[1,2,3]triazol-1-yl)-1,2-diphenyl-hexan-2-ol (17)

^{1}H NMR spectrum

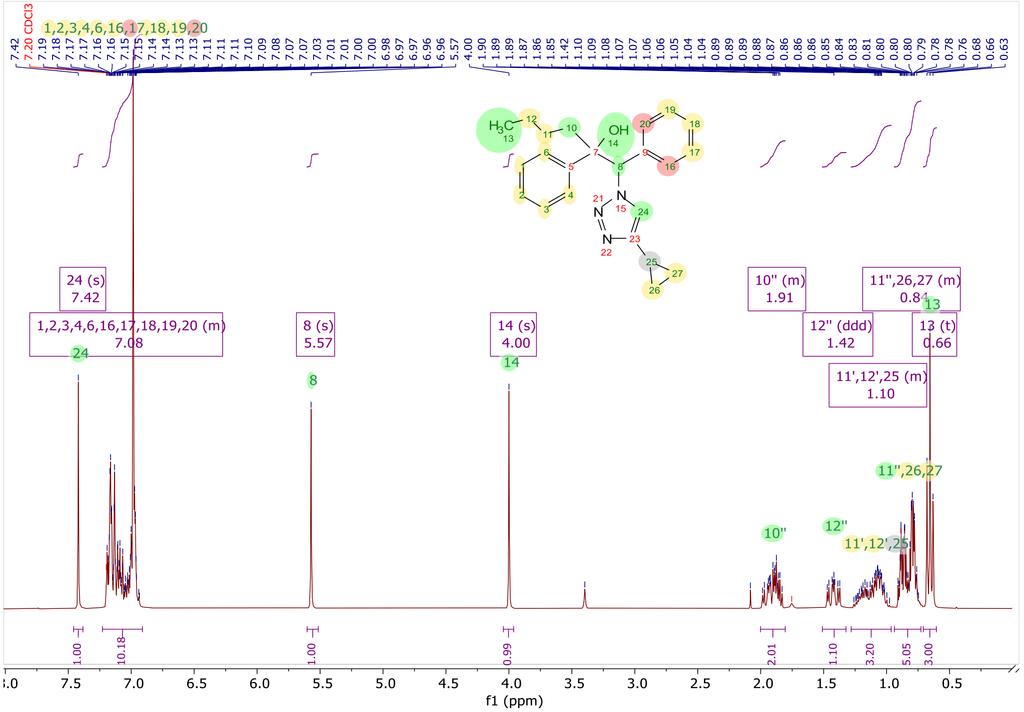

^{13}C NMR spectrum

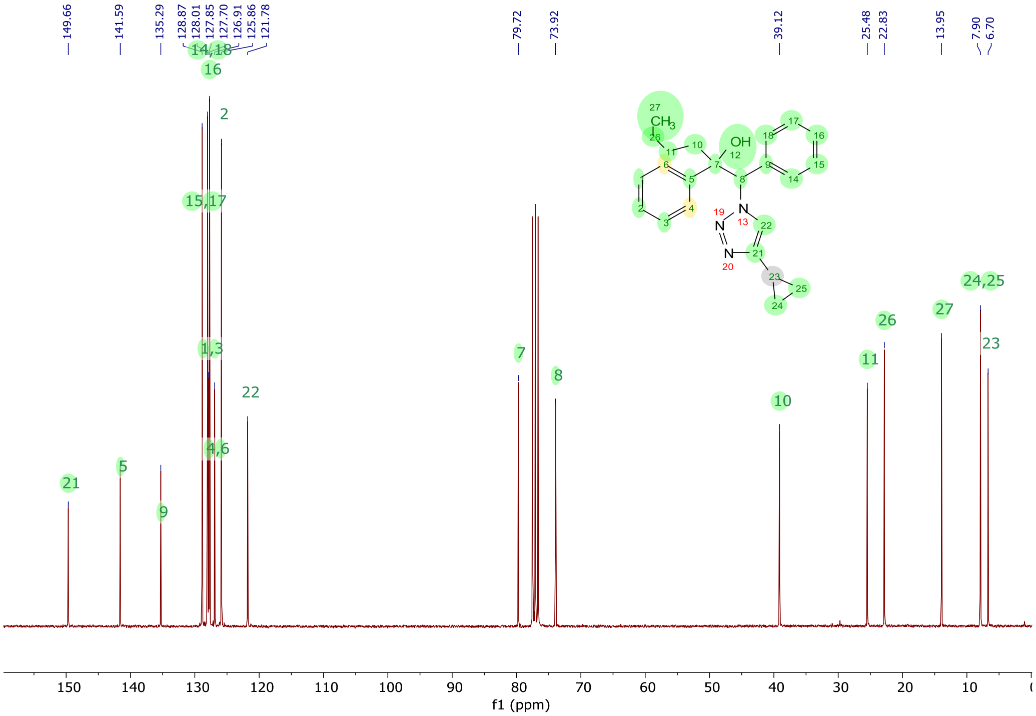

1-(4-Cyclopropyl-[1,2,3]triazol-1-yl)-1,2-diphenyl-hexan-2-ol (18)

^{1}H NMR spectrum

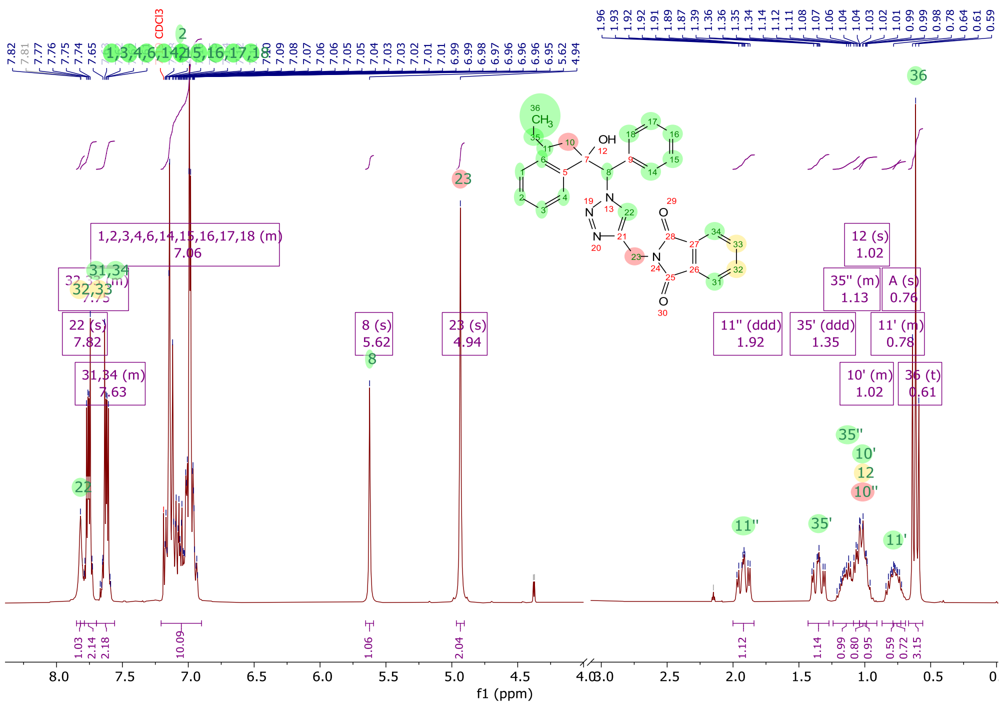

^{13}C NMR spectrum

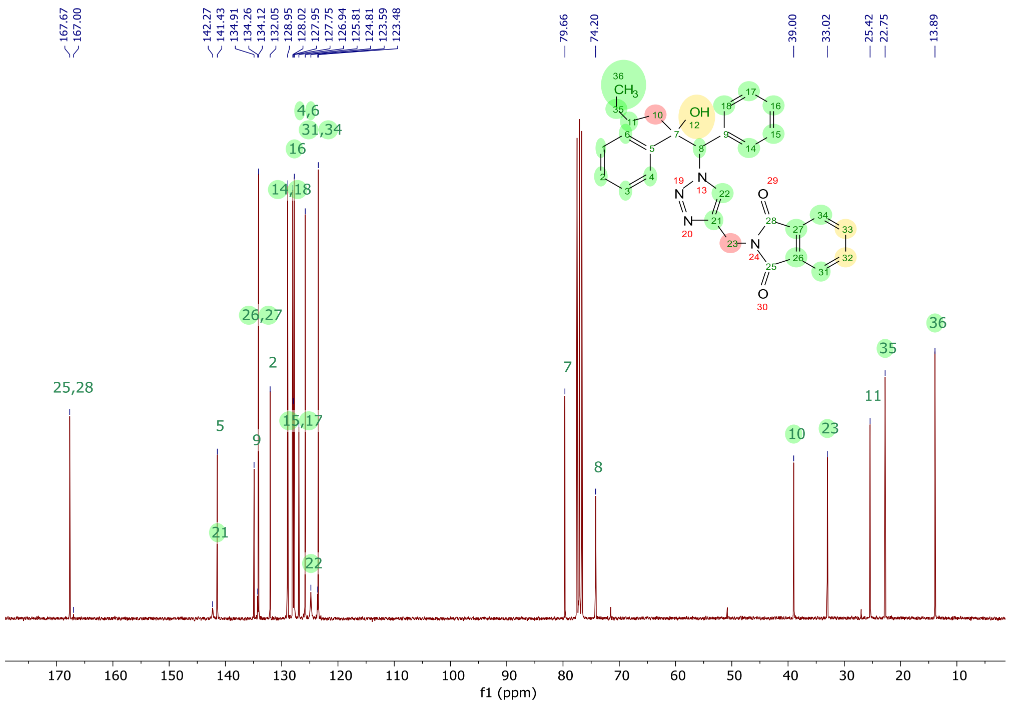

1-[4-(2,6-Diisopropy-phenoxymethyl)-[1,2,3]triazol-1-yl]-1,2-diphenyl-hexan-2-ol (19)

^{1}H NMR spectrum

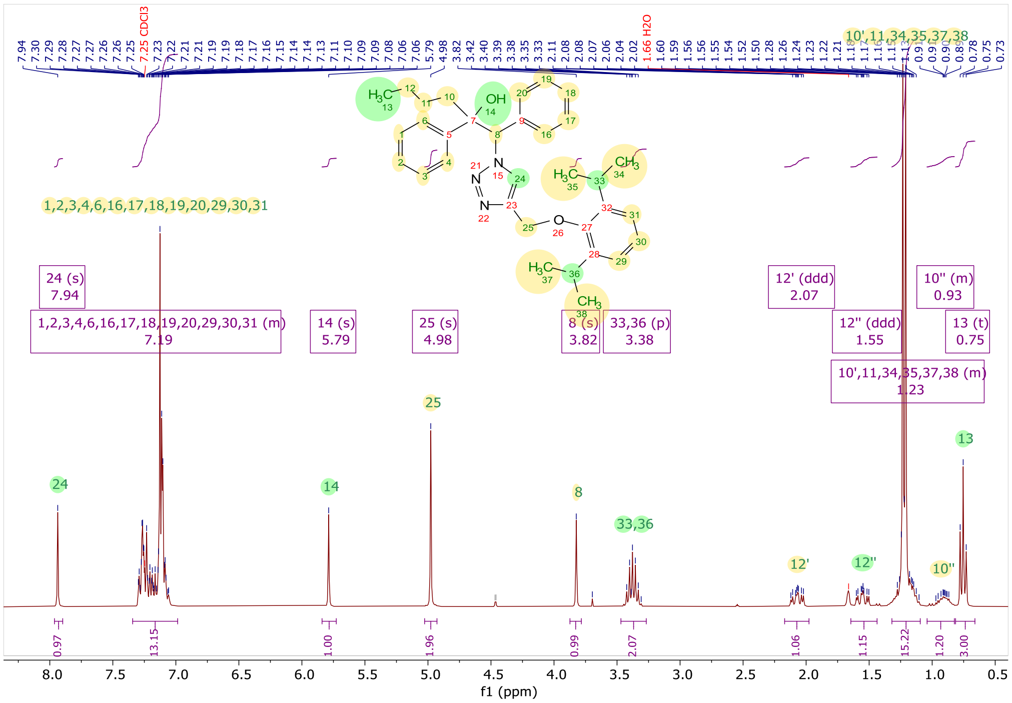

^{13}C NMR spectrum

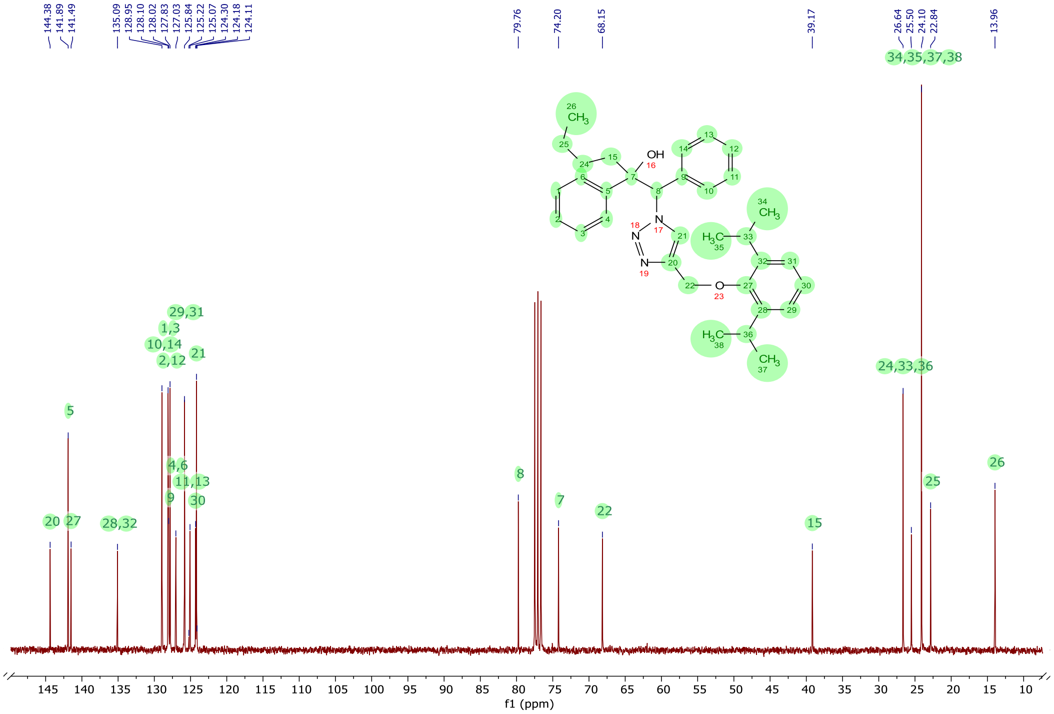

1-(4-Morpholin-4-ylmethyl-[1,2,3]triazol-1-yl)-1,2-diphenyl-hexan-2-ol (20)

^{1}H NMR spectrum

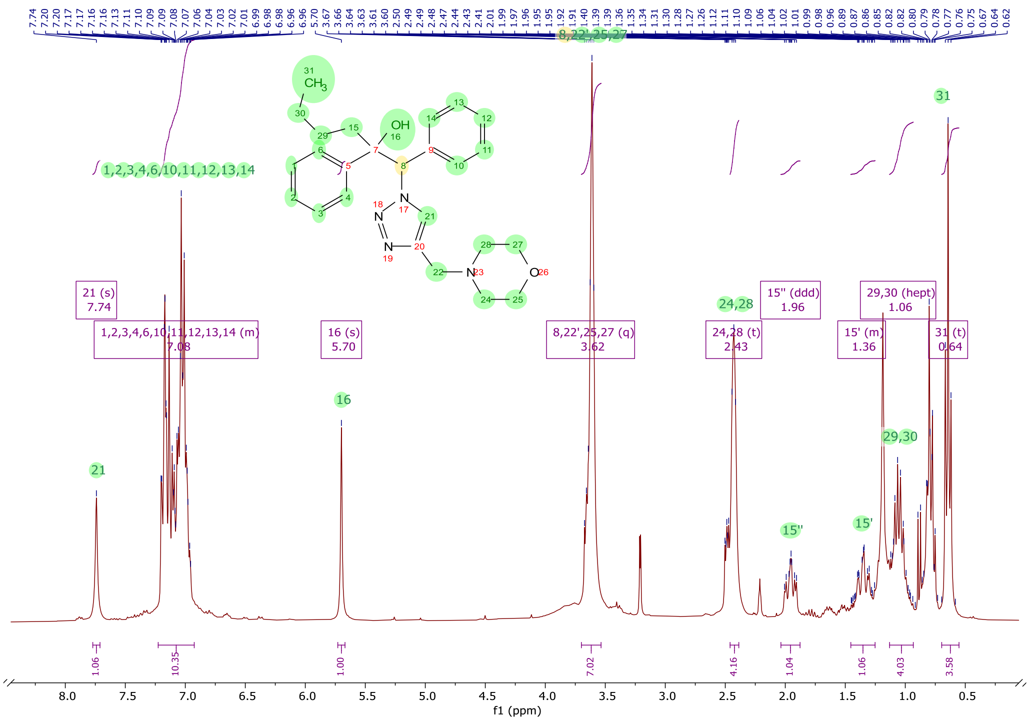

^{13}C NMR spectrum

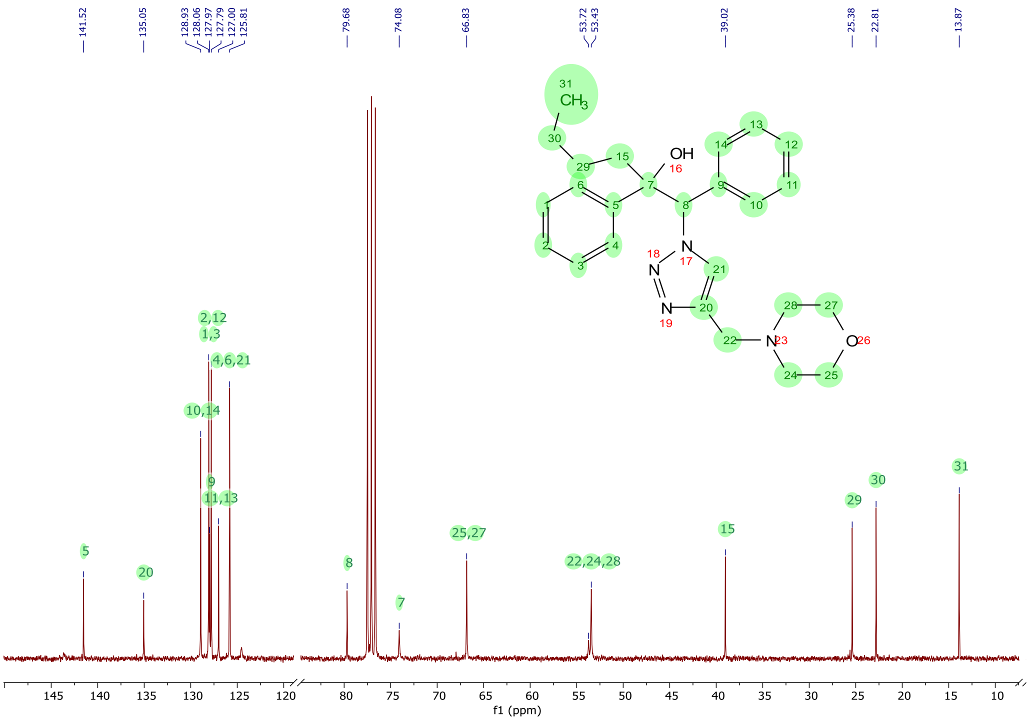

1,2,3-Triphenyl-1-(4-phenyl-[1,2,3]triazol-1-yl)-propan-2-ol (21)

^{1}H NMR spectrum

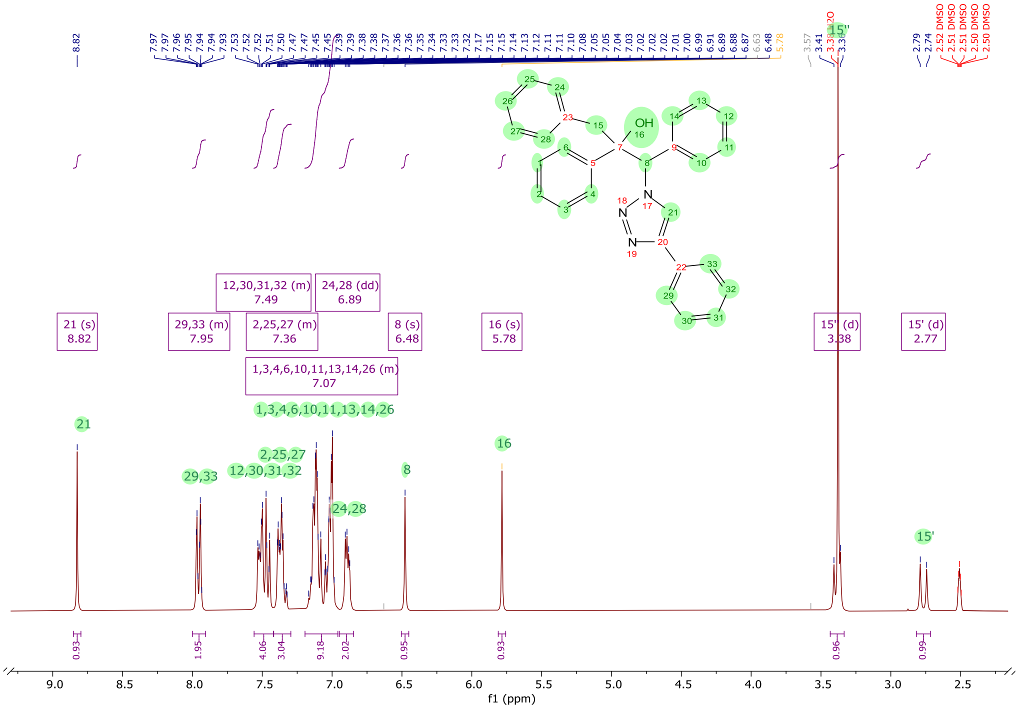

^{13}C NMR spectrum

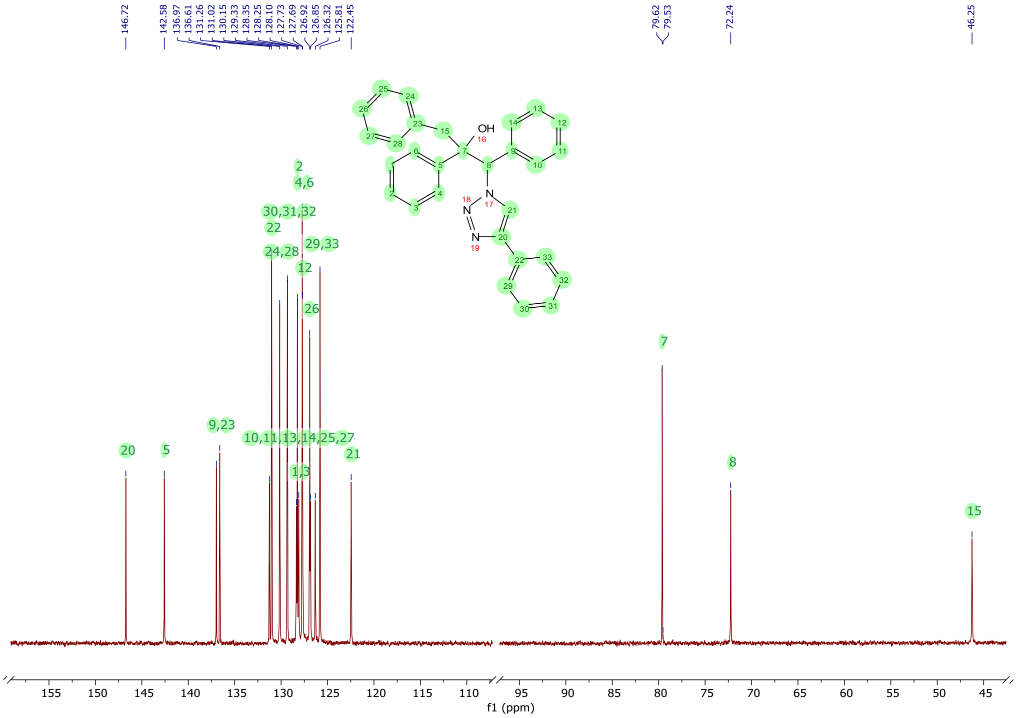

1-(4-Cyclopropyl-[1,2,3]triazol-1-yl)-1,2,3-triphenyl-propan-2-ol (22)

^{1}H NMR spectrum

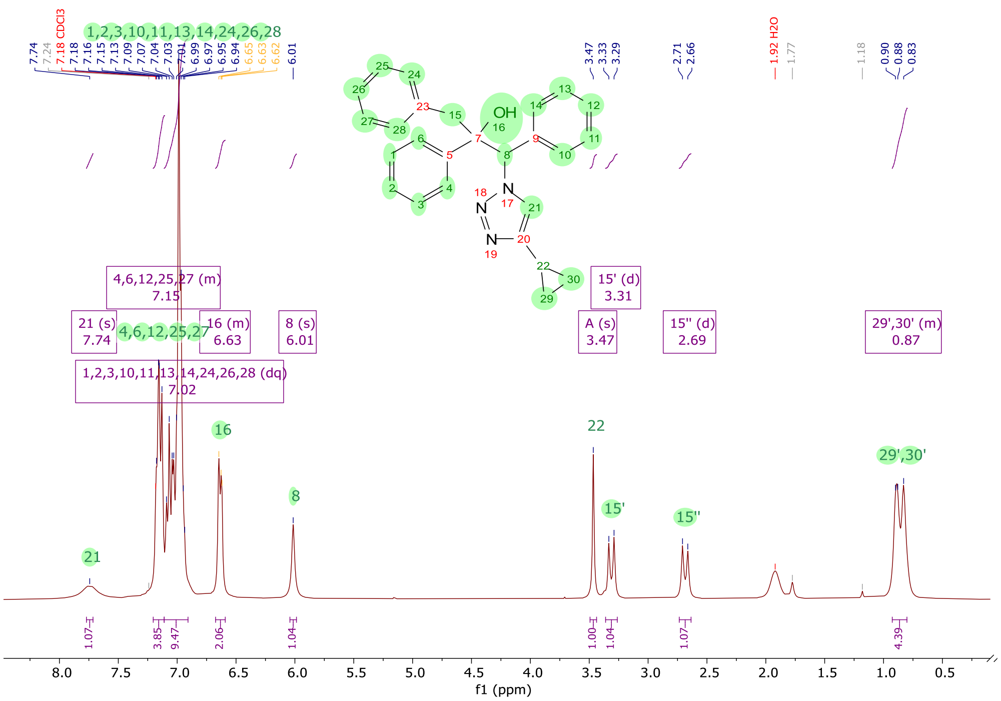

^{13}C NMR spectrum

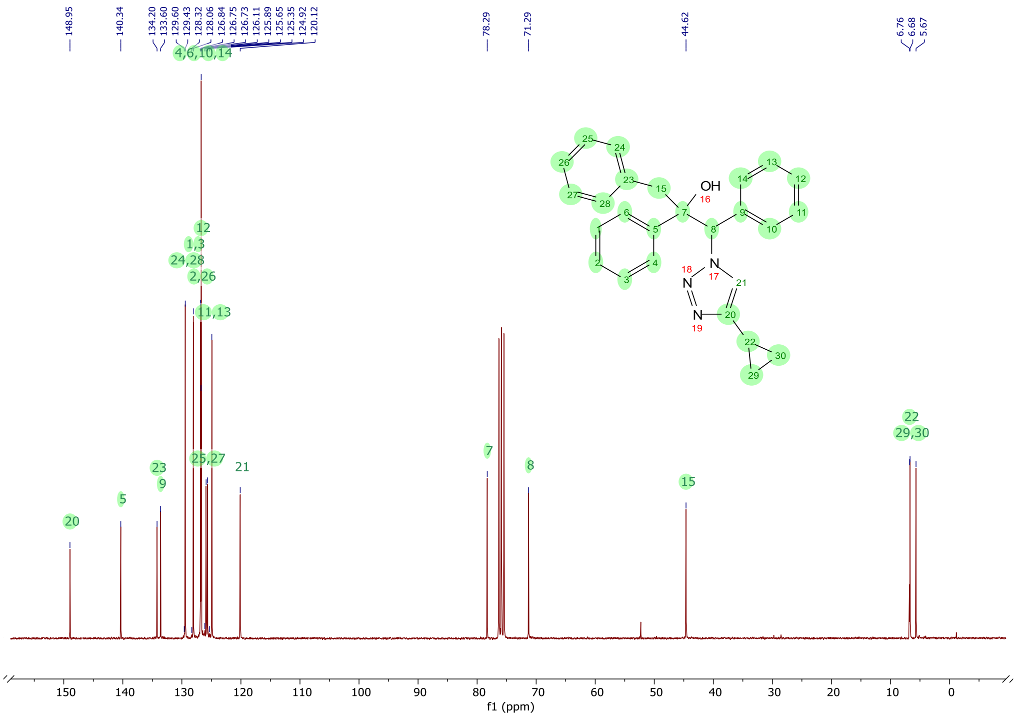

2-[1-(2-Hydroxy-1,2,3-triphenyl-propyl)-[1,2,3]triazol-4-ylmethyl]-isoindole-1,3-dione (23)

^{1}H NMR spectrum

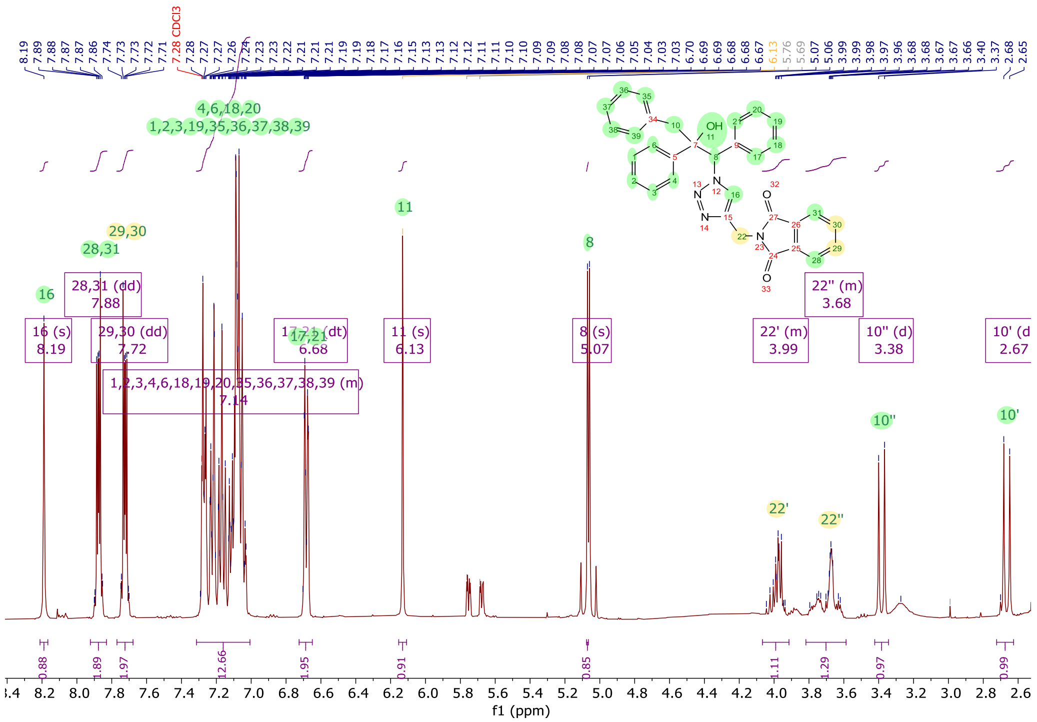

^{13}C NMR spectrum

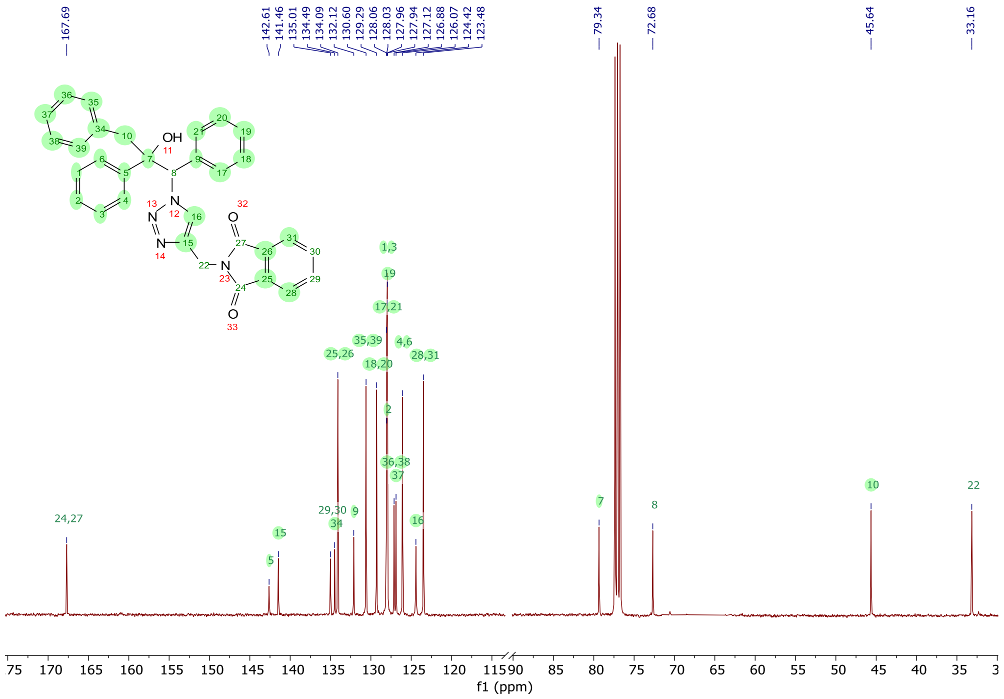

1-[4-(2,6-Diisopropyl-phenoxymethyl)-[1,2,3]triazol-1-yl]-1,2,3-triphenyl-propan-2-ol (24)

^{1}H NMR spectrum

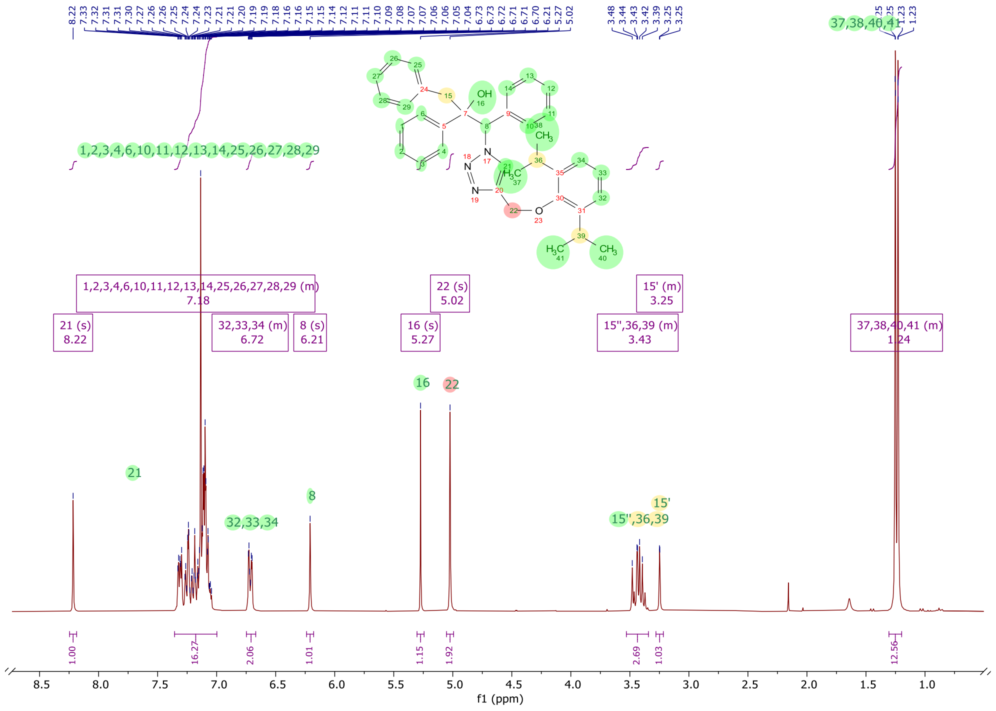

^{13}C NMR spectrum

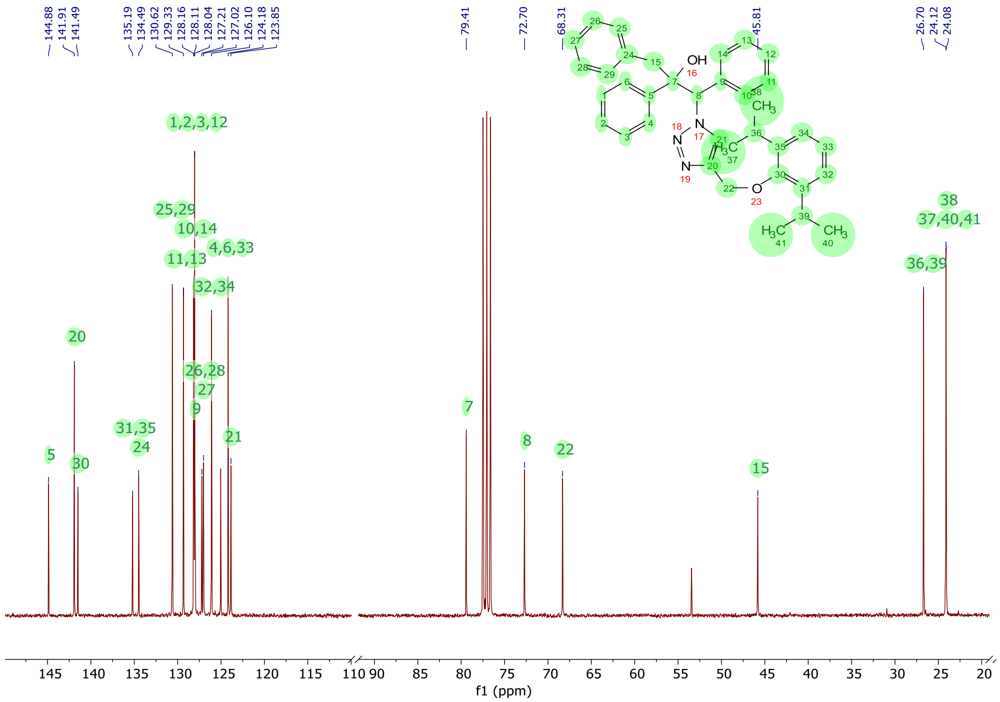

1-(4-Morpholin-4-ylmethyl-[1,2,3]triazol-1-yl)-1,2,3-triphenyl-propan-2-ol (25)

^{1}H NMR spectrum

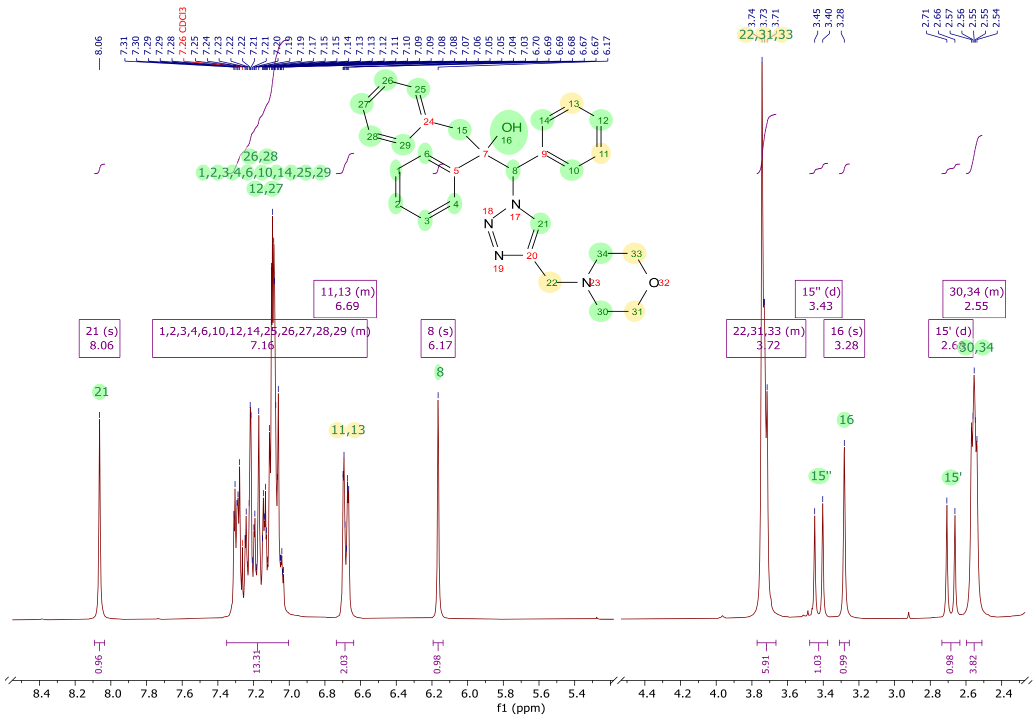

^{13}C NMR spectrum

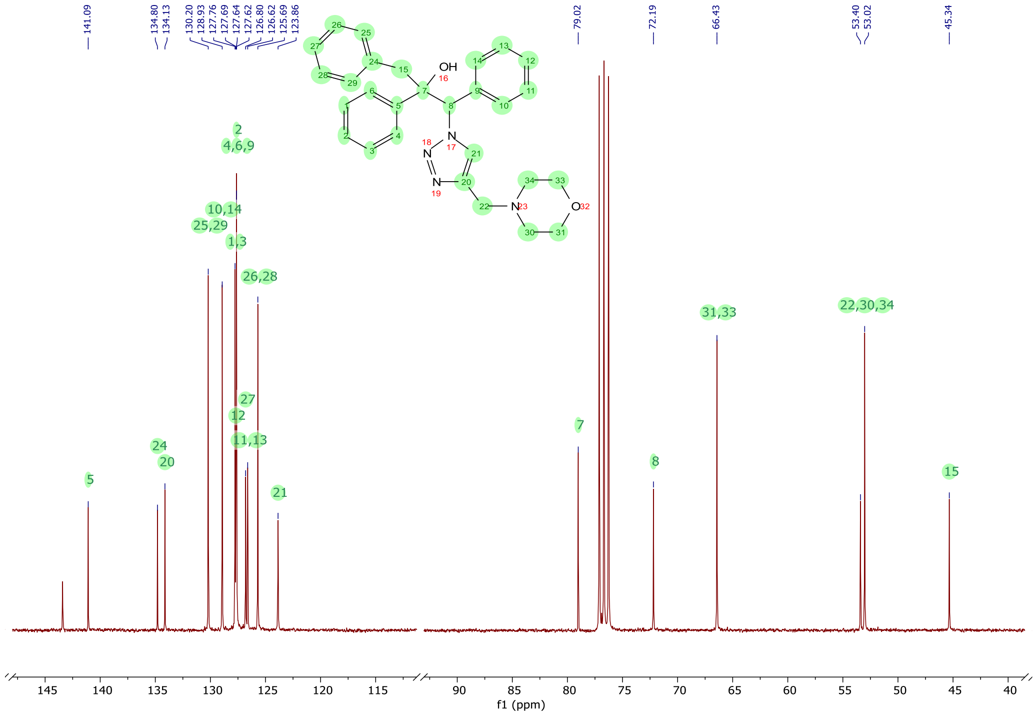

1-Cyclohexyl-1,2-diphenyl-2-(4-phenyl-[1,2,3]triazol-1-yl)-ethanol (26)

^{1}H NMR spectrum

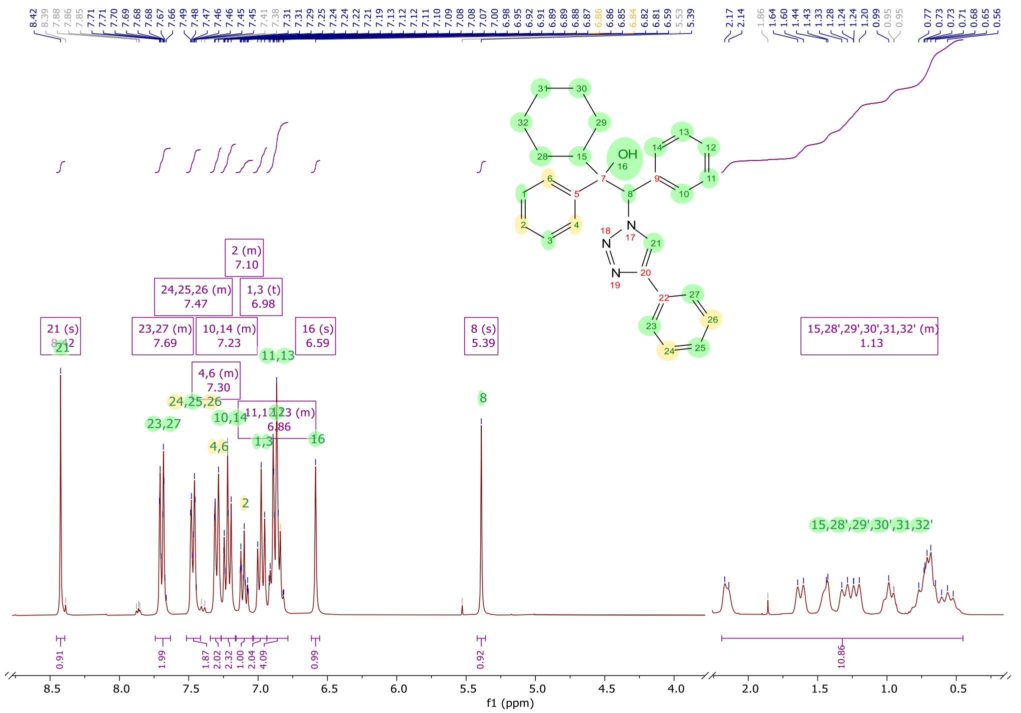

^{13}C NMR spectrum

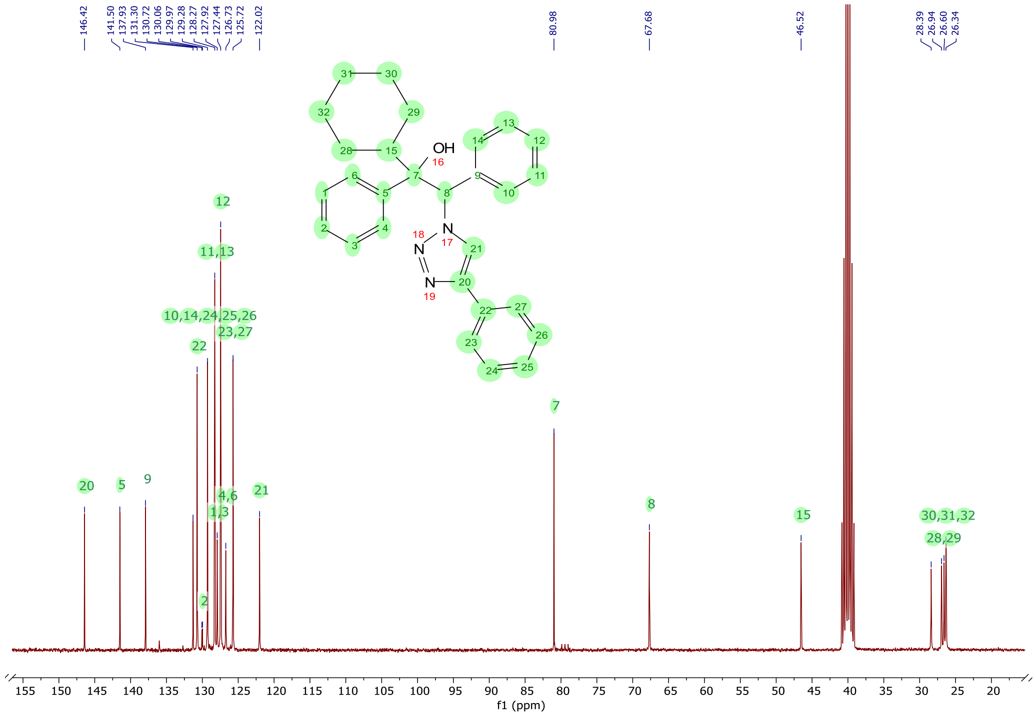

1-Cyclohexyl-1,2-diphenyl-2-(4-phenyl-[1,2,3]triazol-1-yl)-ethanol (27)

^{1}H NMR spectrum

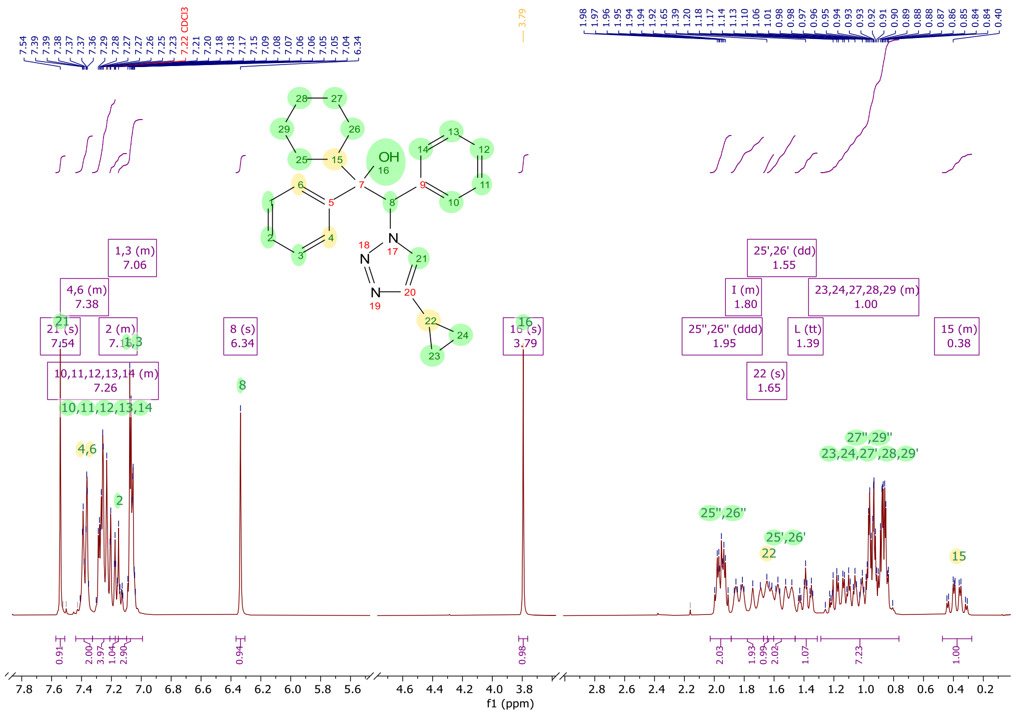

^{13}C NMR spectrum

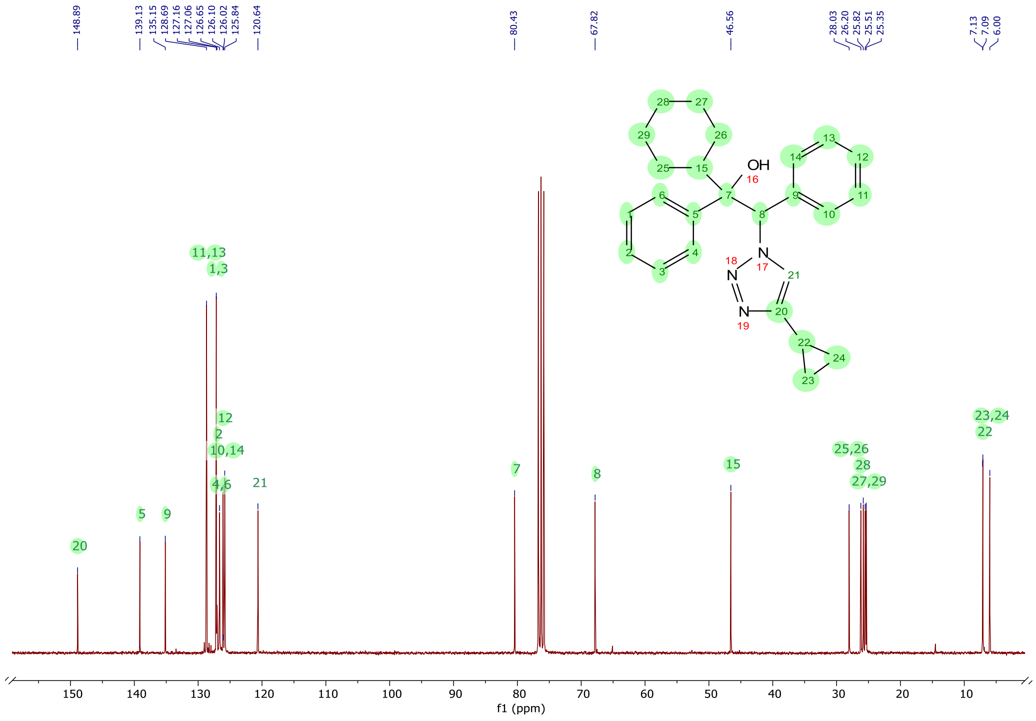

2-[1-(2-Cyclohexyl-2-hydroxy-1,2-diphenyl-ethyl)-[1,2,3]triazol-4-ylmethyl]-isoindole-1,3-dione (28)

^{1}H NMR spectrum

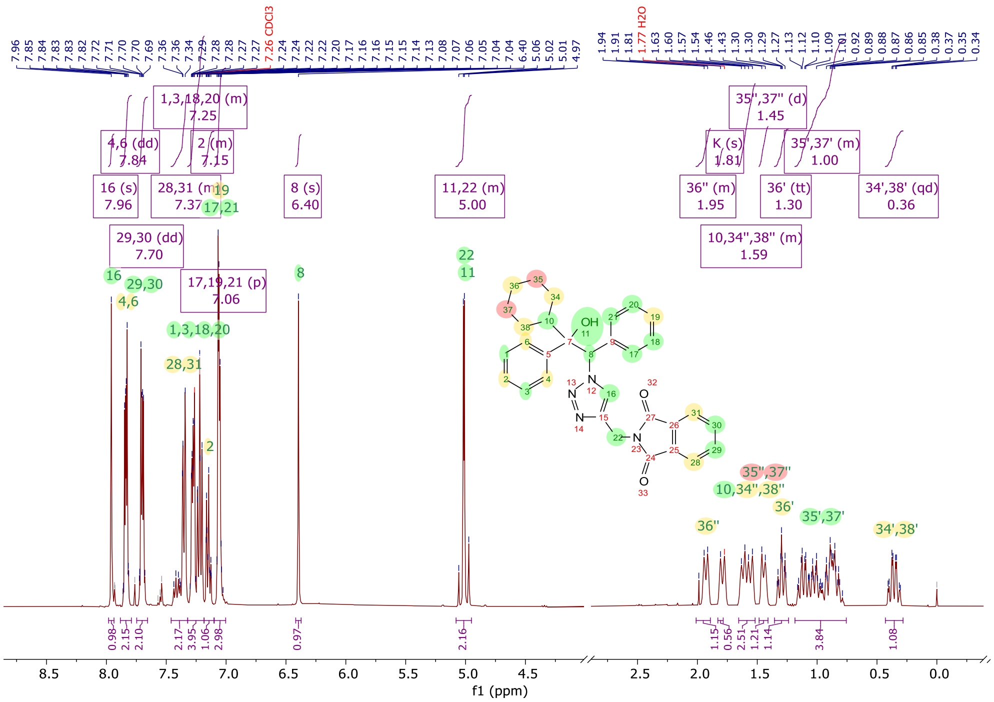

^{13}C NMR spectrum

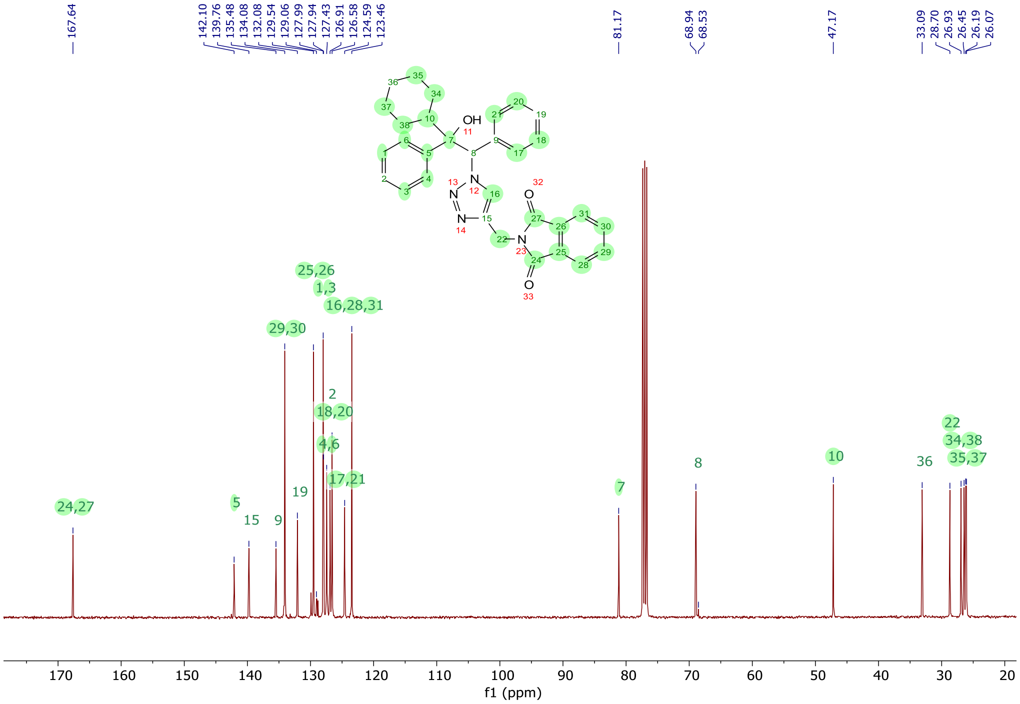

1-Cyclohexyl-2-[4-(2,6-diisopropyl-phenoxymethyl)-[1,2,3]triazol-1-yl]-1,2-diphenyl-ethanol (29)

^{1}H NMR spectrum

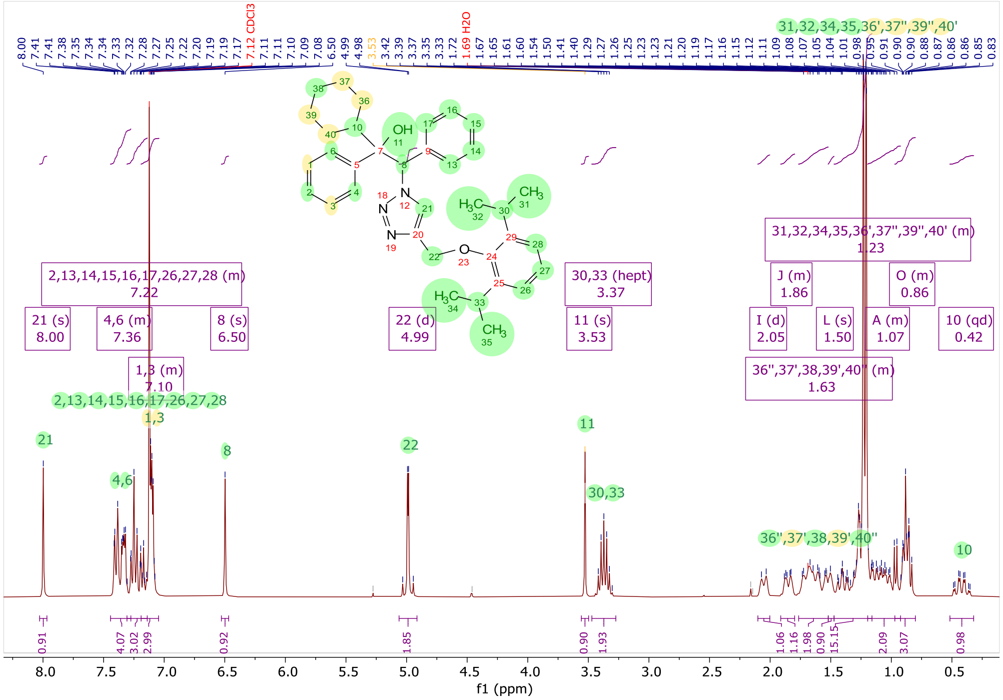

^{13}C NMR spectrum

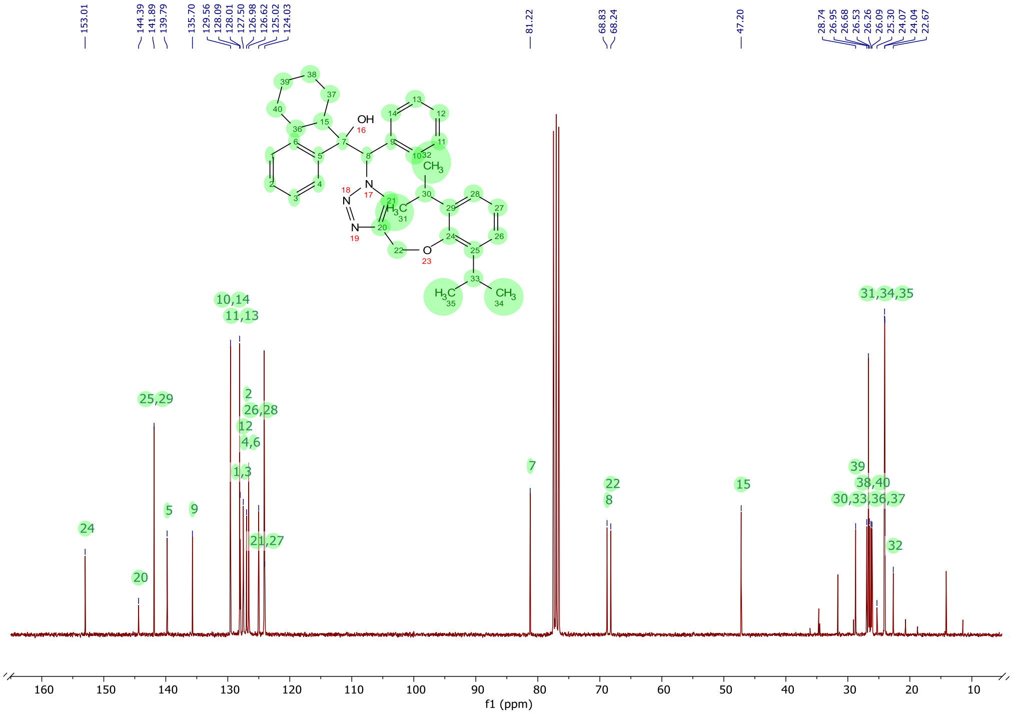

1-Cyclohexyl-2-(4-morpholin-4-ylmethyl-[1,2,3]triazol-1-yl)-1,2-diphenyl-ethanol (30)

^{1}H NMR spectrum

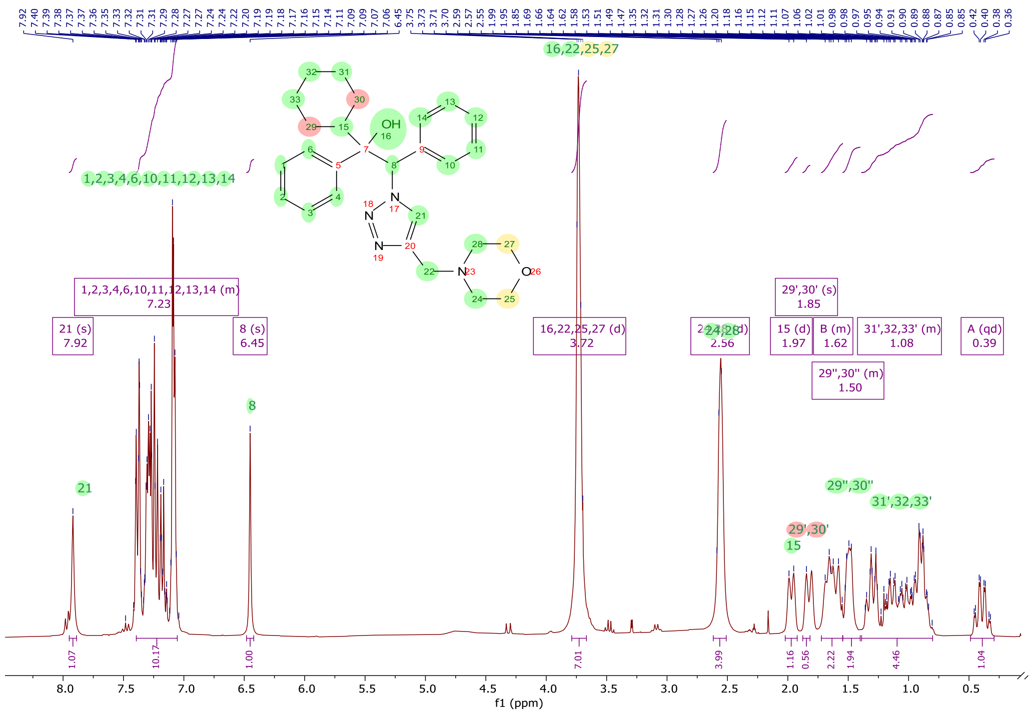

^{13}C NMR spectrum

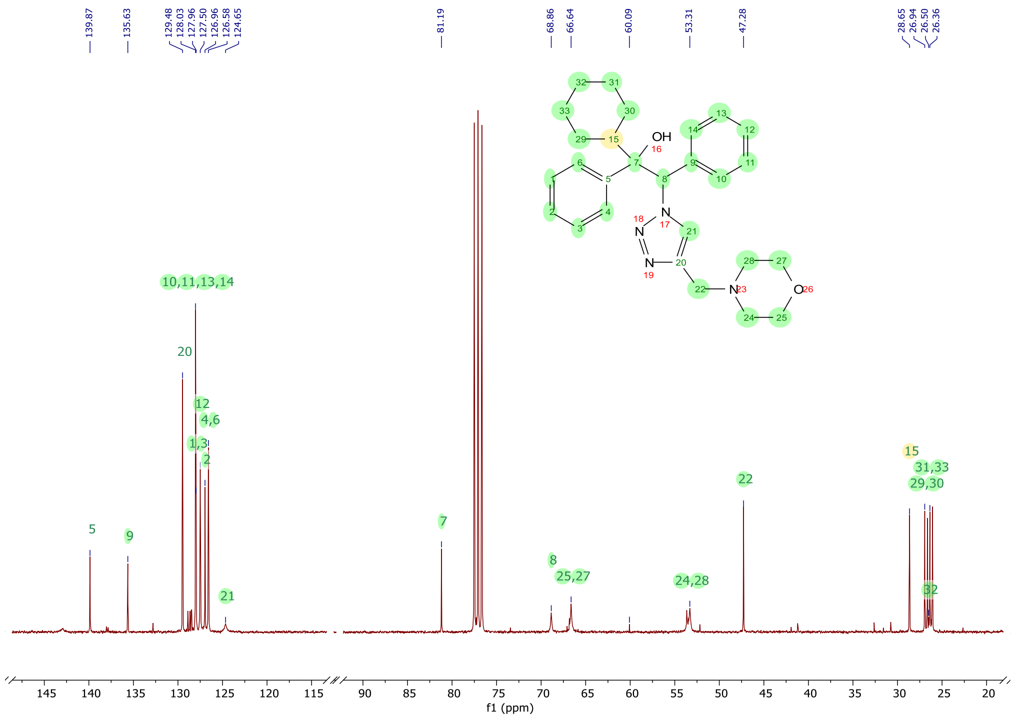

1,2-Bis-(4-methoxyphenyl)-1-(4-phenyl-[1,2,3]triazol-1-yl)-butan-2-ol (31)

^{1}H NMR spectrum

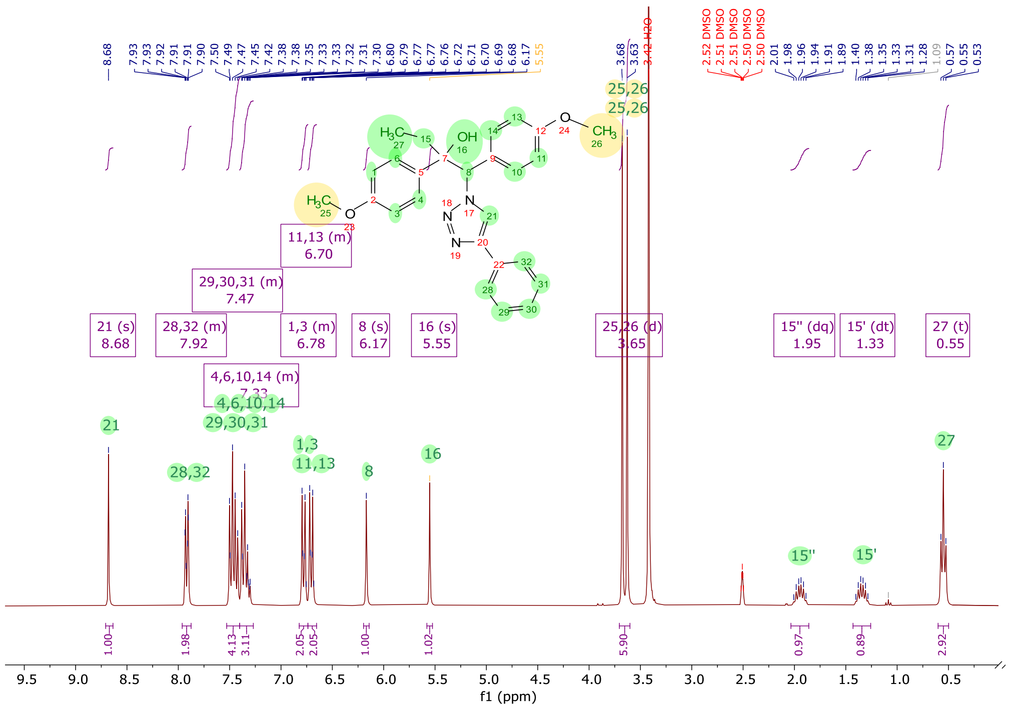

^{13}C NMR spectrum

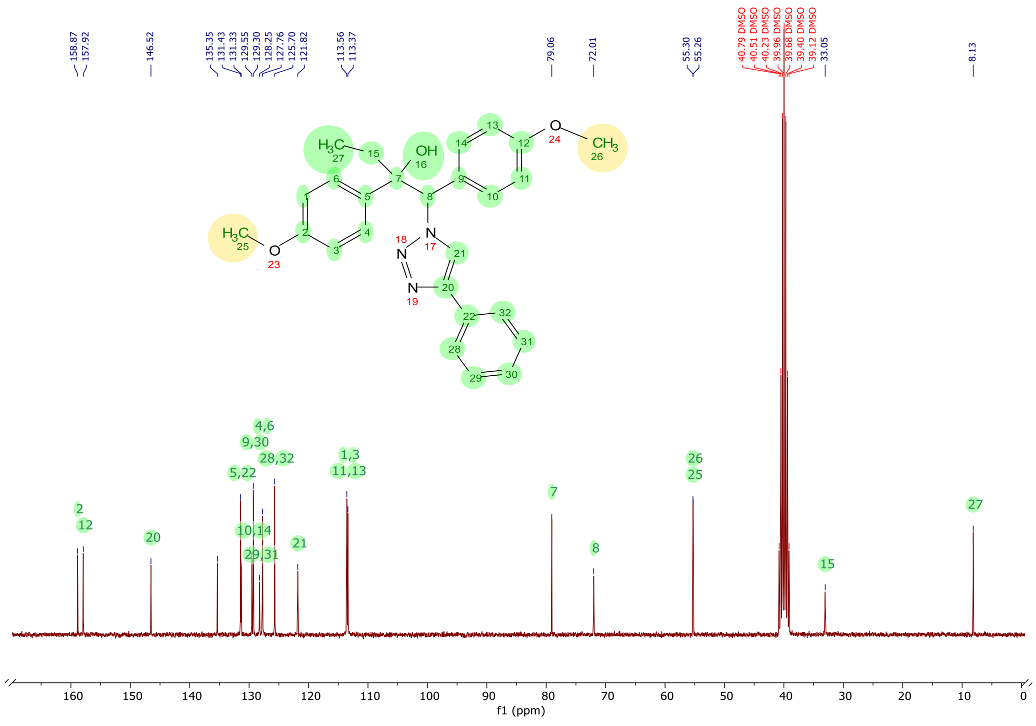

1-(4-Cyclopropyl-[1,2,3]triazol-1-yl)-1,2-bis-(4-methoxyphenyl)-butan-2-ol (32)

^{1}H NMR spectrum

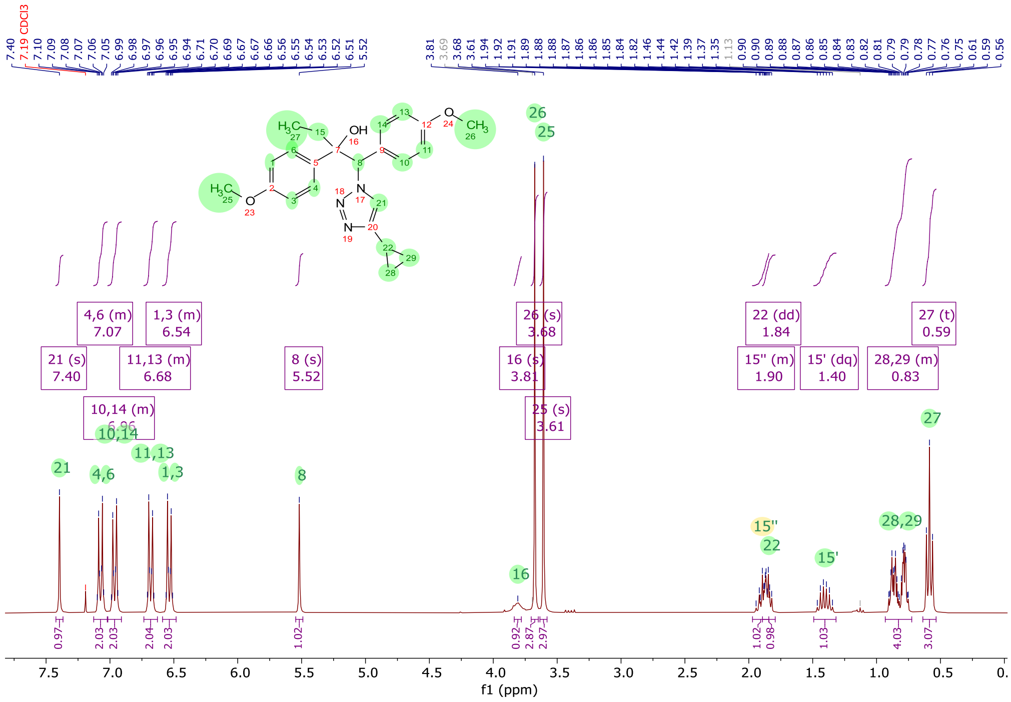

^{13}C NMR spectrum

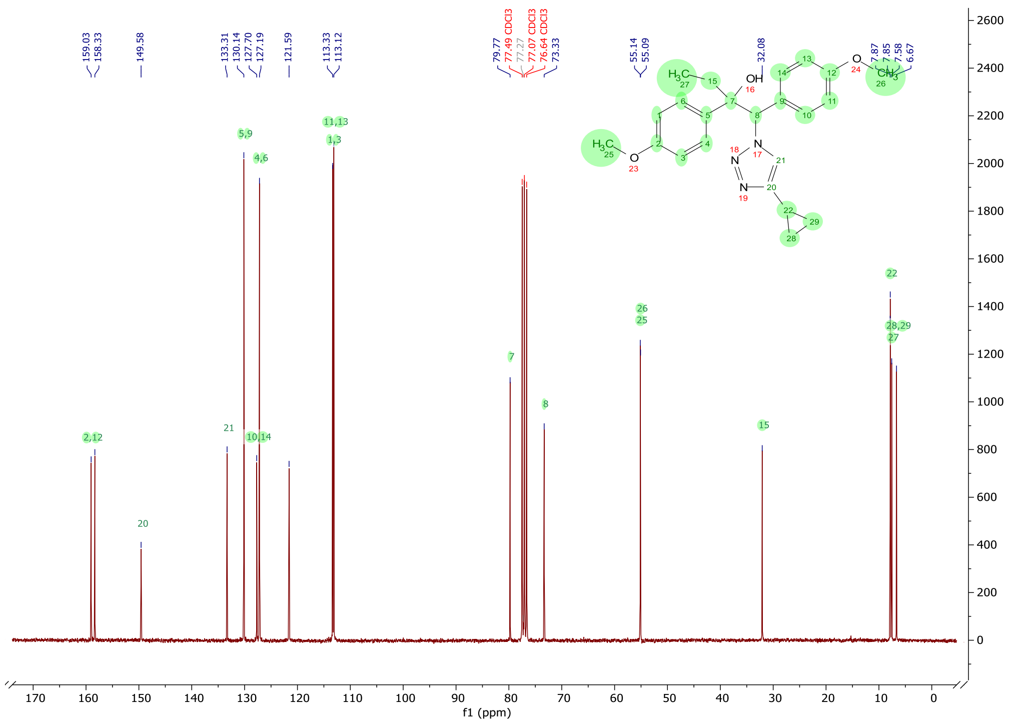

2-{1-[2-Hydroxy-1,2-bis-(4-methoxy-phenyl)-butyl]-[1,2,3]triazol-4-ylmethyl}-isoindole-1,3-dione (33)

^{1}H NMR spectrum

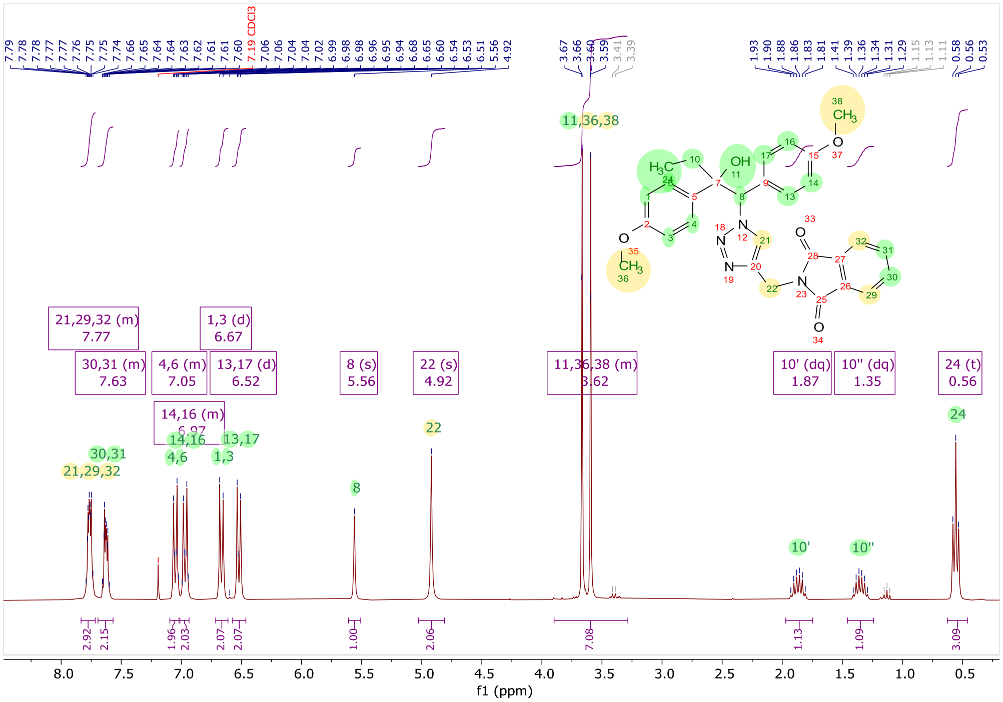

^{13}C NMR spectrum

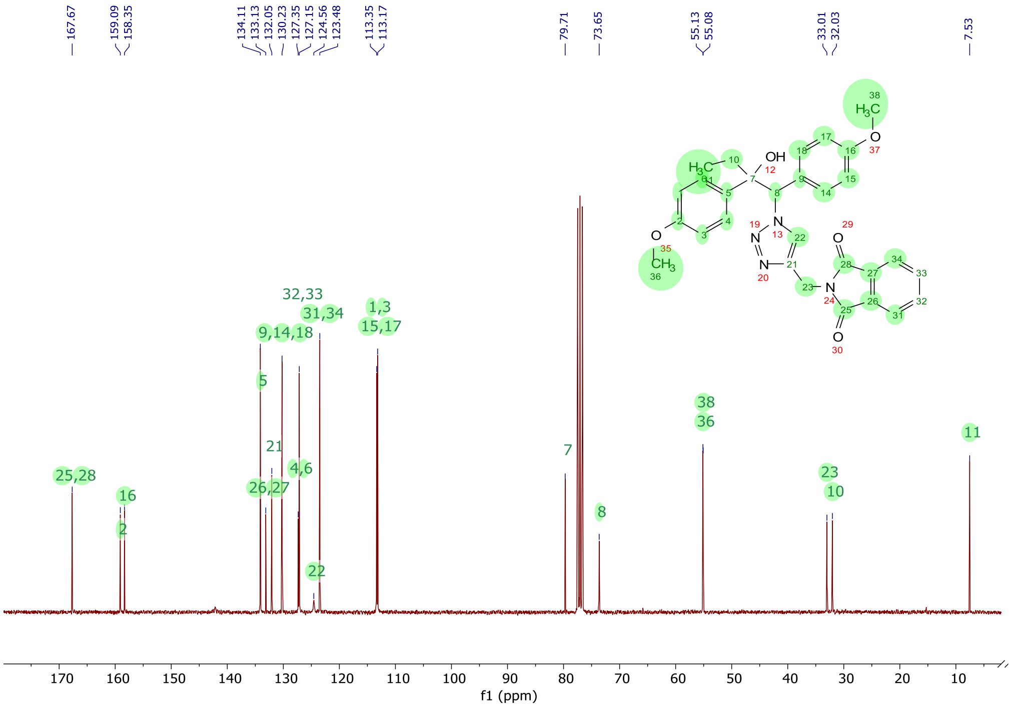

1-[4-(2,6-Diisopropylphenoxymethyl)-[1,2,3]triazol-1-yl]-1,2-bis-(4-methoxy-phenyl)-butan-2-ol (34)

^{1}H NMR spectrum

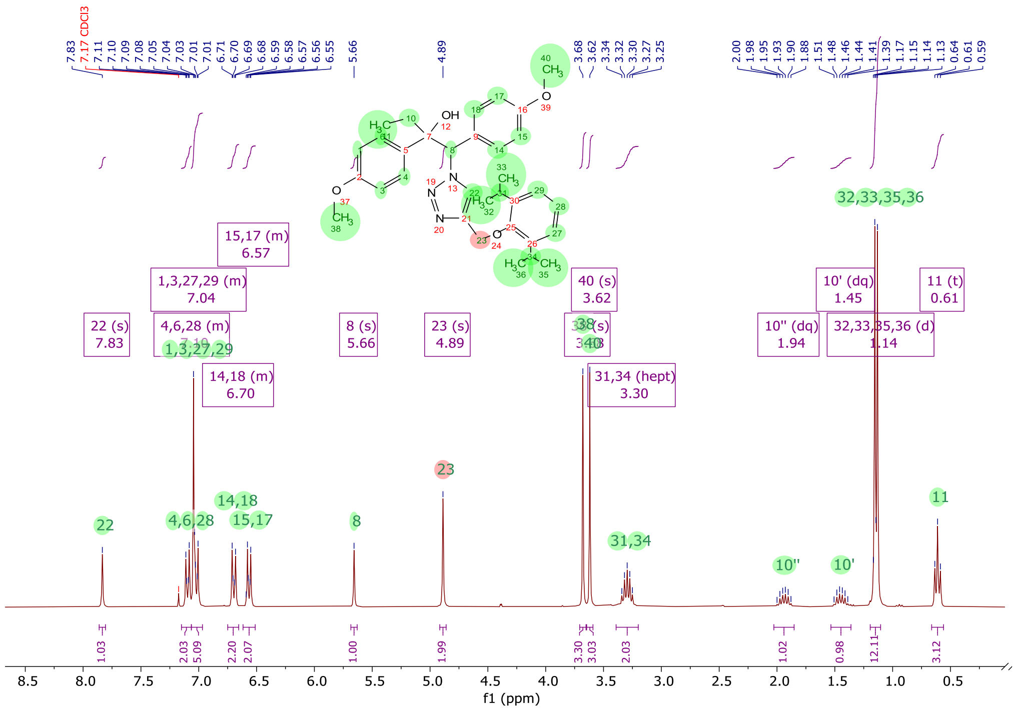

^{13}C NMR spectrum

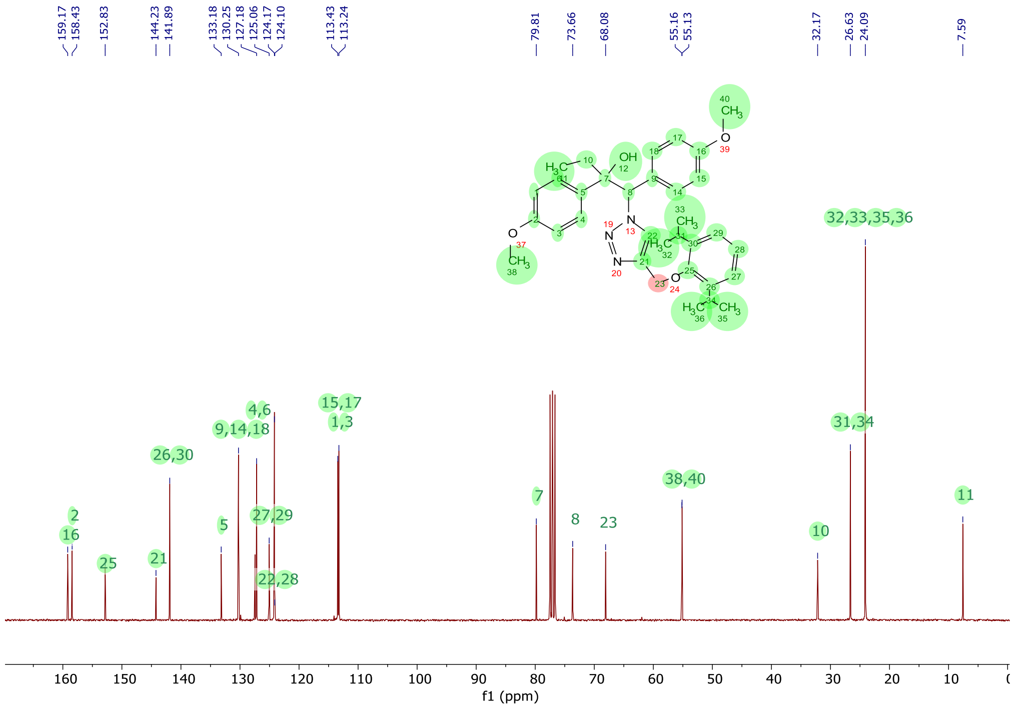

1-(4-Morpholin-4-ylmethyl-[1,2,3]triazol-1-yl)-1,2-diphenyl-butan-2-ol (35)

^{1}H NMR spectrum

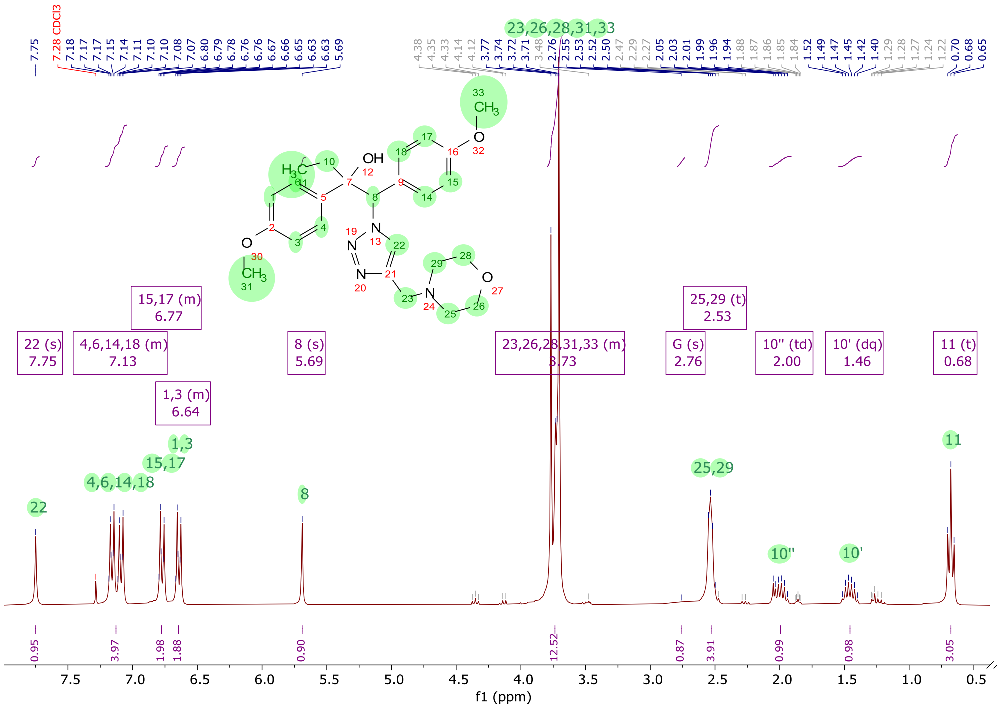

^{13}C NMR spectrum

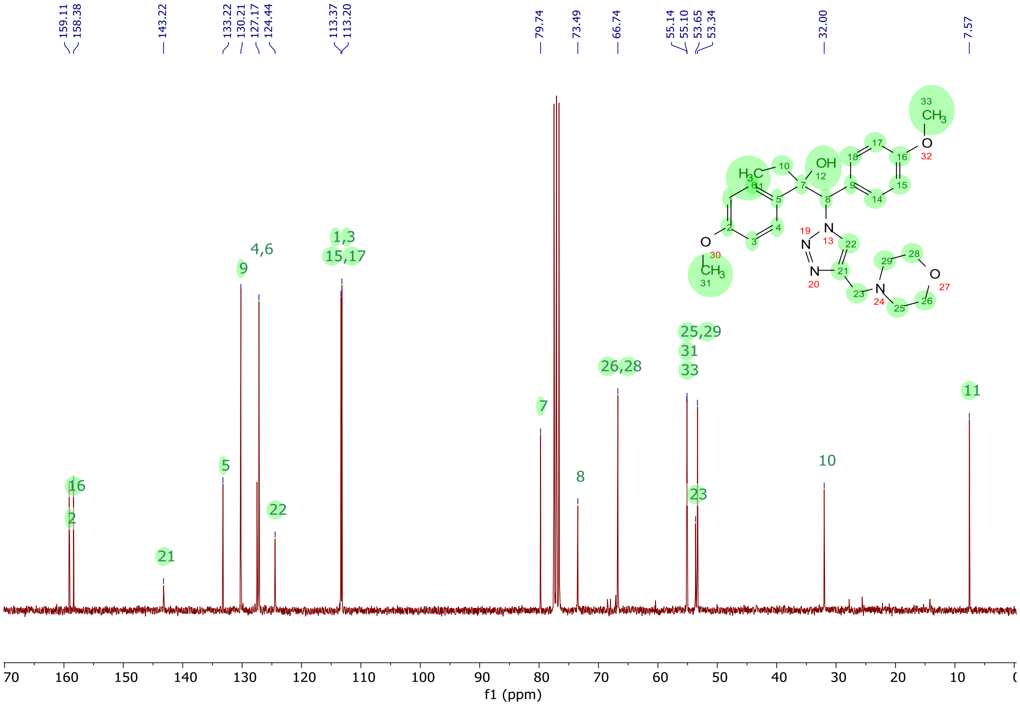

1,2-Bis-(4-methoxyphenyl)-1-(4-phenyl-[1,2,3]triazol-1-yl)-hexan-2-ol (36)

^{1}H NMR spectrum

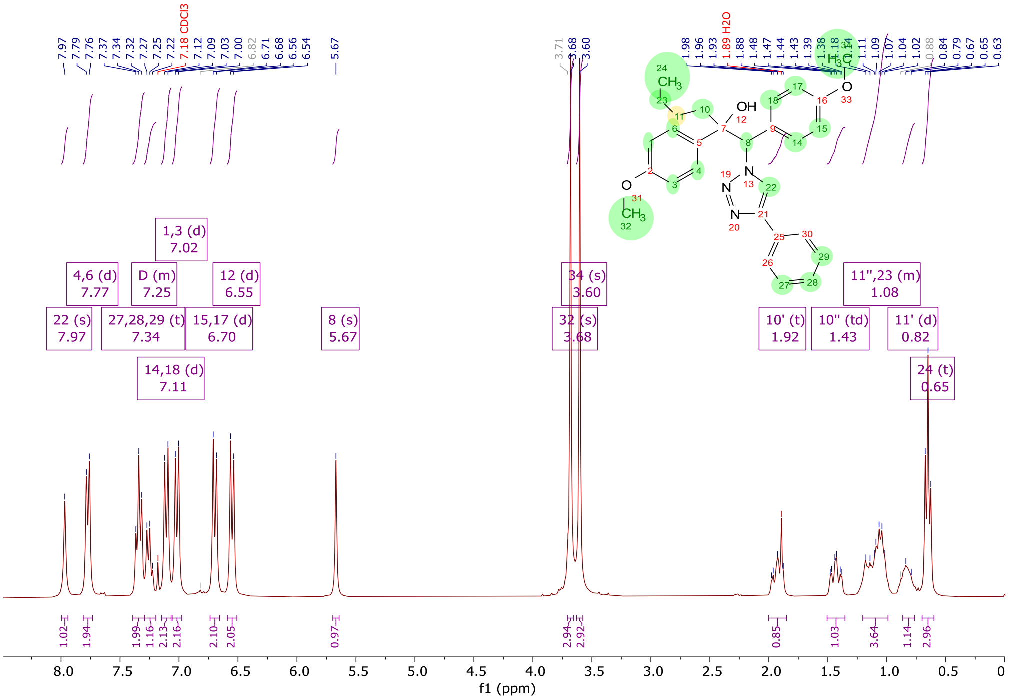

^{13}C NMR spectrum

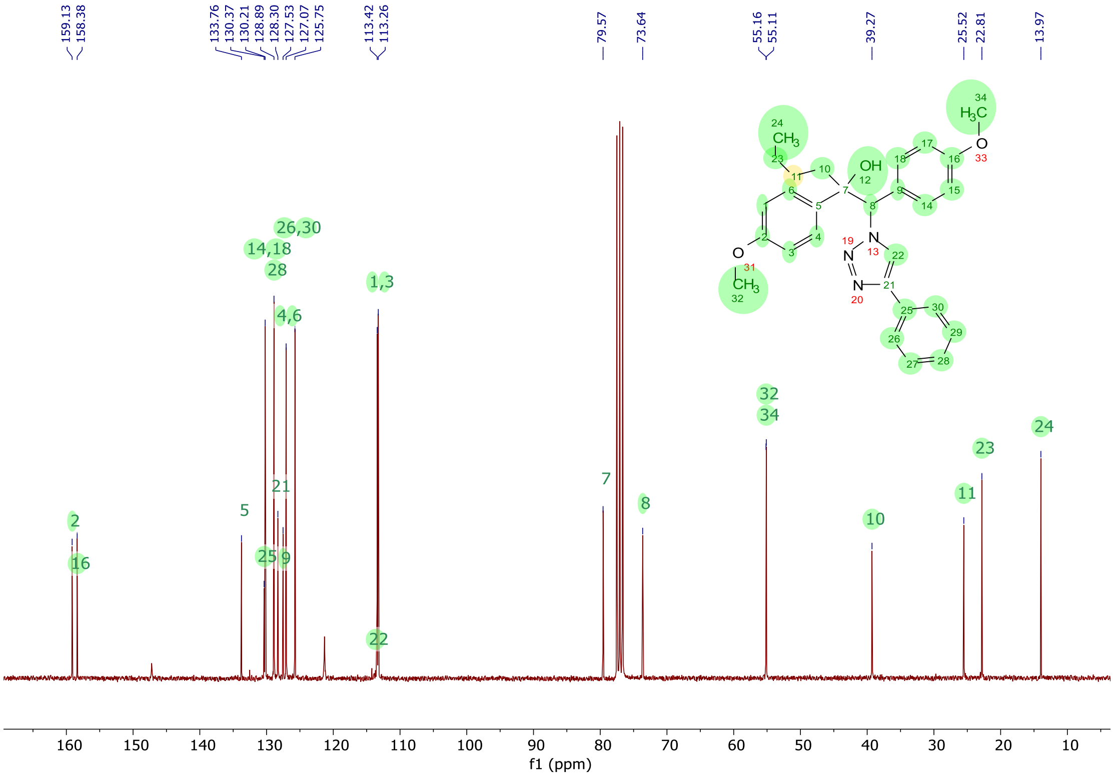

1-(4-Cyclopropyl-[1,2,3]triazol-1-yl)-1,2-bis-(4-methoxyphenyl)-hexan-2-ol (37)

^{1}H NMR spectrum

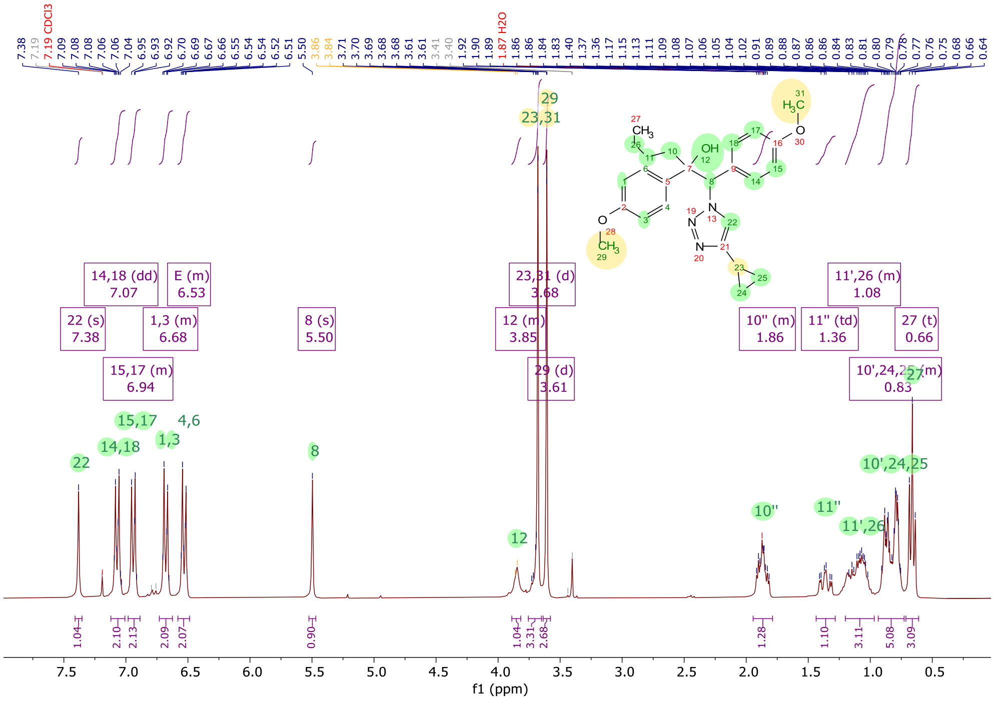

^{13}C NMR spectrum

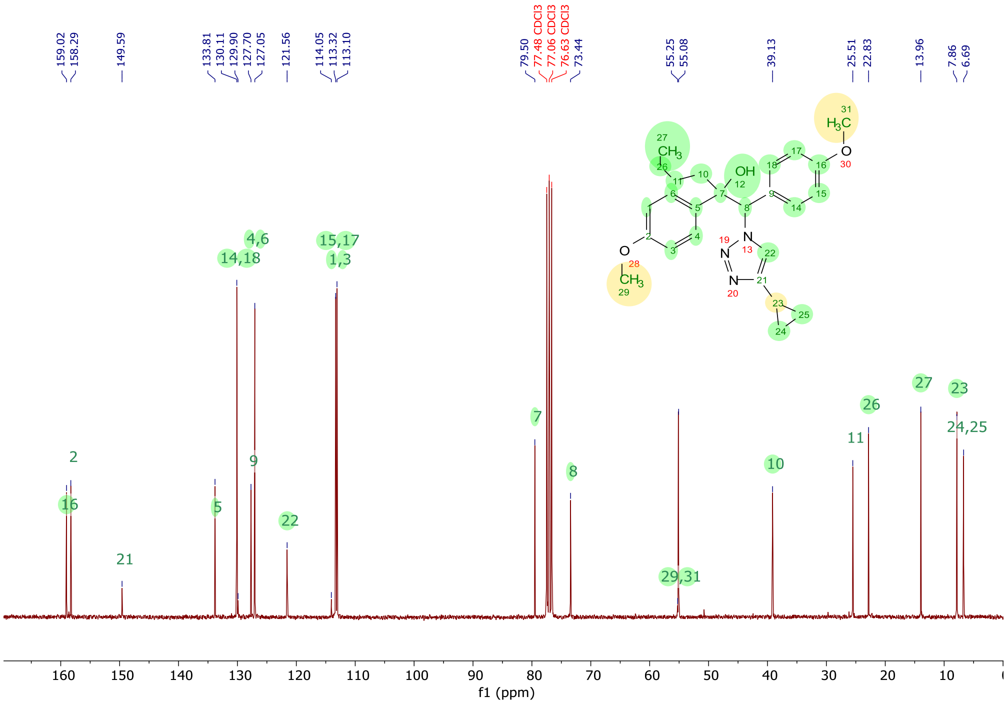

2-{1-[2-Hydroxy-1,2-bis-(4-methoxyphenyl)-hexyl]-[1,2,3]triazol-4-ylmethyl}-isoindole-1,3-dione (38)

^{1}H NMR spectrum

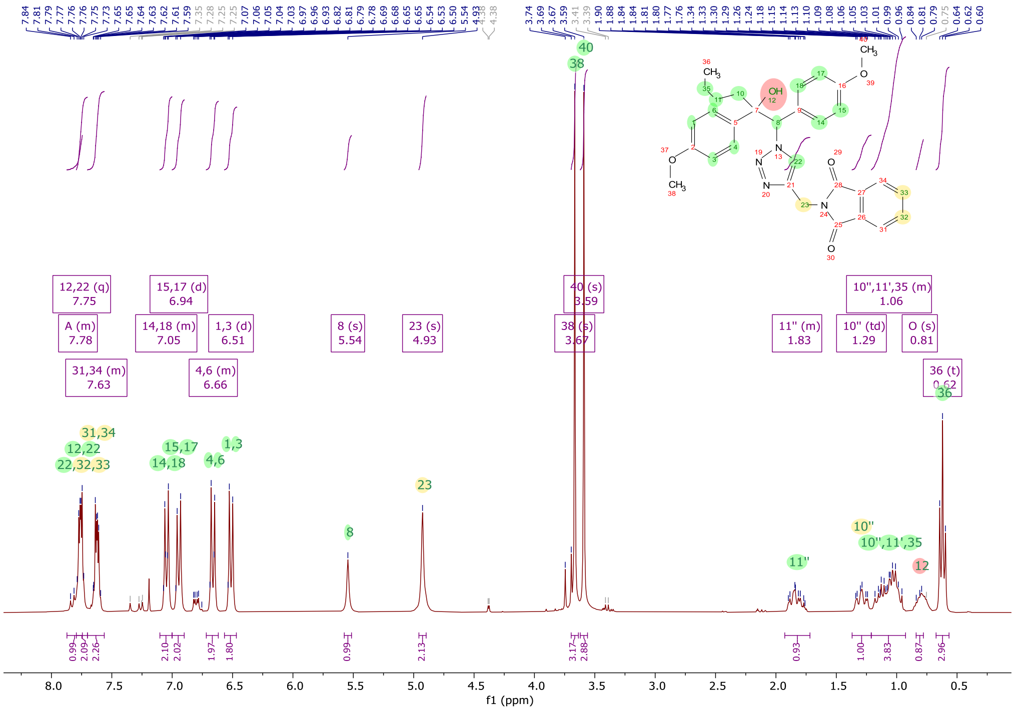

^{13}C NMR spectrum

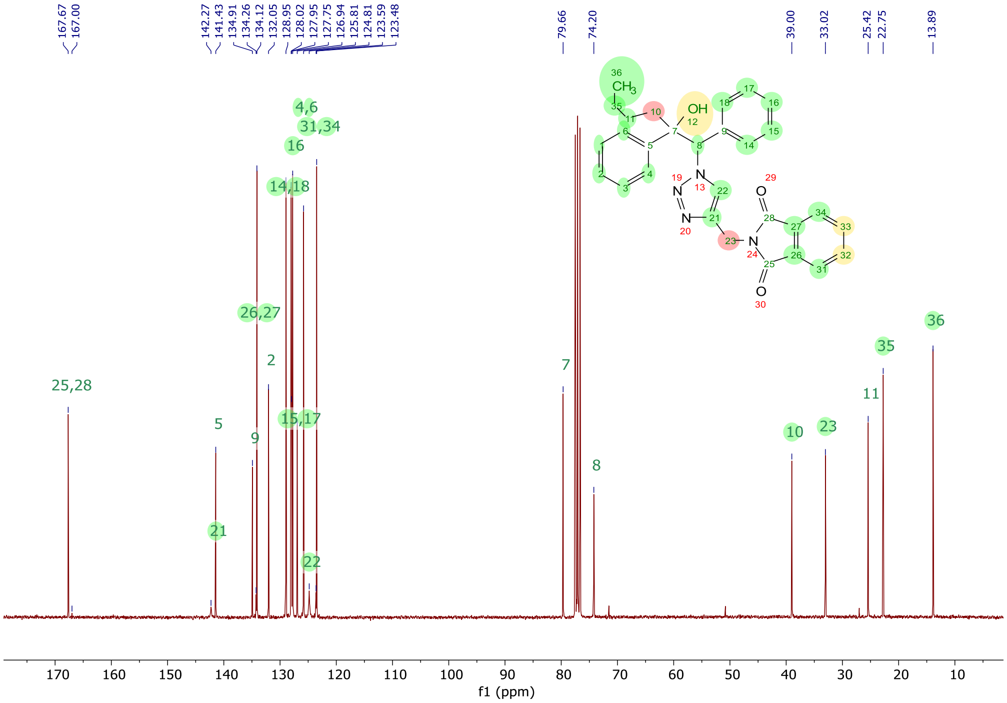

1-[4-(2,6-Diisopropylphenoxymethyl)-[1,2,3]triazol-1-yl]-1,2-bis-(4-methoxyphenyl)-hexan-2-ol (39)

^{1}H NMR spectrum

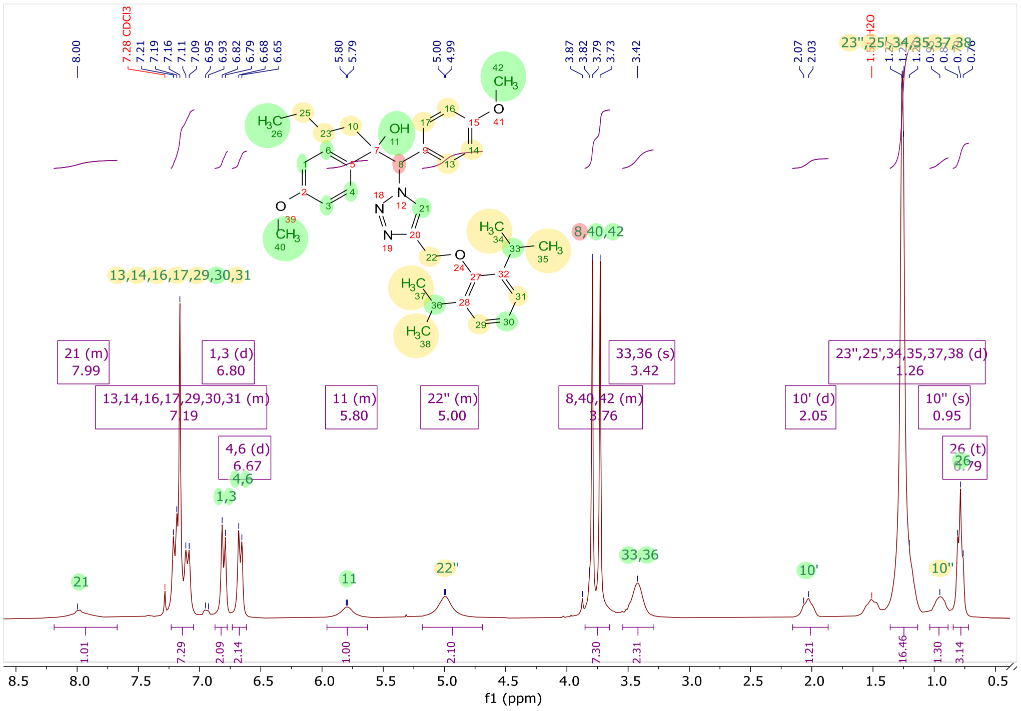

^{13}C NMR spectrum

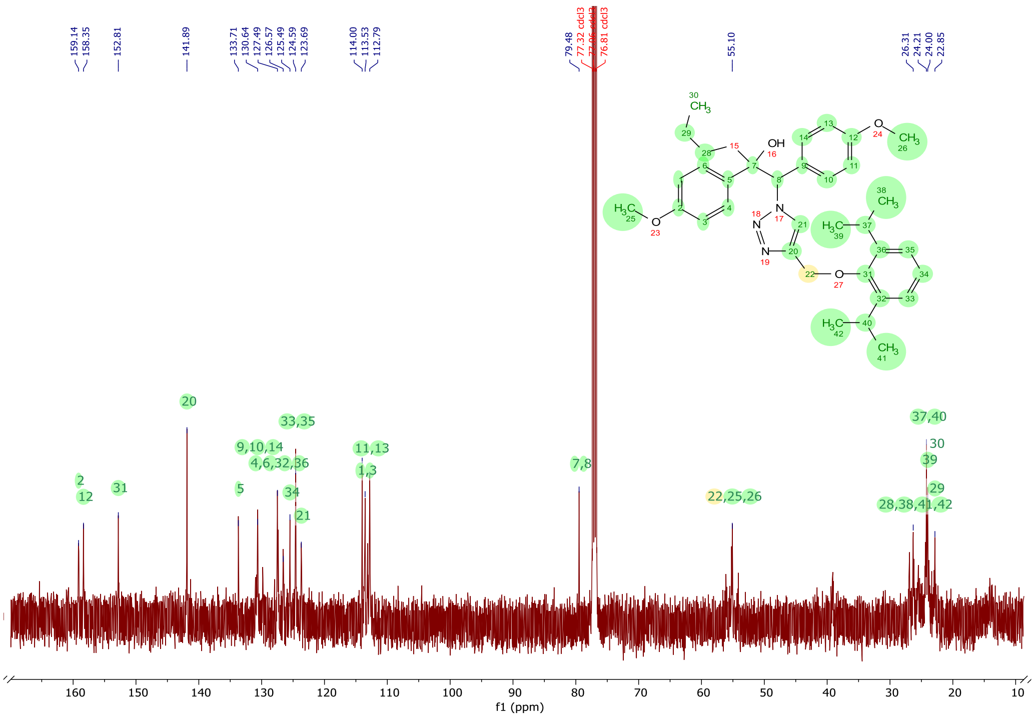

1,2-Bis-(4-methoxyphenyl)-1-(4-morpholin-4-ylmethyl-[1,2,3]triazol-1-yl)-hexan-2-ol (40)

^{1}H NMR spectrum

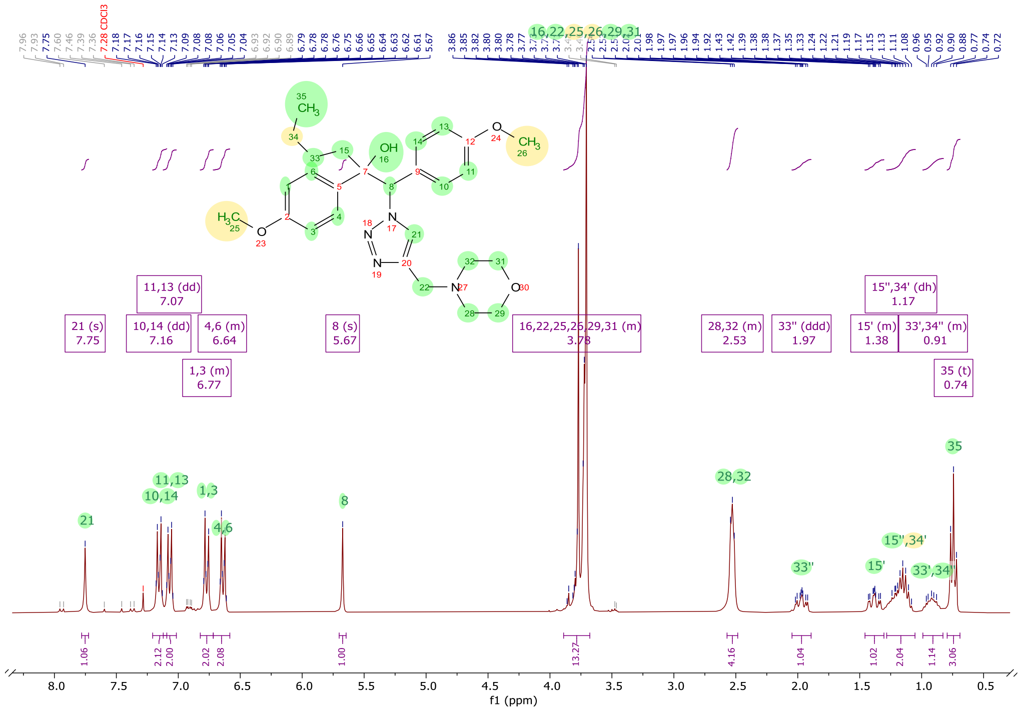

^{13}C NMR spectrum

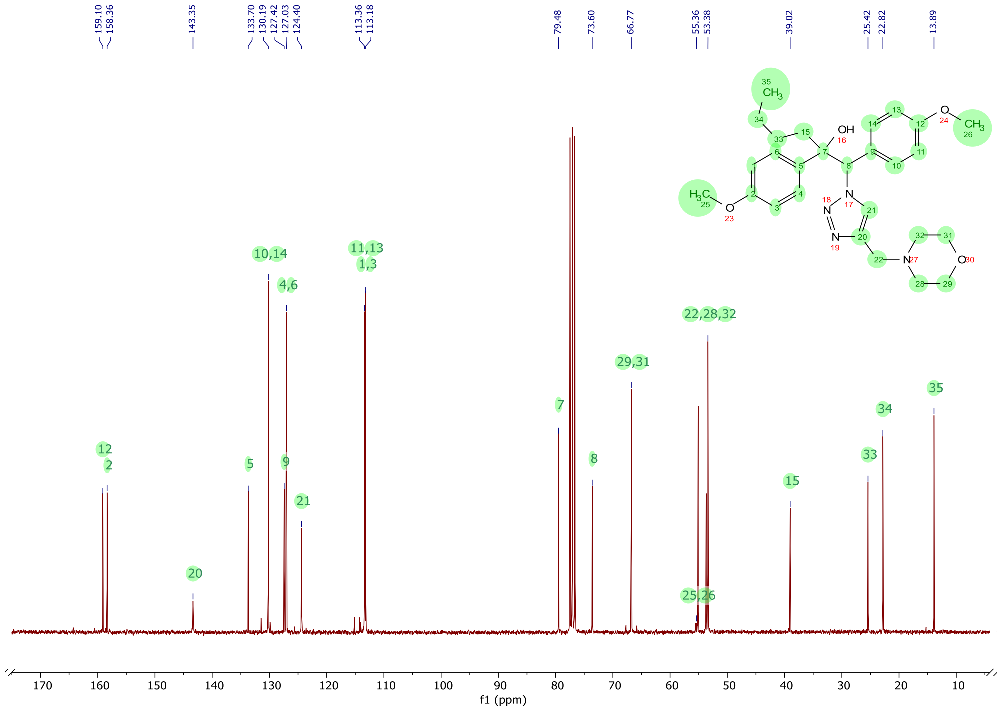

1,2-Bis-(4-methoxyphenyl)-3-phenyl-1-(4-phenyl-[1,2,3]triazol-1-yl)-propan-2-ol (41)

^{1}H NMR spectrum

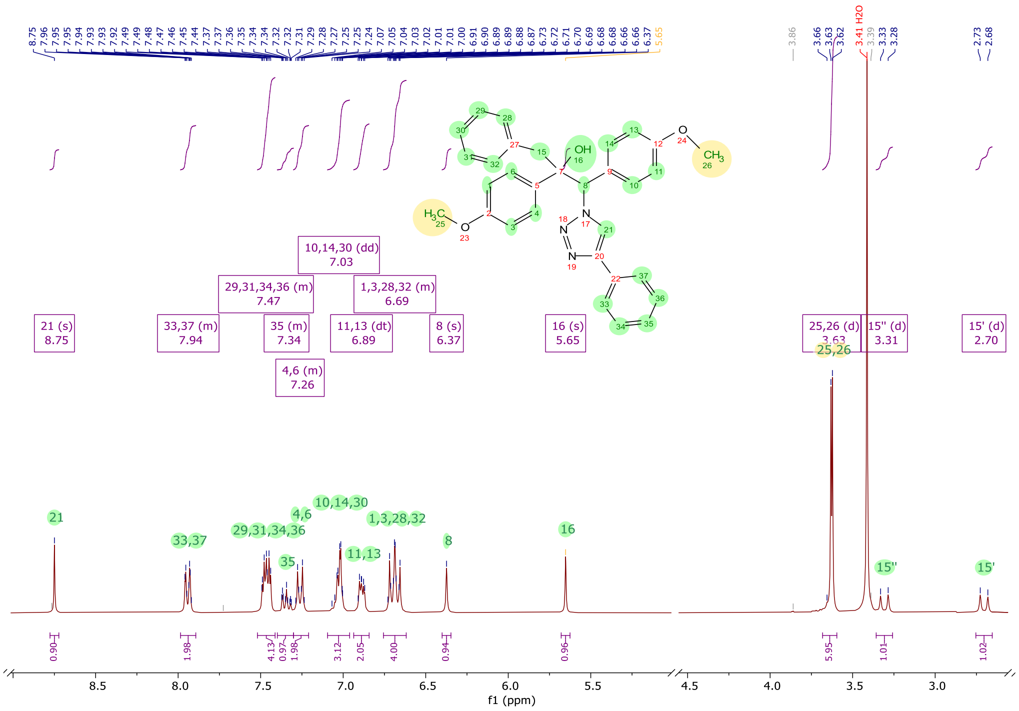

^{13}C NMR spectrum

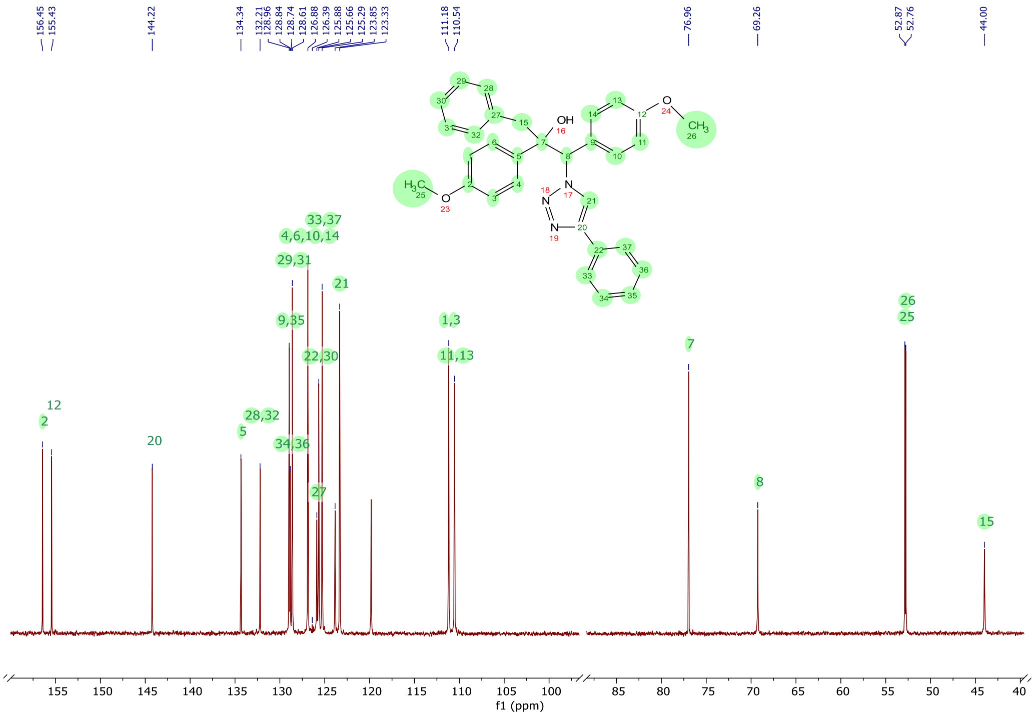

1-(4-Cyclopropyl-[1,2,3]triazol-1-yl)-1,2-bis-(4-methoxyphenyl)-3-phenyl-propan-2-ol (42)

^{1}H NMR spectrum

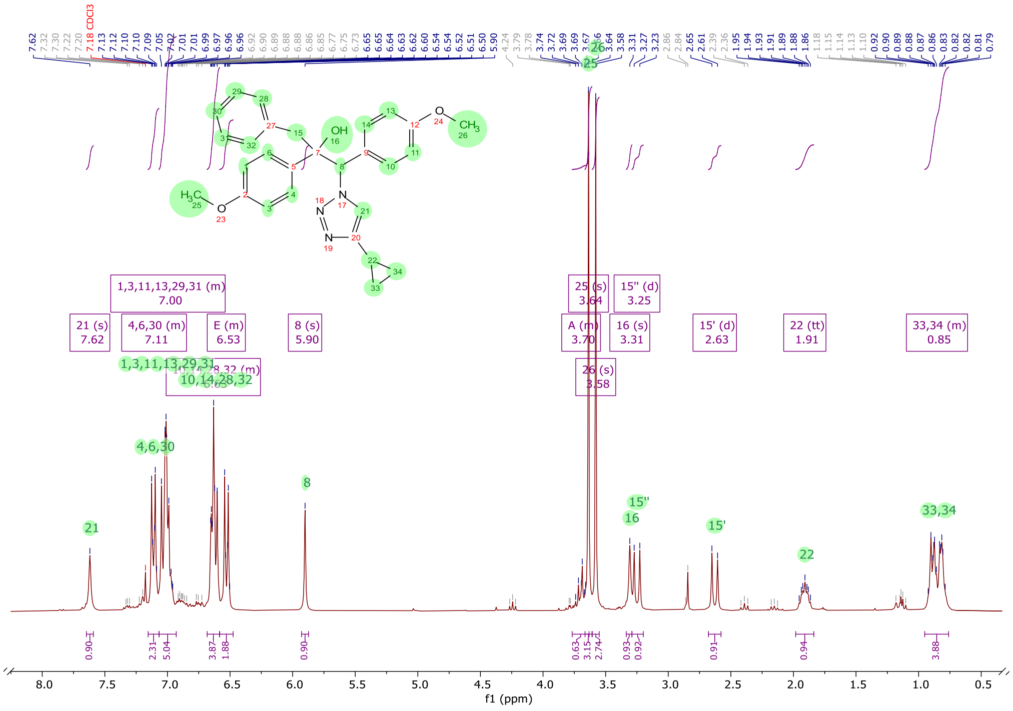

^{13}C NMR spectrum

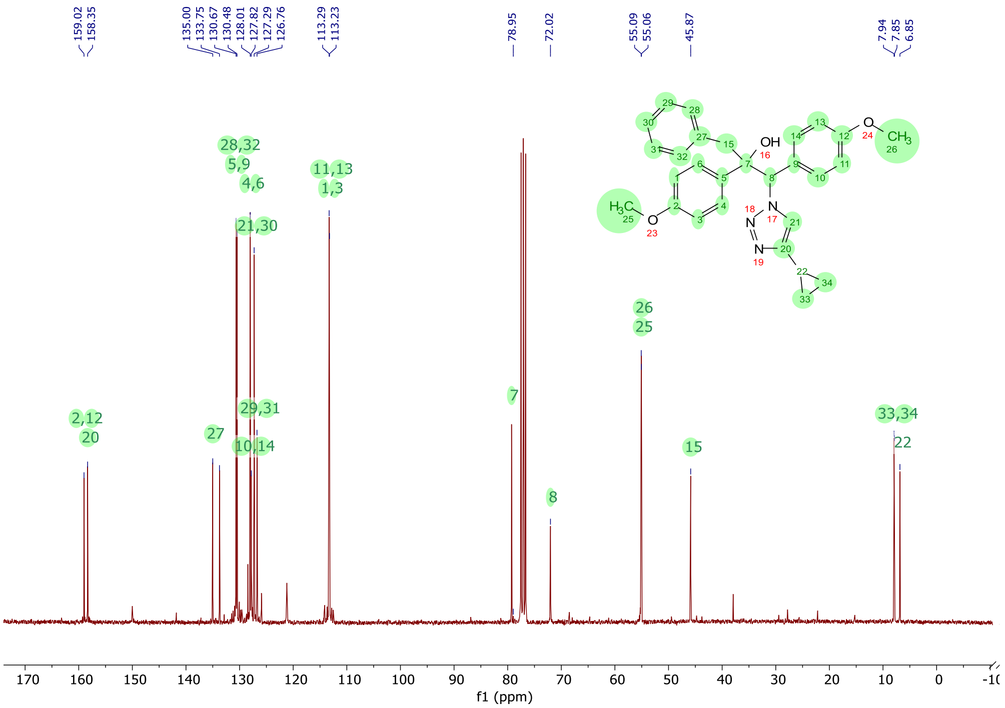

2-{1-[2-Hydroxy-1,2-bis-(4-methoxyphenyl)-3-phenyl-propyl]-[1,2,3]triazol-4-ylmethyl}-isoindole-1,3-dione (43)

^{1}H NMR spectrum

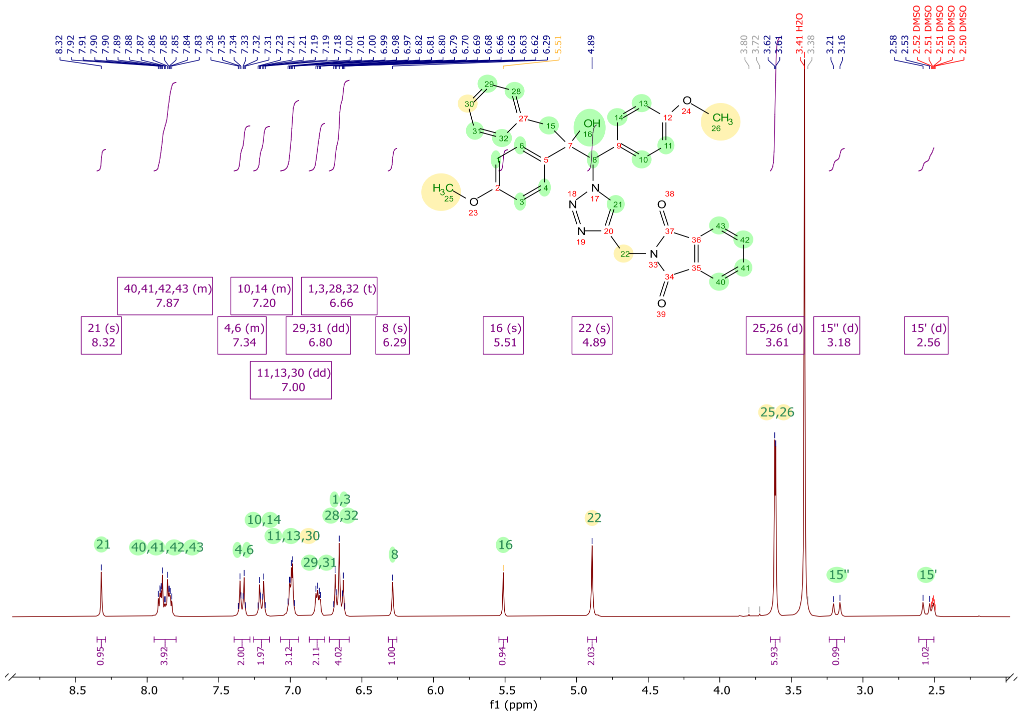

^{13}C NMR spectrum

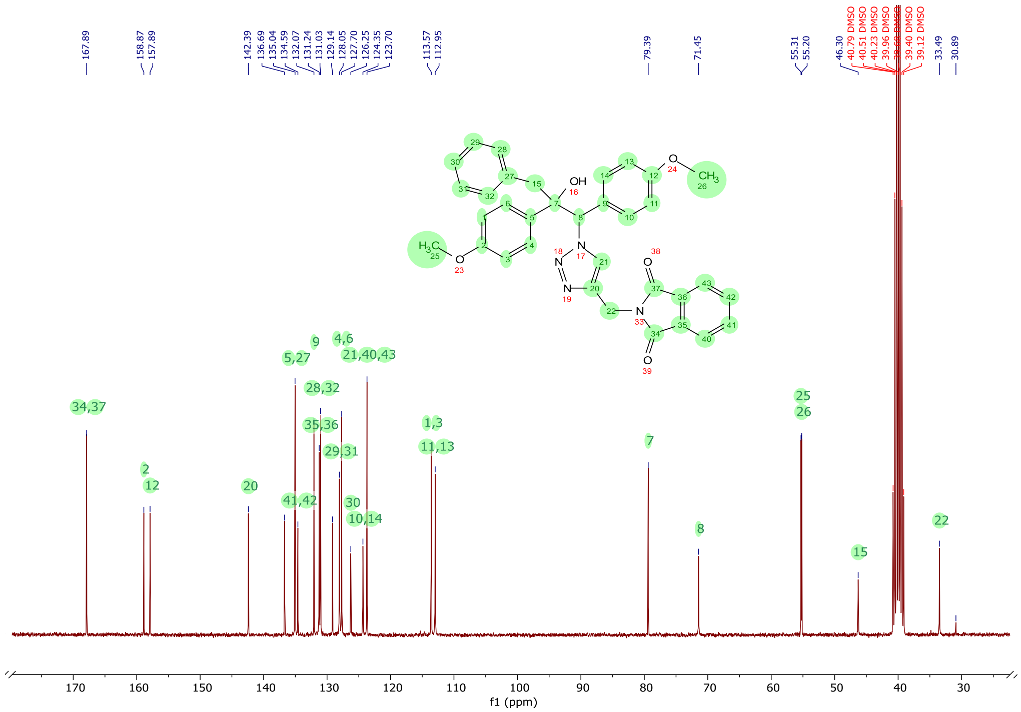

1-[4-(2,6-Diisopropylphenoxymethyl)-[1,2,3]triazol-1-yl]-1,2-bis-(4-methoxy-phenyl)-3-phenyl-propan-2-ol (44)

^{1}H NMR spectrum

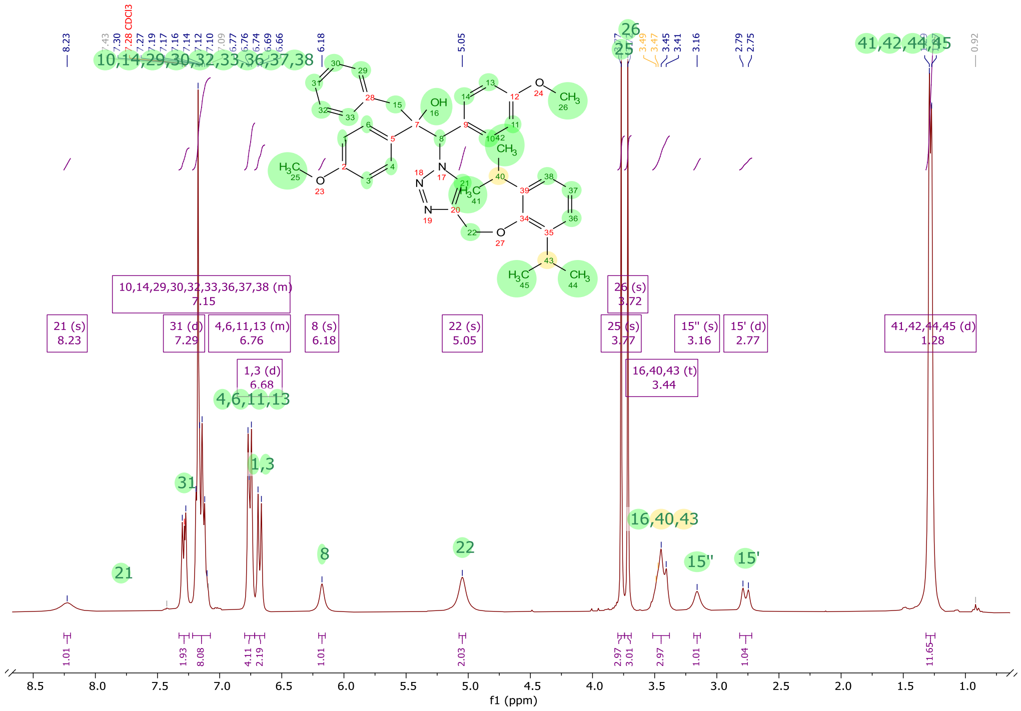

^{13}C NMR spectrum

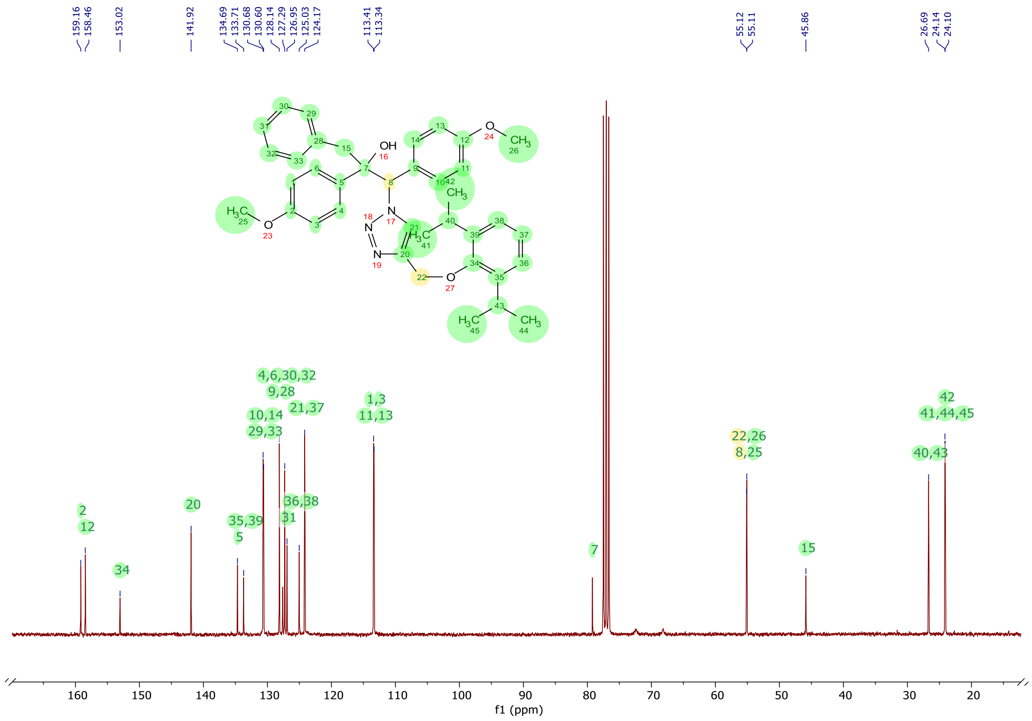

1,2-Bis-(4-methoxyphenyl)-1-(4-morpholin-4-ylmethyl-[1,2,3]triazol-1-yl)-3-phenyl-propan-2-ol (45)

^{1}H NMR spectrum

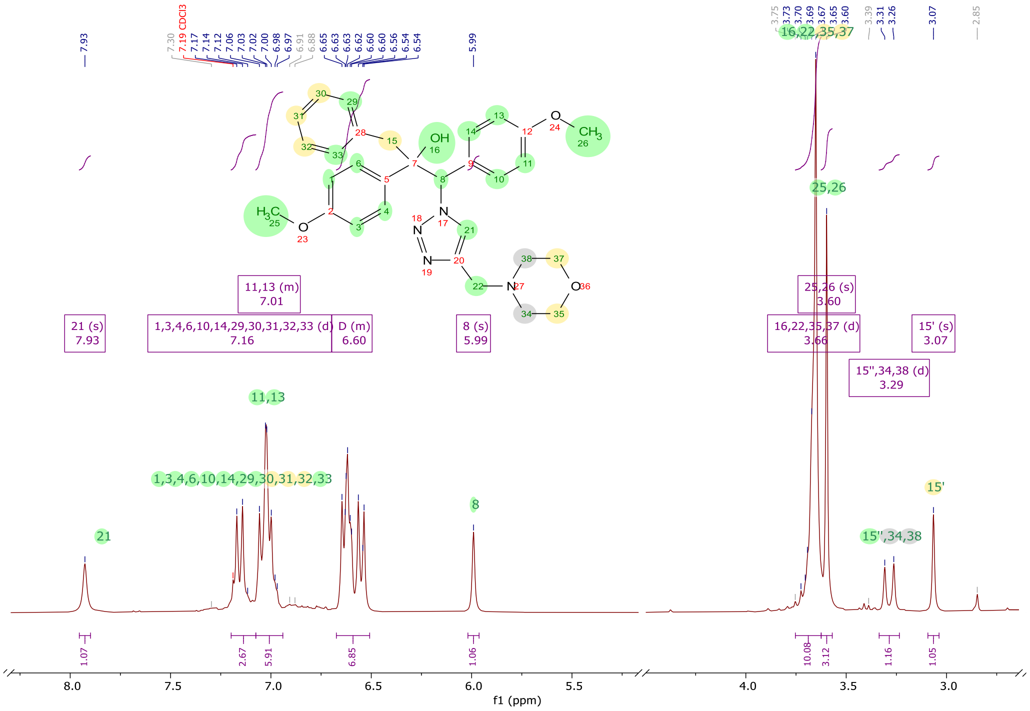

^{13}C NMR spectrum

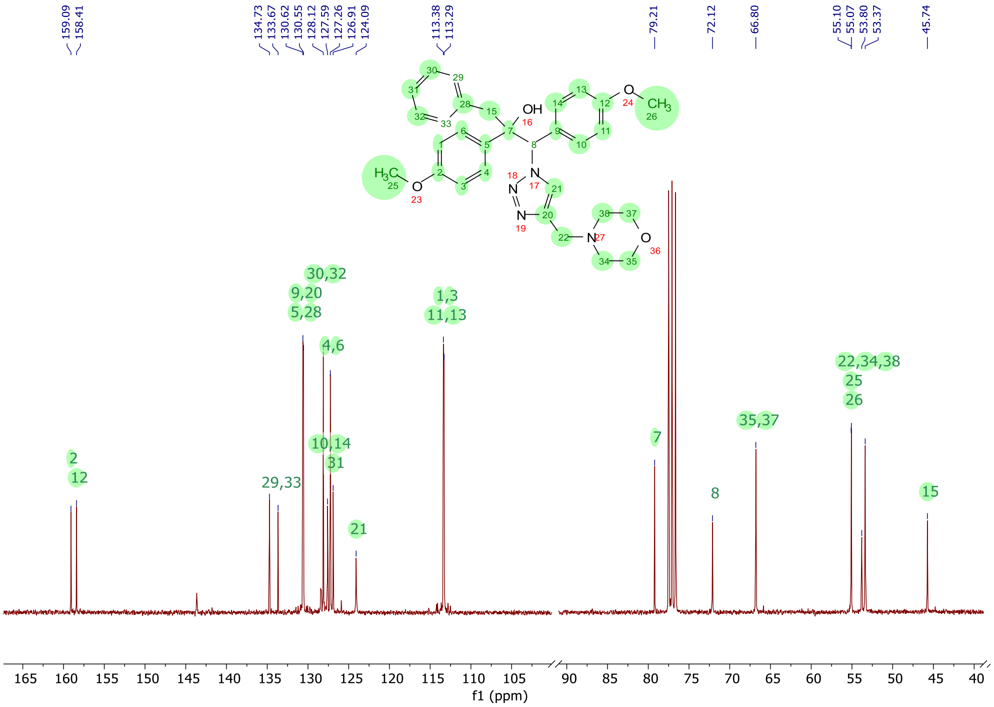

1,2-Bis-(4-methoxyphenyl)-5-methyl-1-(4-phenyl-[1,2,3]triazol-1-yl)-hexan-2-ol (46)

^{1}H NMR spectrum

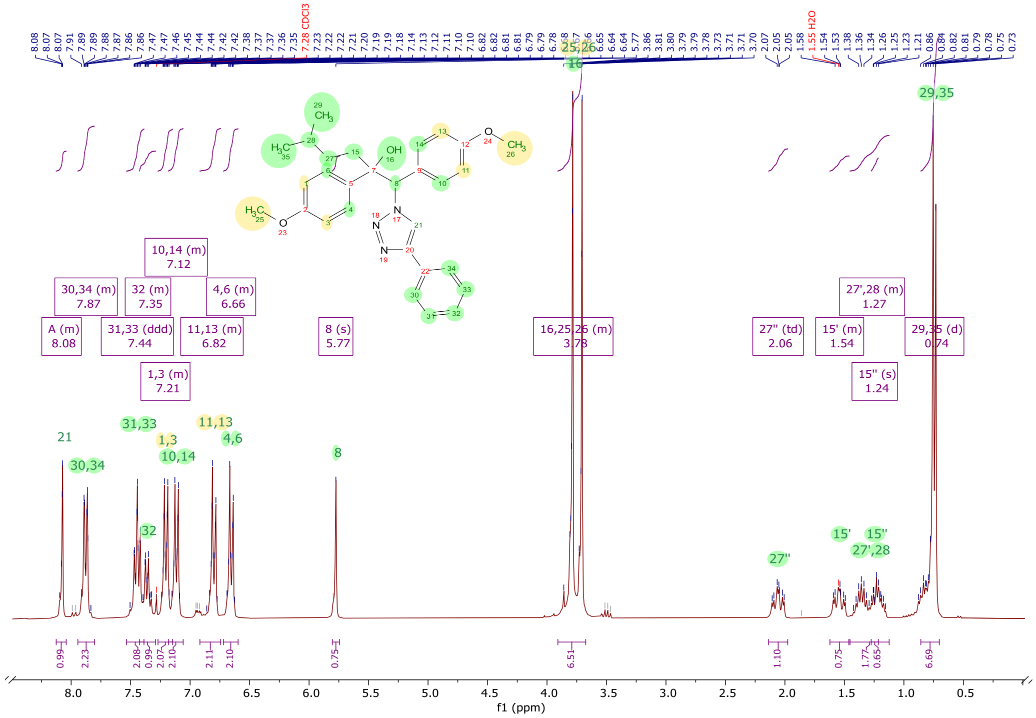

^{13}C NMR spectrum

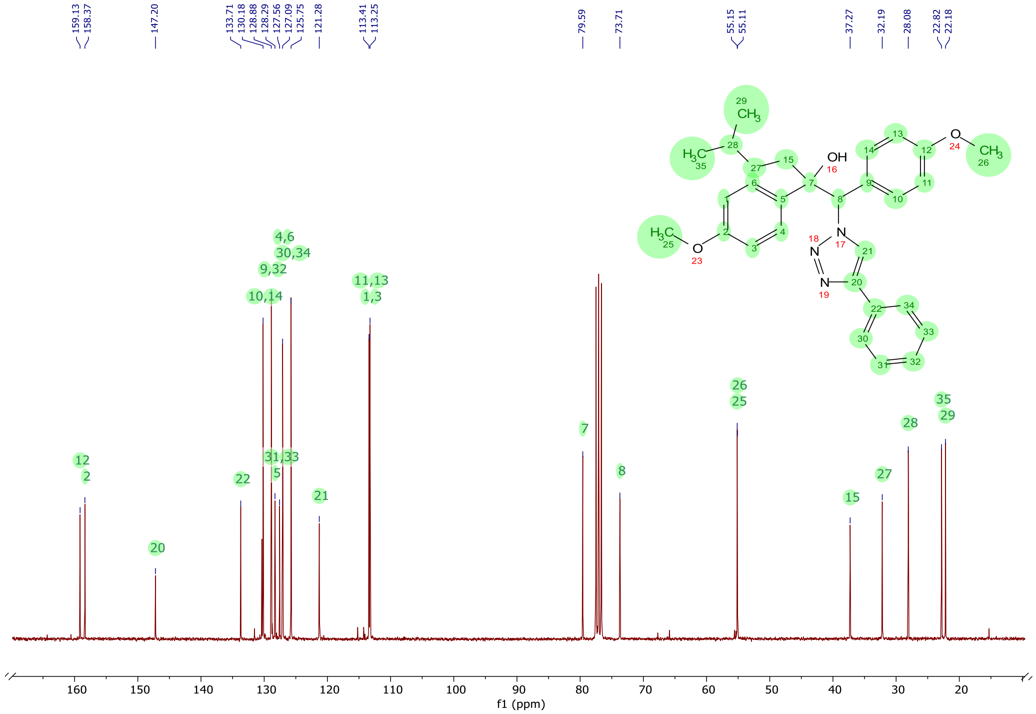

1-(4-Cyclopropyl-[1,2,3]triazol-1-yl)-1,2-bis-(4-methoxyphenyl)-5-methyl-hexan-2-ol (47)

^{1}H NMR spectrum

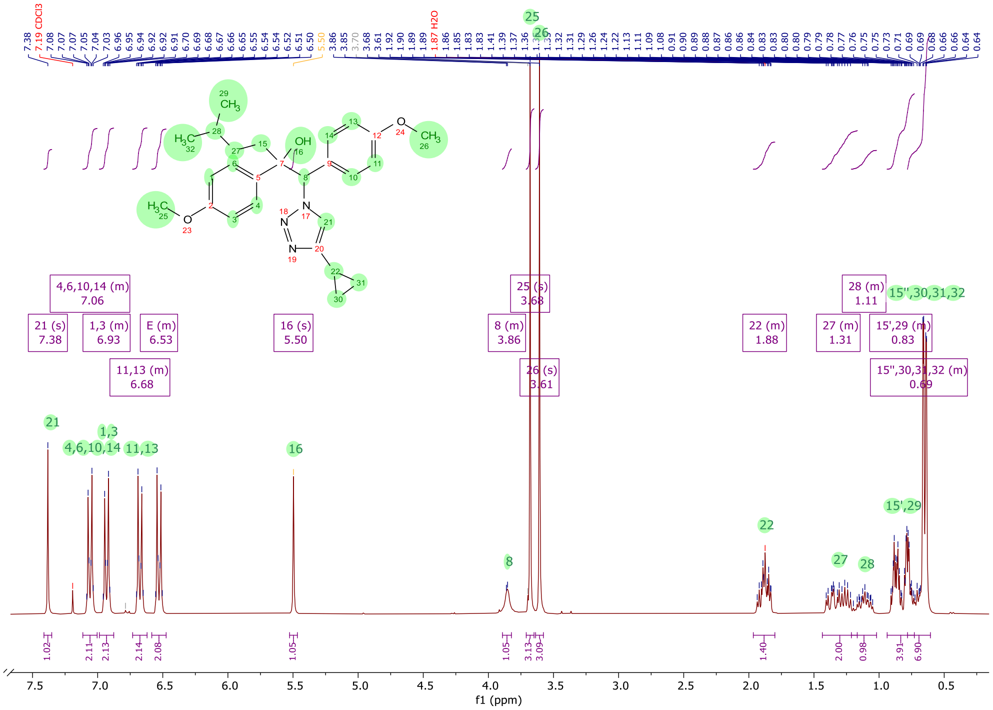

^{13}C NMR spectrum

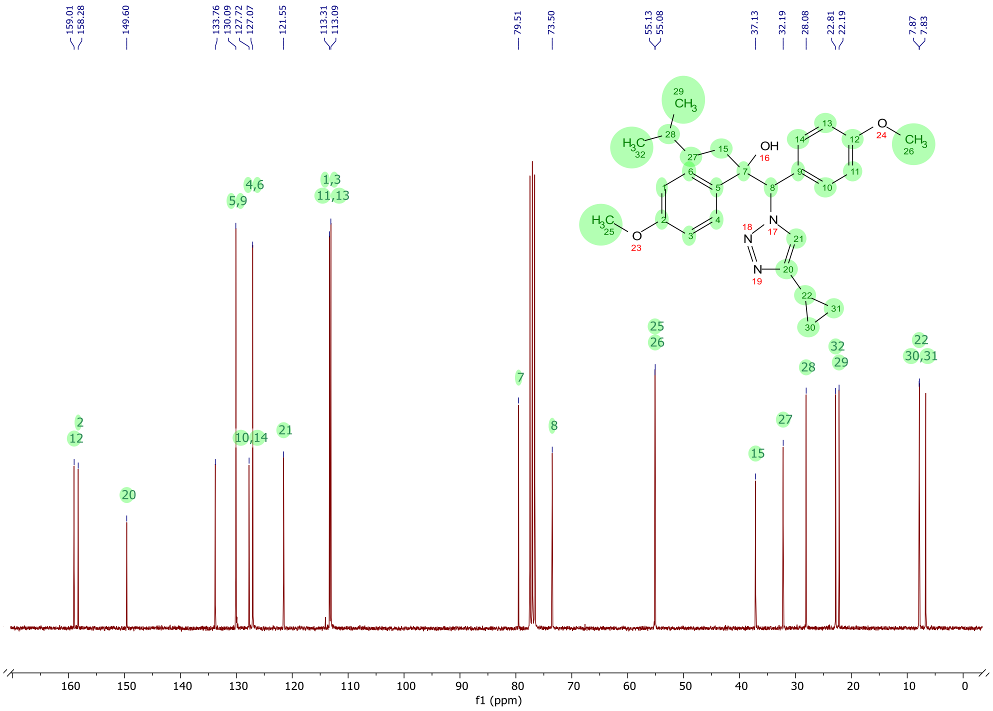

2-{1-[2-Hydroxy-1,2-bis-(4-methoxyphenyl)-5-methyl-hexyl]-[1,2,3]triazol-4-ylmethyl}-isoindole-1,3-dione (48)

^{1}H NMR spectrum

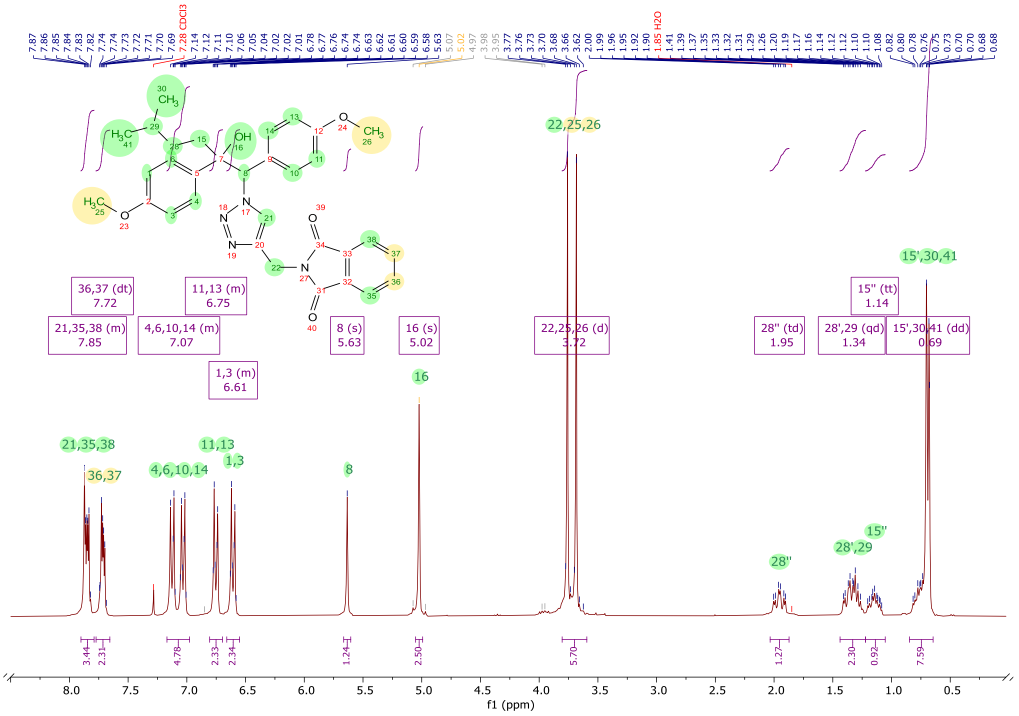

^{13}C NMR spectrum

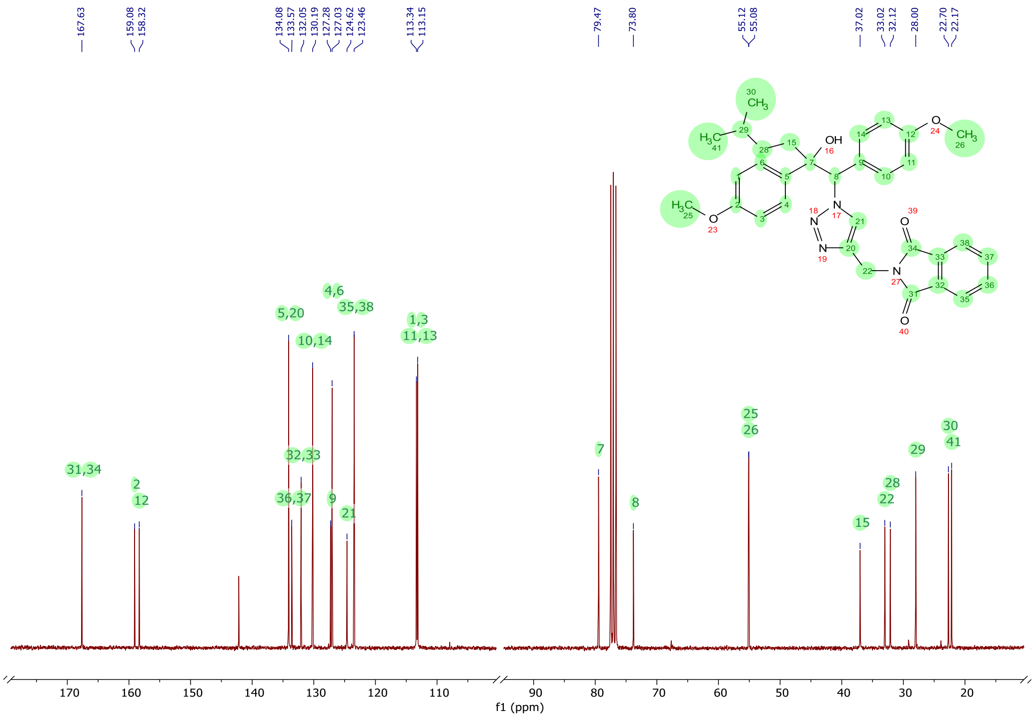

1-[4-(2,6-Diisopropylphenoxymethyl)-[1,2,3]triazol-1-yl]-1,2-bis-(4-methoxyphenyl)-5-methyl-hexan-2-ol (49)

^{1}H NMR spectrum

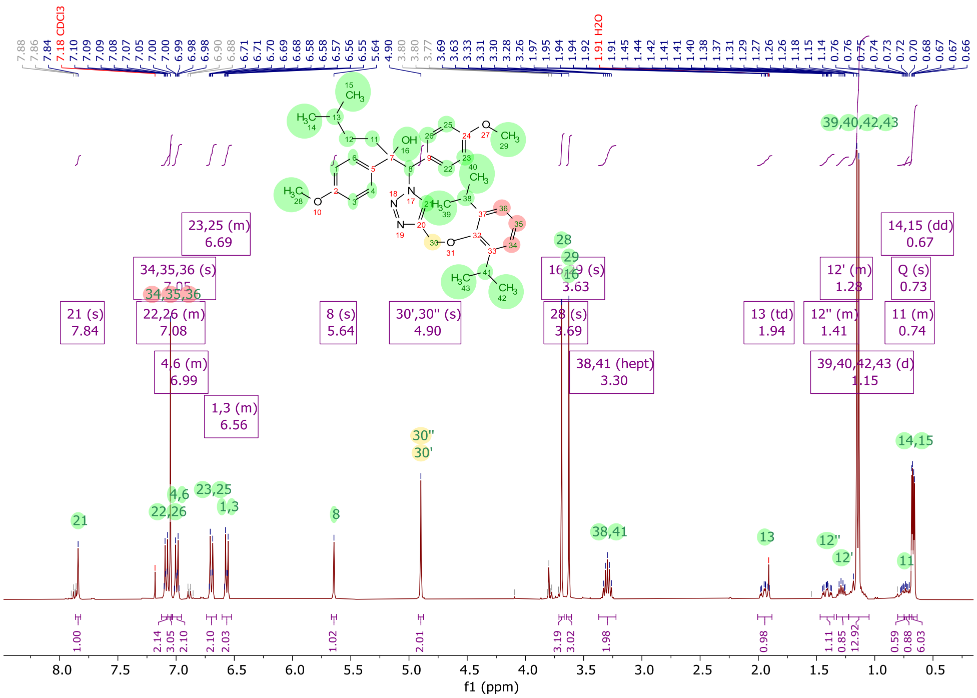

^{13}C NMR spectrum

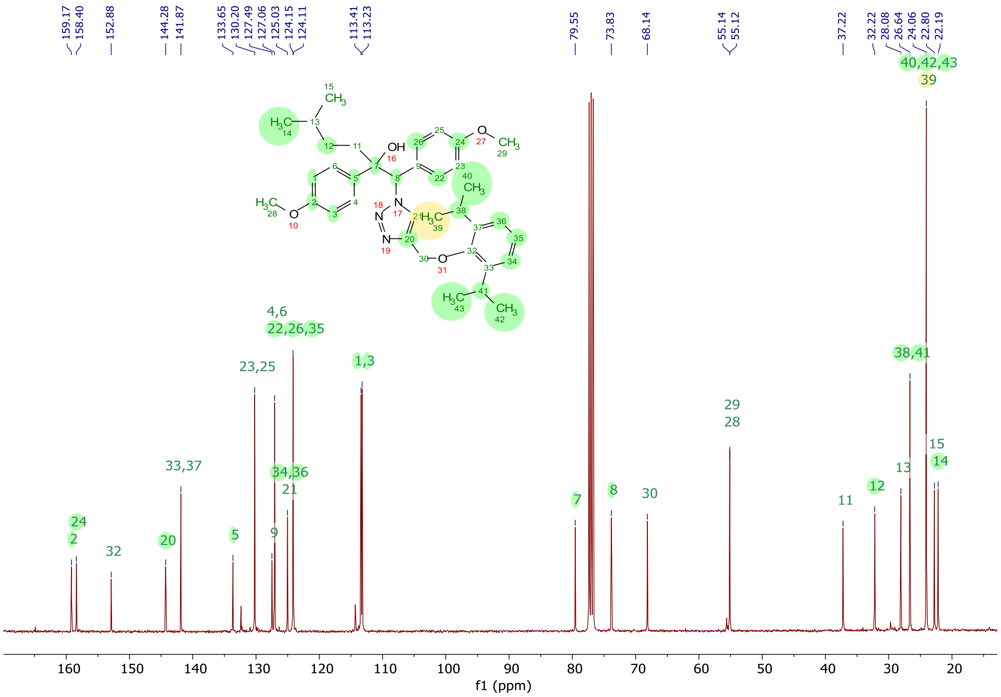

1,2-Bis-(4-methoxyphenyl)-5-methyl-1-(4-morpholin-4-ylmethyl-[1,2,3]triazol-1-yl)-hexan-2-ol (50)

^{1}H NMR spectrum

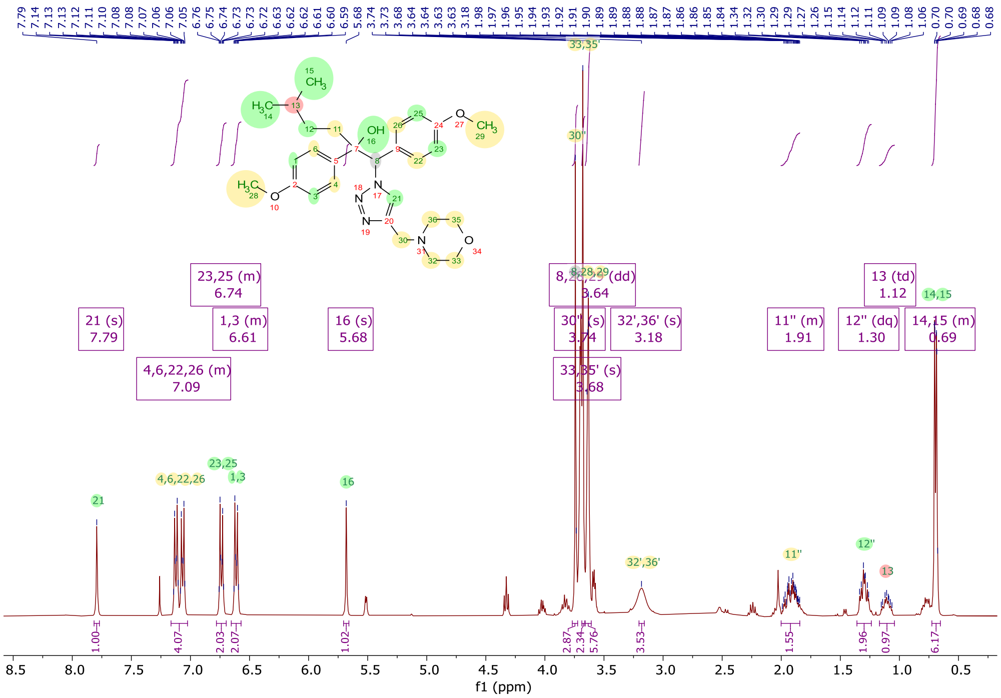

^{13}C NMR spectrum

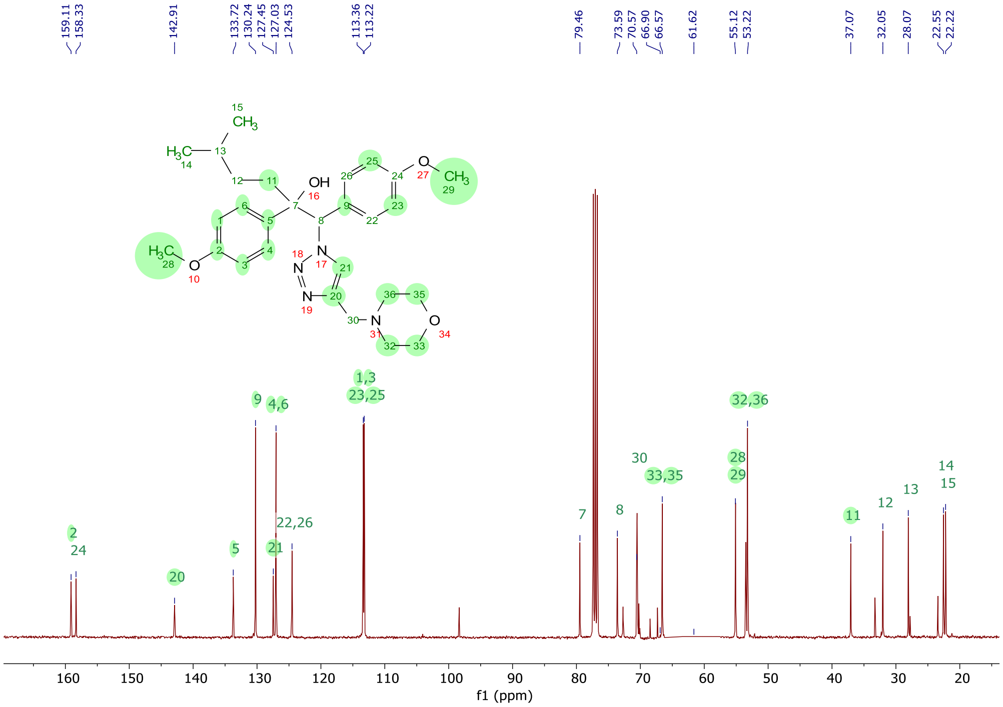

Supplement: Supplementary file 1 [file molecules-31-00170-s001.zip › molecules-3995893-supplementary.pdf]
